# Supplementary material for: Phanerosides A–X, Phenylpropanoid Esters of Sucrose from the Rattans of Phanera championii Benth
Source: Molecules. 2023 Jun 14;28(12):4767. doi: 10.3390/molecules28124767 (PMC10301492; doi:10.3390/molecules28124767)
Supplement: Supplementary file 1 [file molecules-28-04767-s001.zip › molecules-2416984-supplementary.pdf]

Phanerosides A–X, Phenylpropanoid Esters of Sucrose from the Rattans of *Phanera championii* Benth.

Ya-Jie Hu, Qian Lan, Bao-Jun Su, and Dong Liang*

State Key Laboratory for Chemistry and Molecular Engineering of Medicinal Resources, Collaborative Innovation Center for Guangxi Ethnic Medicine, School of Chemistry and Pharmaceutical Sciences, Guangxi Normal University, Guilin 541004, People's Republic of China

* Correspondence: liangdong@mailbox.gxnu.edu.cn, liangdonggxnu@163.com (D.L.)

Figures of Contents

Figure S1. UV Spectrum of Compound **1**........10

Figure S2. IR (KBr disc) Spectrum of Compound **1**........10

Figure S3. (+) HRESIMS Spectrum of Compound **1**........11

Figure S4. ^{1}H NMR Spectrum of Compound **1** in MeOH-d_4........11

Figure S5. ^{13}C NMR Spectrum of Compound **1** in MeOH-d_4........12

Figure S6. ^{1}H–^{1}H COSY Spectrum of Compound **1** in MeOH-d_4........12

Figure S7. HSQC Spectrum of Compound **1** in MeOH-d_4........13

Figure S8. HMBC Spectrum of Compound **1** in MeOH-d_4........13

Figure S9. NOESY Spectrum of Compound **1** in MeOH-d_4........14

Figure S10. Chiral-HPLC profile from acid hydrolysis of **1** compared to authentic standard 14

Figure S11. UV Spectrum of Compound **2**........15

Figure S12. IR (KBr disc) Spectrum of Compound **2**........15

Figure S13. (+) HRESIMS Spectrum of Compound **2**........16

Figure S14. ^{1}H NMR Spectrum of Compound **2** in MeOH-d_4........16

Figure S15. ^{13}C NMR Spectrum of Compound **2** in MeOH-d_4........17

Figure S16. ^{1}H–^{1}H COSY Spectrum of Compound **2** in MeOH-d_4........17

Figure S17. HSQC Spectrum of Compound **2** in MeOH-d_4........18

Figure S18. HMBC Spectrum of Compound **2** in MeOH-d_4........18

Figure S19. NOESY Spectrum of Compound **2** in MeOH-d_4........19

Figure S20. UV Spectrum of Compound **3**........19

Figure S21. IR (KBr disc) Spectrum of Compound **3**........20

Figure S22. (+) HRESIMS Spectrum of Compound **3**........20

Figure S23. ^{1}H NMR Spectrum of Compound **3** in MeOH-d_4........21

Figure S24. ^{13}C NMR Spectrum of Compound **3** in MeOH-d_4........21

Figure S25. ^{1}H–^{1}H COSY Spectrum of Compound **3** in MeOH-d_4........22

Figure S26. HSQC Spectrum of Compound **3** in MeOH-d_4........22

Figure S27. HMBC Spectrum of Compound **3** in MeOH-d_4........23

Figure S28. NOESY Spectrum of Compound **3** in MeOH-d_4........23

Figure S29. UV Spectrum of Compound **4**........24

Figure S30. IR (KBr disc) Spectrum of Compound **4**....24
Figure S31. (+) HRESIMS Spectrum of Compound **4**....25
Figure S32. ^{1}H NMR Spectrum of Compound **4** in MeOH-d_4....25
Figure S33. ^{13}C NMR Spectrum of Compound **4** in MeOH-d_4....26
Figure S34. ^{1}H–^{1}H COSY Spectrum of Compound **4** in MeOH-d_4....26
Figure S35. HSQC Spectrum of Compound **4** in MeOH-d_4....27
Figure S36. HMBC Spectrum of Compound **4** in MeOH-d_4....27
Figure S37. NOESY Spectrum of Compound **4** in MeOH-d_4....28
Figure S38. UV Spectrum of Compound **5**....28
Figure S39. IR (KBr disc) Spectrum of Compound **5**....29
Figure S40. (+) HRESIMS Spectrum of Compound **5**....29
Figure S41. ^{1}H NMR Spectrum of Compound **5** in MeOH-d_4....30
Figure S42. ^{13}C NMR Spectrum of Compound **5** in MeOH-d_4....30
Figure S43. ^{1}H–^{1}H COSY Spectrum of Compound **5** in MeOH-d_4....31
Figure S44. HSQC Spectrum of Compound **5** in MeOH-d_4....31
Figure S45. HMBC Spectrum of Compound **5** in MeOH-d_4....32
Figure S46. NOESY Spectrum of Compound **5** in MeOH-d_4....32
Figure S47. UV Spectrum of Compound **6**....33
Figure S48. IR (KBr disc) Spectrum of Compound **6**....33
Figure S49. (+) HRESIMS Spectrum of Compound **6**....34
Figure S50. ^{1}H NMR Spectrum of Compound **6** in MeOH-d_4....34
Figure S51. ^{13}C NMR Spectrum of Compound **6** in MeOH-d_4....35
Figure S52. ^{1}H–^{1}H COSY Spectrum of Compound **6** in MeOH-d_4....35
Figure S53. HSQC Spectrum of Compound **6** in MeOH-d_4....36
Figure S54. HMBC Spectrum of Compound **6** in MeOH-d_4....36
Figure S55. NOESY Spectrum of Compound **6** in MeOH-d_4....37
Figure S56. UV Spectrum of Compound **7**....37
Figure S57. IR (KBr disc) Spectrum of Compound **7**....38
Figure S58. (+) HRESIMS Spectrum of Compound **7**....38
Figure S59. ^{1}H NMR Spectrum of Compound **7** in MeOH-d_4....39
Figure S60. ^{13}C NMR Spectrum of Compound **7** in MeOH-d_4....39

Figure S61. ^{1}H–^{1}H COSY Spectrum of Compound **7** in MeOH-d_4 40
Figure S62. HSQC Spectrum of Compound **7** in MeOH-d_4 40
Figure S64. NOESY Spectrum of Compound **7** in MeOH-d_4 41
Figure S65. UV Spectrum of Compound **8** 42
Figure S66. IR (KBr disc) Spectrum of Compound **8** 42
Figure S67. (+) HRESIMS Spectrum of Compound **8** 43
Figure S68. ^{1}H NMR Spectrum of Compound **8** in MeOH-d_4 43
Figure S69. ^{13}C NMR Spectrum of Compound **8** in MeOH-d_4 44
Figure S70. ^{1}H–^{1}H COSY Spectrum of Compound **8** in MeOH-d_4 44
Figure S71. HSQC Spectrum of Compound **8** in MeOH-d_4 45
Figure S72. HMBC Spectrum of Compound **8** in MeOH-d_4 45
Figure S73. NOESY Spectrum of Compound **8** in MeOH-d_4 46
Figure S74. UV Spectrum of Compound **9** 46
Figure S75. IR (KBr disc) Spectrum of Compound **9** 47
Figure S76. (+) HRESIMS Spectrum of Compound **9** 47
Figure S77. ^{1}H NMR Spectrum of Compound **9** in MeOH-d_4 48
Figure S78. ^{13}C NMR Spectrum of Compound **9** in MeOH-d_4 48
Figure S79. ^{1}H–^{1}H COSY Spectrum of Compound **9** in MeOH-d_4 49
Figure S80. HSQC Spectrum of Compound **9** in MeOH-d_4 49
Figure S81. HMBC Spectrum of Compound **9** in MeOH-d_4 50
Figure S82. NOESY Spectrum of Compound **9** in MeOH-d_4 50
Figure S83. UV Spectrum of Compound **10** 51
Figure S84. IR (KBr disc) Spectrum of Compound **10** 51
Figure S85. (+) HRESIMS Spectrum of Compound **10** 52
Figure S86. ^{1}H NMR Spectrum of Compound **10** in MeOH-d_4 52
Figure S87. ^{13}C NMR Spectrum of Compound **10** in MeOH-d_4 53
Figure S88. ^{1}H–^{1}H COSY Spectrum of Compound **10** in MeOH-d_4 53
Figure S89. HSQC Spectrum of Compound **10** in MeOH-d_4 54
Figure S90. HMBC Spectrum of Compound **10** in MeOH-d_4 54
Figure S91. NOESY Spectrum of Compound **10** in MeOH-d_4 55
Figure S92. UV Spectrum of Compound **11** 55

Figure S93. IR (KBr disc) Spectrum of Compound **11**....56
Figure S94. (+) HRESIMS Spectrum of Compound **11**....56
Figure S95. ^{1}H NMR Spectrum of Compound **11** in MeOH-d_4....57
Figure S96. ^{13}C NMR Spectrum of Compound **11** in MeOH-d_4....57
Figure S97. ^{1}H–^{1}H COSY Spectrum of Compound **11** in MeOH-d_4....58
Figure S98. HSQC Spectrum of Compound **11** in MeOH-d_4....58
Figure S99. HMBC Spectrum of Compound **11** in MeOH-d_4....59
Figure S100. NOESY Spectrum of Compound **11** in MeOH-d_4....59
Figure S101. UV Spectrum of Compound **12**....60
Figure S102. IR (KBr disc) Spectrum of Compound **12**....60
Figure S103. (+) HRESIMS Spectrum of Compound **12**....61
Figure S104. ^{1}H NMR Spectrum of Compound **12** in MeOH-d_4....61
Figure S105. ^{13}C NMR Spectrum of Compound **12** in MeOH-d_4....62
Figure S106. ^{1}H–^{1}H COSY Spectrum of Compound **12** in MeOH-d_4....62
Figure S107. HSQC Spectrum of Compound **12** in MeOH-d_4....63
Figure S108. HMBC Spectrum of Compound **12** in MeOH-d_4....63
Figure S109. NOESY Spectrum of Compound **12** in MeOH-d_4....64
Figure S110. UV Spectrum of Compound **13**....64
Figure S111. IR (KBr disc) Spectrum of Compound **13**....65
Figure S112. (+) HRESIMS Spectrum of Compound **13**....65
Figure S113. ^{1}H NMR Spectrum of Compound **13** in MeOH-d_4....66
Figure S114. ^{13}C NMR Spectrum of Compound **13** in MeOH-d_4....66
Figure S115. ^{1}H–^{1}H COSY Spectrum of Compound **13** in MeOH-d_4....67
Figure S116. HSQC Spectrum of Compound **13** in MeOH-d_4....67
Figure S117. HMBC Spectrum of Compound **13** in MeOH-d_4....68
Figure S118. NOESY Spectrum of Compound **13** in MeOH-d_4....68
Figure S119. UV Spectrum of Compound **14**....69
Figure S120. IR (KBr disc) Spectrum of Compound **14**....69
Figure S121. (+) HRESIMS Spectrum of Compound **14**....70
Figure S122. ^{1}H NMR Spectrum of Compound **14** in MeOH-d_4....70
Figure S123. ^{13}C NMR Spectrum of Compound **14** in MeOH-d_4....71

Figure S124. ^1H–^1H COSY Spectrum of Compound **14** in MeOH-d_4 71
Figure S125. HSQC Spectrum of Compound **14** in MeOH-d_4 72
Figure S126. HMBC Spectrum of Compound **14** in MeOH-d_4 72
Figure S127. NOESY Spectrum of Compound **14** in MeOH-d_4 73
Figure S128. UV Spectrum of Compound **15** 73
Figure S129. IR (KBr disc) Spectrum of Compound **15** 74
Figure S130. (+) HRESIMS Spectrum of Compound **15** 74
Figure S131. ^1H NMR Spectrum of Compound **15** in MeOH-d_4 75
Figure S132. ^{13}C NMR Spectrum of Compound **15** in MeOH-d_4 75
Figure S133. ^1H–^1H COSY Spectrum of Compound **15** in MeOH-d_4 76
Figure S134. HSQC Spectrum of Compound **15** in MeOH-d_4 76
Figure S135. HMBC Spectrum of Compound **15** in MeOH-d_4 77
Figure S136. NOESY Spectrum of Compound **15** in MeOH-d_4 77
Figure S137. UV Spectrum of Compound **16** 78
Figure S138. IR (KBr disc) Spectrum of Compound **16** 78
Figure S139. (+) HRESIMS Spectrum of Compound **16** 79
Figure S140. ^1H NMR Spectrum of Compound **16** in MeOH-d_4 79
Figure S141. ^{13}C NMR Spectrum of Compound **16** in MeOH-d_4 80
Figure S142. ^1H–^1H COSY Spectrum of Compound **16** in MeOH-d_4 80
Figure S143. HSQC Spectrum of Compound **16** in MeOH-d_4 81
Figure S144. HMBC Spectrum of Compound **16** in MeOH-d_4 81
Figure S145. NOESY Spectrum of Compound **16** in MeOH-d_4 82
Figure S146. UV Spectrum of Compound **17** 82
Figure S147. IR (KBr disc) Spectrum of Compound **17** 83
Figure S148. (+) HRESIMS Spectrum of Compound **17** 83
Figure S149. ^1H NMR Spectrum of Compound **17** in MeOH-d_4 84
Figure S150. ^{13}C NMR Spectrum of Compound **17** in MeOH-d_4 84
Figure S151. ^1H–^1H COSY Spectrum of Compound **17** in MeOH-d_4 85
Figure S152. HSQC Spectrum of Compound **17** in MeOH-d_4 85
Figure S153. HMBC Spectrum of Compound **17** in MeOH-d_4 86
Figure S154. NOESY Spectrum of Compound **17** in MeOH-d_4 86

Figure S155. UV Spectrum of Compound **18**...87
Figure S156. IR (KBr disc) Spectrum of Compound **18**...87
Figure S157. (+) HRESIMS Spectrum of Compound **18** 88
Figure S158. ^{1}H NMR Spectrum of Compound **18** in MeOH-d_4 88
Figure S159. ^{13}C NMR Spectrum of Compound **18** in MeOH-d_4 89
Figure S160. ^{1}H–^{1}H COSY Spectrum of Compound **18** in MeOH-d_4 89
Figure S161. HSQC Spectrum of Compound **18** in MeOH-d_4..................................90
Figure S162. HMBC Spectrum of Compound **18** in MeOH-d_4..................................90
Figure S163. NOESY Spectrum of Compound **18** in MeOH-d_4 91
Figure S164. UV Spectrum of Compound **19**...91
Figure S165. IR (KBr disc) Spectrum of Compound **19**...92
Figure S166. (+) HRESIMS Spectrum of Compound **19** 92
Figure S167. ^{1}H NMR Spectrum of Compound **19** in MeOH-d_4 93
Figure S168. ^{13}C NMR Spectrum of Compound **19** in MeOH-d_4 93
Figure S169. ^{1}H–^{1}H COSY Spectrum of Compound **19** in MeOH-d_4 94
Figure S170. HSQC Spectrum of Compound **19** in MeOH-d_4..................................94
Figure S171. HMBC Spectrum of Compound **19** in MeOH-d_4..................................95
Figure S172. NOESY Spectrum of Compound **19** in MeOH-d_4 95
Figure S173. UV Spectrum of Compound **20**...96
Figure S174. IR (KBr disc) Spectrum of Compound **20**...96
Figure S175. (+) HRESIMS Spectrum of Compound **20** 97
Figure S176. ^{1}H NMR Spectrum of Compound **20** in MeOH-d_4 97
Figure S177. ^{13}C NMR Spectrum of Compound **20** in MeOH-d_4 98
Figure S178. ^{1}H–^{1}H COSY Spectrum of Compound **20** in MeOH-d_4 98
Figure S179. HSQC Spectrum of Compound **20** in MeOH-d_4..................................99
Figure S180. HMBC Spectrum of Compound **20** in MeOH-d_4..................................99
Figure S181. NOESY Spectrum of Compound **20** in MeOH-d_4 100
Figure S182. UV Spectrum of Compound **21**...100
Figure S183. IR (KBr disc) Spectrum of Compound **21**...101
Figure S184. (+) HRESIMS Spectrum of Compound **21** 101
Figure S185. ^{1}H NMR Spectrum of Compound **21** in MeOH-d_4 102

Figure S186. ^{13}C NMR Spectrum of Compound **21** in MeOH-d_4 102
Figure S187. ^{1}H–^{1}H COSY Spectrum of Compound **21** in MeOH-d_4 103
Figure S188. HSQC Spectrum of Compound **21** in MeOH-d_4................................103
Figure S189. HMBC Spectrum of Compound **21** in MeOH-d_4...............................104
Figure S190. NOESY Spectrum of Compound **21** in MeOH-d_4 104
Figure S191. UV Spectrum of Compound **22**..105
Figure S192. IR (KBr disc) Spectrum of Compound **22**..105
Figure S193. (+) HRESIMS Spectrum of Compound **22** 106
Figure S194. ^{1}H NMR Spectrum of Compound **22** in MeOH-d_4 106
Figure S195. ^{13}C NMR Spectrum of Compound **22** in MeOH-d_4 107
Figure S196. ^{1}H–^{1}H COSY Spectrum of Compound **22** in MeOH-d_4 107
Figure S197. HSQC Spectrum of Compound **22** in MeOH-d_4................................108
Figure S198. HMBC Spectrum of Compound **22** in MeOH-d_4...............................108
Figure S199. NOESY Spectrum of Compound **22** in MeOH-d_4 109
Figure S200. UV Spectrum of Compound **23**..109
Figure S201. IR (KBr disc) Spectrum of Compound **23**..110
Figure S202. (+) HRESIMS Spectrum of Compound **23** 110
Figure S203. ^{1}H NMR Spectrum of Compound **23** in MeOH-d_4 111
Figure S204. ^{13}C NMR Spectrum of Compound **23** in MeOH-d_4 111
Figure S205. ^{1}H–^{1}H COSY Spectrum of Compound **23** in MeOH-d_4 112
Figure S206. HSQC Spectrum of Compound **23** in MeOH-d_4................................112
Figure S207. HMBC Spectrum of Compound **23** in MeOH-d_4...............................113
Figure S208. NOESY Spectrum of Compound **23** in MeOH-d_4 113
Figure S209. UV Spectrum of Compound **24**..114
Figure S210. IR (KBr disc) Spectrum of Compound **24**..114
Figure S211. (+) HRESIMS Spectrum of Compound **24** 115
Figure S212. ^{1}H NMR Spectrum of Compound **24** in MeOH-d_4 115
Figure S213. ^{13}C NMR Spectrum of Compound **24** in MeOH-d_4 116
Figure S214. ^{1}H–^{1}H COSY Spectrum of Compound **24** in MeOH-d_4 116
Figure S215. HSQC Spectrum of Compound **24** in MeOH-d_4................................117
Figure S216. HMBC Spectrum of Compound **24** in MeOH-d_4...............................117

Figure S217. NOESY Spectrum of Compound **24** in MeOH-d_4 118

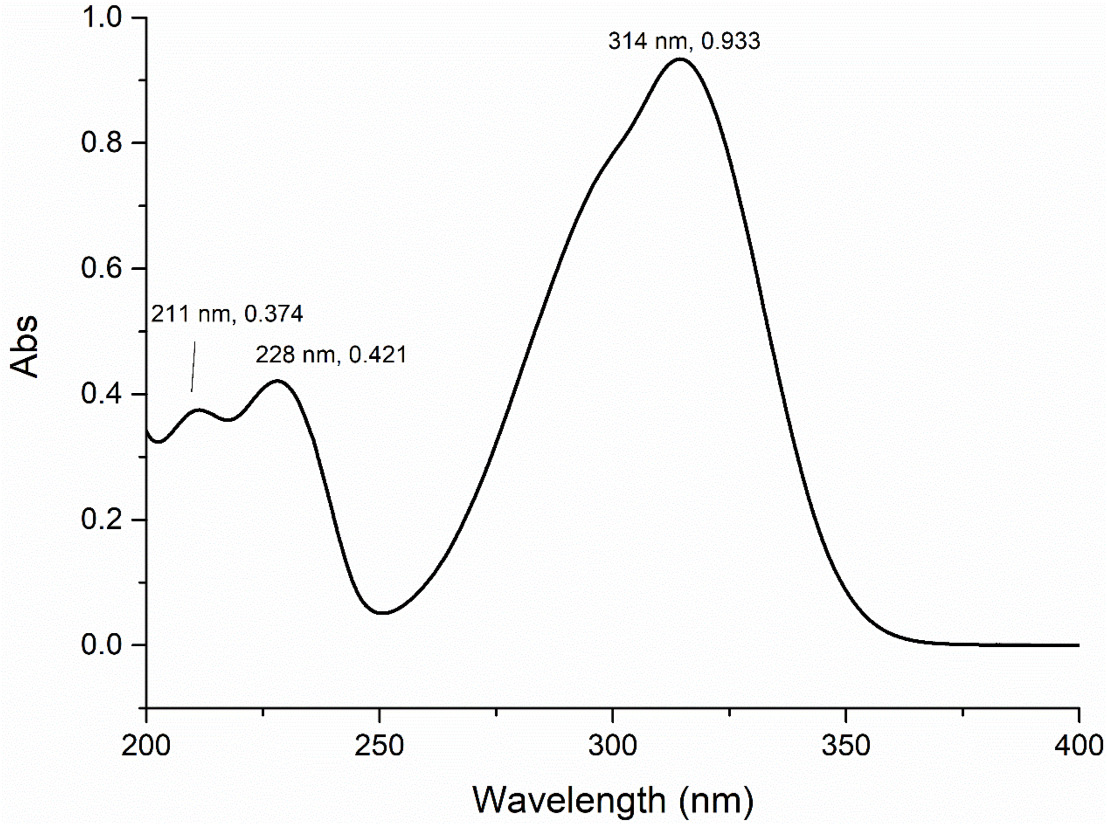

Figure S1. UV Spectrum of Compound **1**

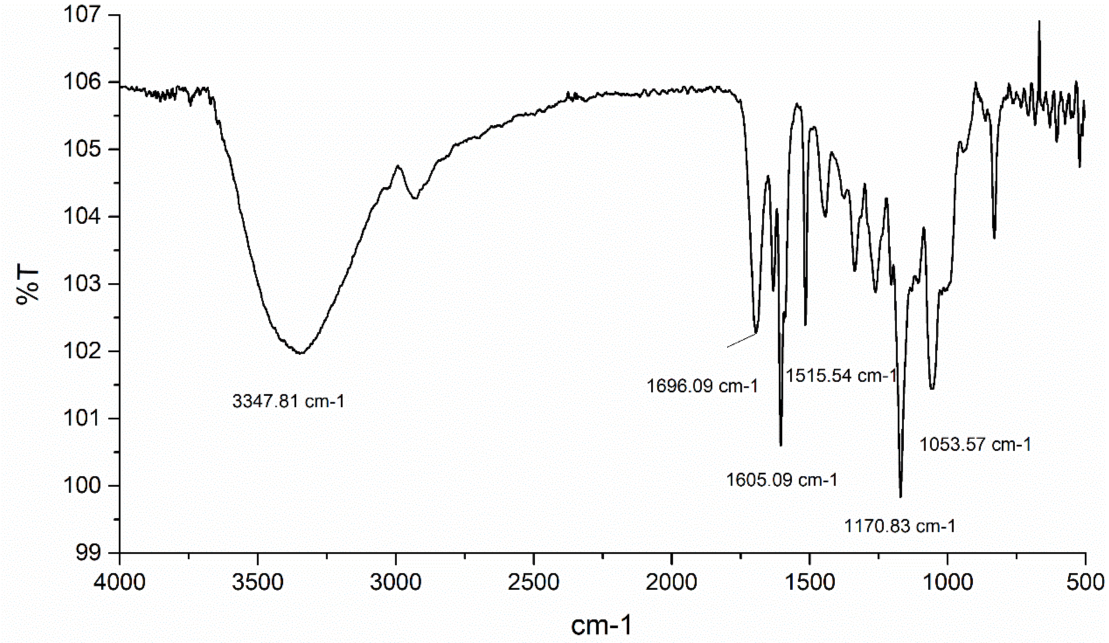

Figure S2. IR (KBr disc) Spectrum of Compound **1**

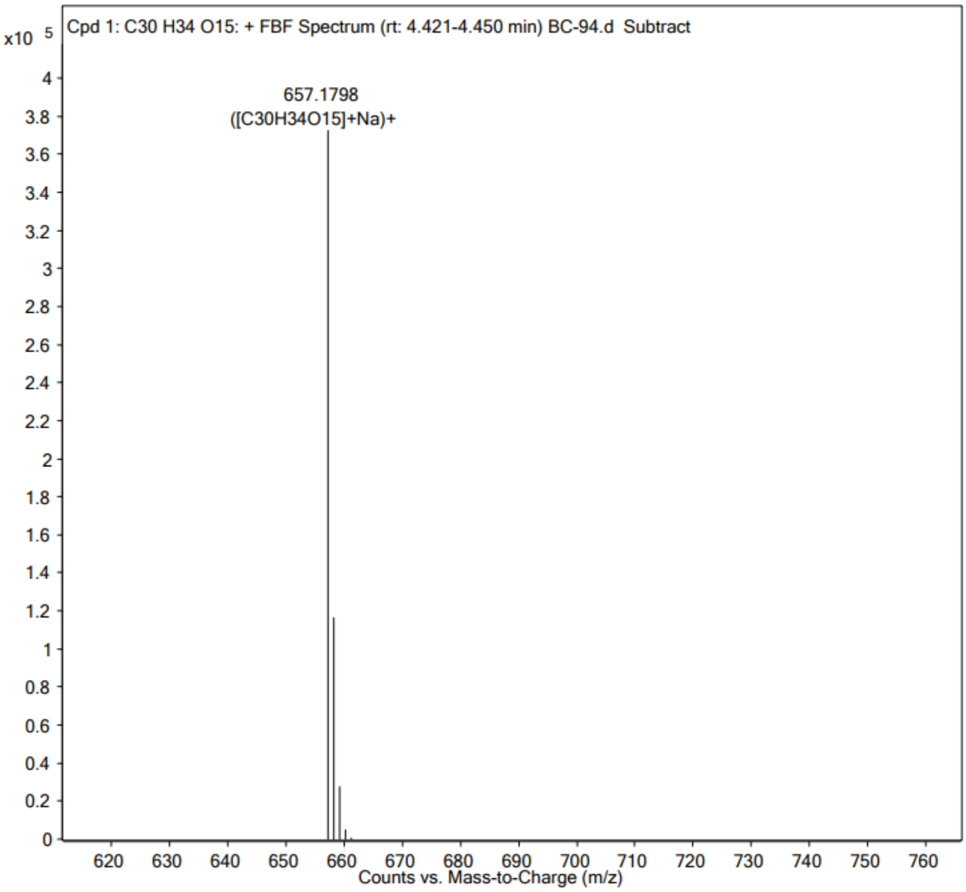

Figure S3. (+) HRESIMS Spectrum of Compound **1**

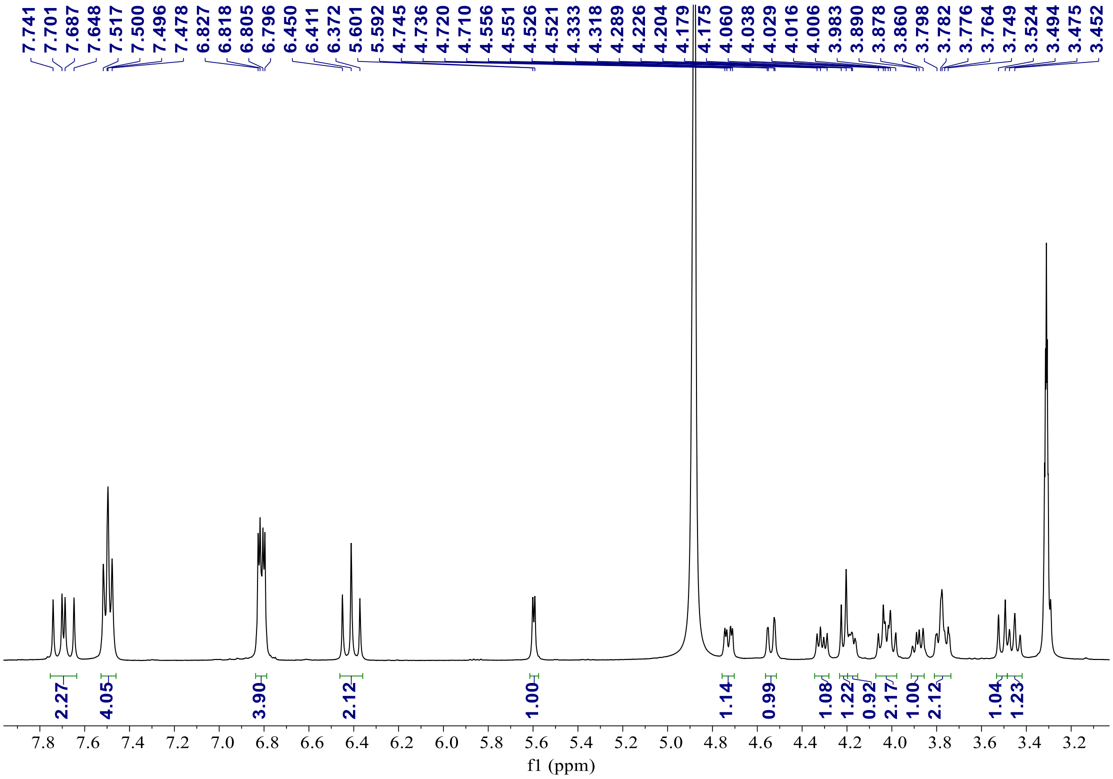

Figure S4. ^{1}H NMR Spectrum of Compound **1** in MeOH-d_4

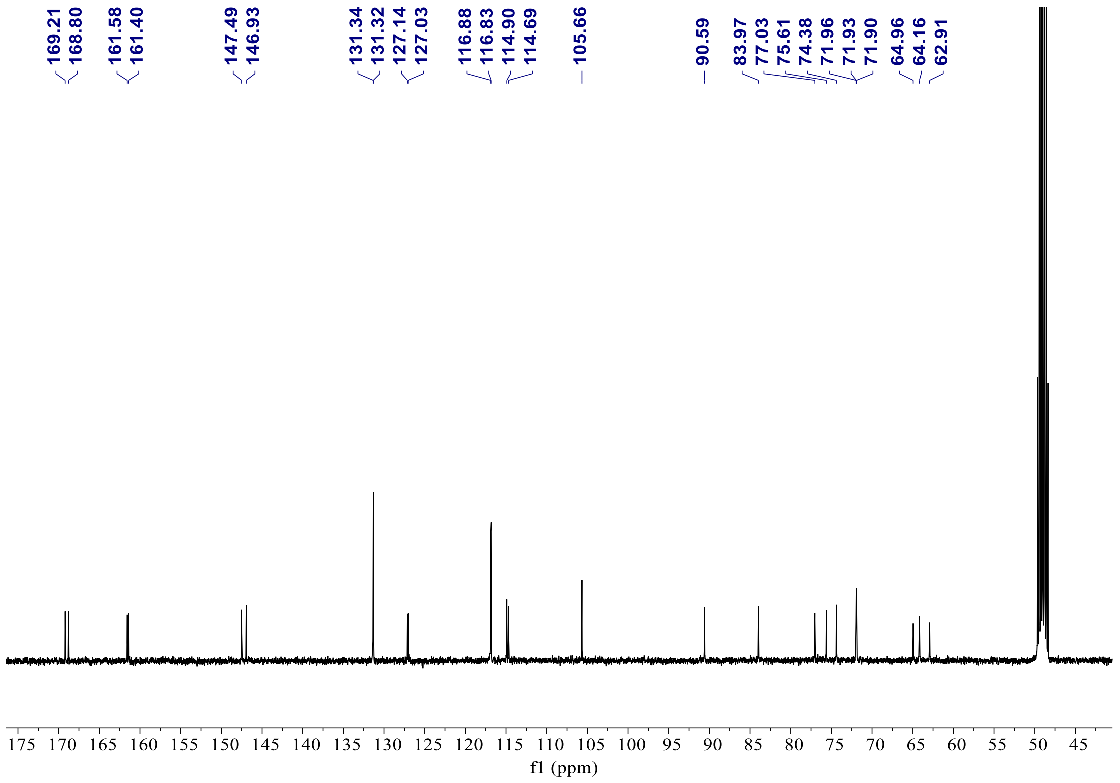

Figure S5. ^{13}C NMR Spectrum of Compound **1** in MeOH-d_4

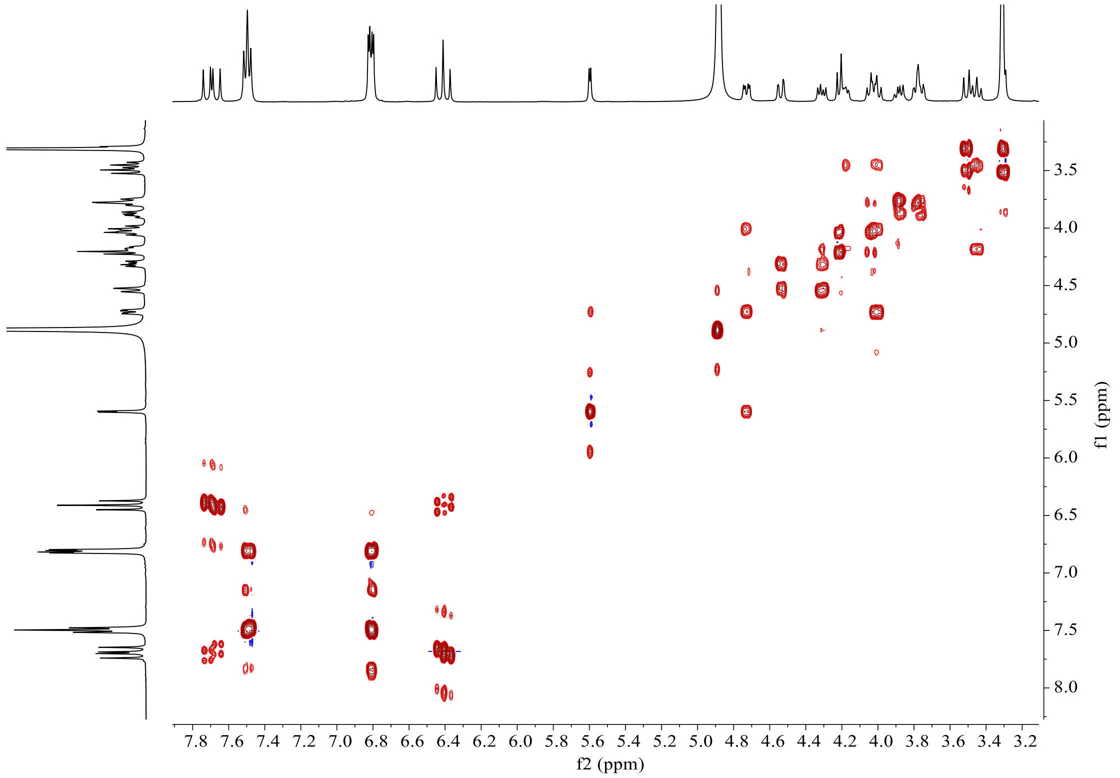

Figure S6. ^{1}H–^{1}H COSY Spectrum of Compound **1** in MeOH-d_4

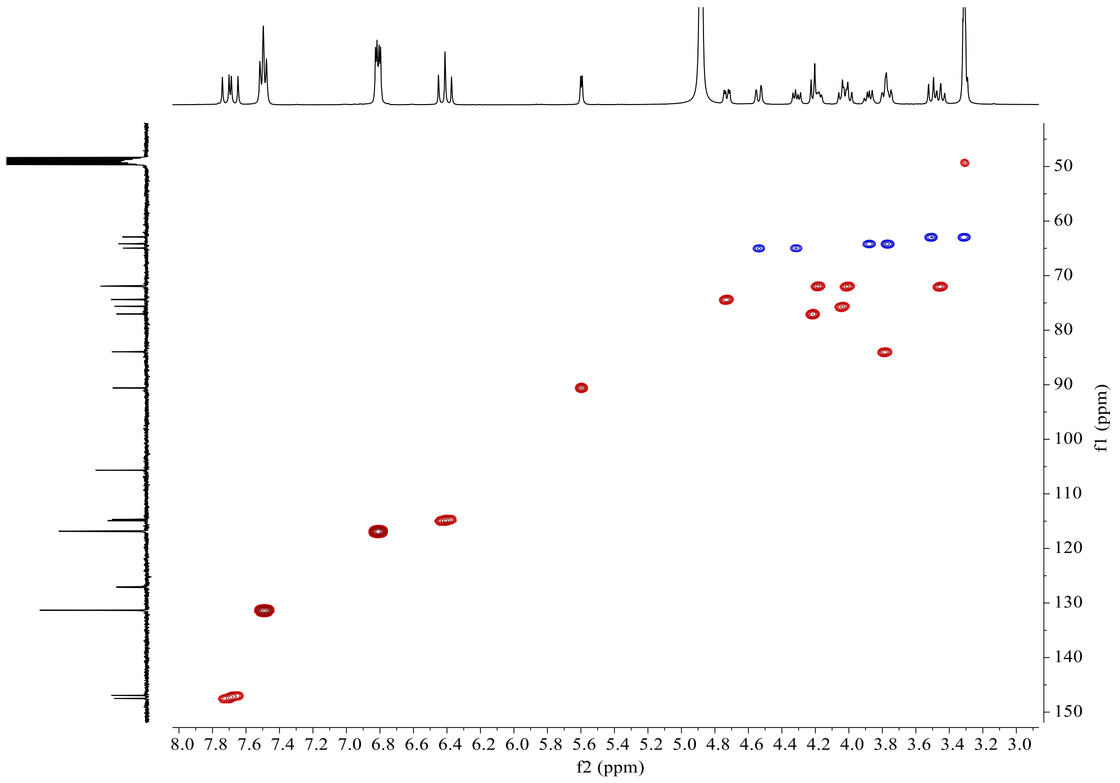

Figure S7. HSQC Spectrum of Compound **1** in MeOH-d_4

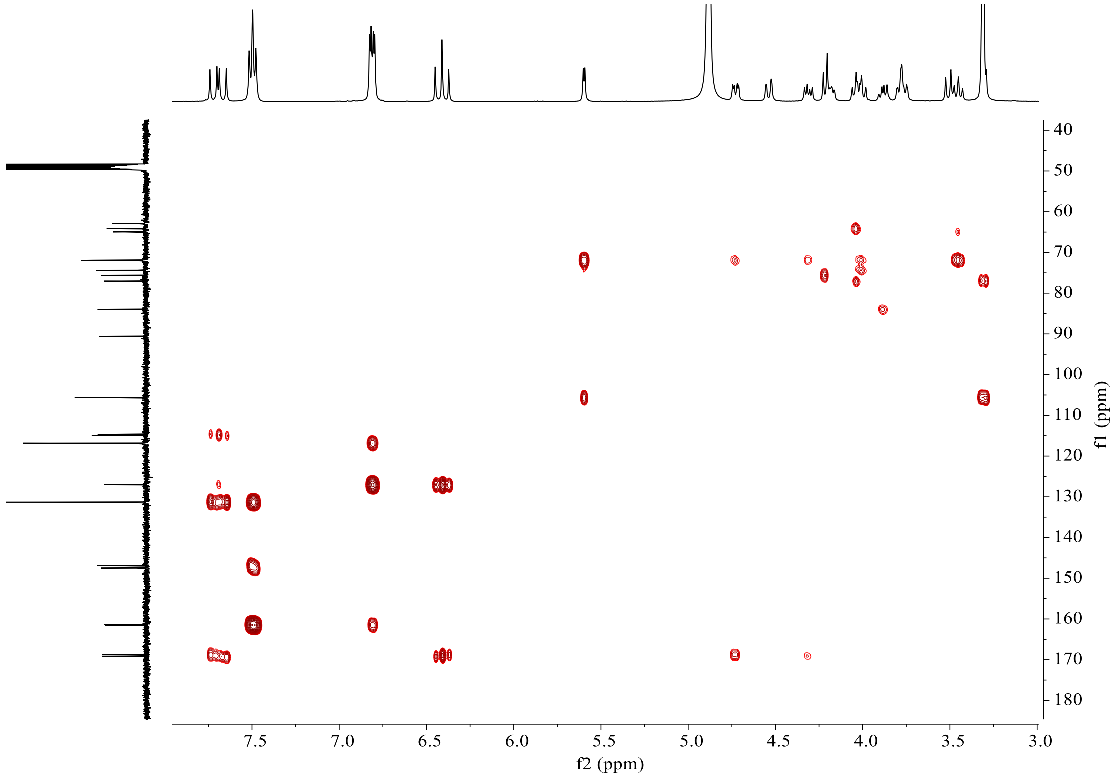

Figure S8. HMBC Spectrum of Compound **1** in MeOH-d_4

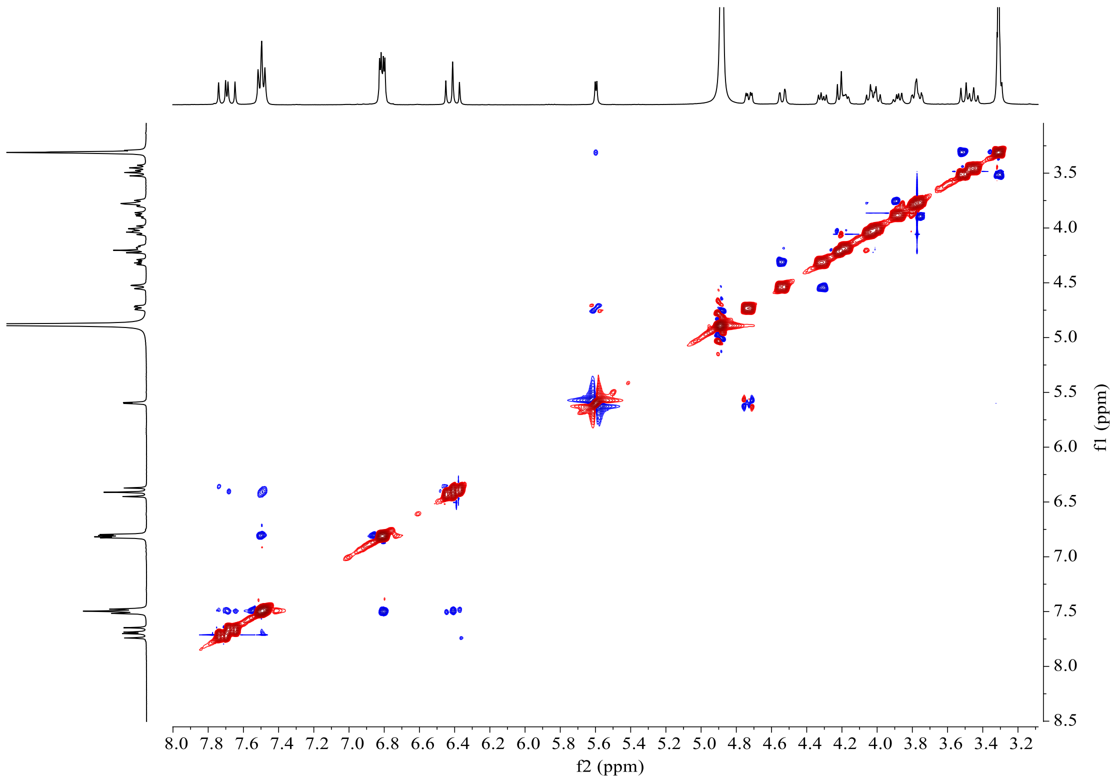

Figure S9. NOESY Spectrum of Compound **1** in MeOH-d_4

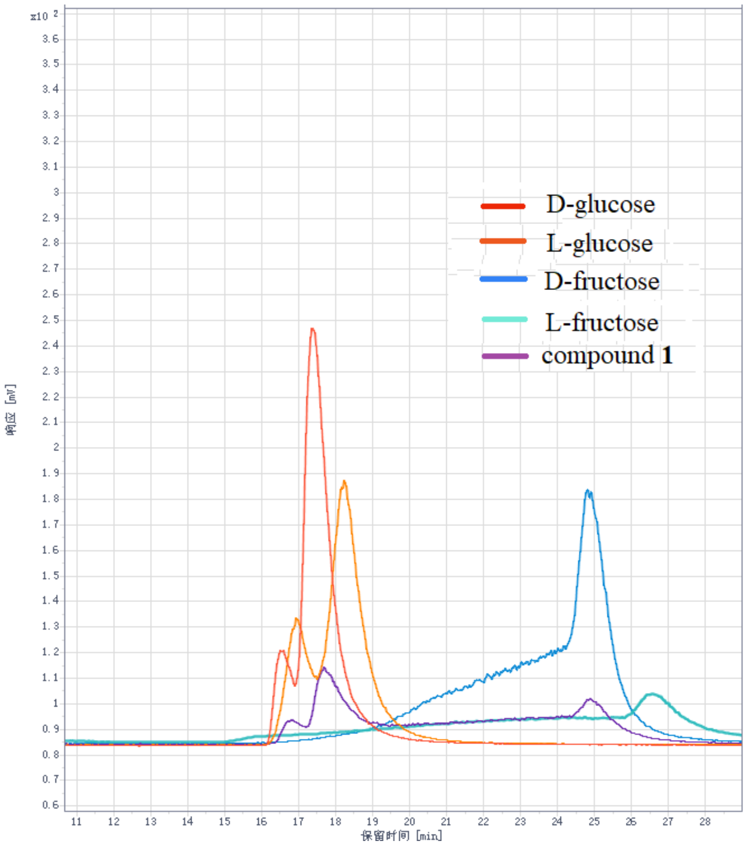

Figure S10. Chiral-HPLC profile from acid hydrolysis of **1** compared to authentic standard

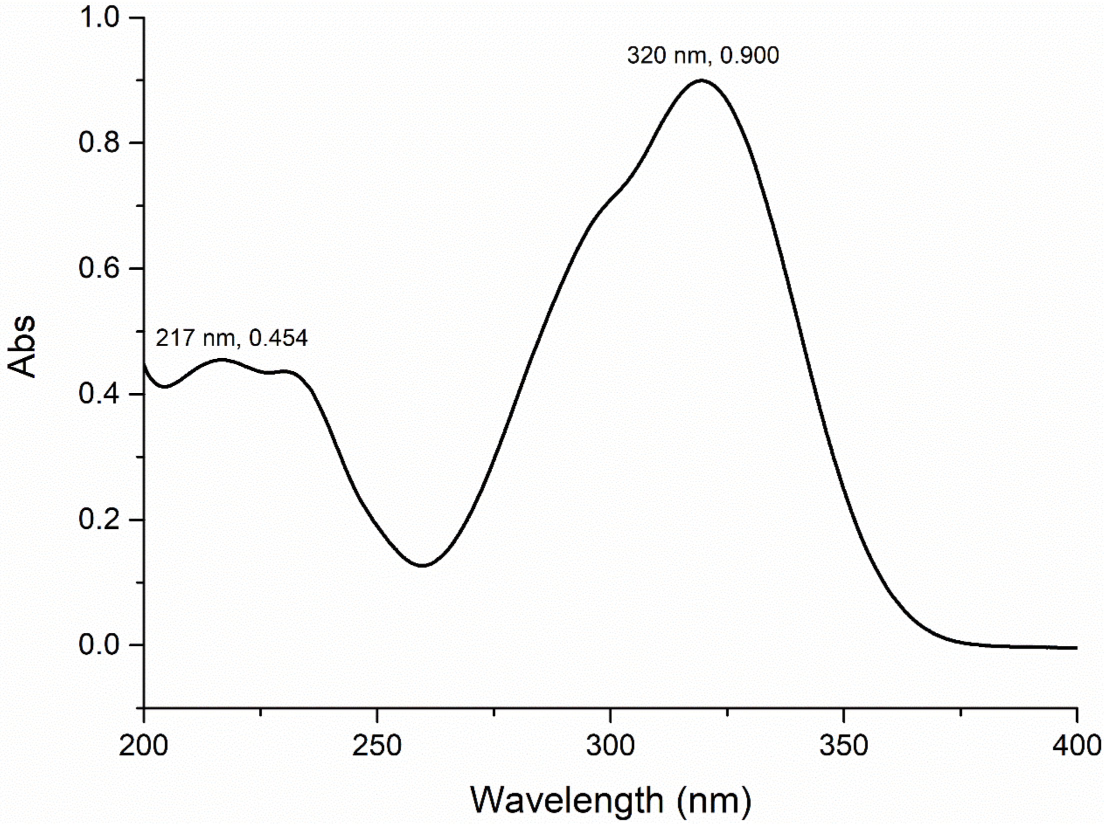

Figure S11. UV Spectrum of Compound **2**

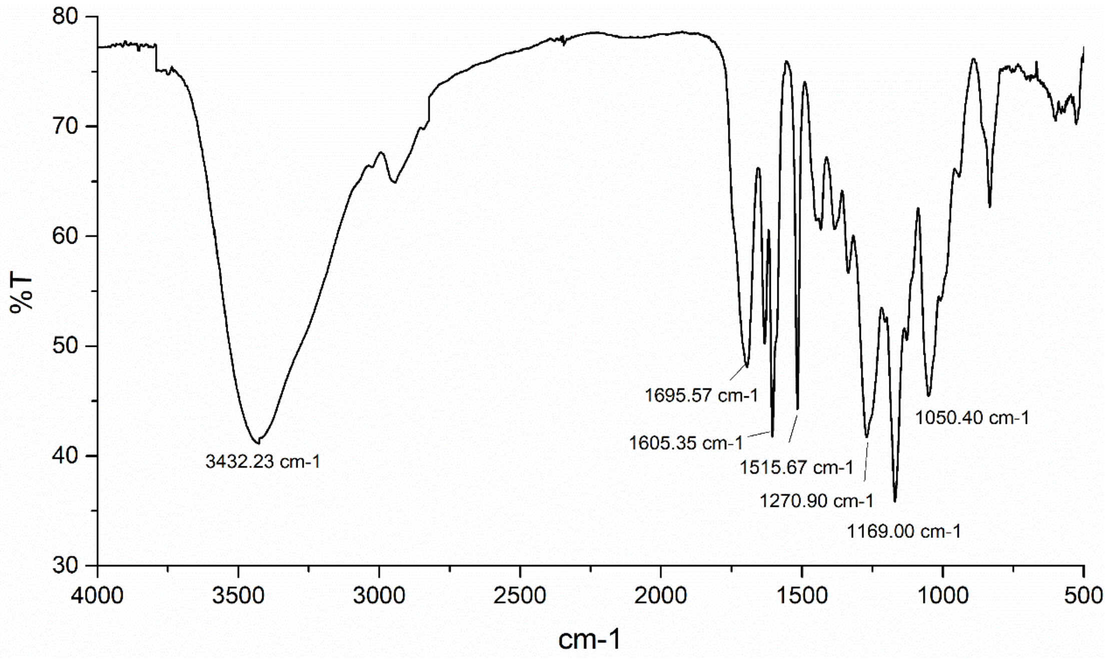

Figure S12. IR (KBr disc) Spectrum of Compound **2**

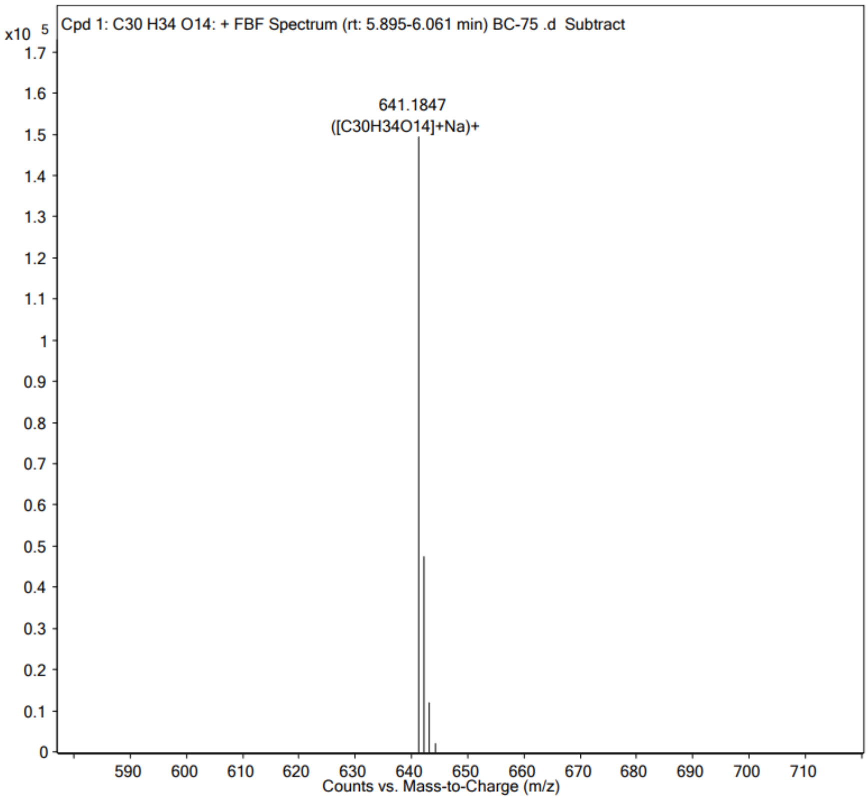

Figure S13. (+) HRESIMS Spectrum of Compound **2**

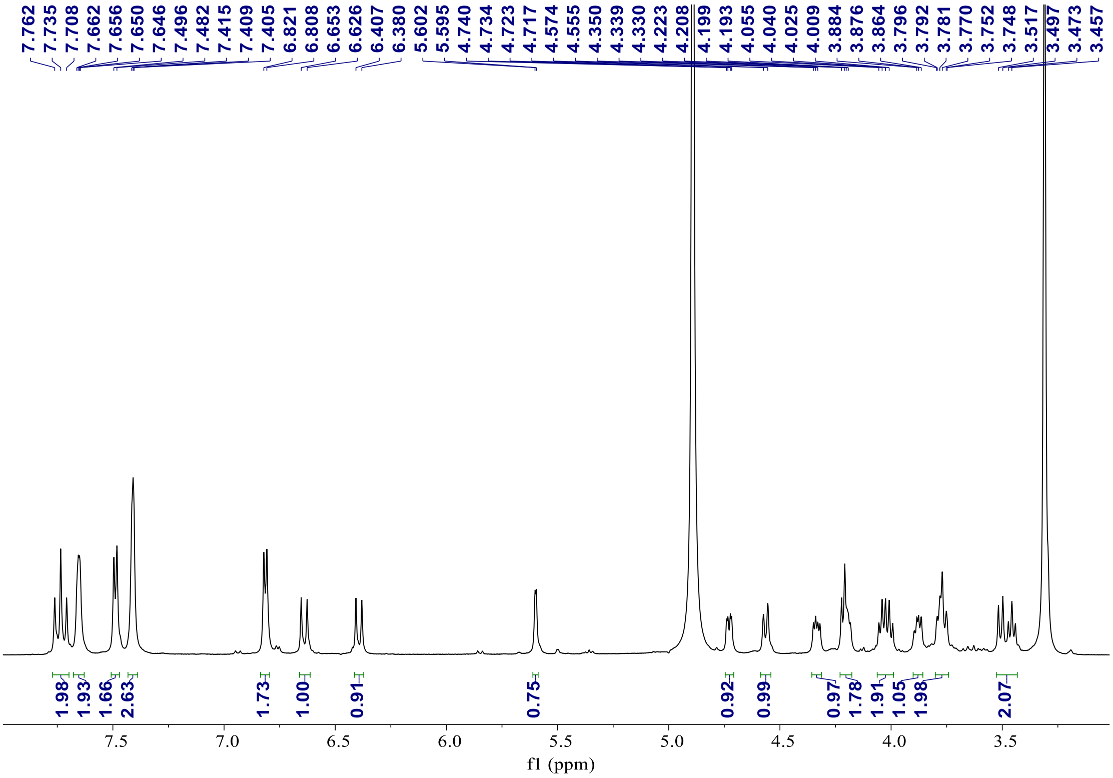

Figure S14. ^{1}H NMR Spectrum of Compound **2** in MeOH-d_4

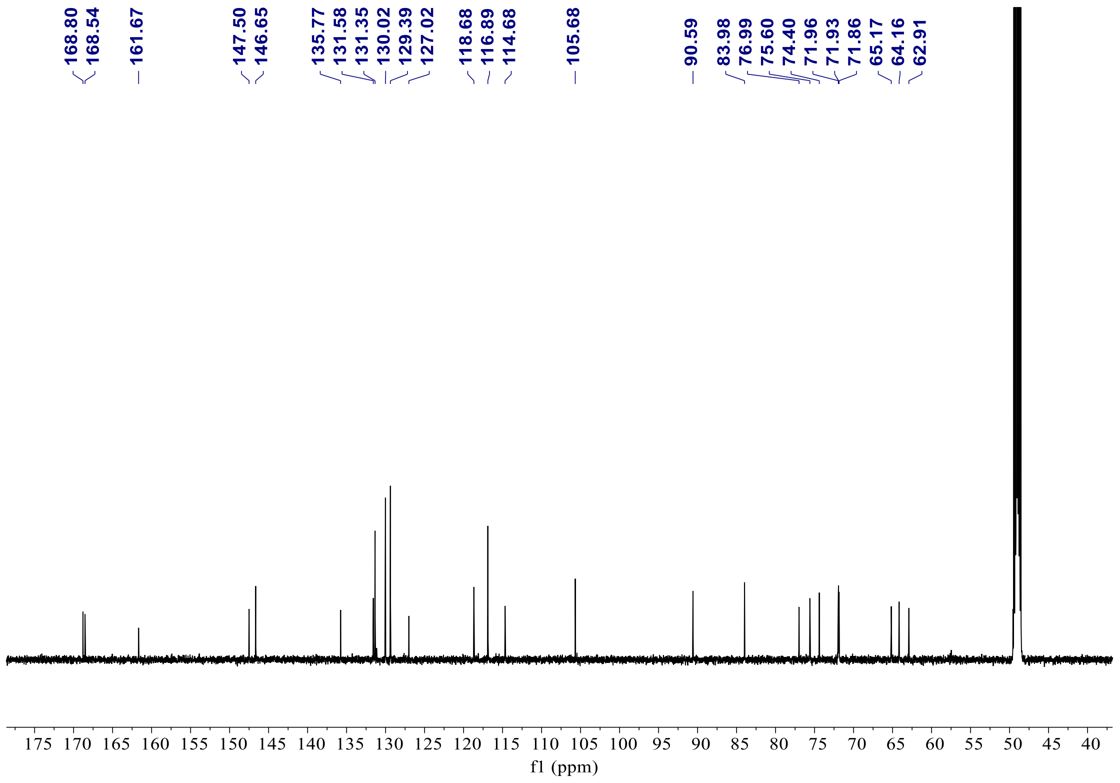

Figure S15. ^{13}C NMR Spectrum of Compound **2** in MeOH-d_4

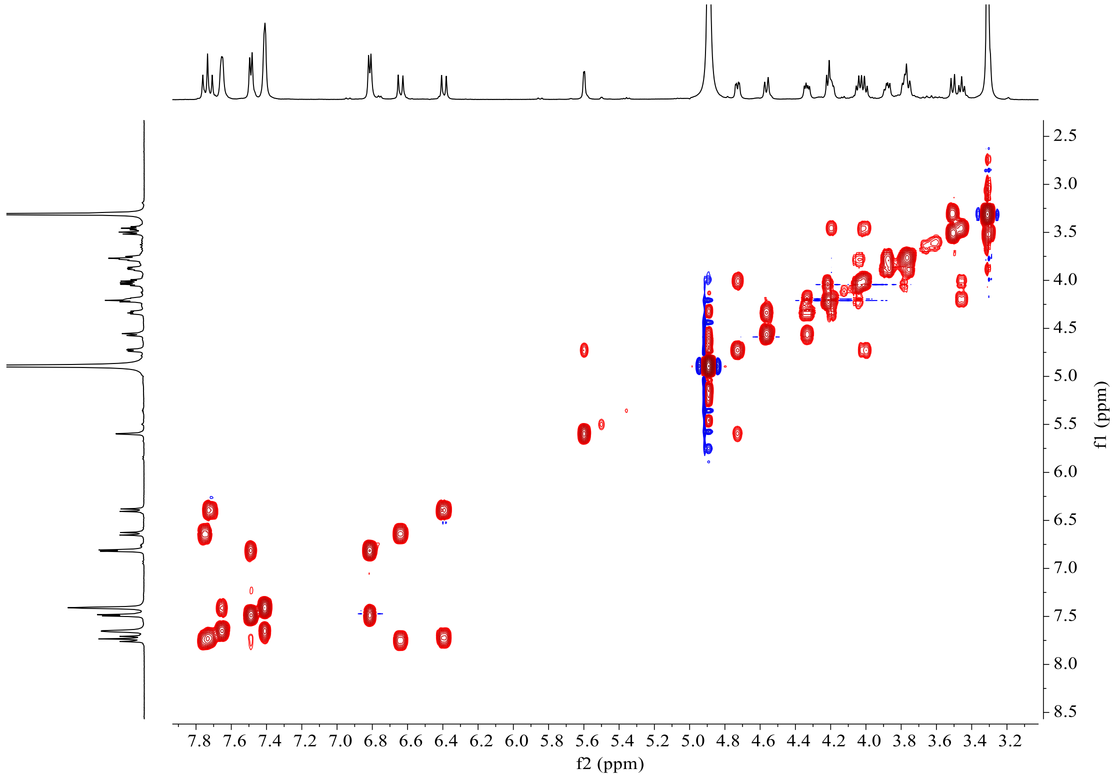

Figure S16. ^{1}H–^{1}H COSY Spectrum of Compound **2** in MeOH-d_4

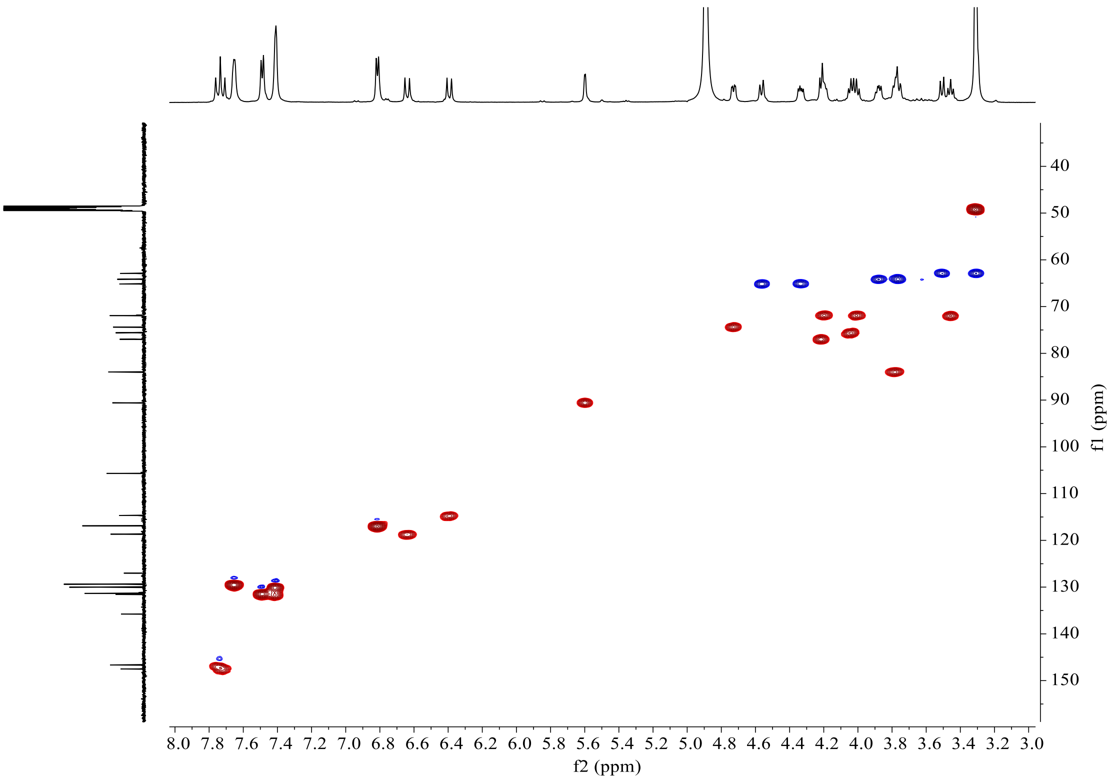

Figure S17. HSQC Spectrum of Compound **2** in MeOH-d_4

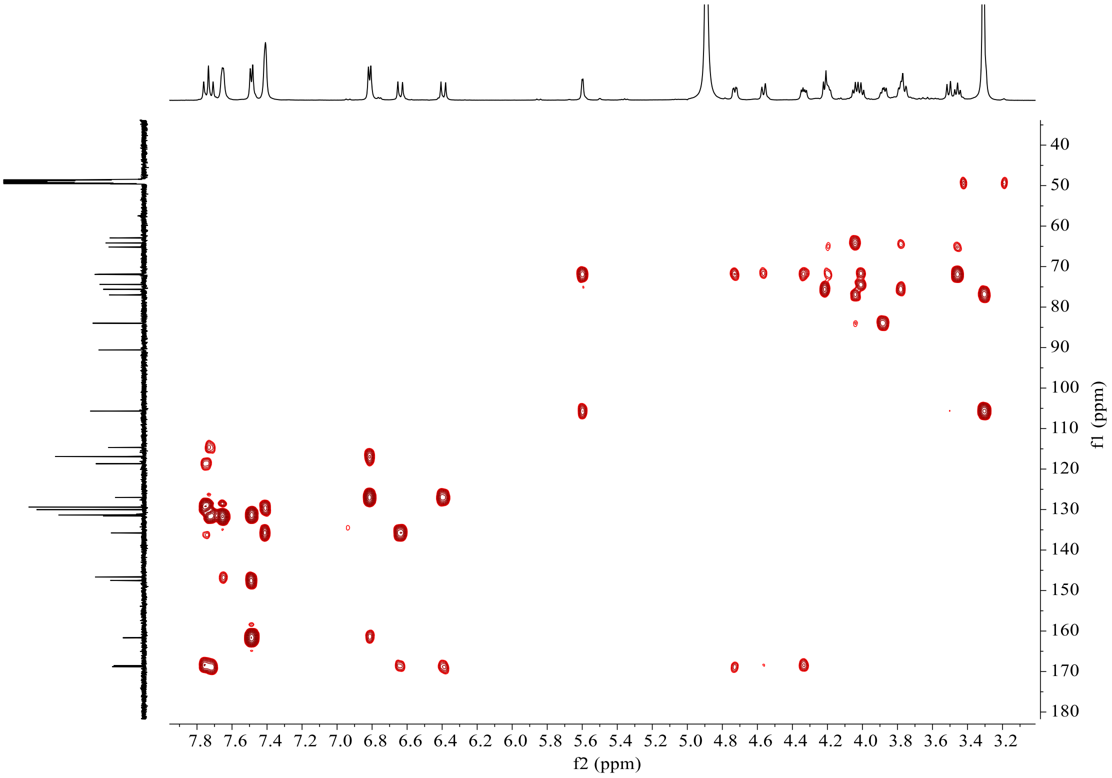

Figure S18. HMBC Spectrum of Compound **2** in MeOH-d_4

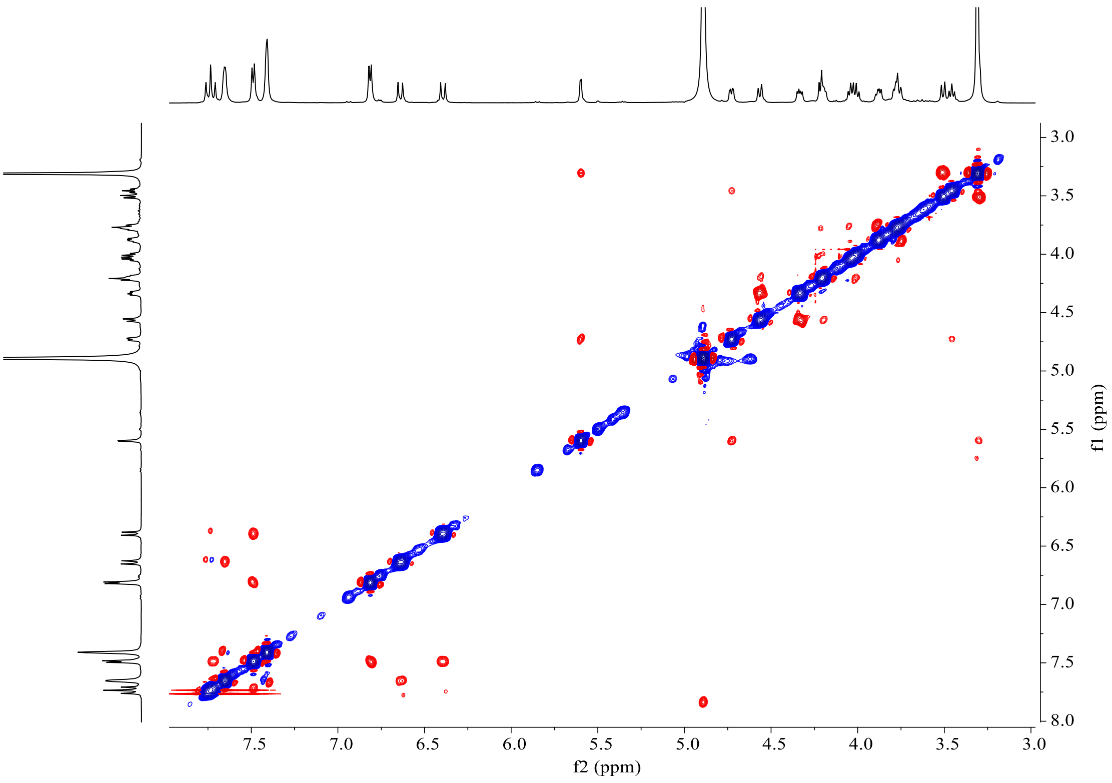

Figure S19. NOESY Spectrum of Compound **2** in MeOH-d_4

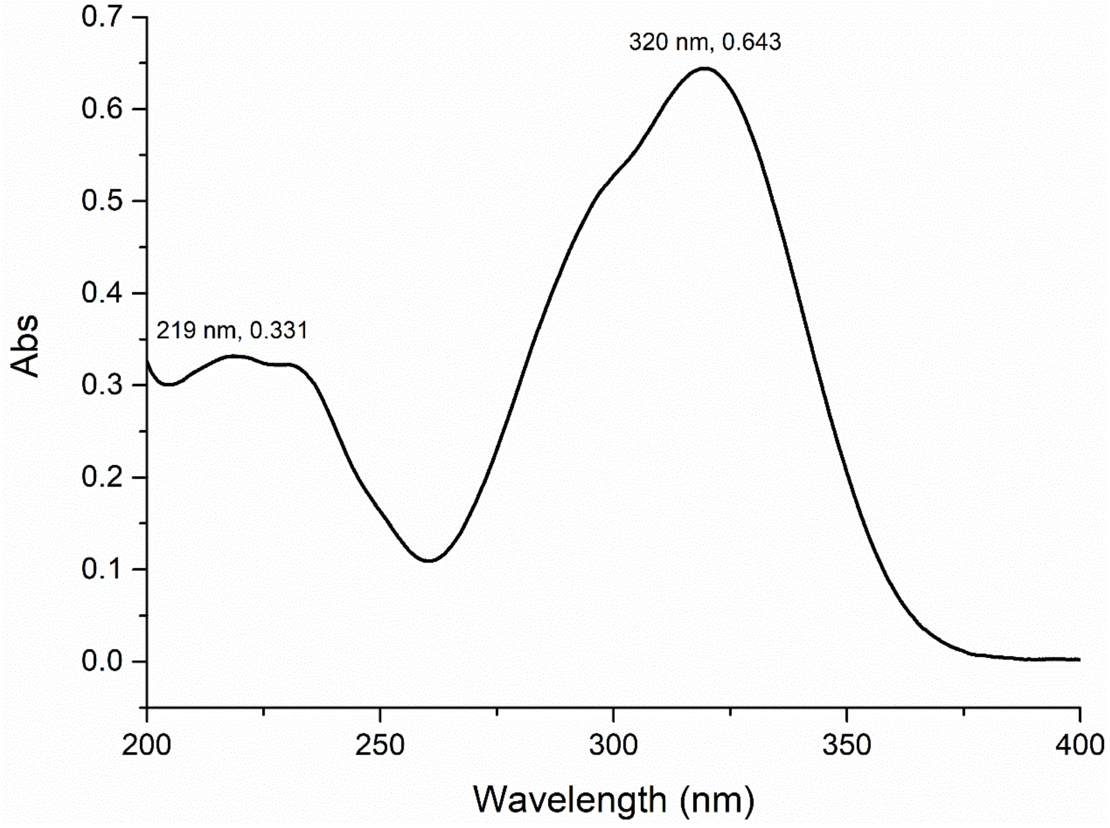

Figure S20. UV Spectrum of Compound **3**

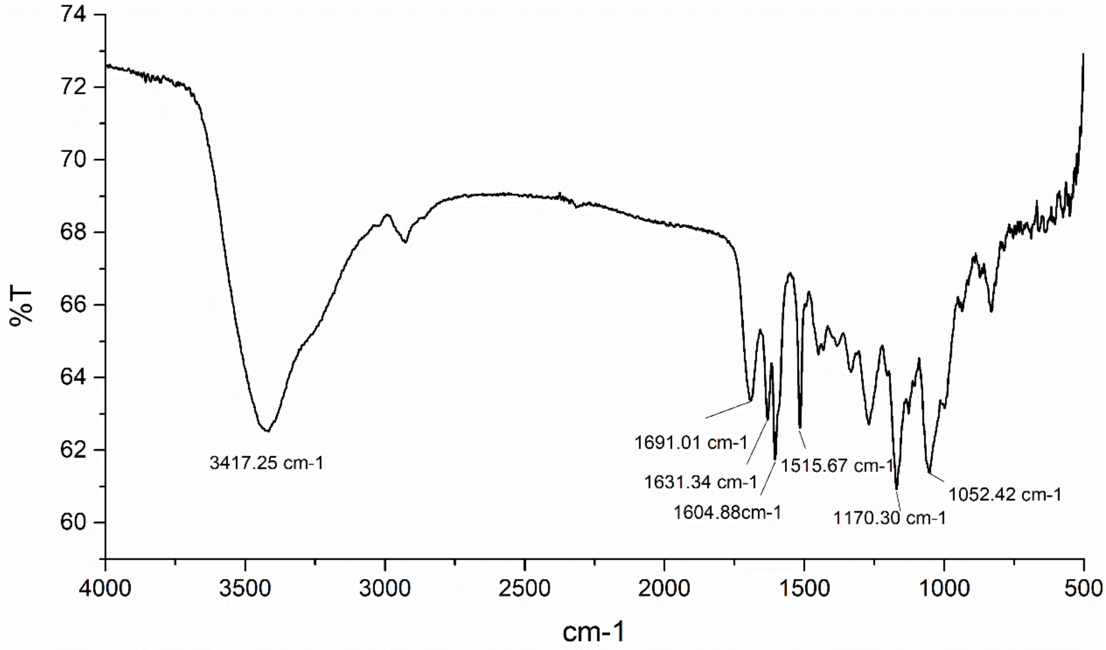

Figure S21. IR (KBr disc) Spectrum of Compound **3**

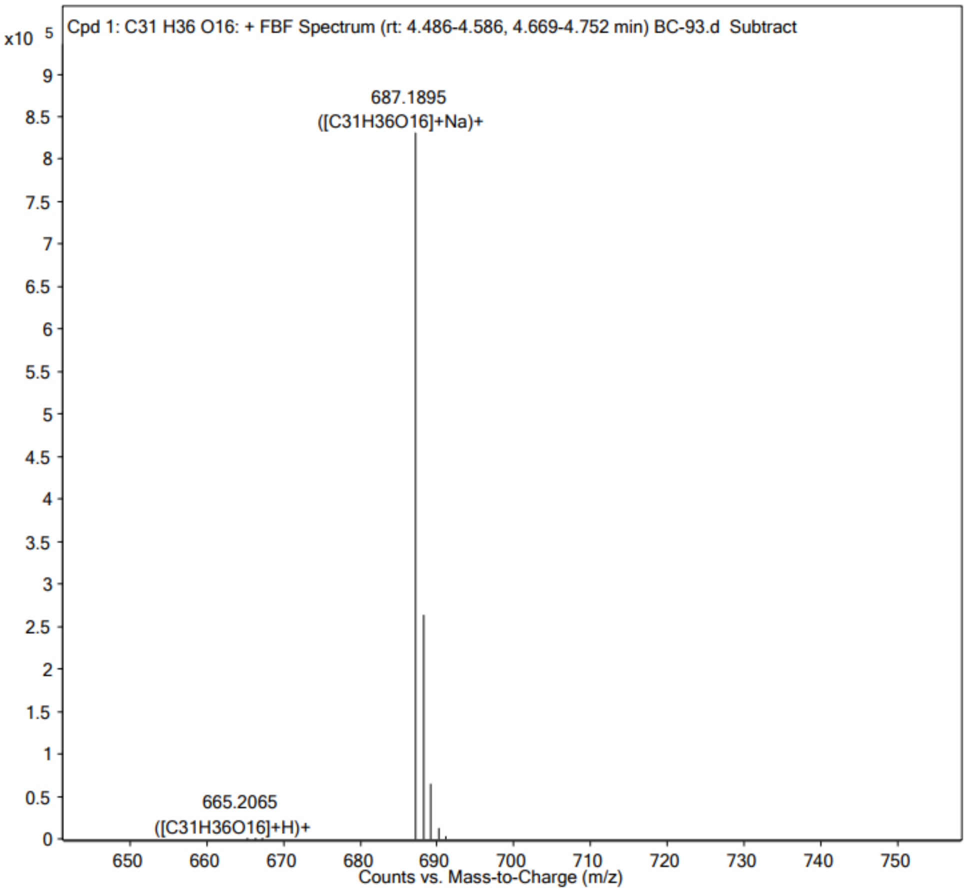

Figure S22. (+) HRESIMS Spectrum of Compound **3**

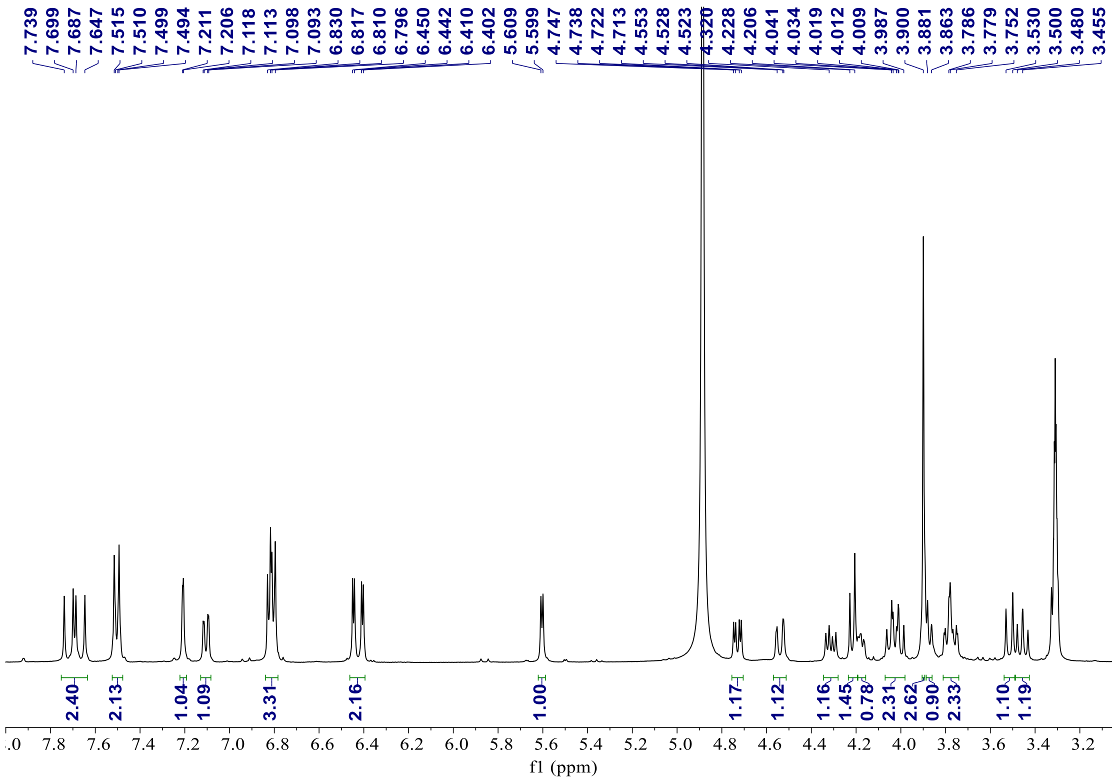

Figure S23. ^{1}H NMR Spectrum of Compound **3** in MeOH-d_4

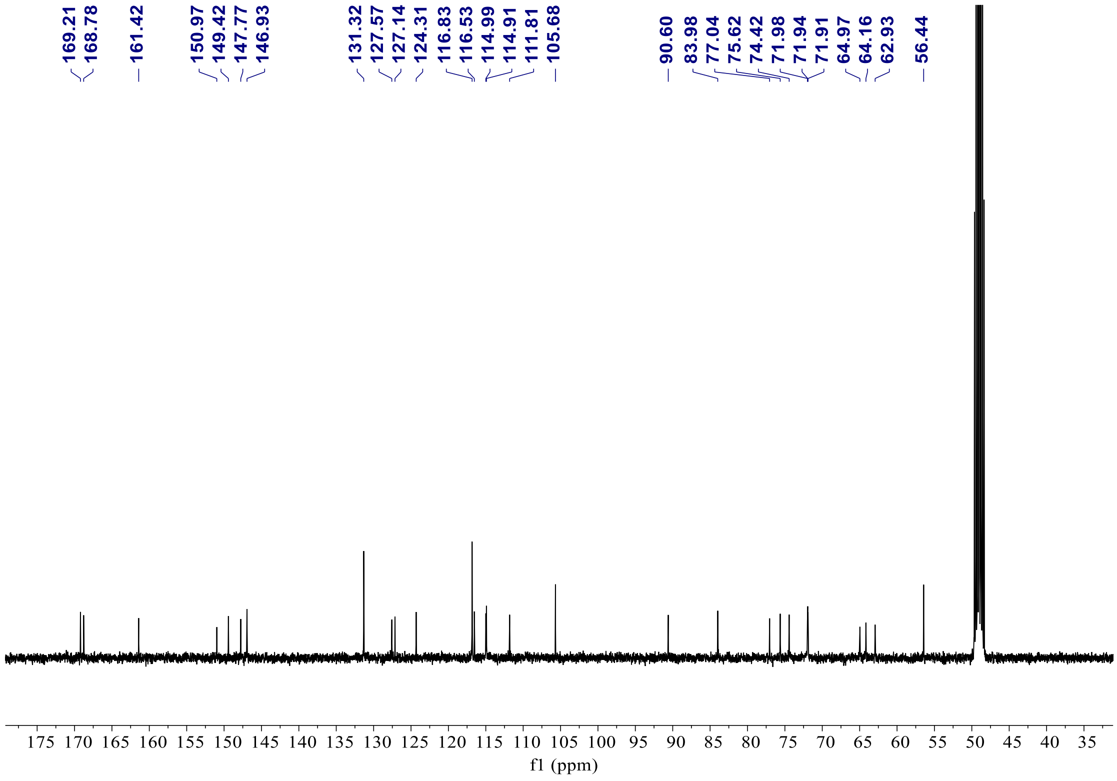

Figure S24. ^{13}C NMR Spectrum of Compound **3** in MeOH-d_4

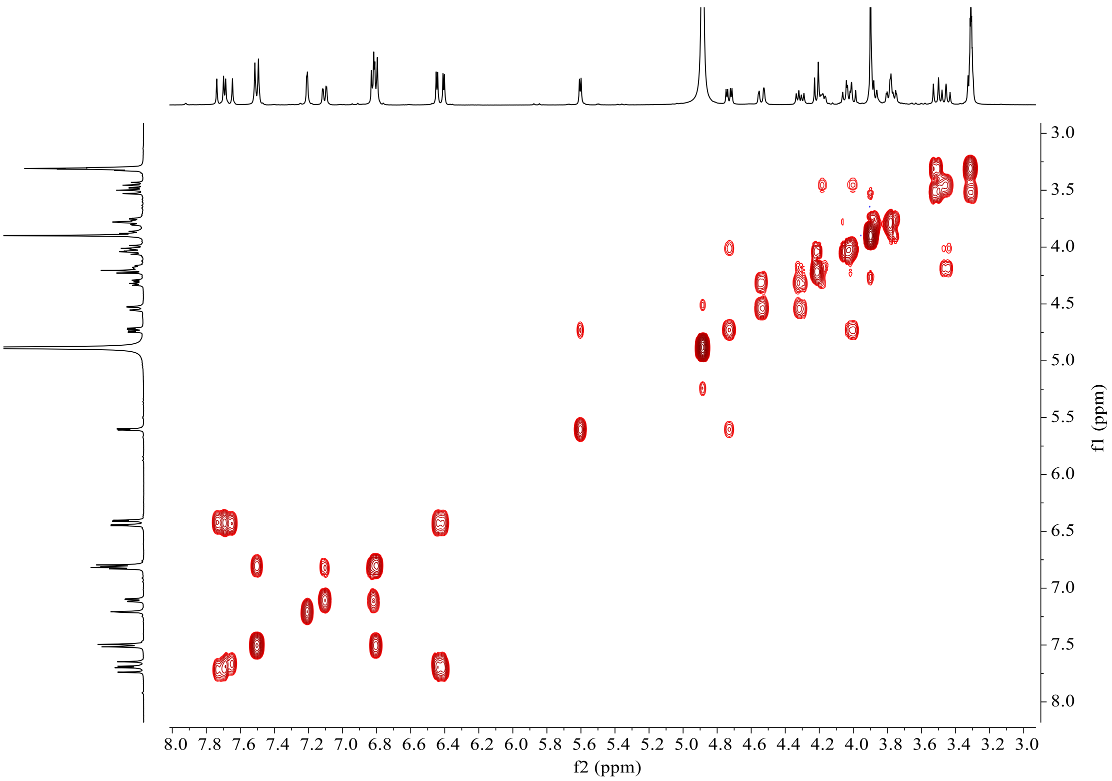

Figure S25. ^{1}H–^{1}H COSY Spectrum of Compound **3** in MeOH-d_4

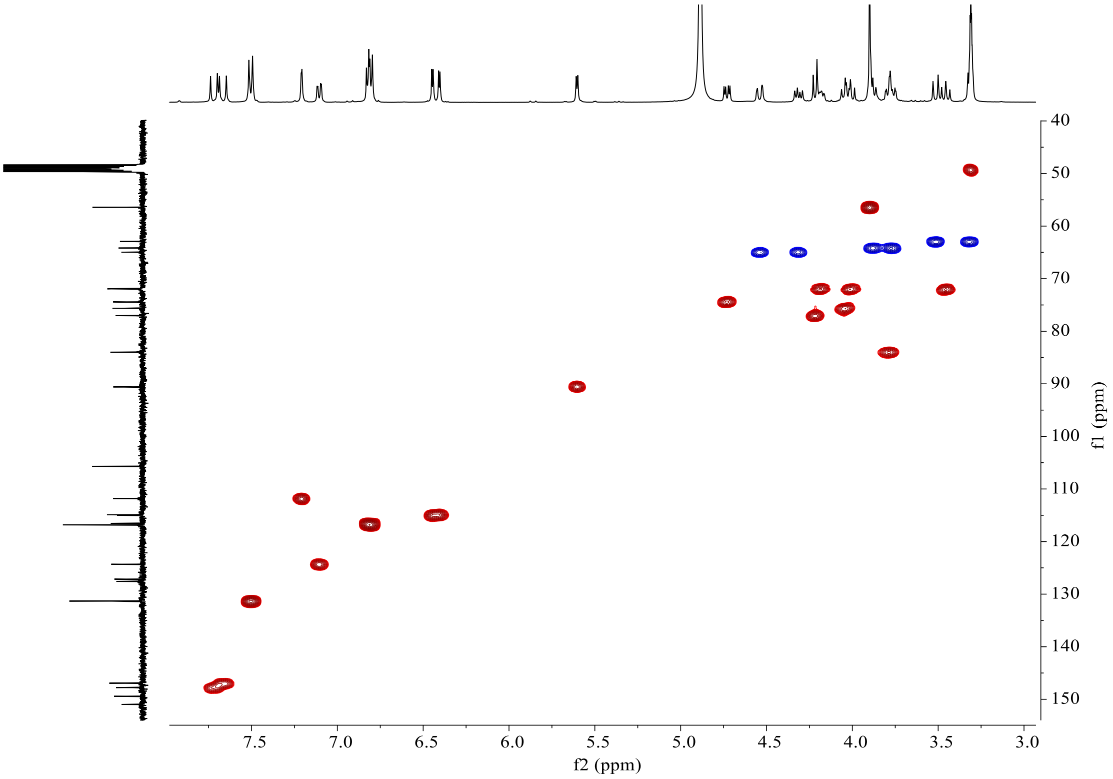

Figure S26. HSQC Spectrum of Compound **3** in MeOH-d_4

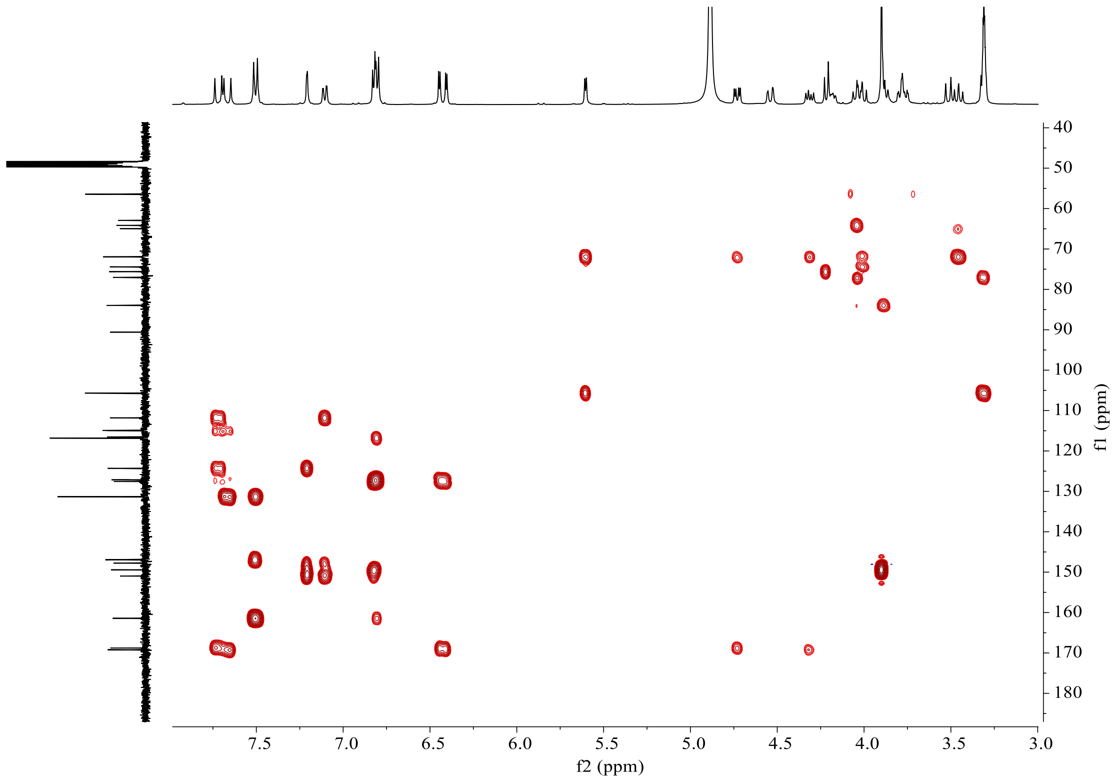

Figure S27. HMBC Spectrum of Compound **3** in MeOH-d_4

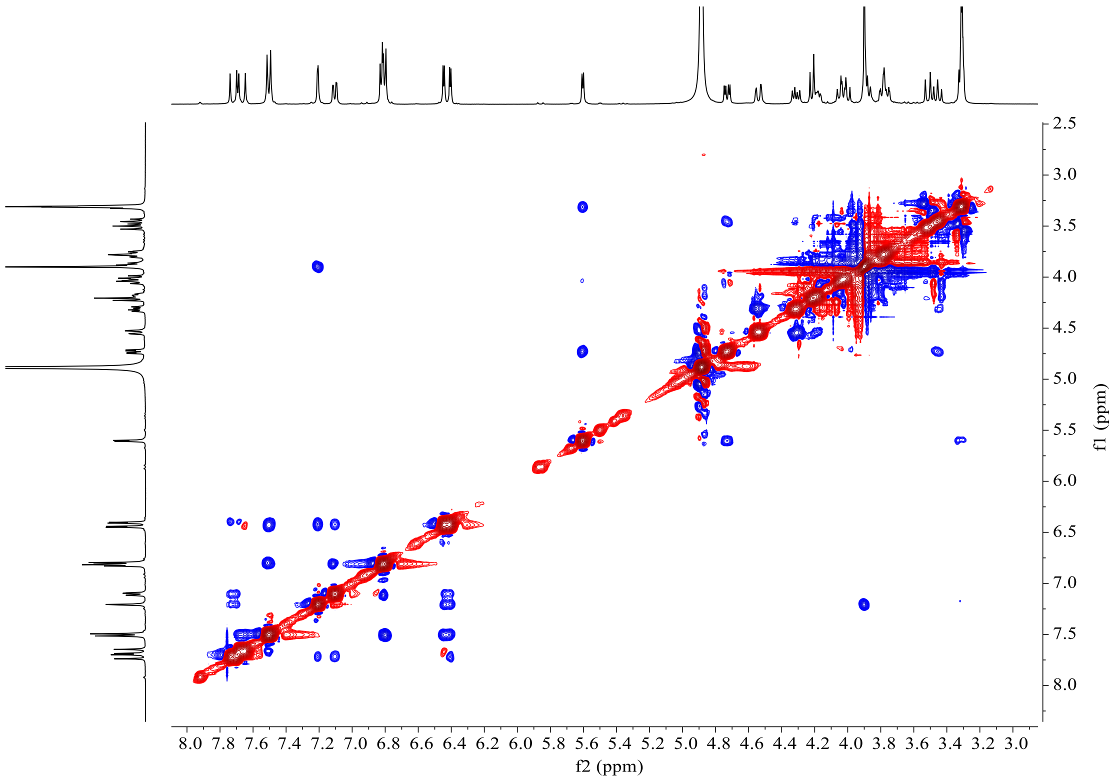

Figure S28. NOESY Spectrum of Compound **3** in MeOH-d_4

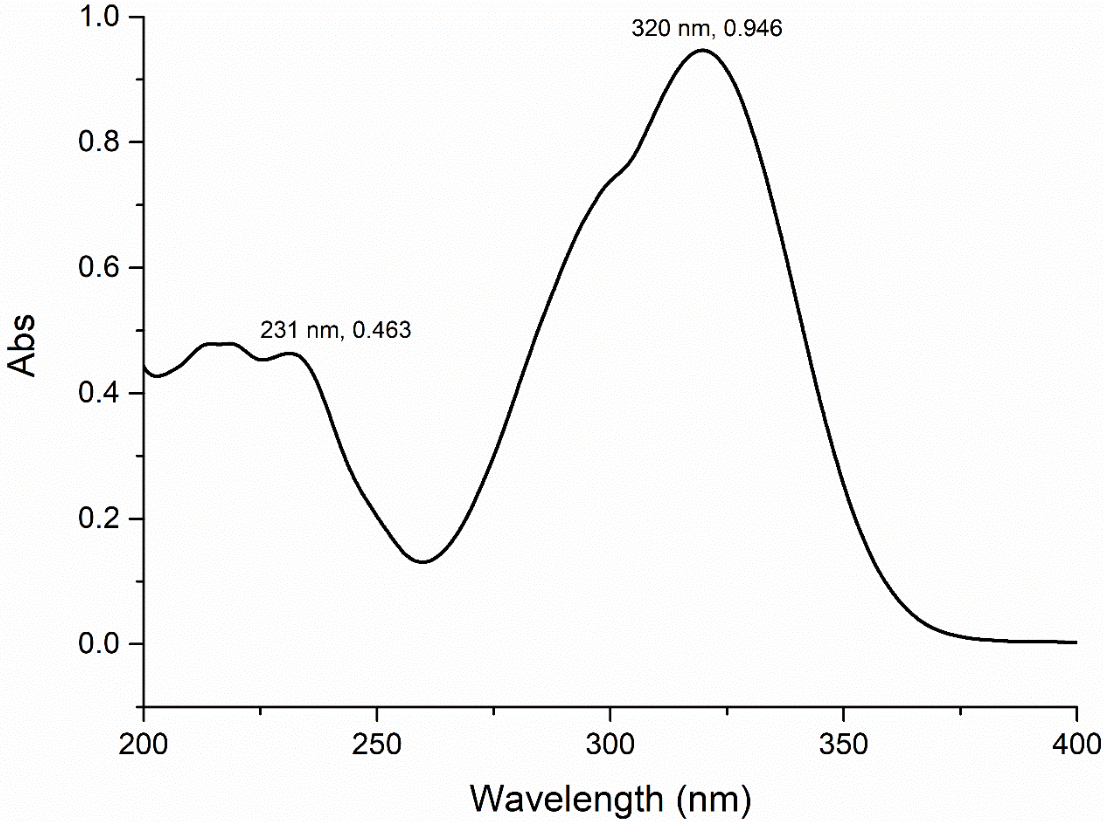

Figure S29. UV Spectrum of Compound **4**

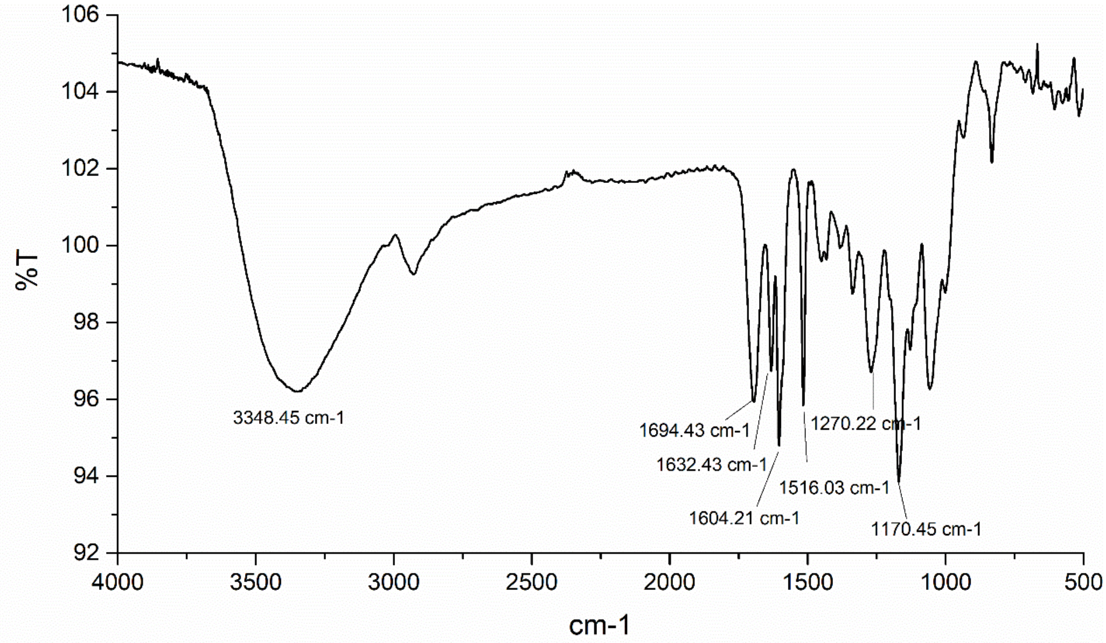

Figure S30. IR (KBr disc) Spectrum of Compound **4**

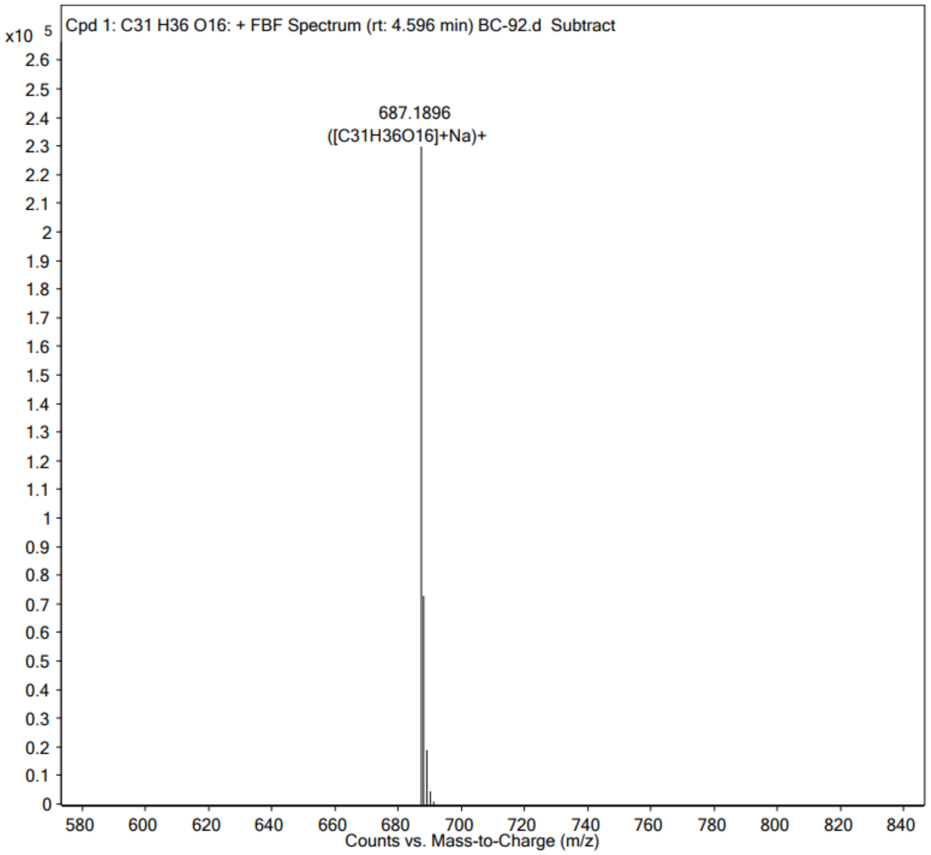

Figure S31. (+) HRESIMS Spectrum of Compound **4**

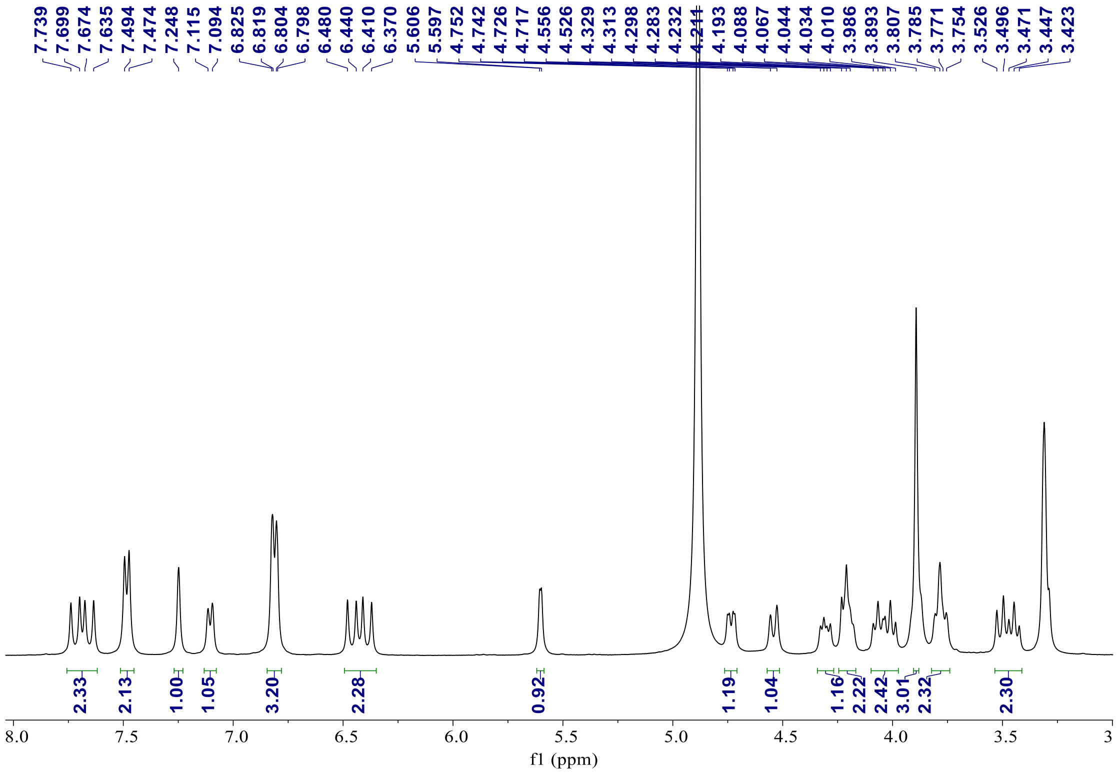

Figure S32. ^{1}H NMR Spectrum of Compound **4** in MeOH-d_4

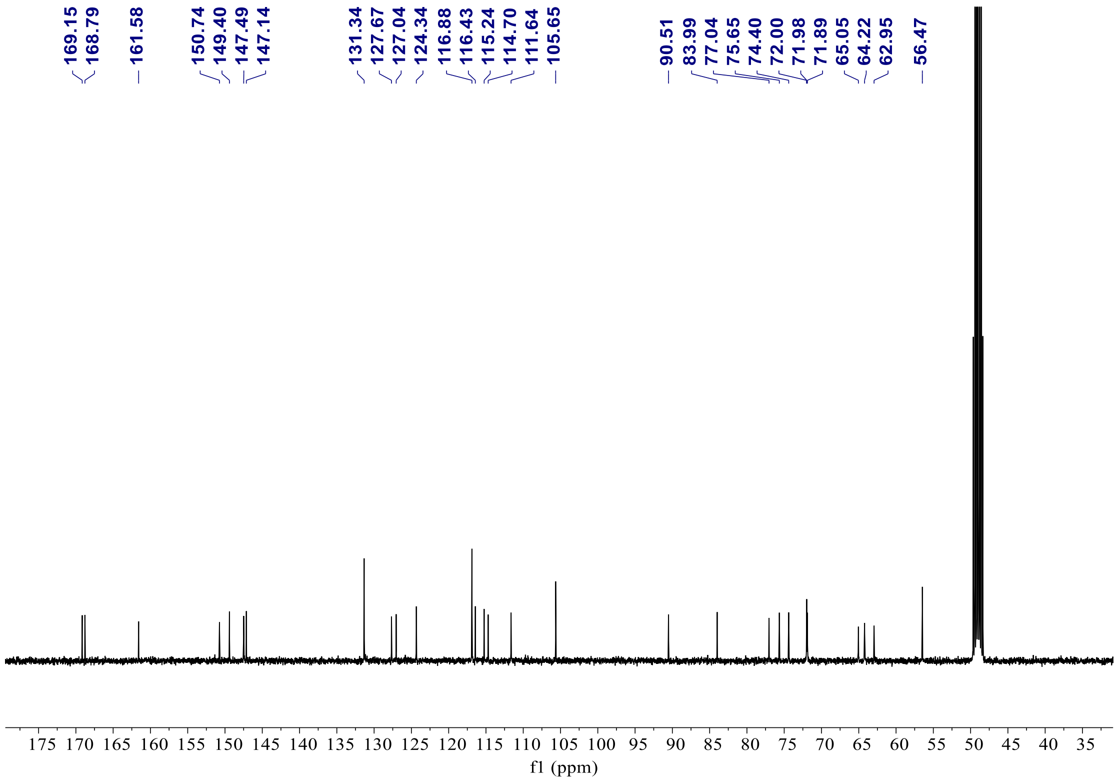

Figure S33. ^{13}C NMR Spectrum of Compound **4** in MeOH-d_4

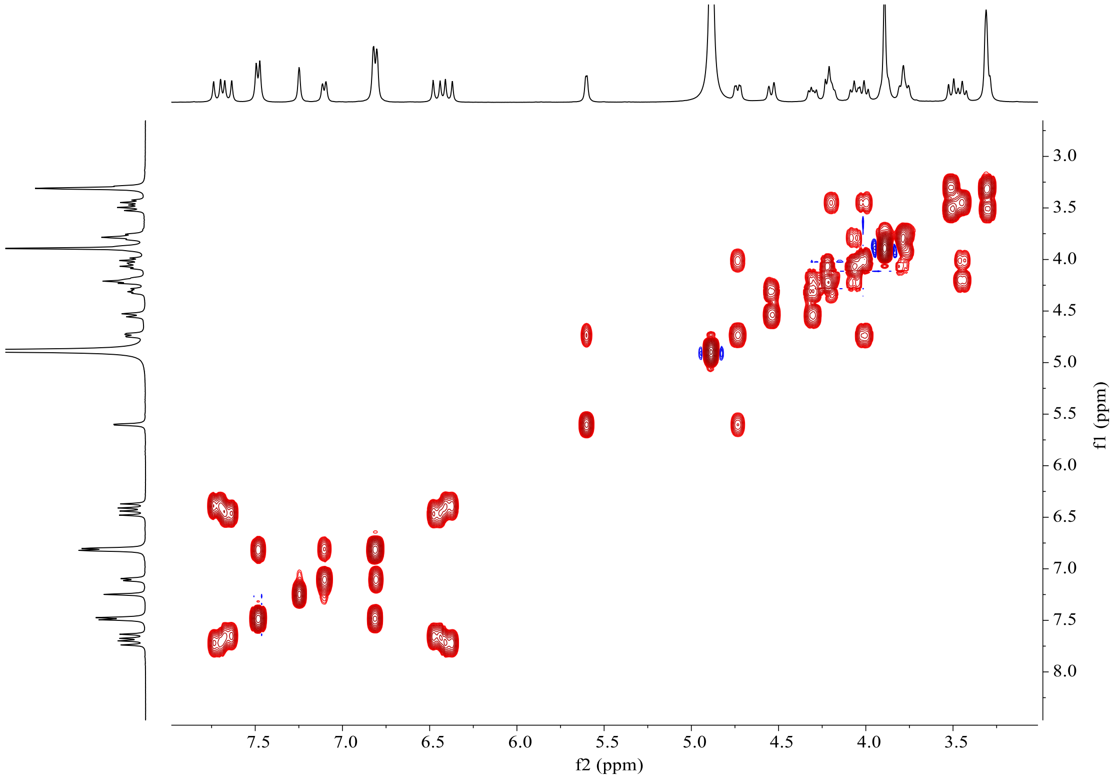

Figure S34. ^{1}H–^{1}H COSY Spectrum of Compound **4** in MeOH-d_4

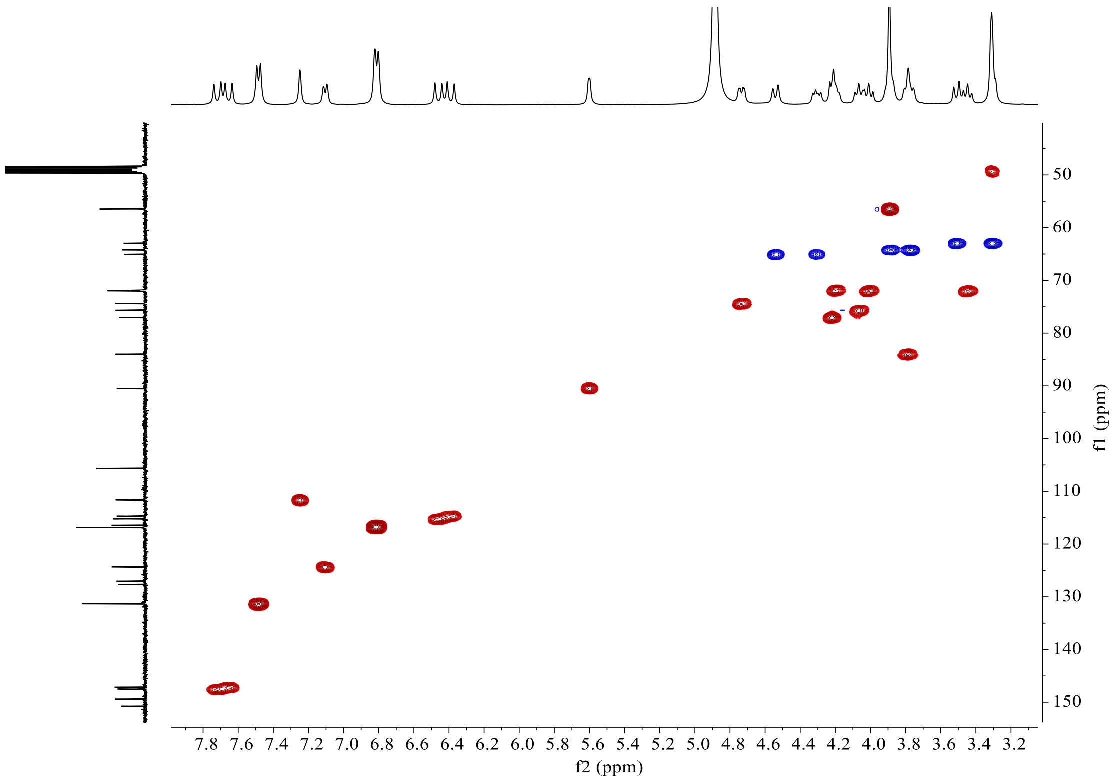

Figure S35. HSQC Spectrum of Compound **4** in MeOH-d_4

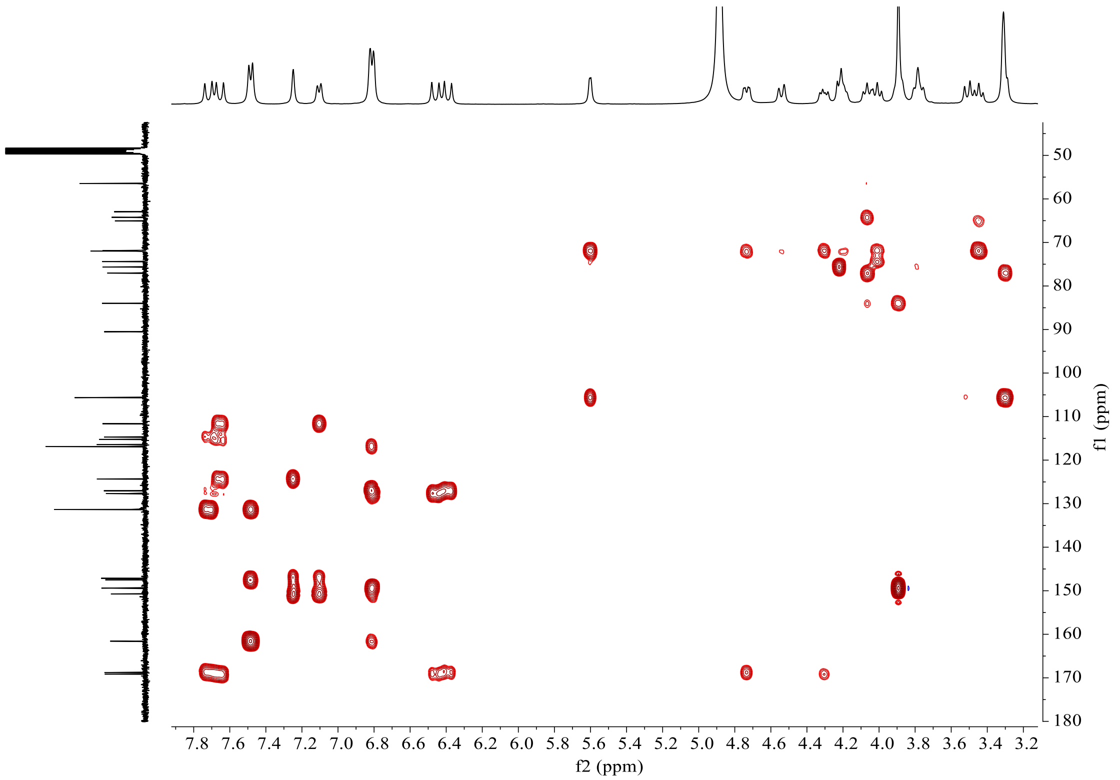

Figure S36. HMBC Spectrum of Compound **4** in MeOH-d_4

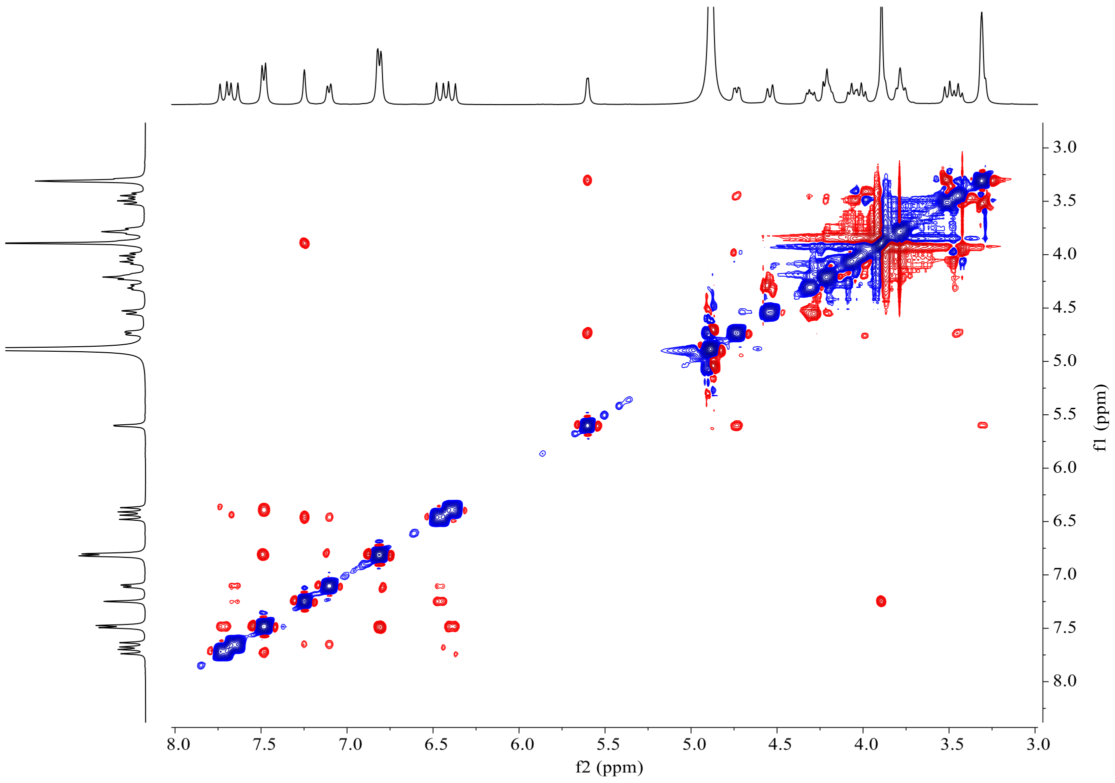

Figure S37. NOESY Spectrum of Compound **4** in MeOH-d_4

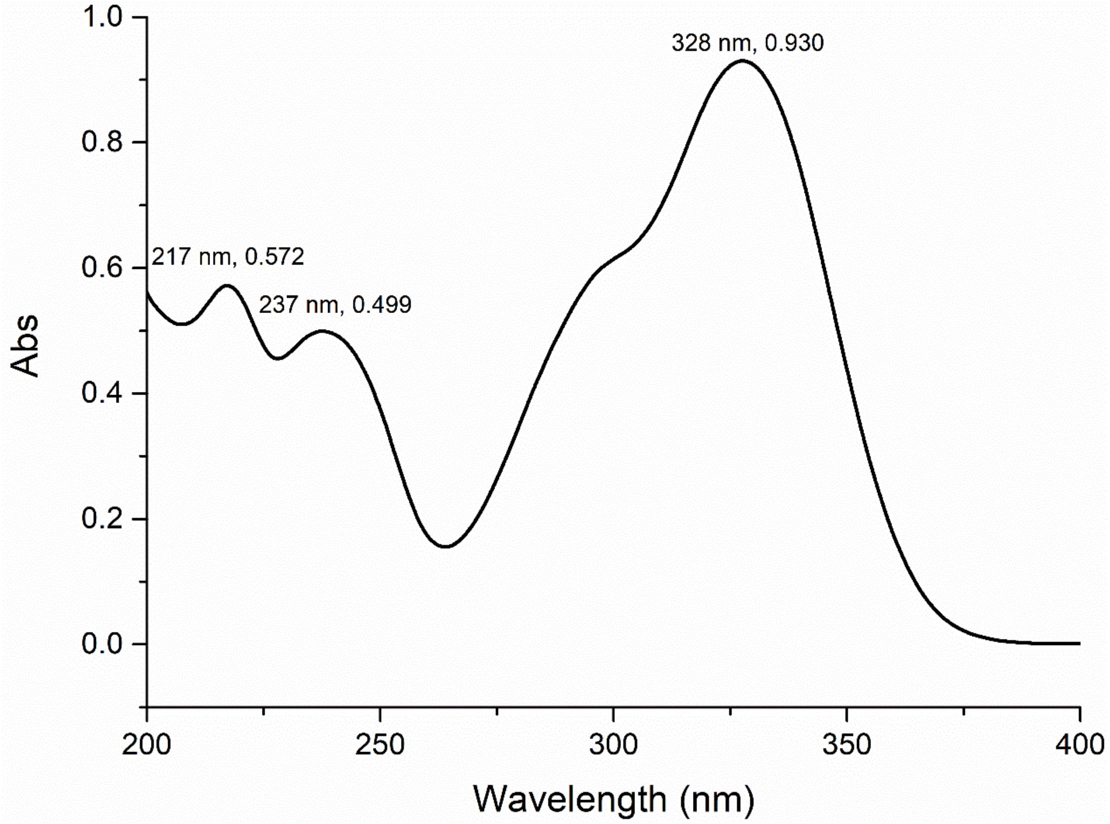

Figure S38. UV Spectrum of Compound **5**

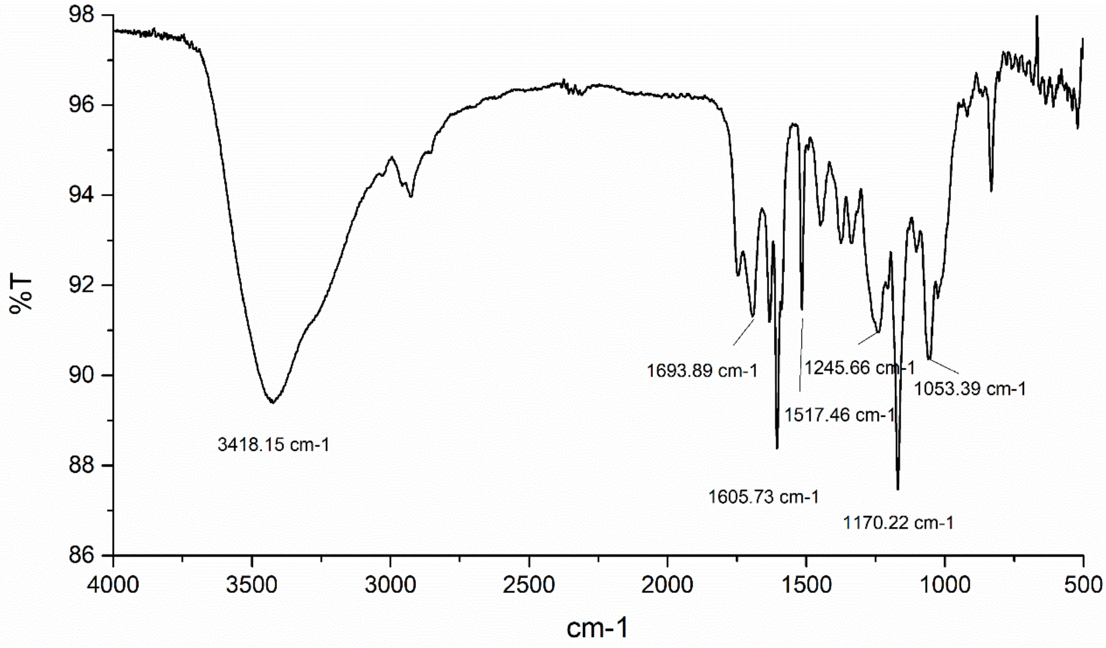

Figure S39. IR (KBr disc) Spectrum of Compound **5**

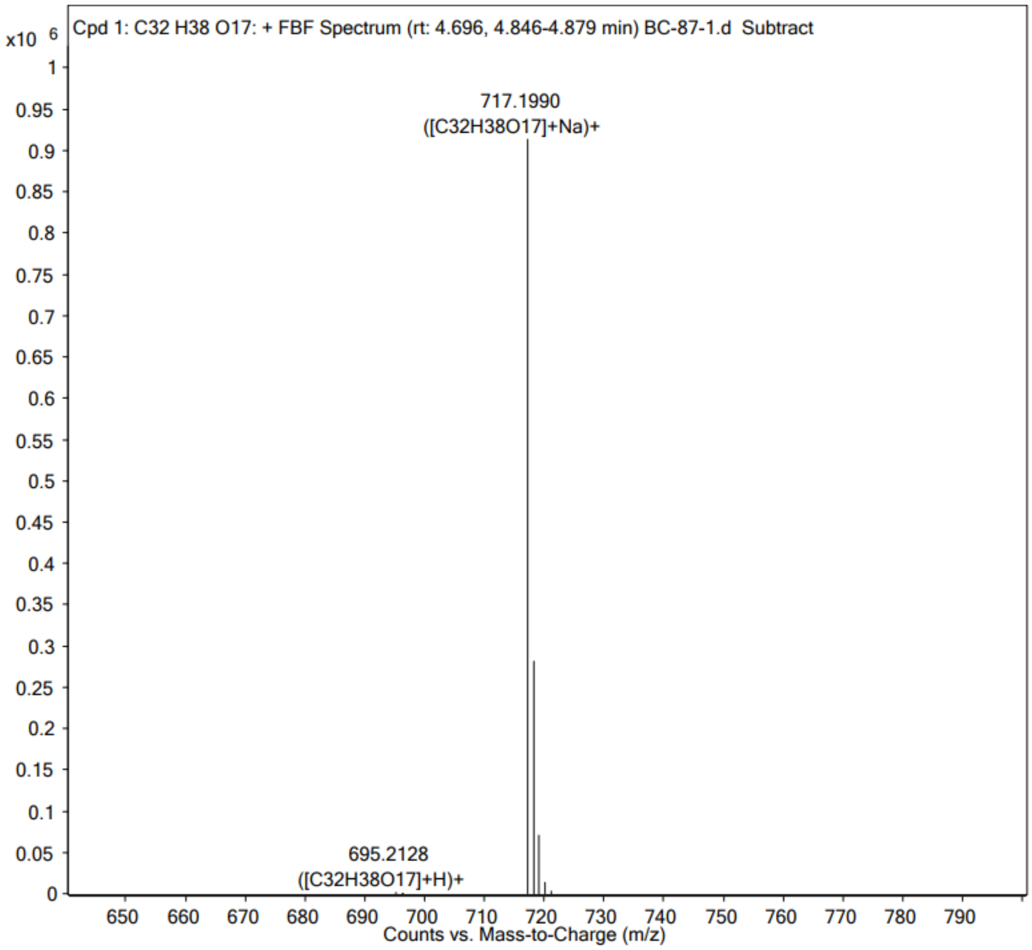

Figure S40. (+) HRESIMS Spectrum of Compound **5**

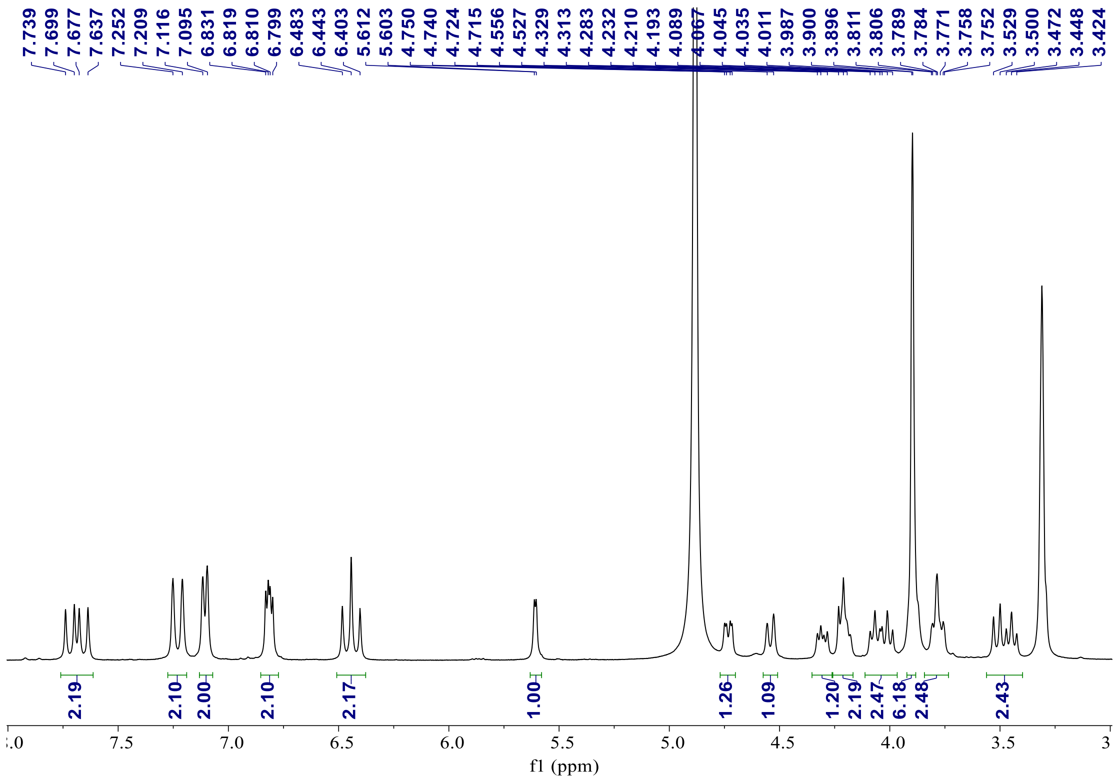

Figure S41. ^{1}H NMR Spectrum of Compound **5** in MeOH-d_4

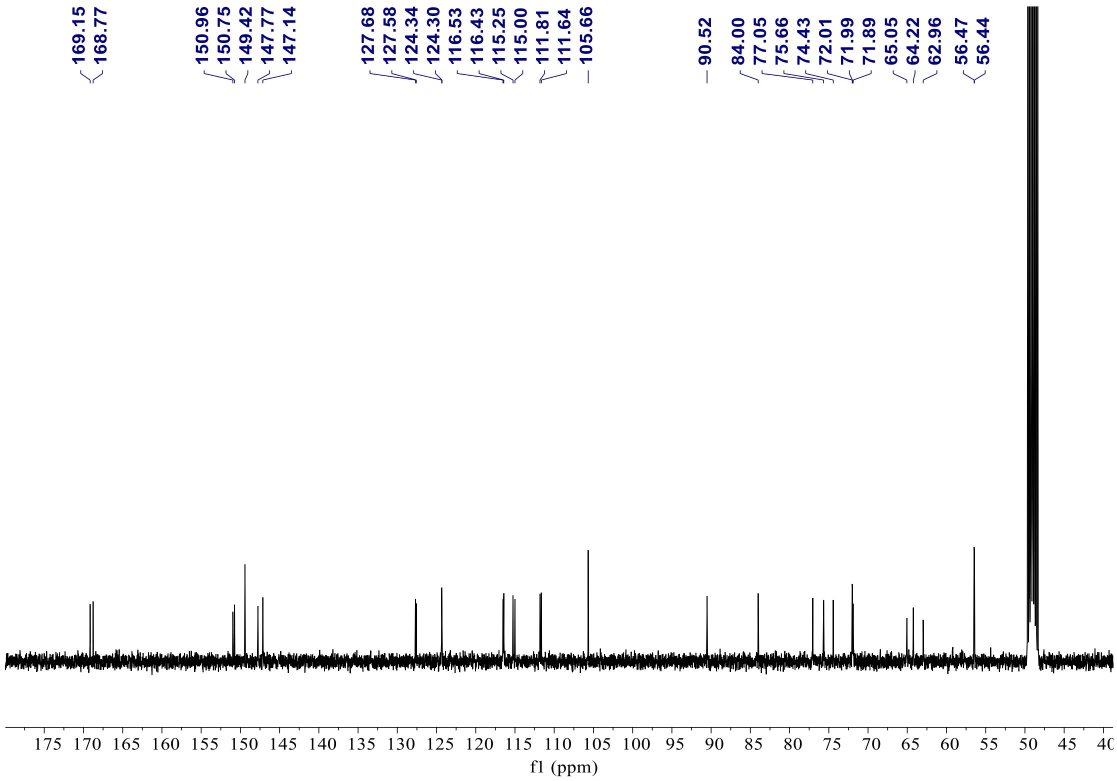

Figure S42. ^{13}C NMR Spectrum of Compound **5** in MeOH-d_4

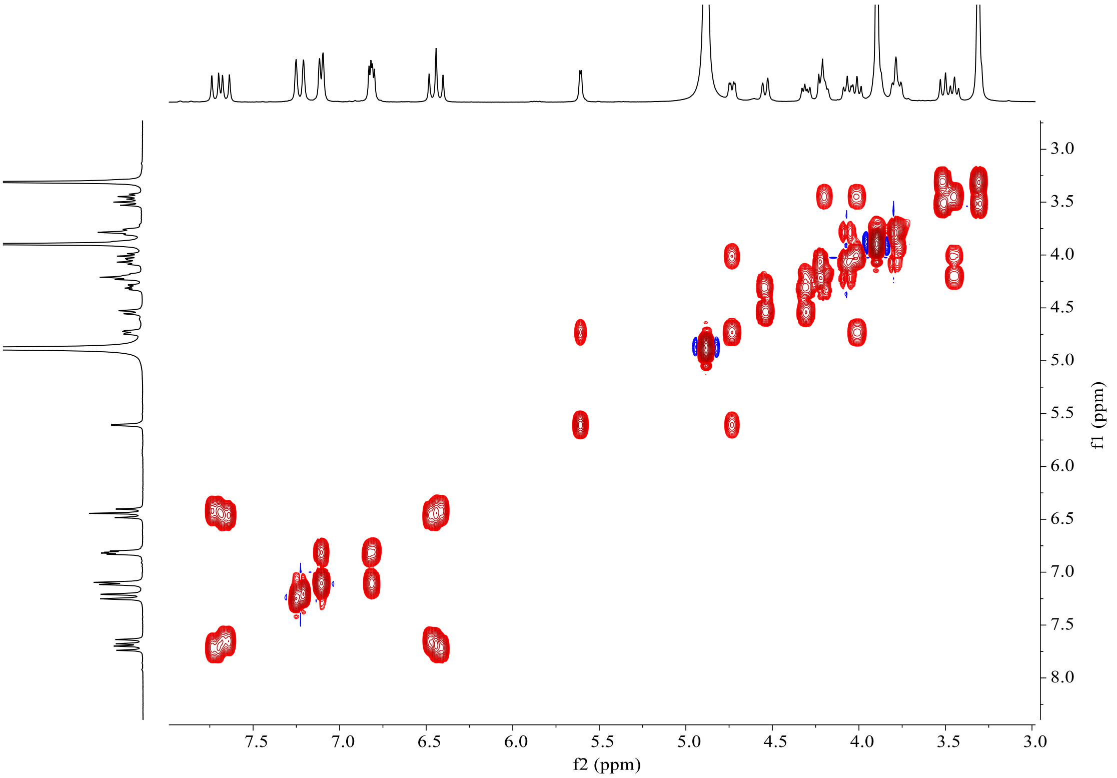

Figure S43. ^{1}H–^{1}H COSY Spectrum of Compound **5** in MeOH-d_4

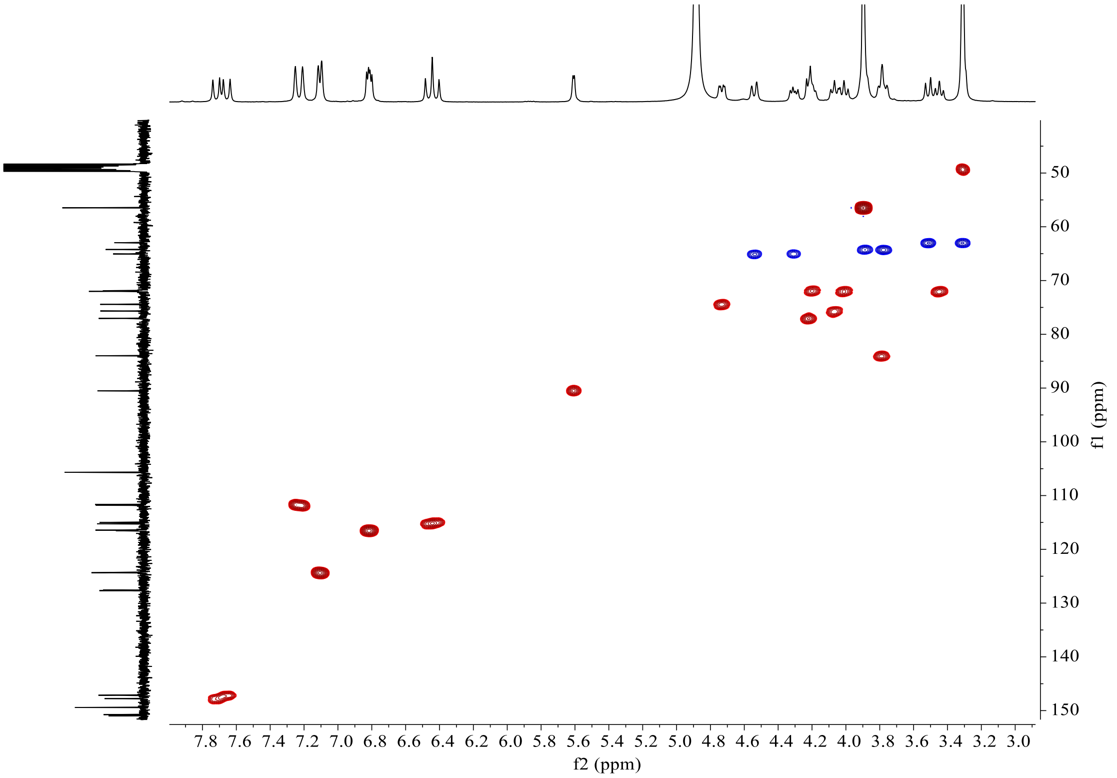

Figure S44. HSQC Spectrum of Compound **5** in MeOH-d_4

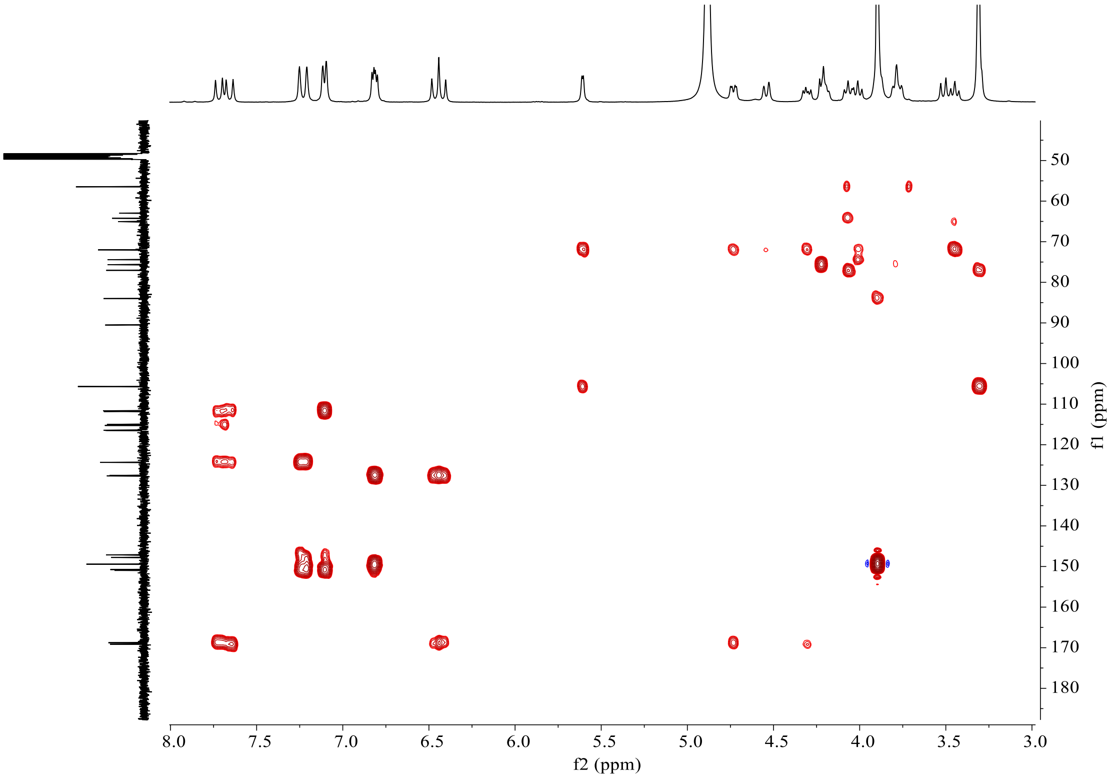

Figure S45. HMBC Spectrum of Compound **5** in MeOH-d_4

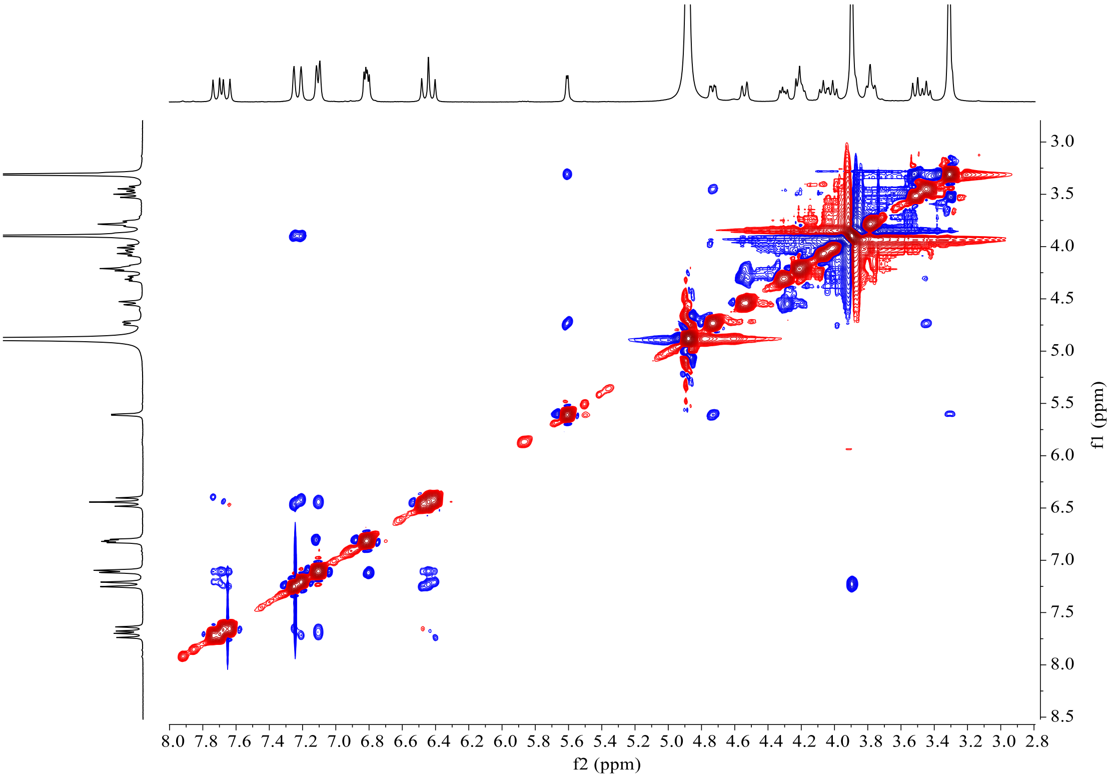

Figure S46. NOESY Spectrum of Compound **5** in MeOH-d_4

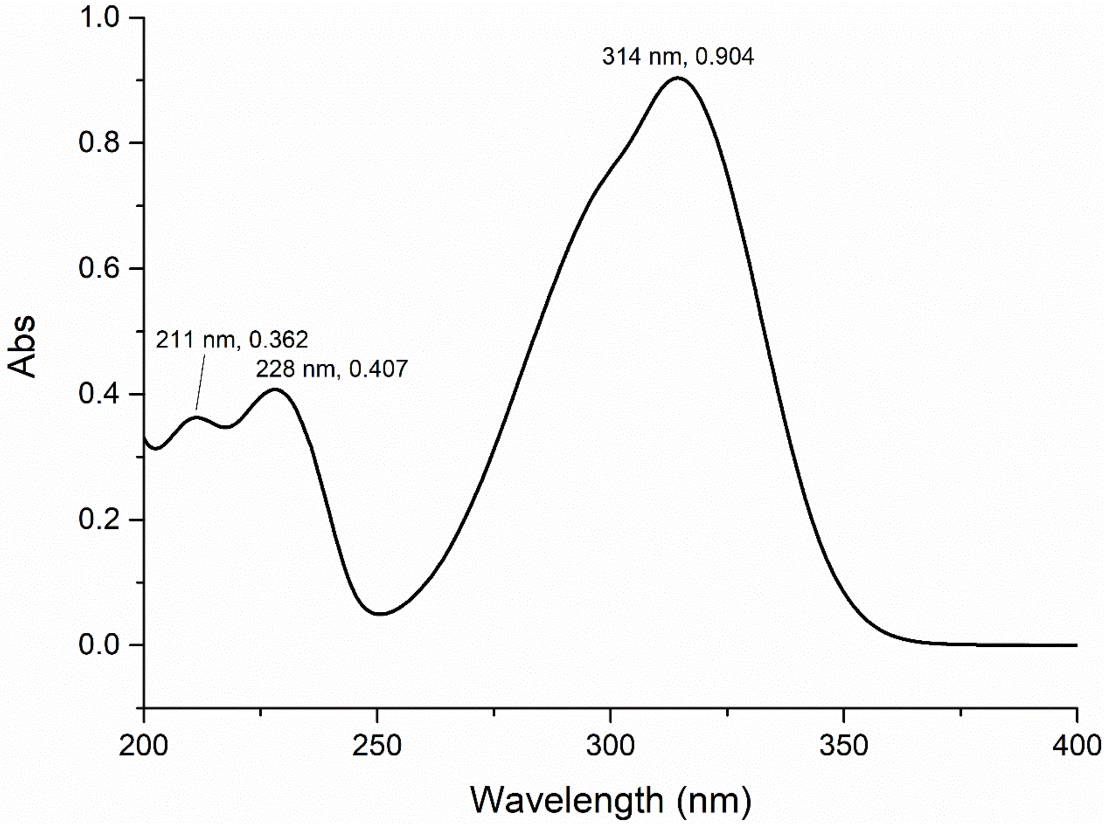

Figure S47. UV Spectrum of Compound **6**

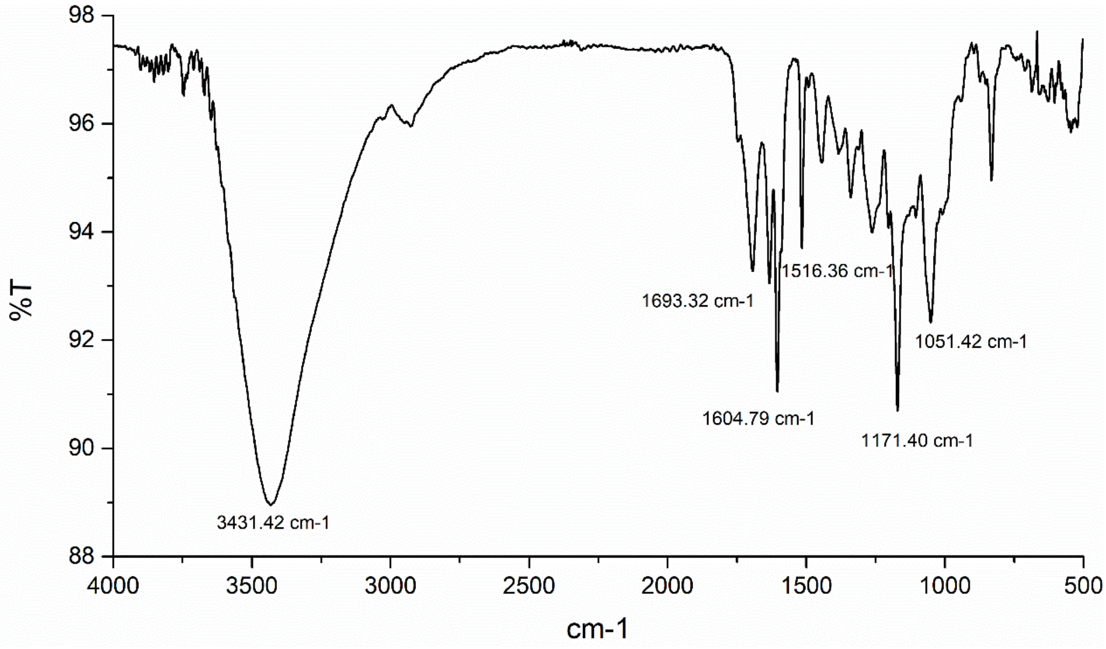

Figure S48. IR (KBr disc) Spectrum of Compound **6**

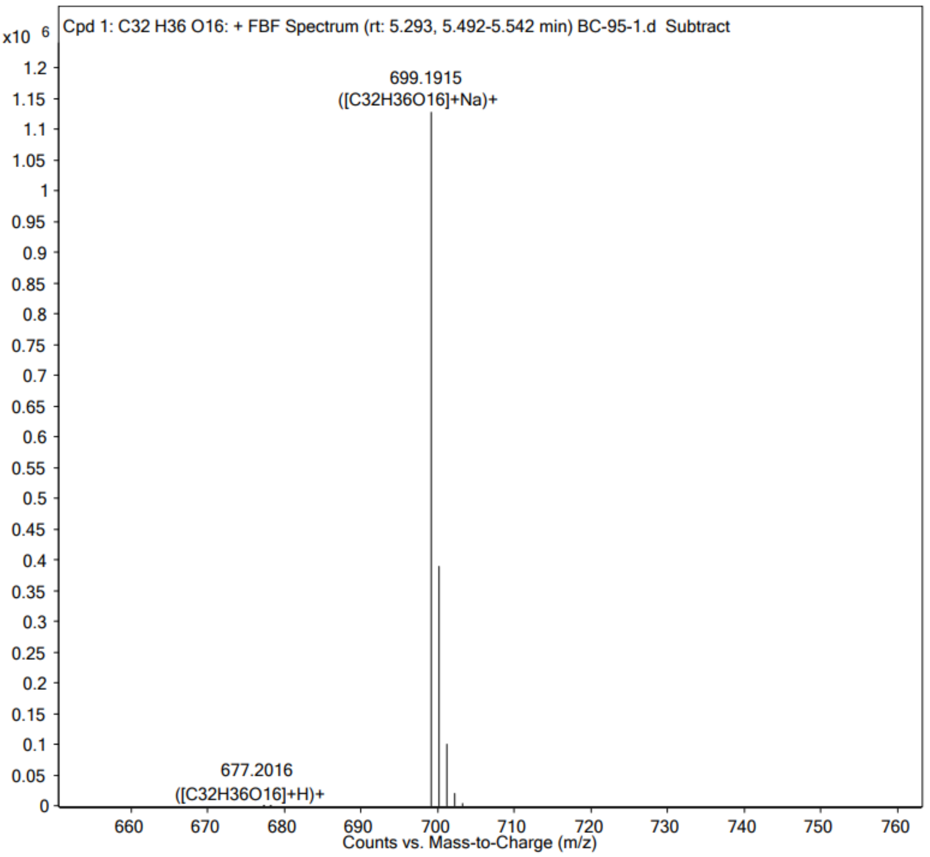

Figure S49. (+) HRESIMS Spectrum of Compound **6**

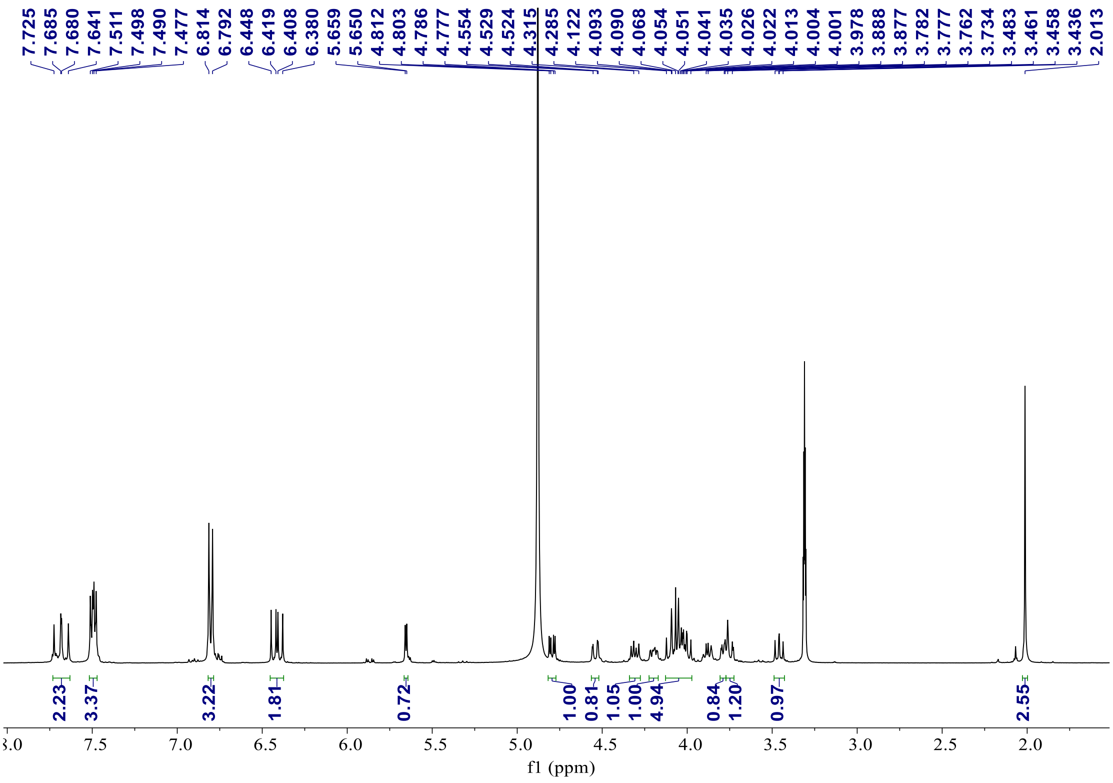

Figure S50. ^{1}H NMR Spectrum of Compound **6** in MeOH-d_4

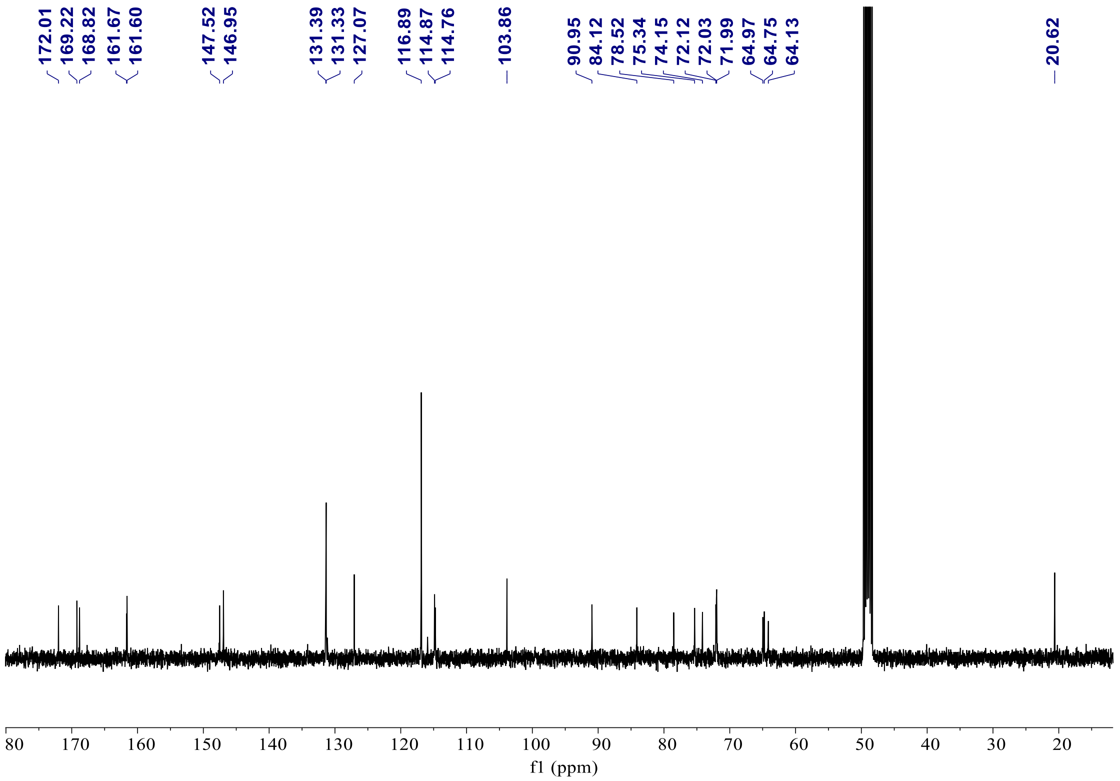

Figure S51. ^{13}C NMR Spectrum of Compound **6** in MeOH-d_4

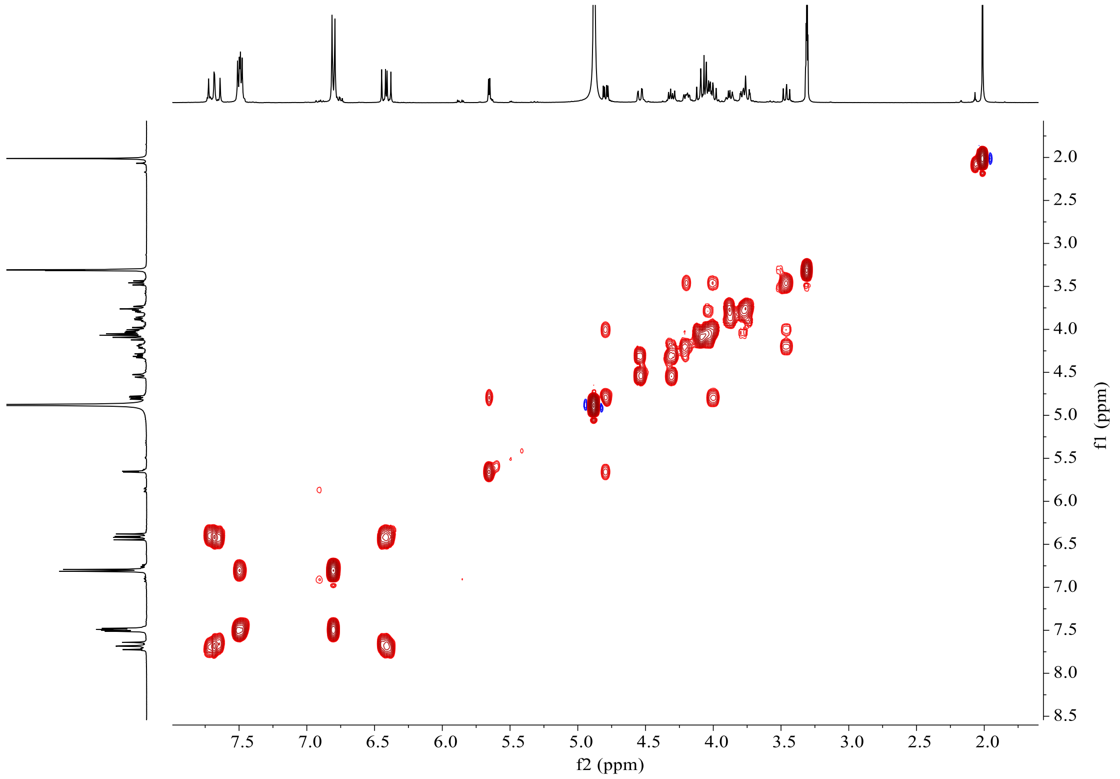

Figure S52. ^{1}H–^{1}H COSY Spectrum of Compound **6** in MeOH-d_4

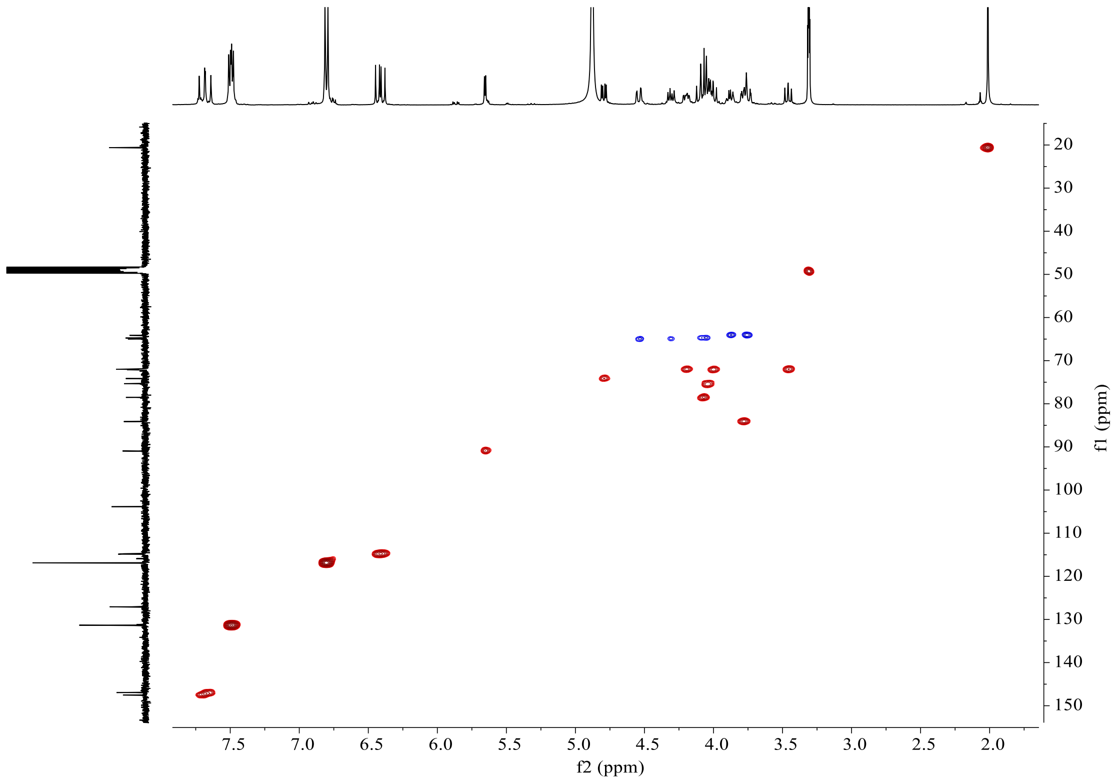

Figure S53. HSQC Spectrum of Compound **6** in MeOH-d_4

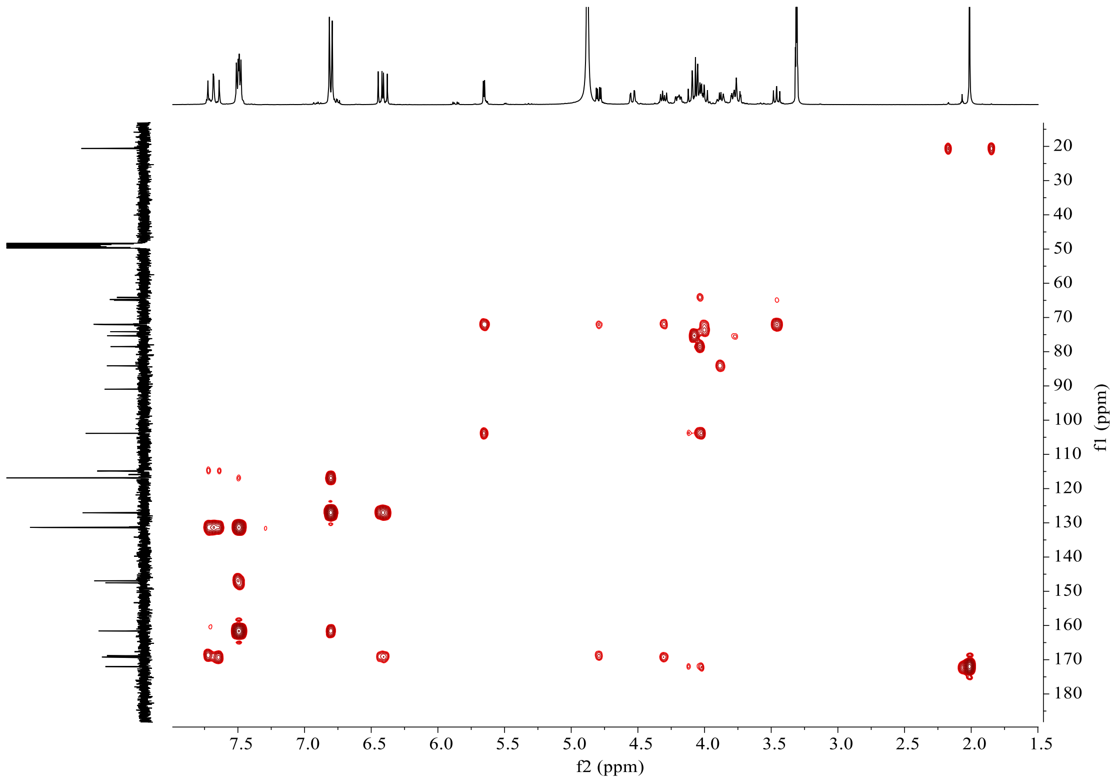

Figure S54. HMBC Spectrum of Compound **6** in MeOH-d_4

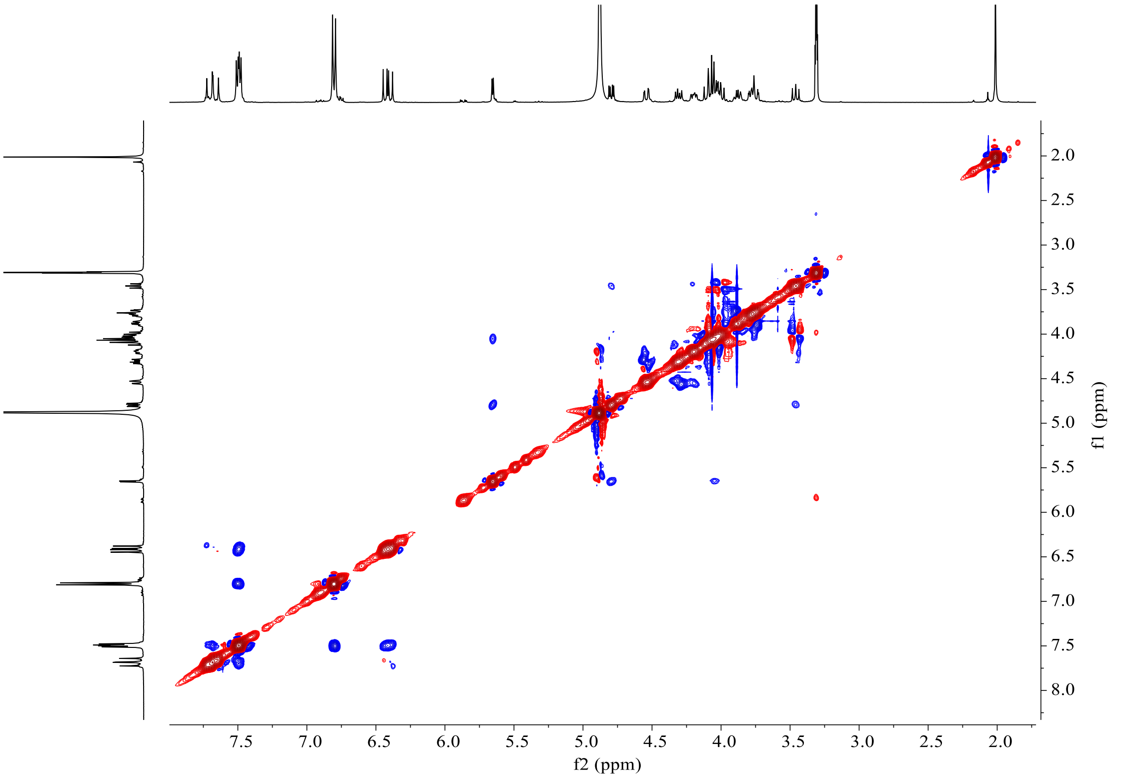

Figure S55. NOESY Spectrum of Compound **6** in MeOH-d_4

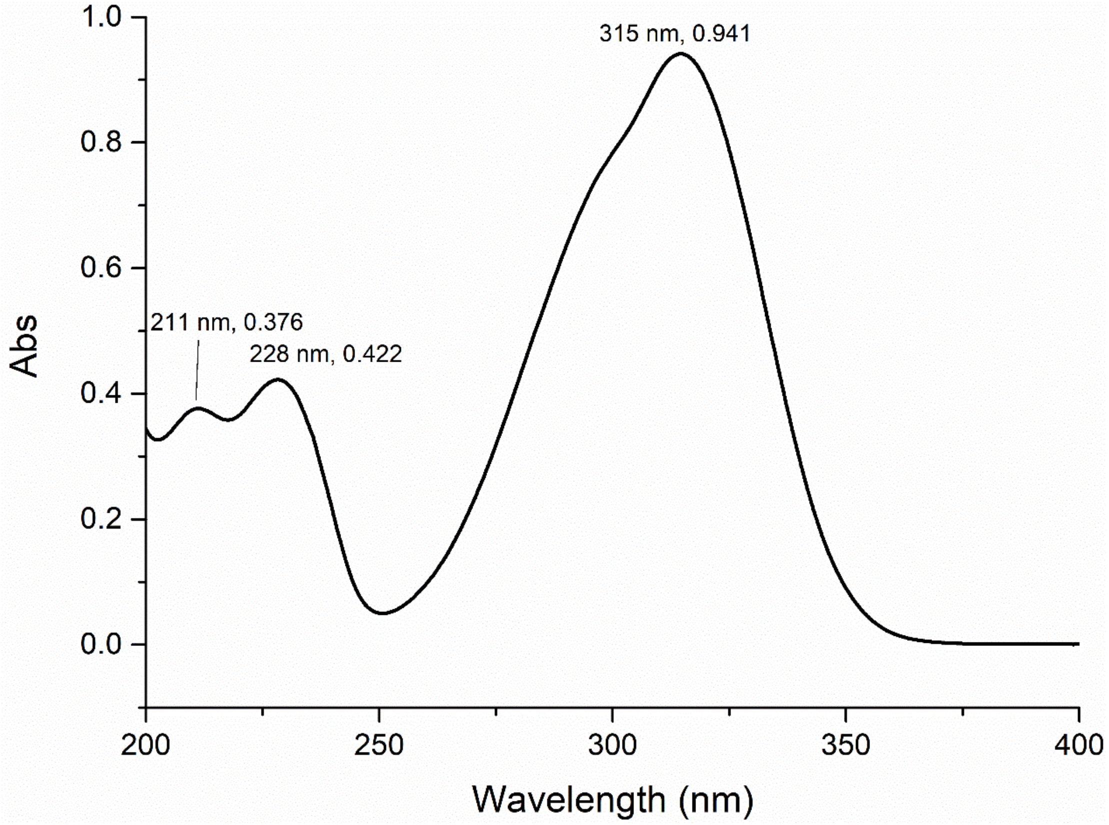

Figure S56. UV Spectrum of Compound **7**

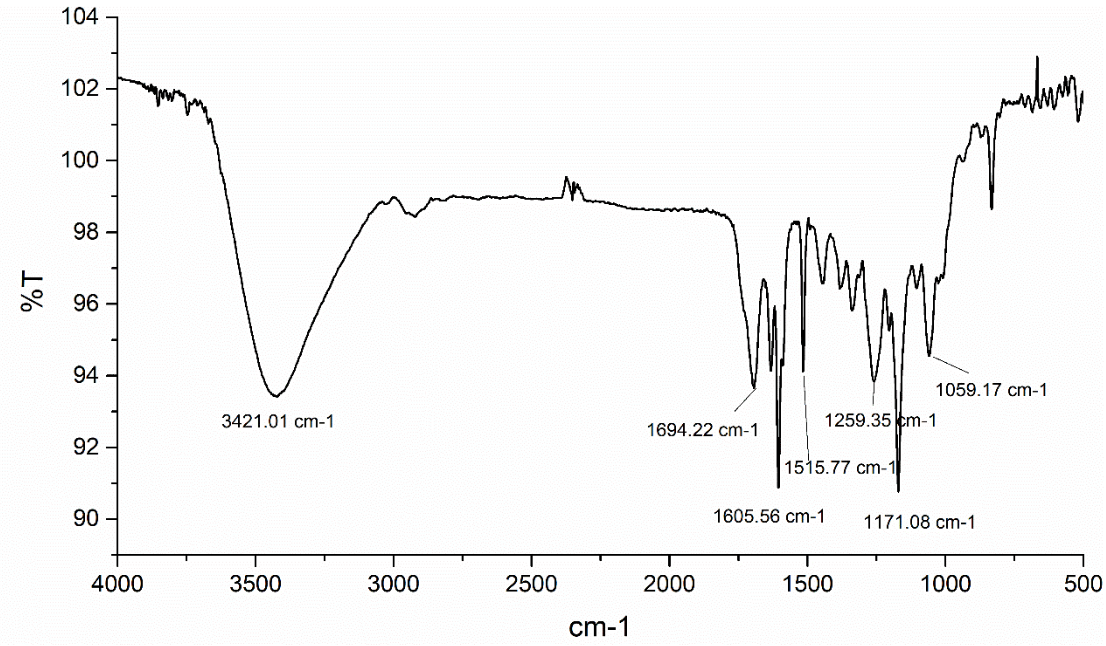

Figure S57. IR (KBr disc) Spectrum of Compound **7**

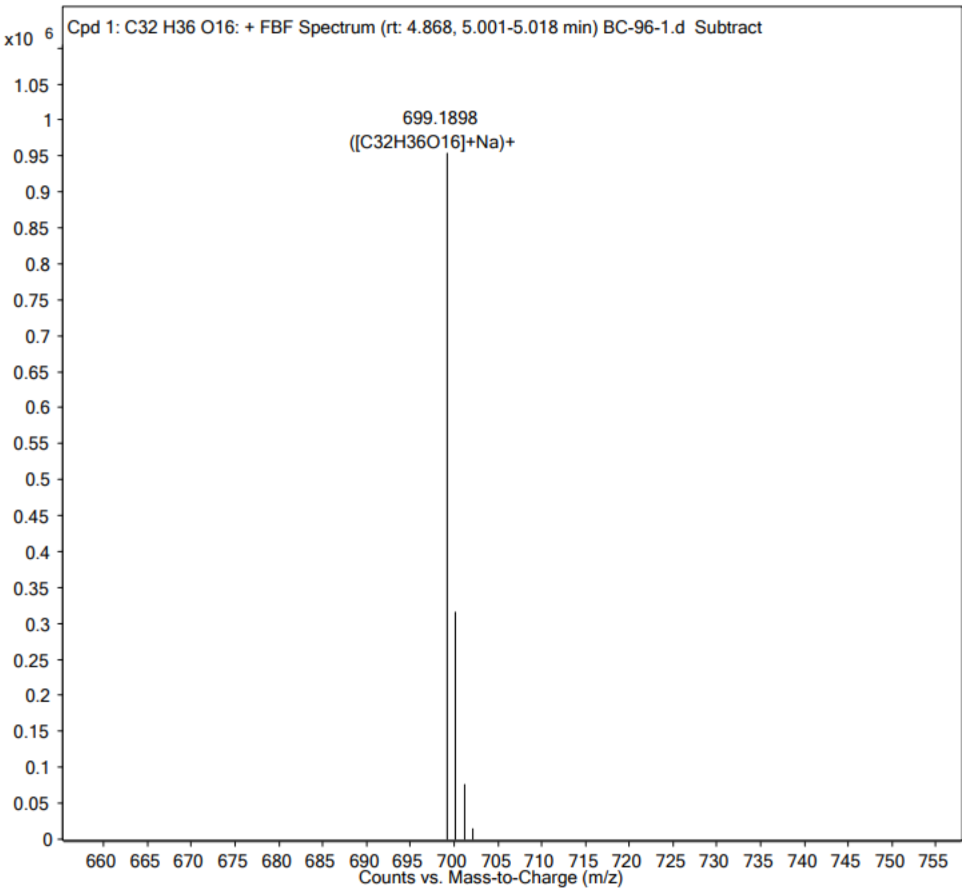

Figure S58. (+) HRESIMS Spectrum of Compound **7**

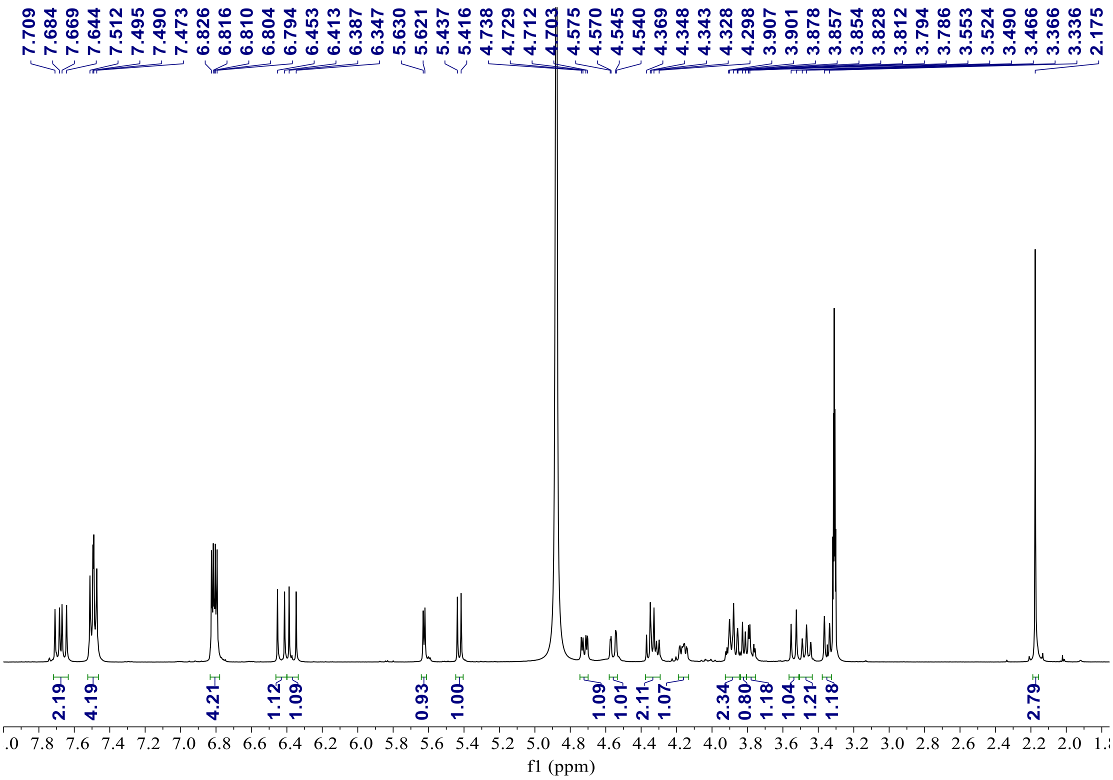

Figure S59. ^{1}H NMR Spectrum of Compound **7** in MeOH-d_4

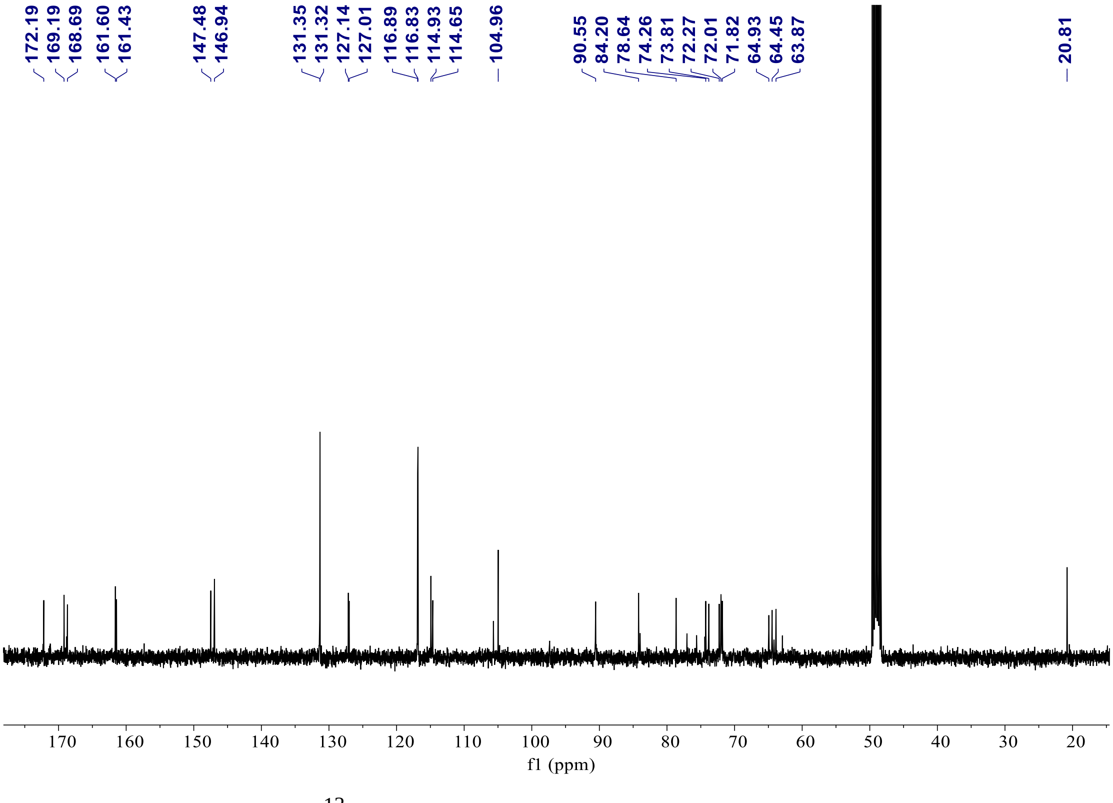

Figure S60. ^{13}C NMR Spectrum of Compound **7** in MeOH-d_4

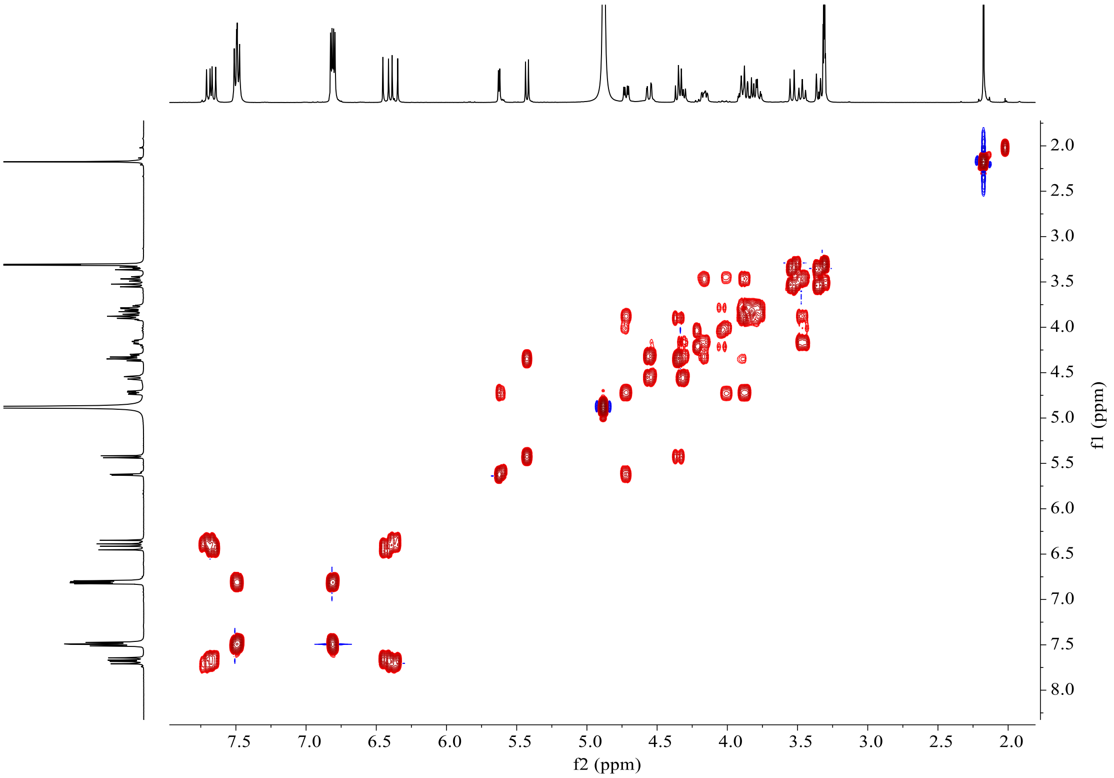

Figure S61. ^1H–^1H COSY Spectrum of Compound **7** in MeOH-d_4

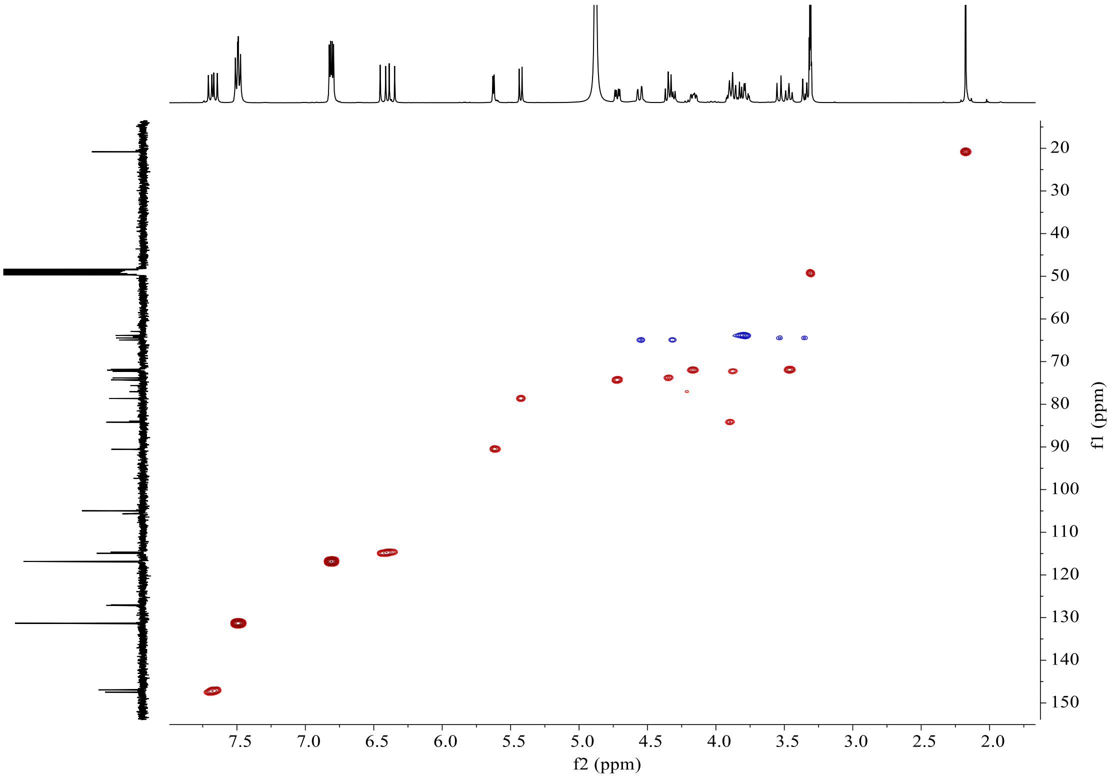

Figure S62. HSQC Spectrum of Compound **7** in MeOH-d_4

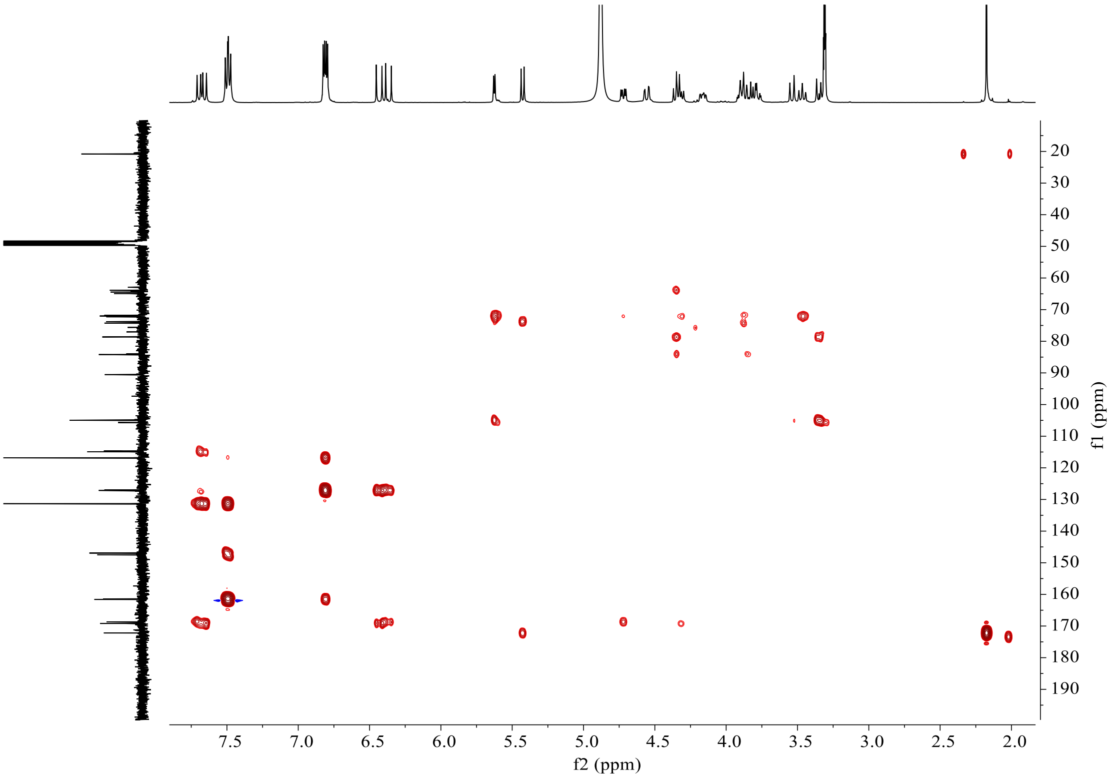

Figure S63. HMBC Spectrum of Compound **7** in MeOH-d_4

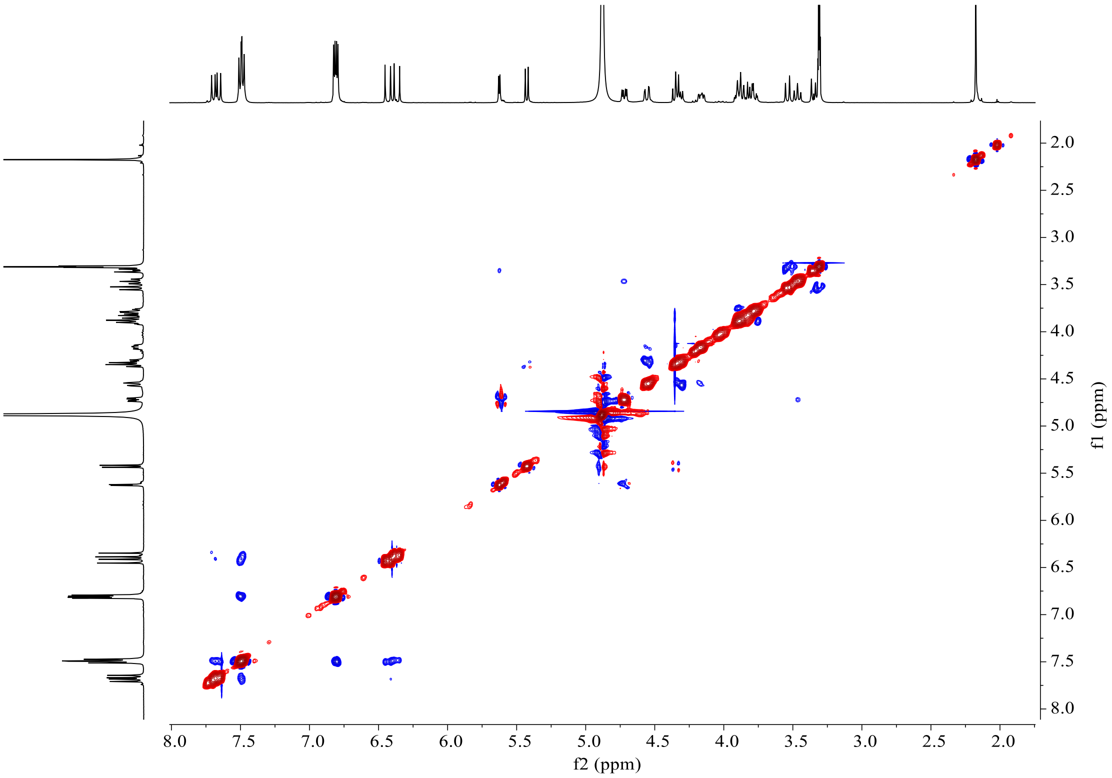

Figure S64. NOESY Spectrum of Compound **7** in MeOH-d_4

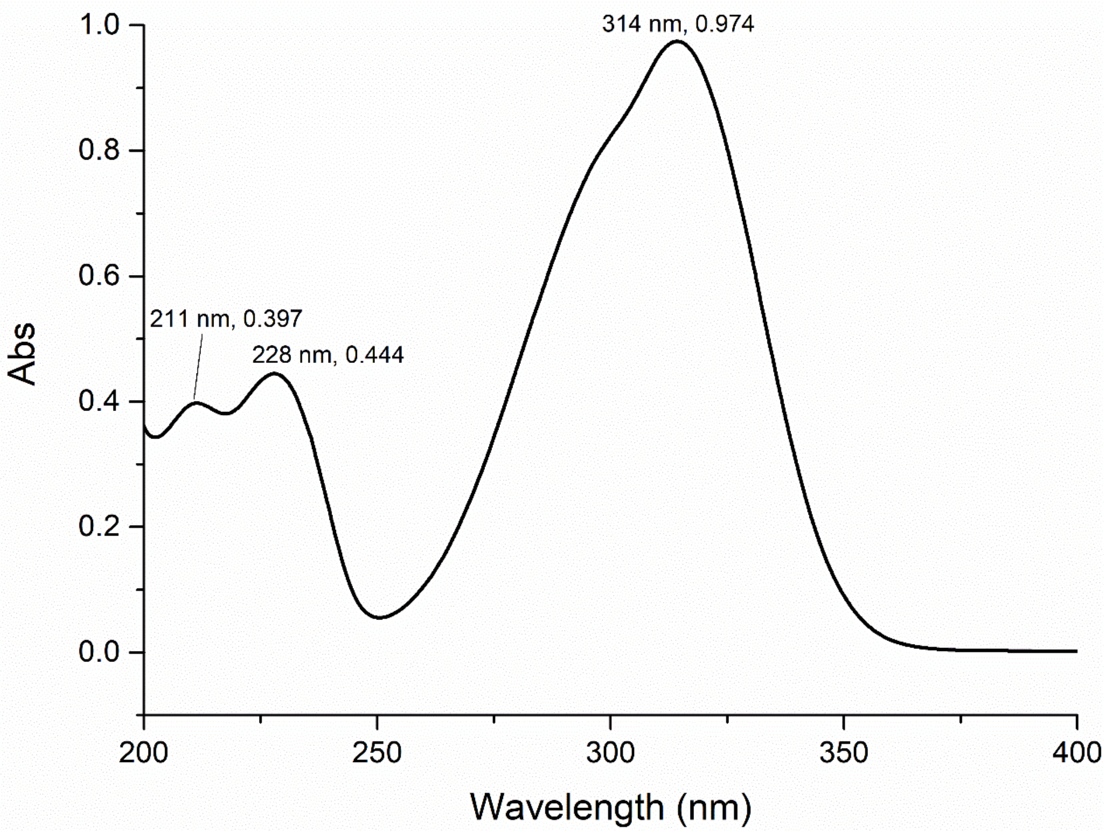

Figure S65. UV Spectrum of Compound **8**

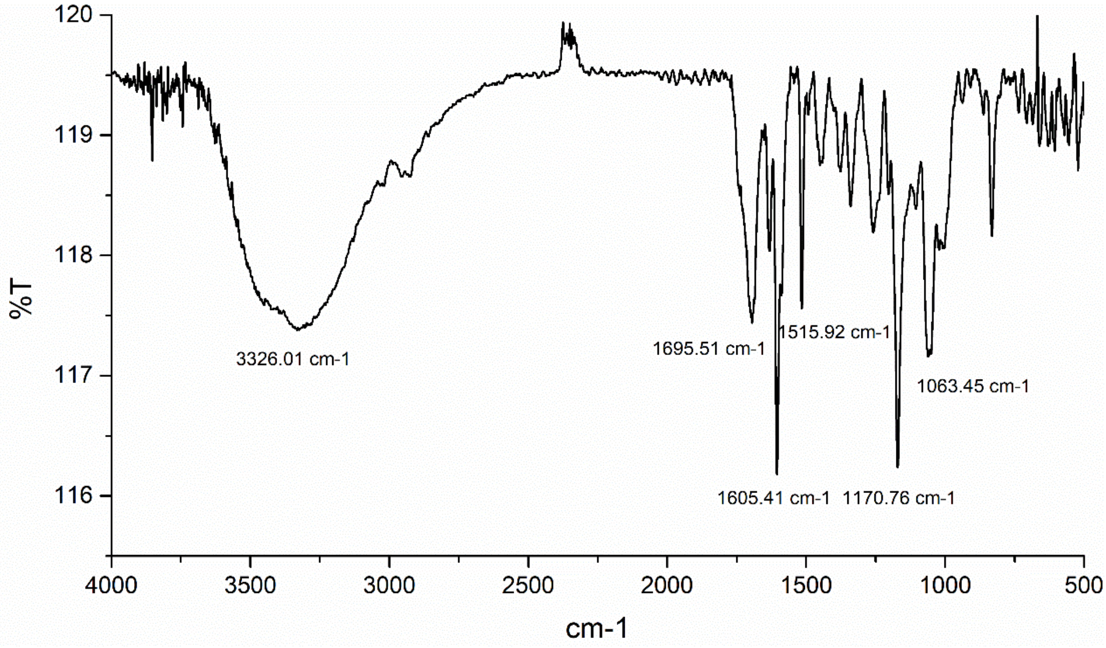

Figure S66. IR (KBr disc) Spectrum of Compound **8**

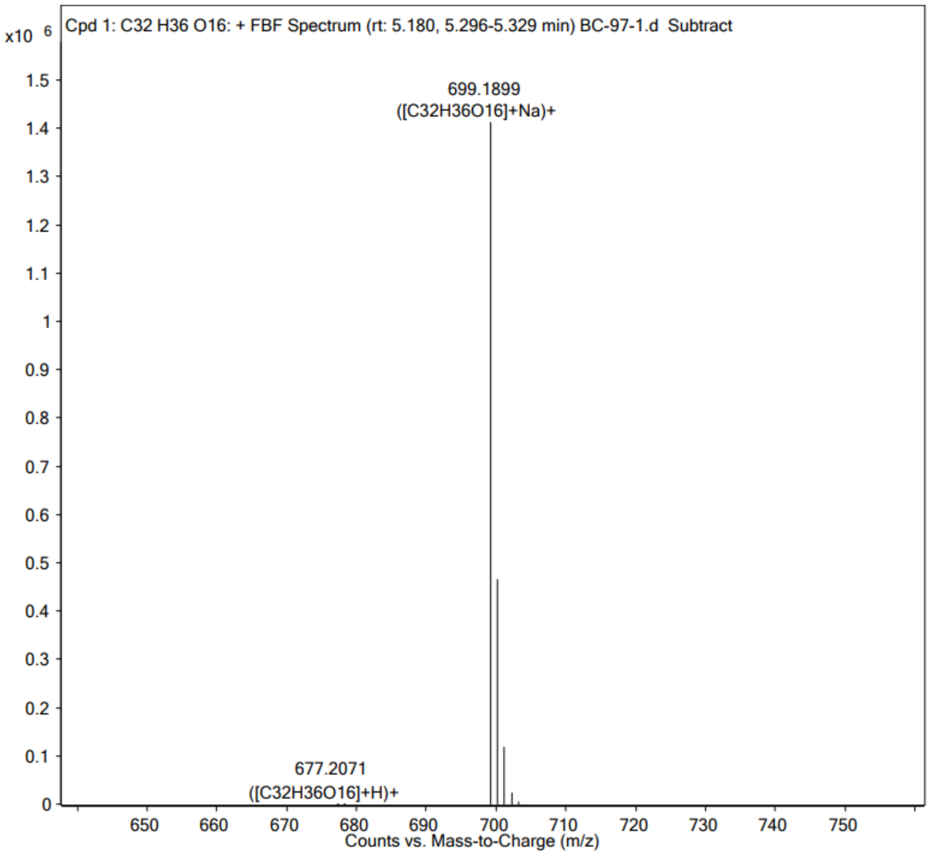

Figure S67. (+) HRESIMS Spectrum of Compound **8**

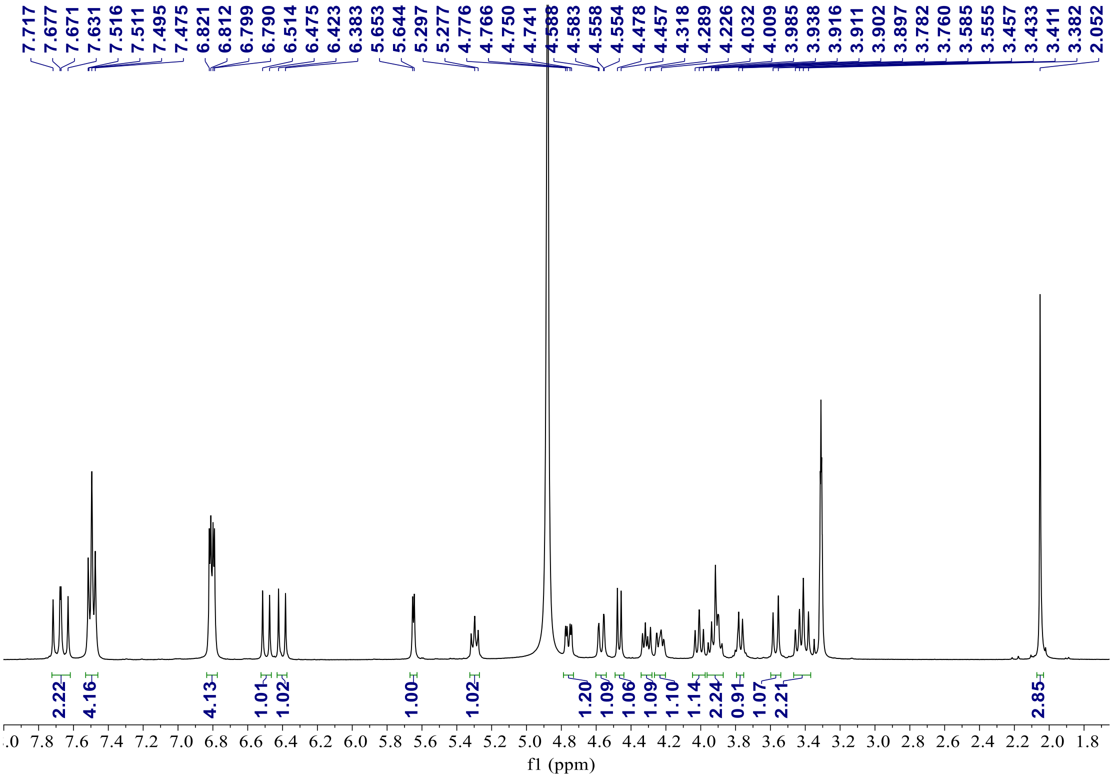

Figure S68. ^{1}H NMR Spectrum of Compound **8** in MeOH-d_4

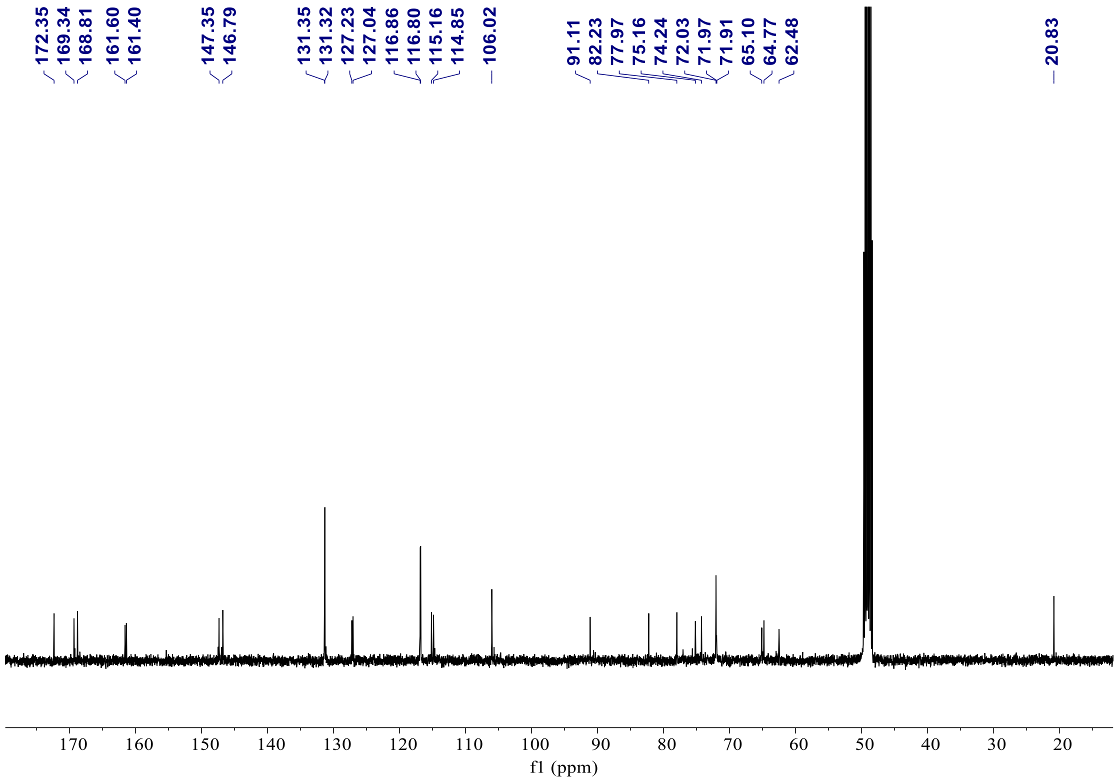

Figure S69. ^{13}C NMR Spectrum of Compound **8** in MeOH-d_4

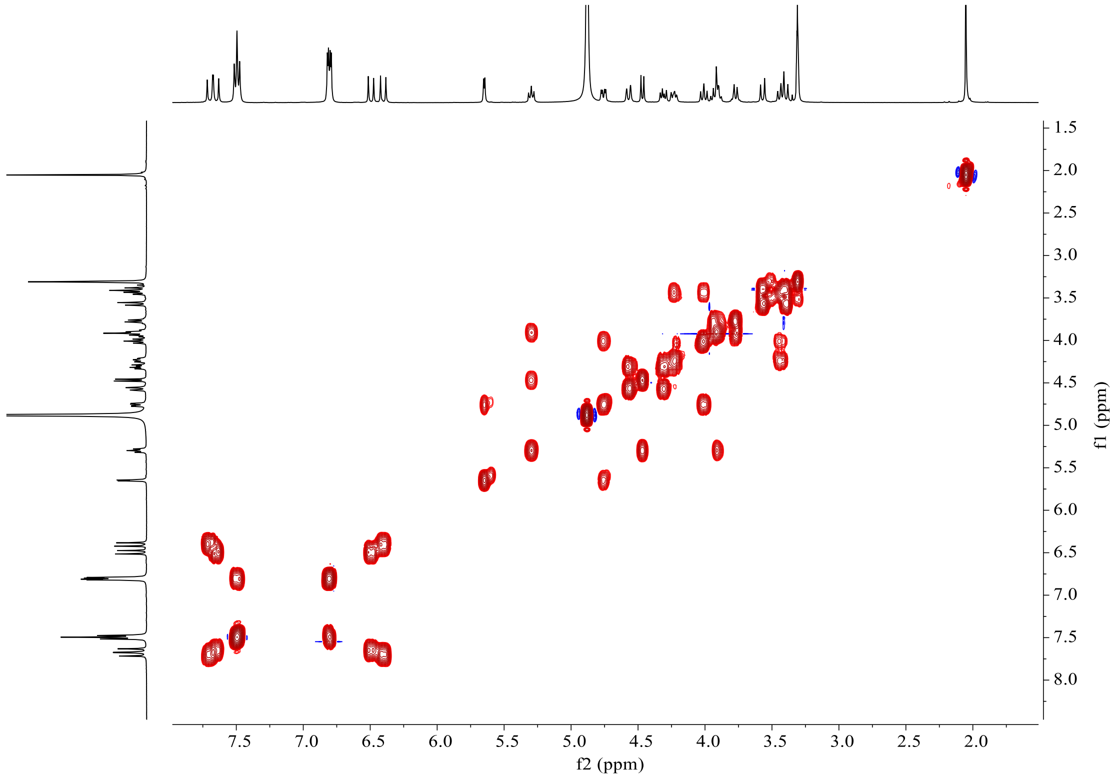

Figure S70. ^{1}H–^{1}H COSY Spectrum of Compound **8** in MeOH-d_4

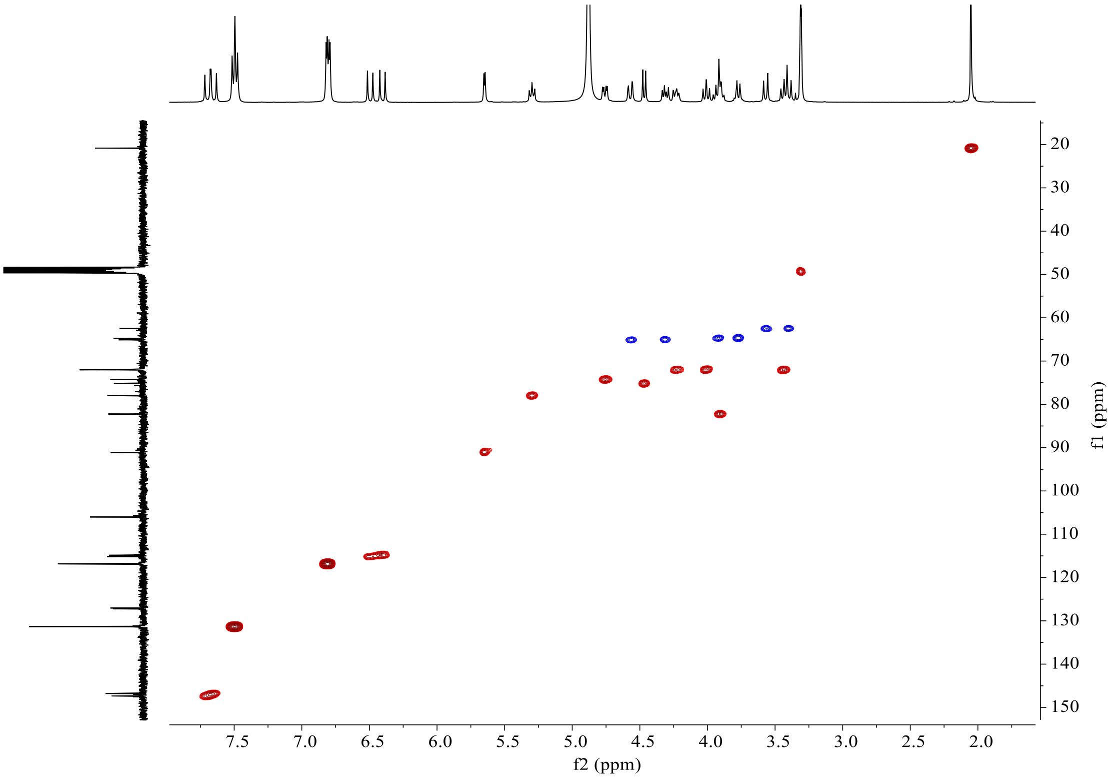

Figure S71. HSQC Spectrum of Compound **8** in MeOH-d_4

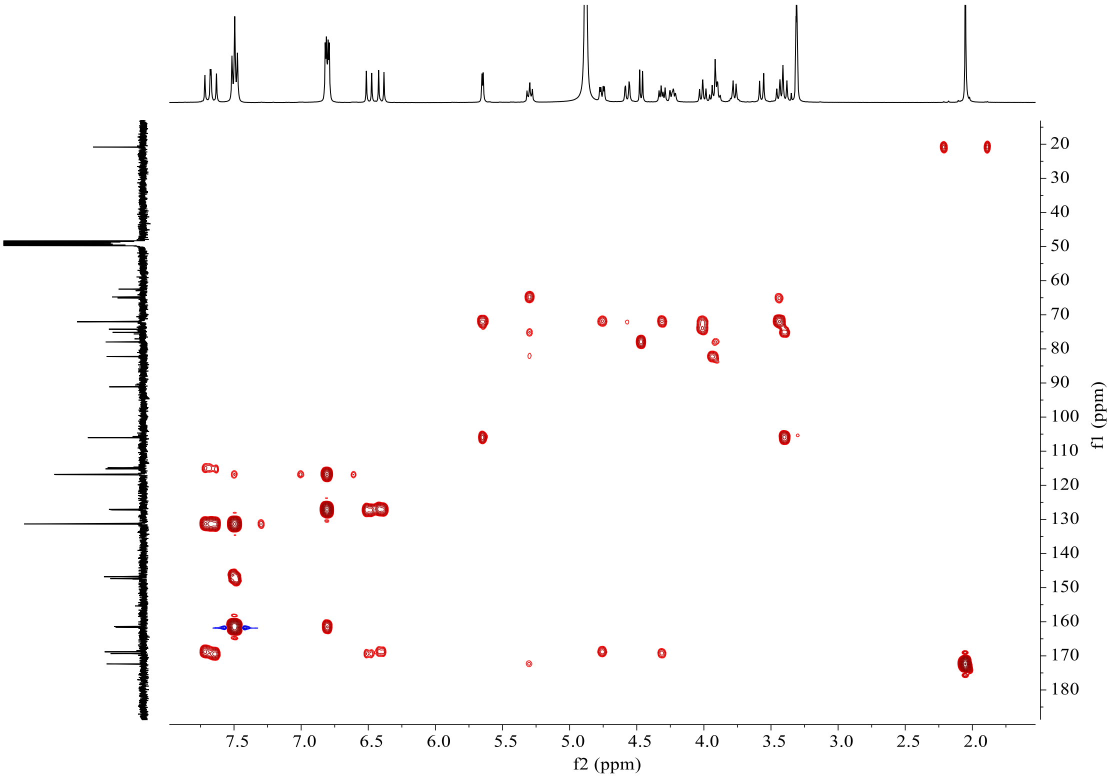

Figure S72. HMBC Spectrum of Compound **8** in MeOH-d_4

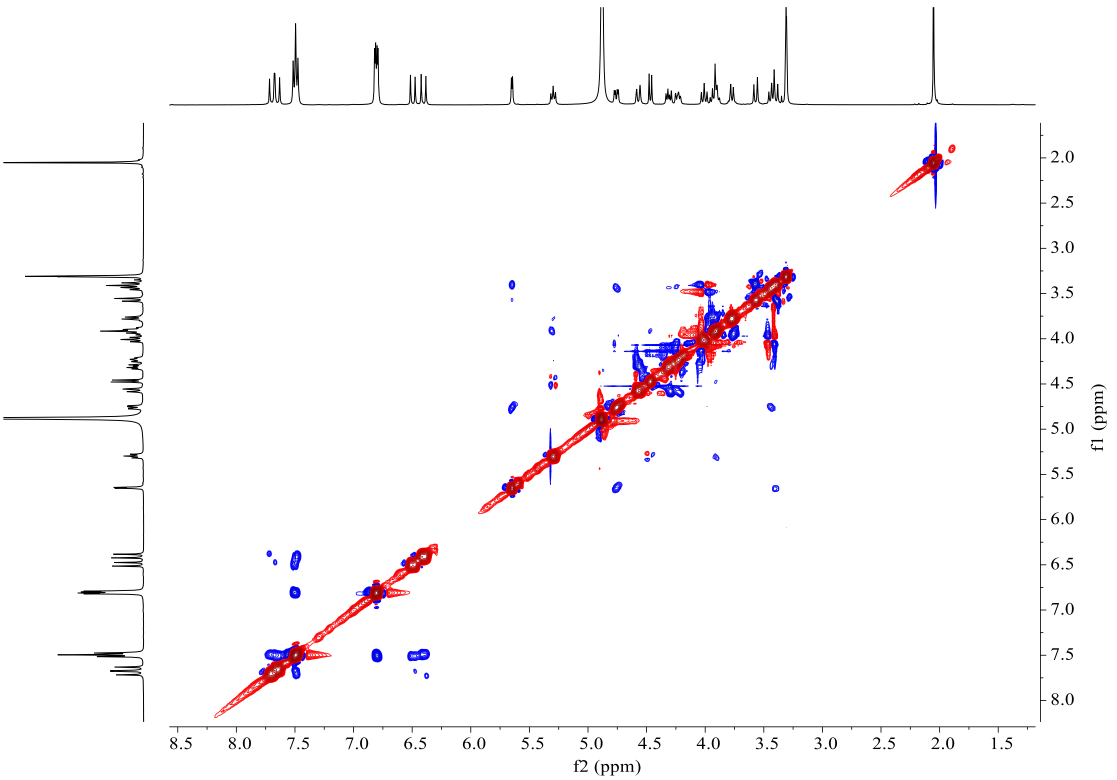

Figure S73. NOESY Spectrum of Compound **8** in MeOH-d_4

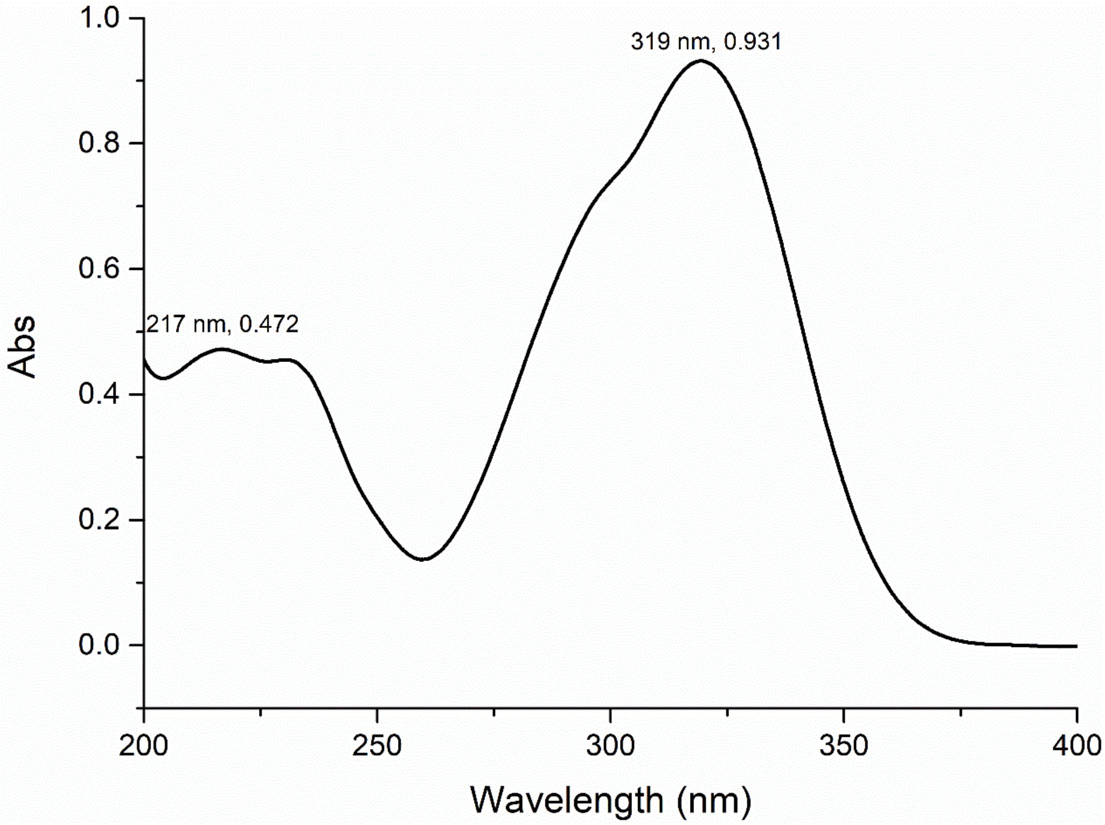

Figure S74. UV Spectrum of Compound **9**

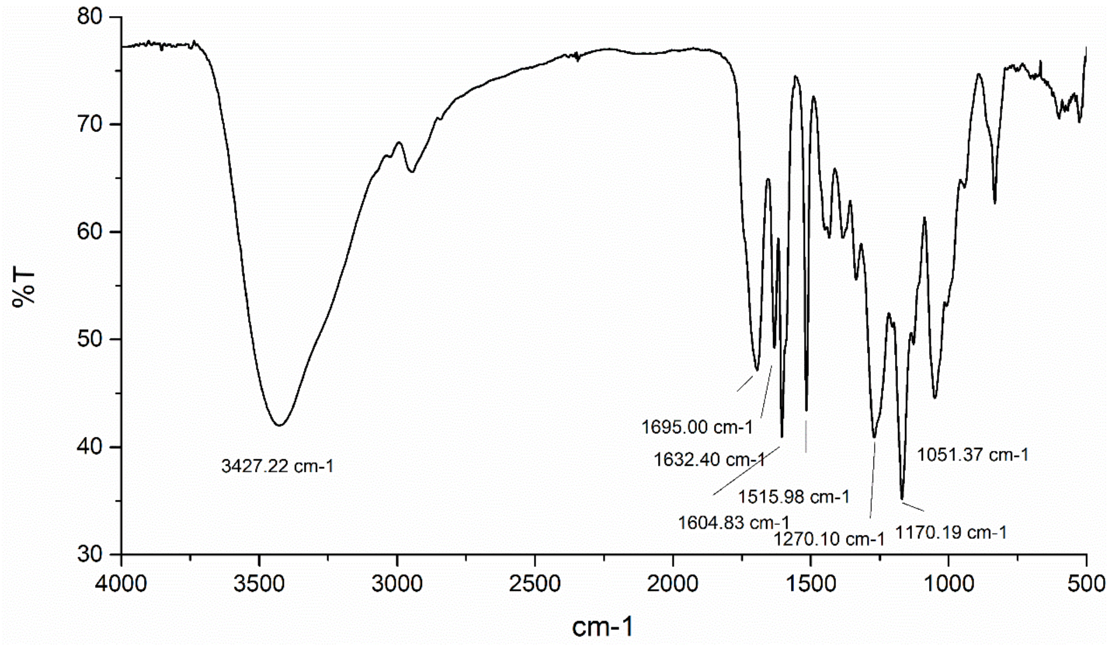

Figure S75. IR (KBr disc) Spectrum of Compound **9**

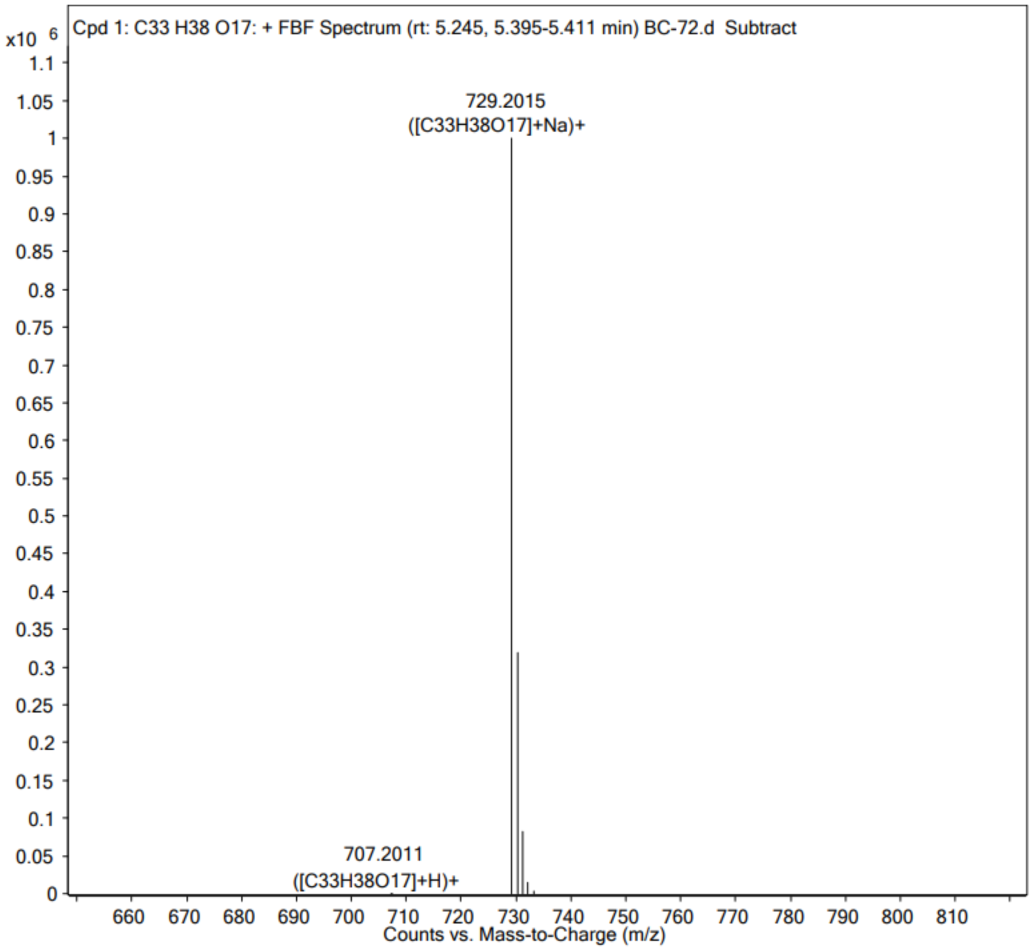

Figure S76. (+) HRESIMS Spectrum of Compound **9**

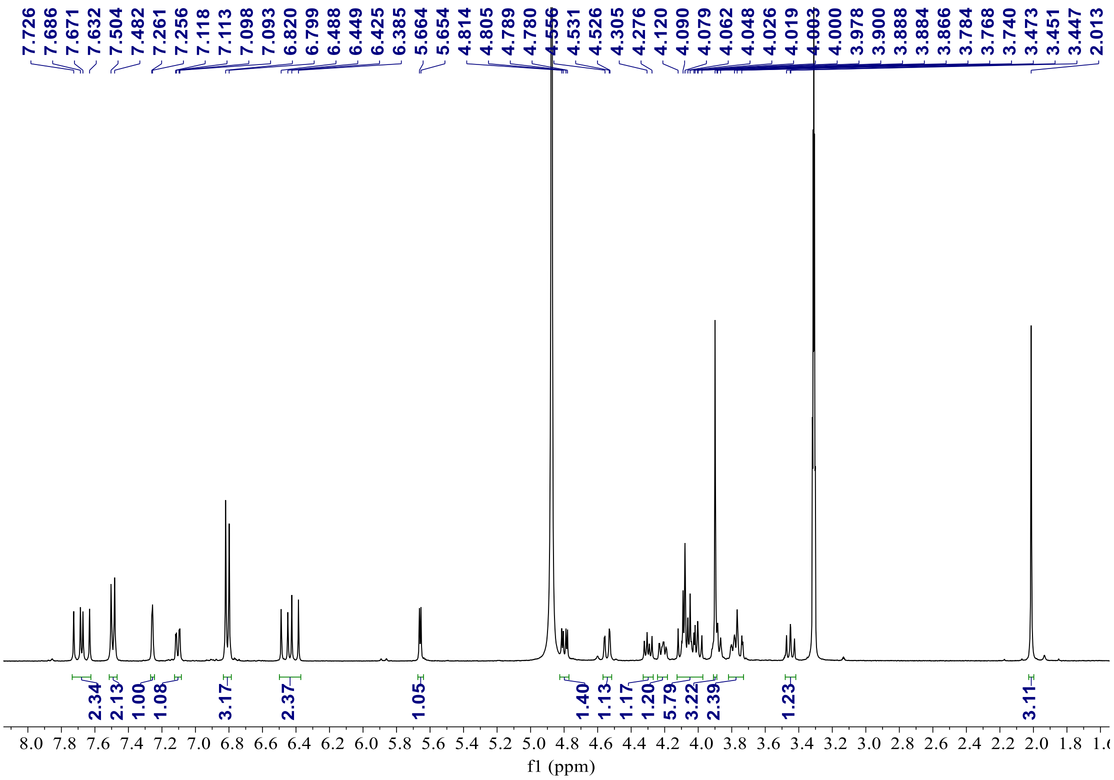

Figure S77. ^{1}H NMR Spectrum of Compound **9** in MeOH-d_4

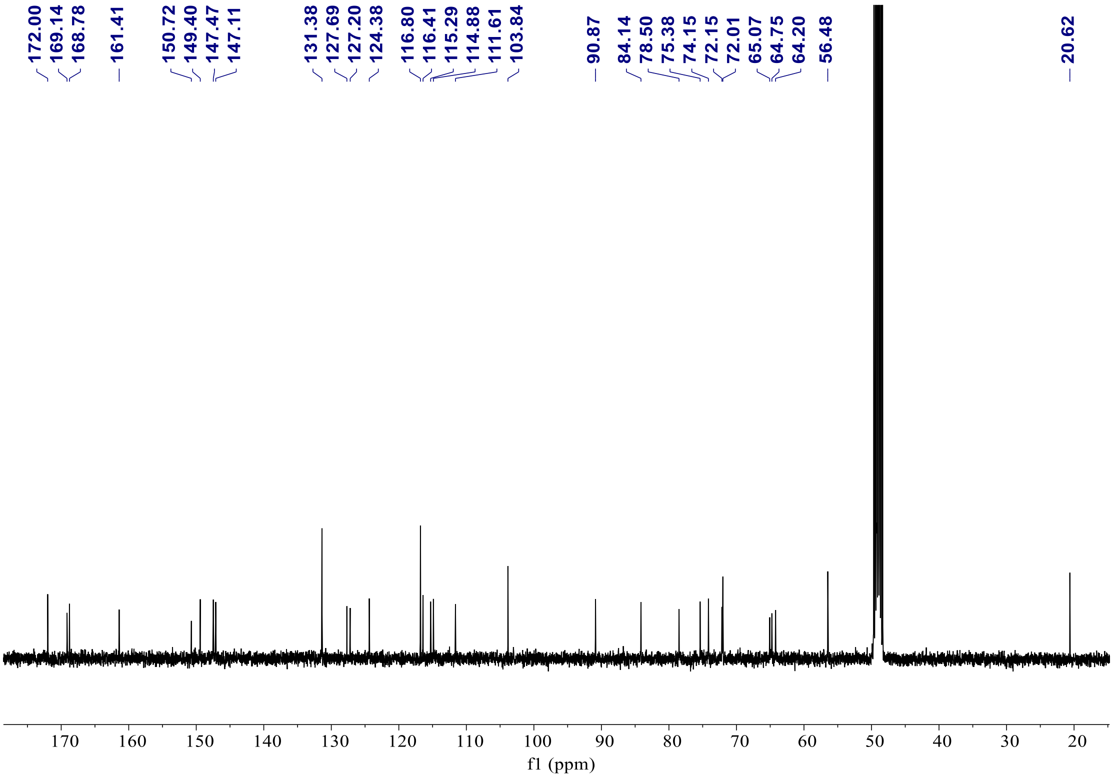

Figure S78. ^{13}C NMR Spectrum of Compound **9** in MeOH-d_4

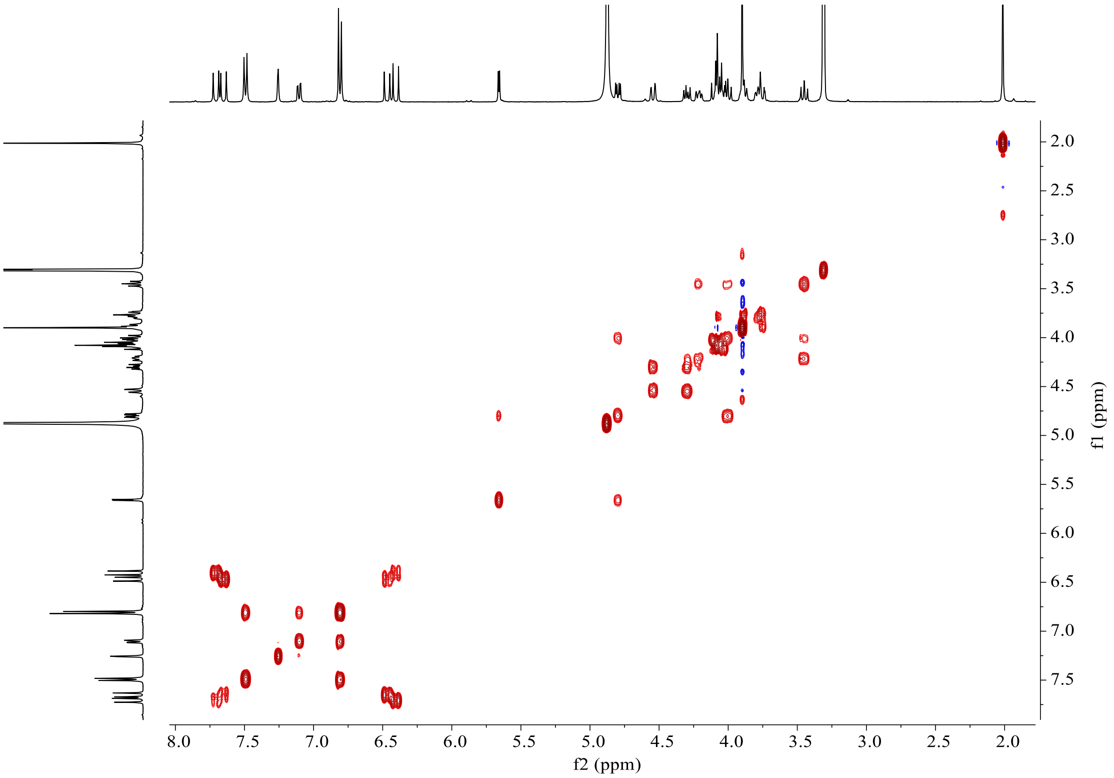

Figure S79. $^{1}H–^{1}H$ COSY Spectrum of Compound **9** in MeOH-d_4

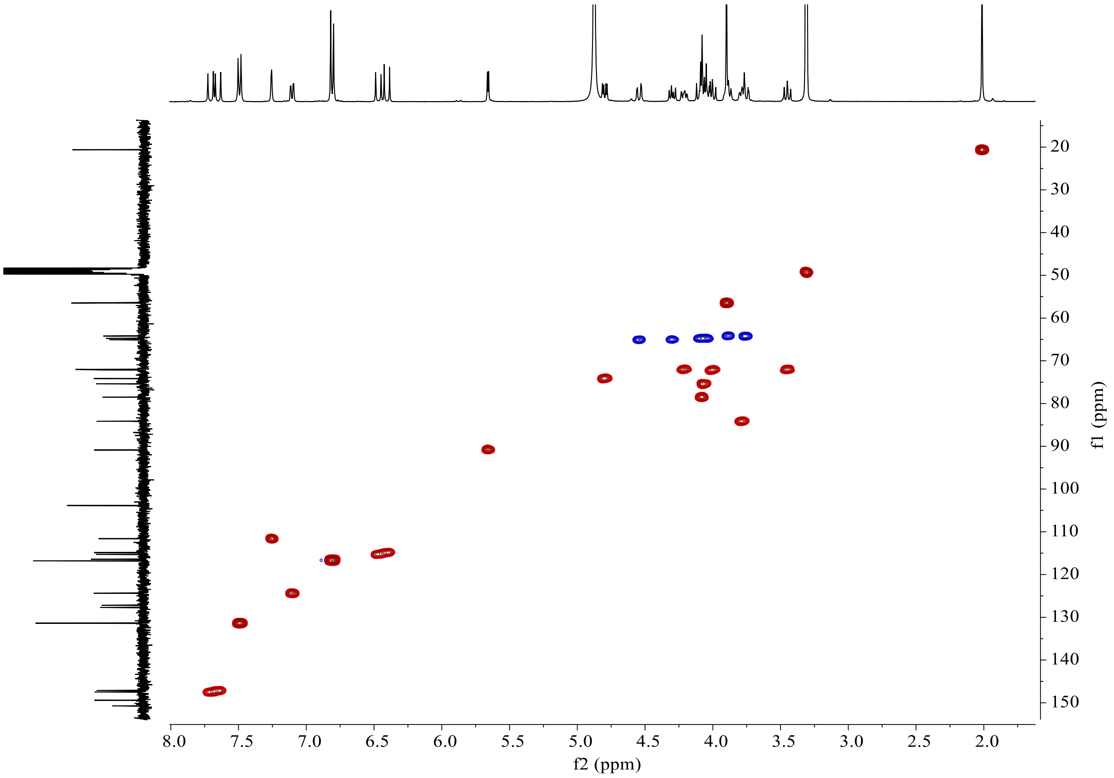

Figure S80. HSQC Spectrum of Compound **9** in MeOH-d_4

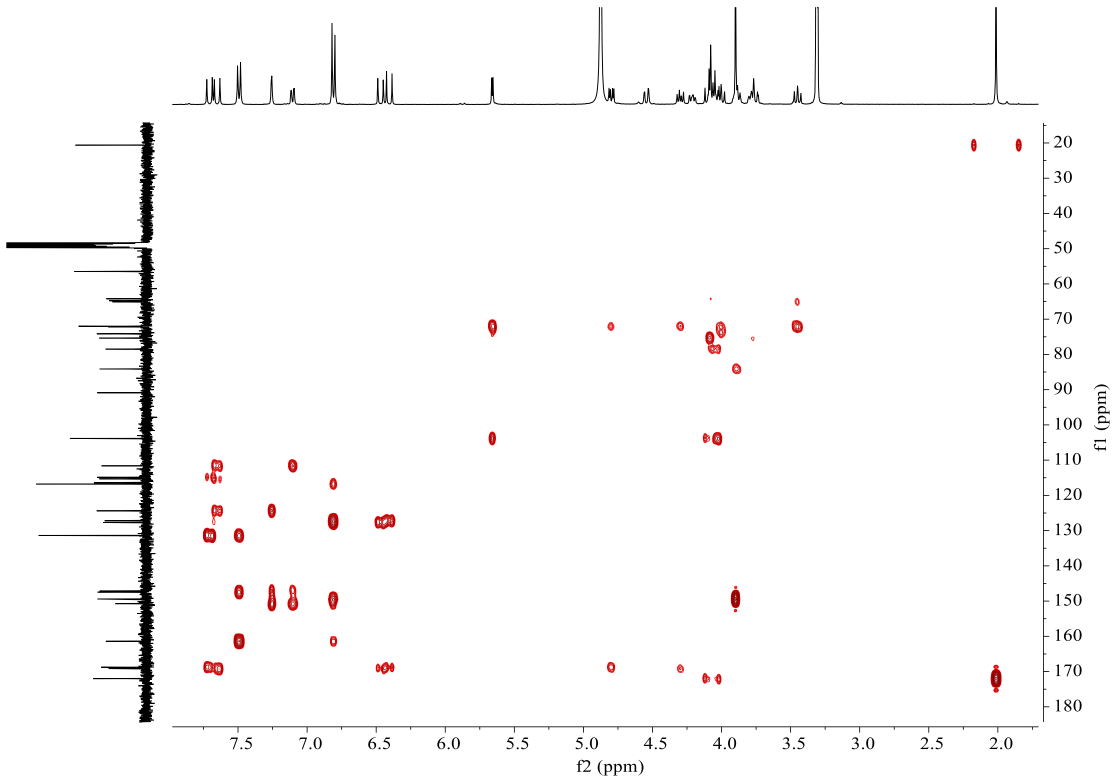

Figure S81. HMBC Spectrum of Compound **9** in MeOH-d_4

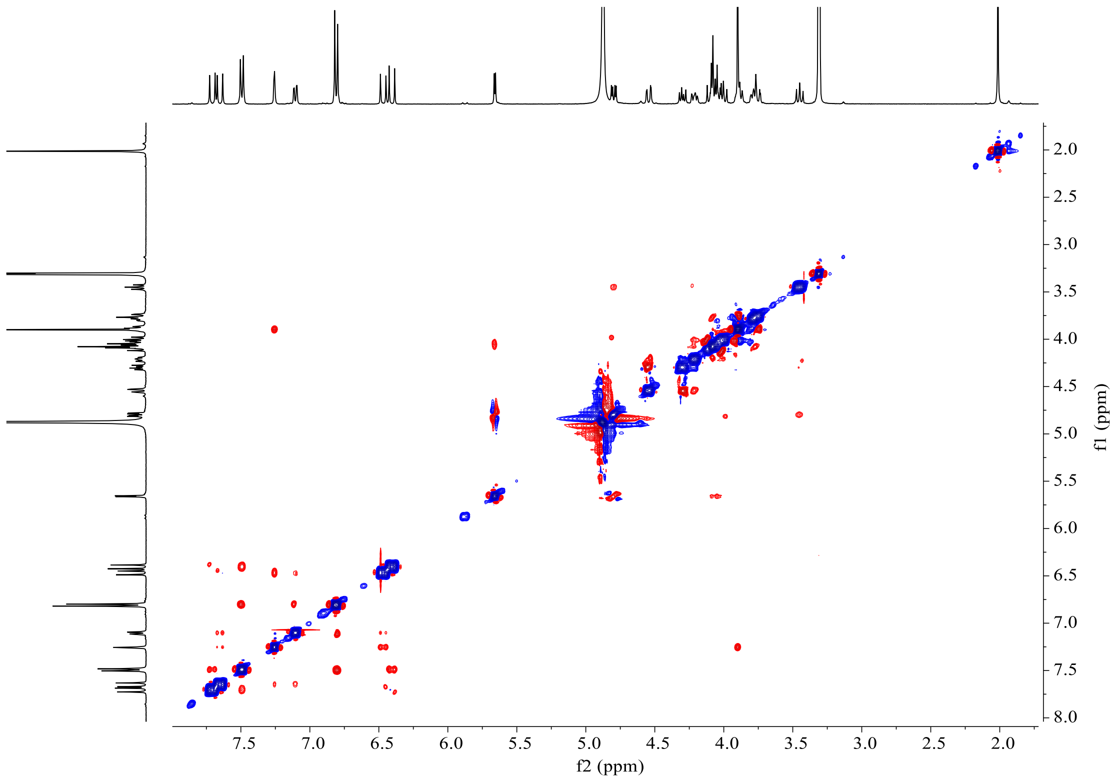

Figure S82. NOESY Spectrum of Compound **9** in MeOH-d_4

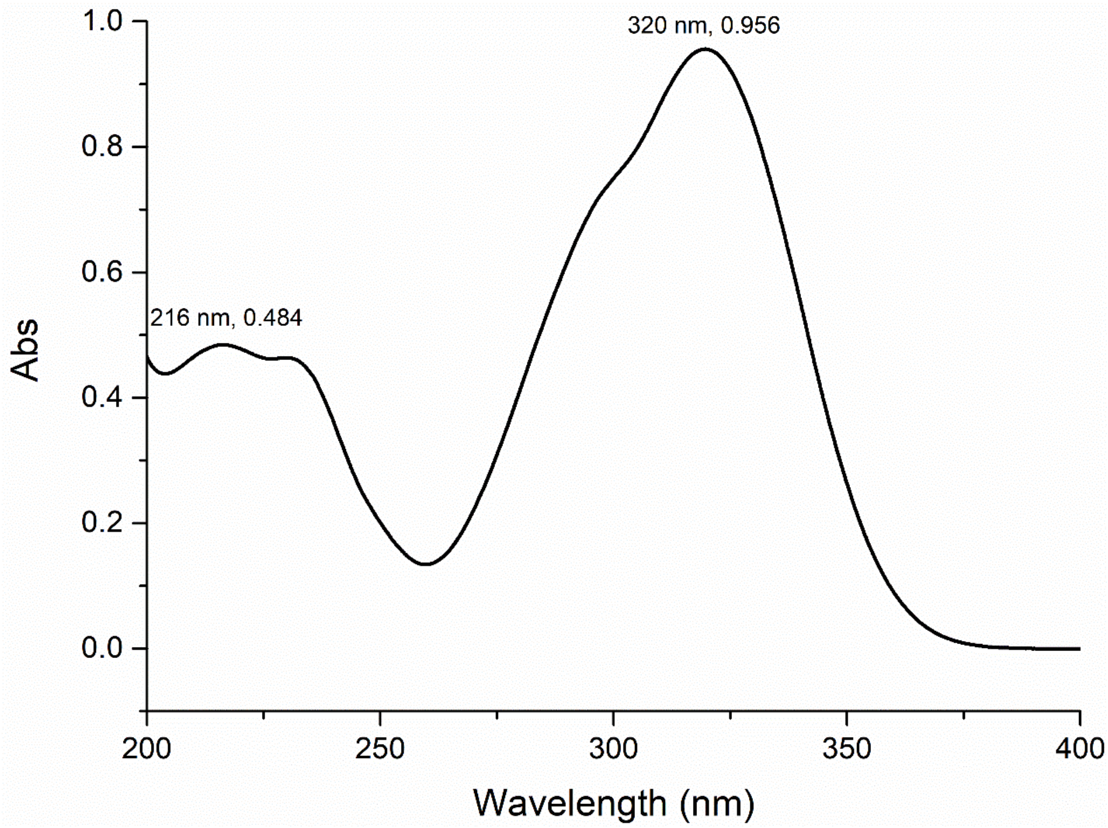

Figure S83. UV Spectrum of Compound **10**

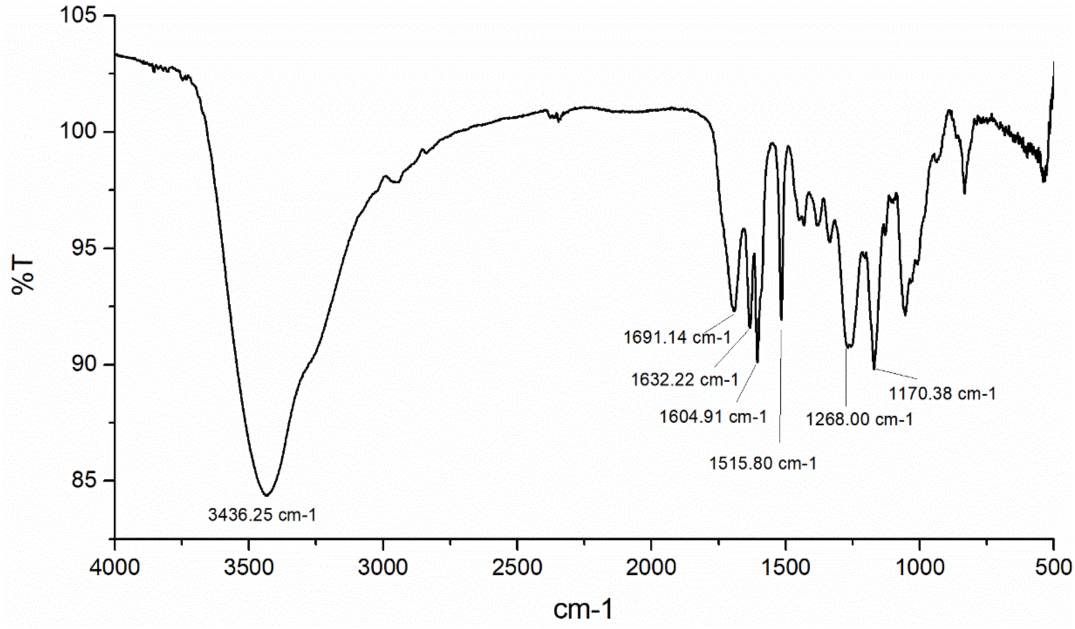

Figure S84. IR (KBr disc) Spectrum of Compound **10**

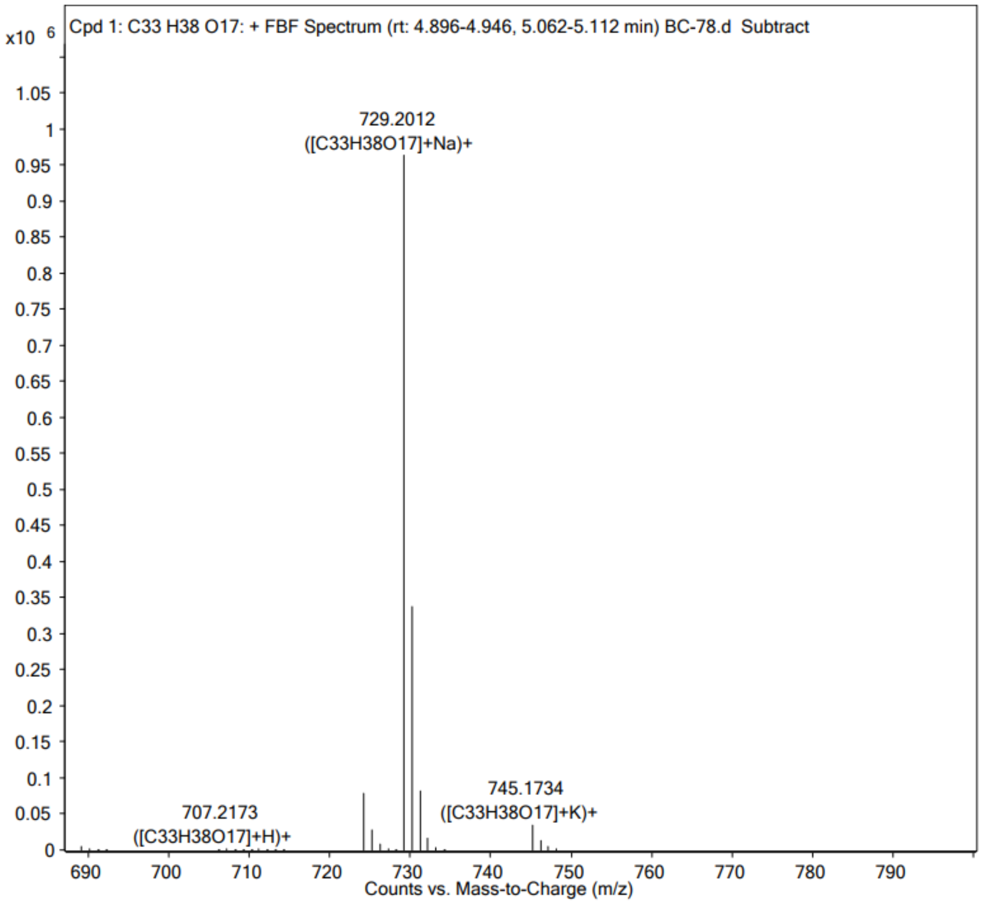

Figure S85. (+) HRESIMS Spectrum of Compound **10**

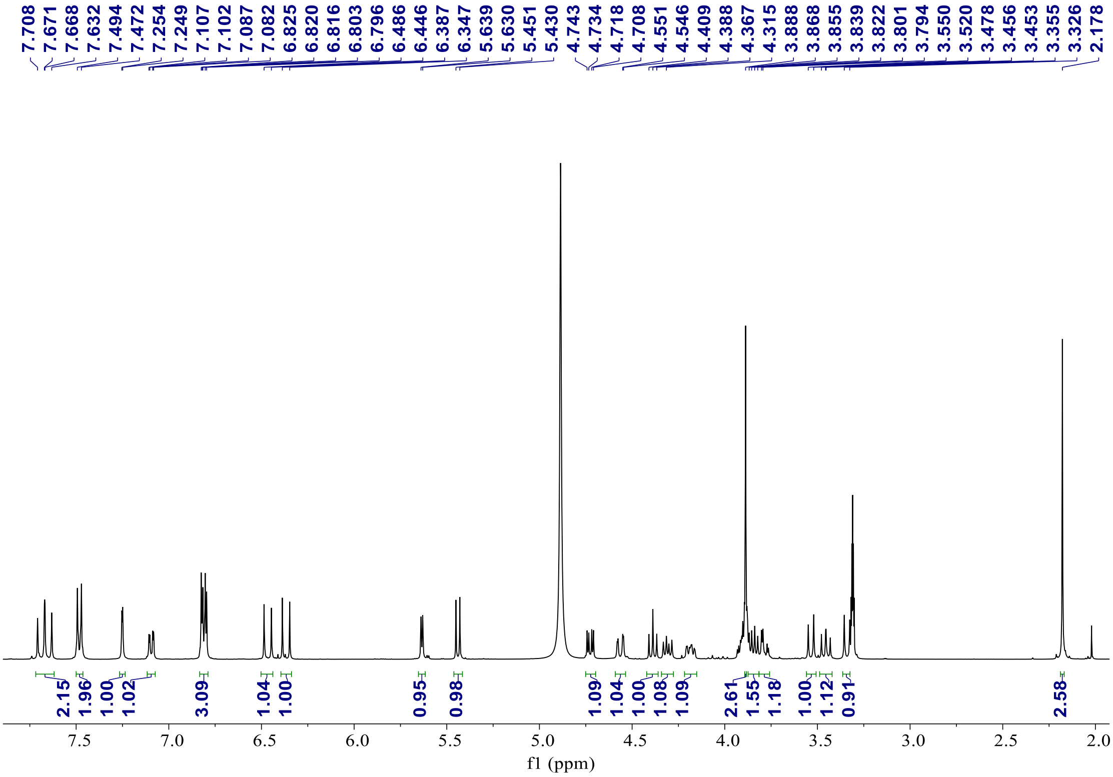

Figure S86. ^{1}H NMR Spectrum of Compound **10** in MeOH-d_4

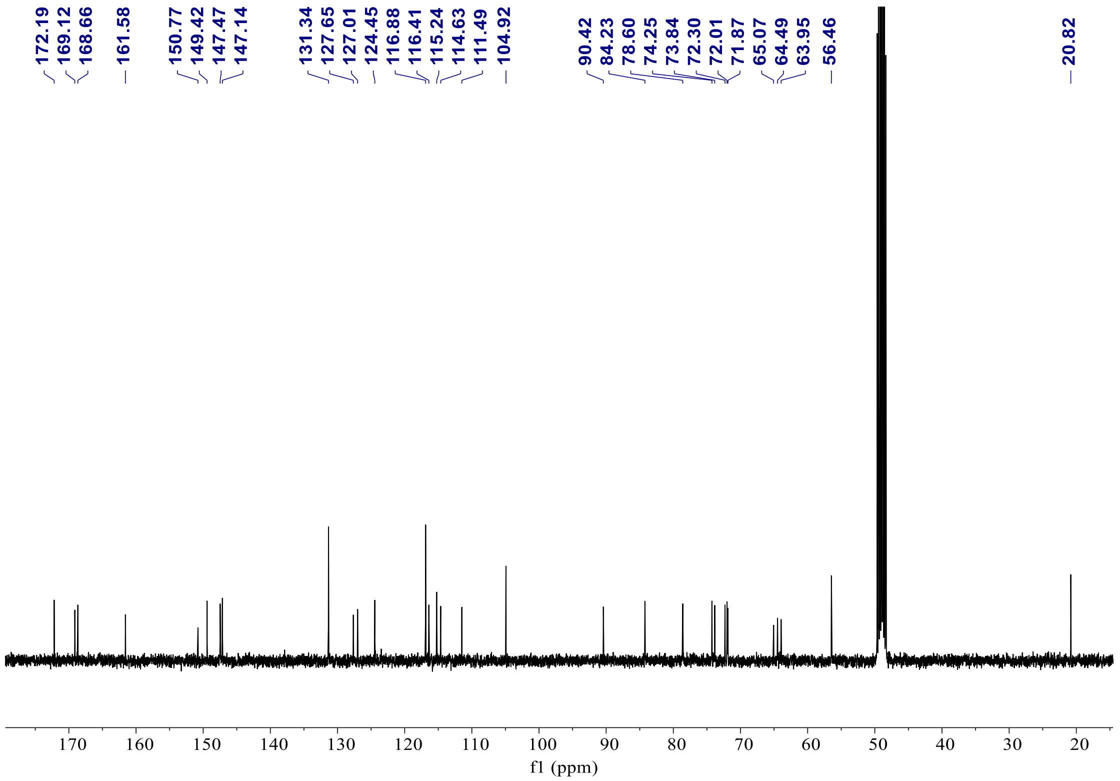

Figure S87. ^{13}C NMR Spectrum of Compound **10** in MeOH-d_4

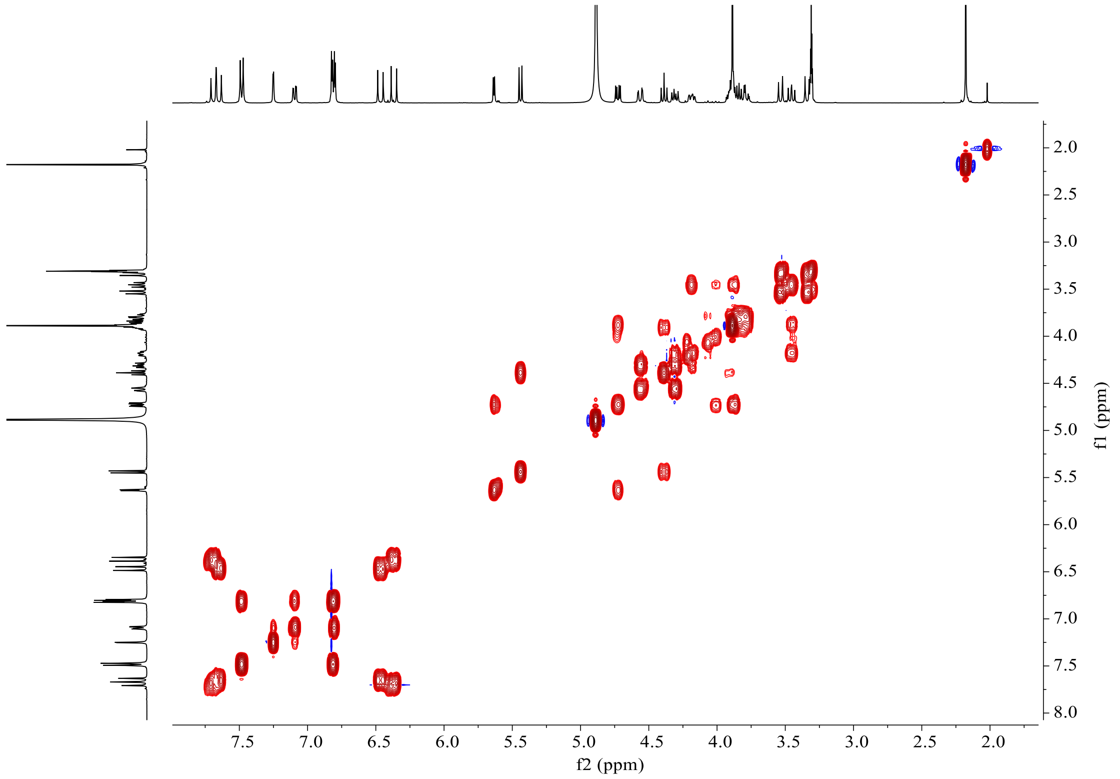

Figure S88. ^{1}H–^{1}H COSY Spectrum of Compound **10** in MeOH-d_4

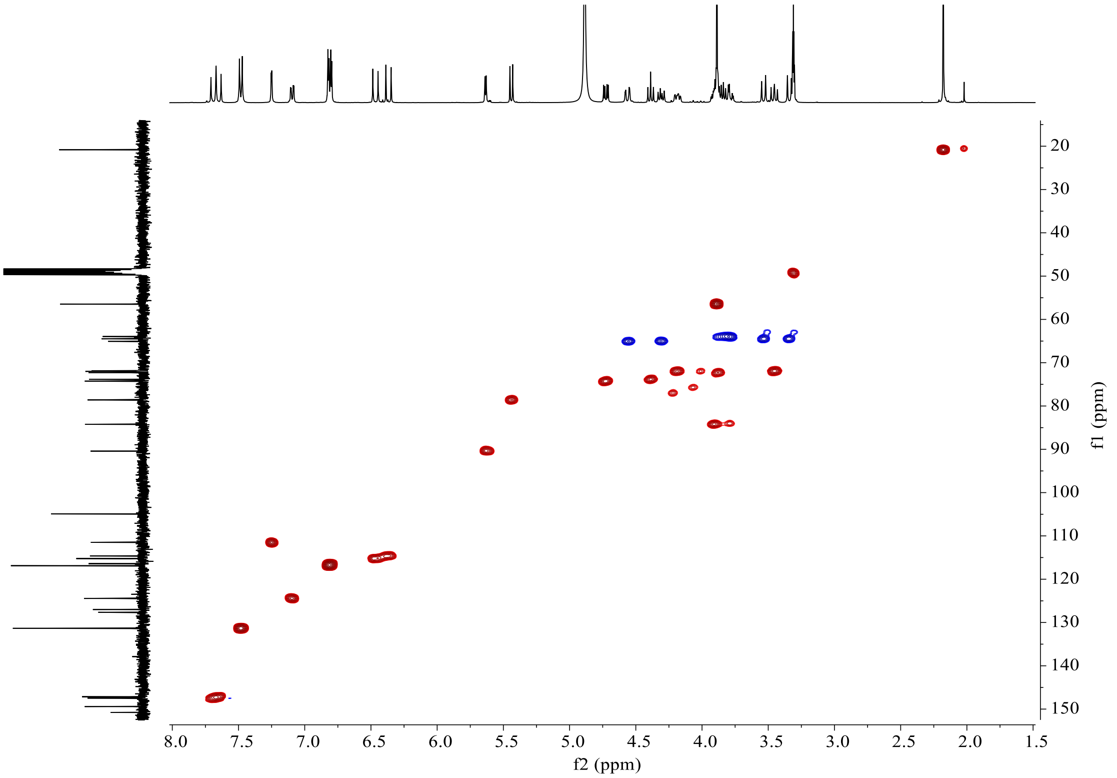

Figure S89. HSQC Spectrum of Compound **10** in MeOH-d_4

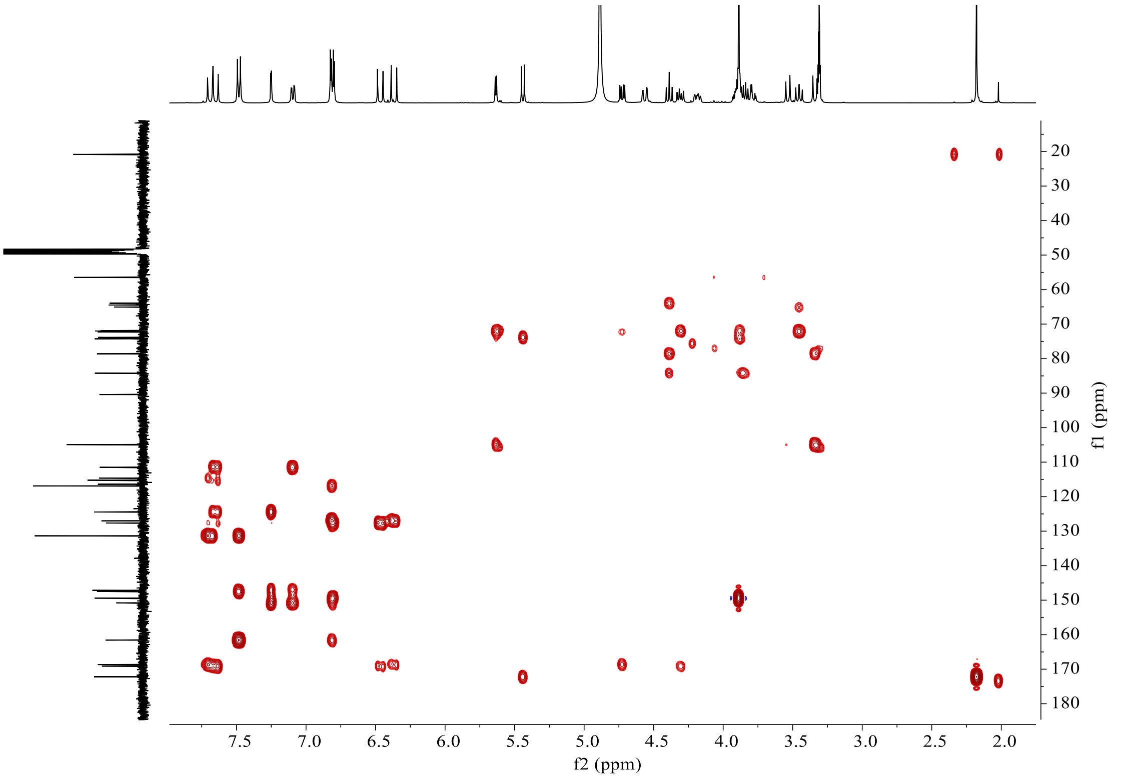

Figure S90. HMBC Spectrum of Compound **10** in MeOH-d_4

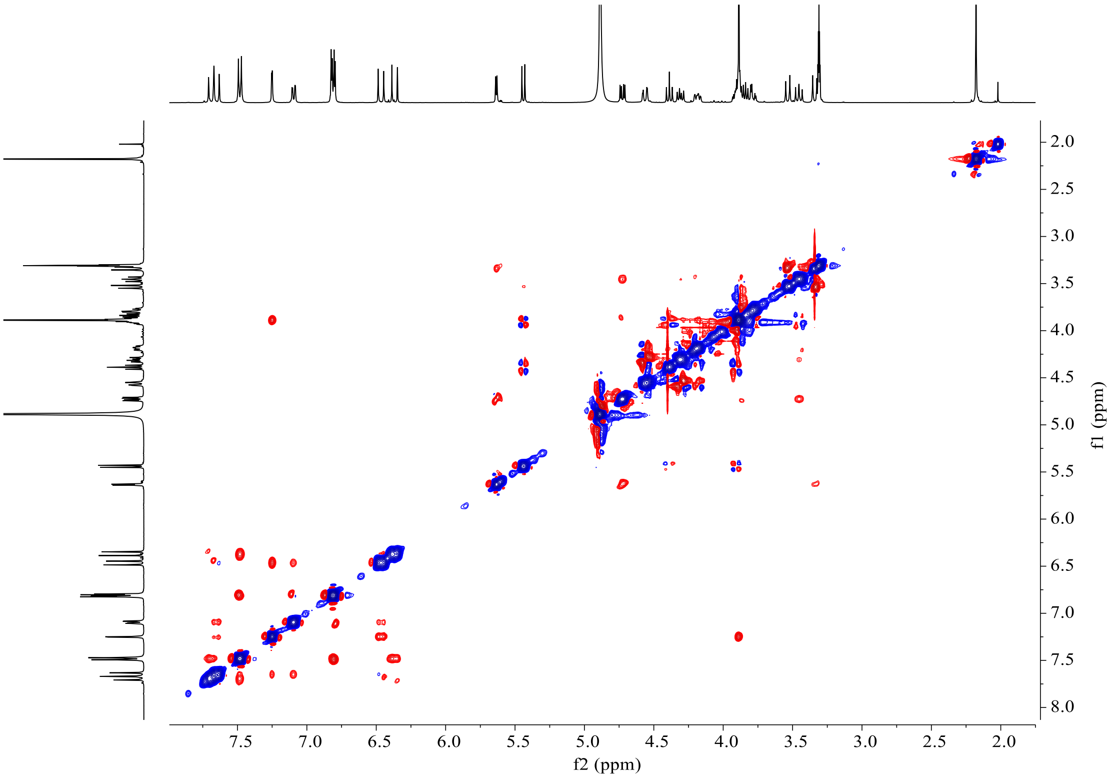

Figure S91. NOESY Spectrum of Compound **10** in MeOH-d_4

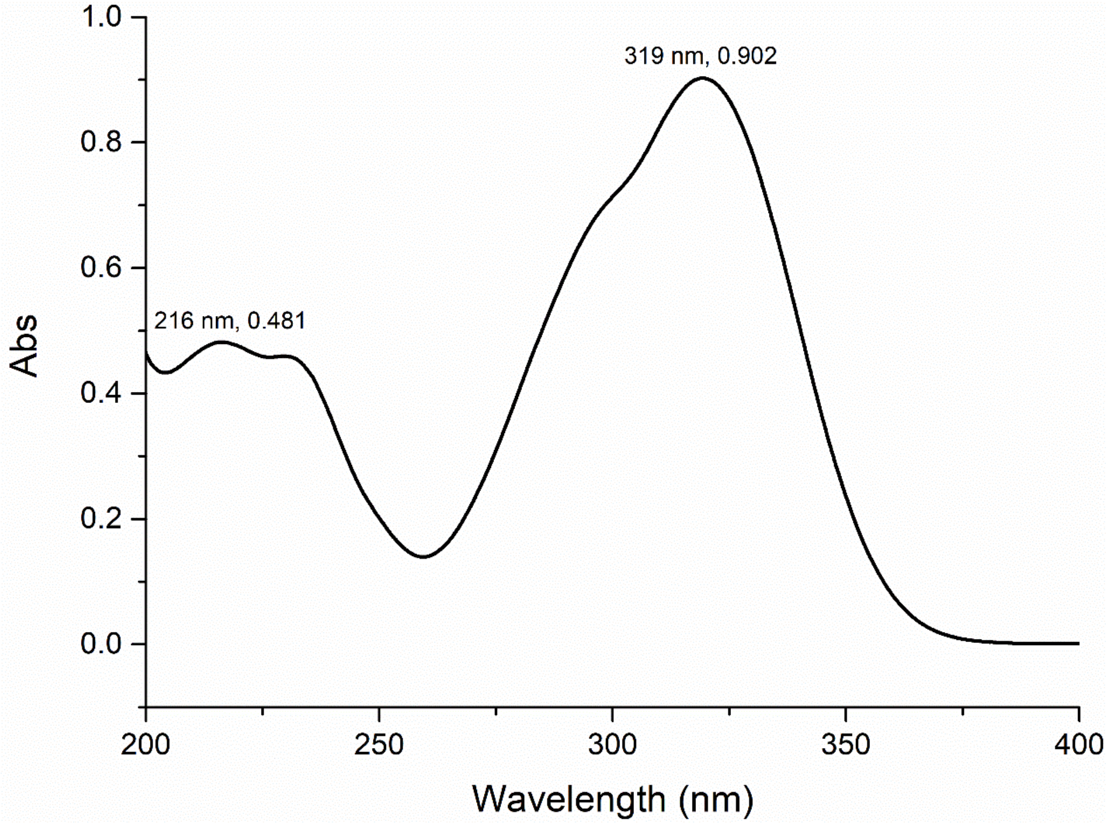

Figure S92. UV Spectrum of Compound **11**

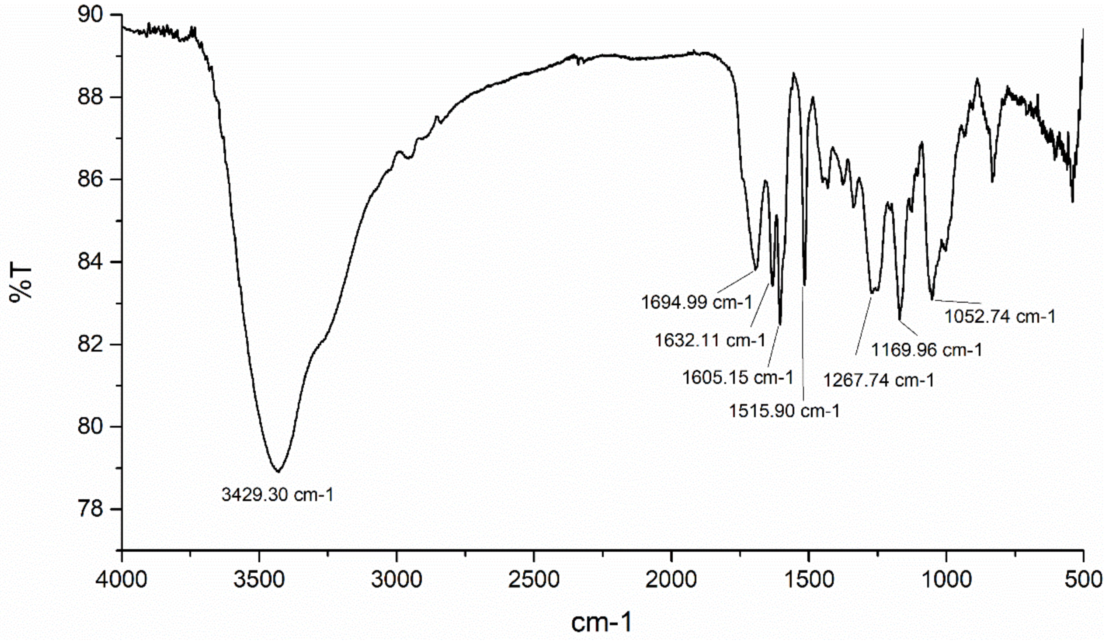

Figure S93. IR (KBr disc) Spectrum of Compound **11**

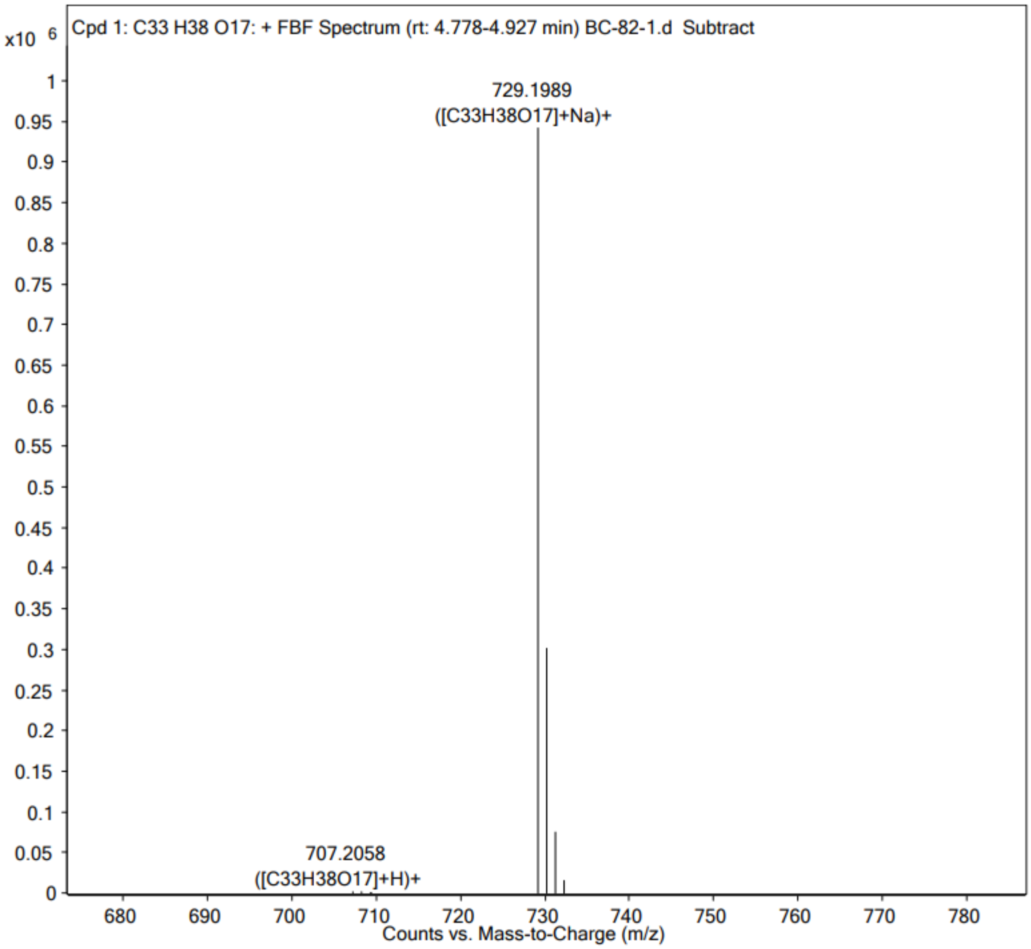

Figure S94. (+) HRESIMS Spectrum of Compound **11**

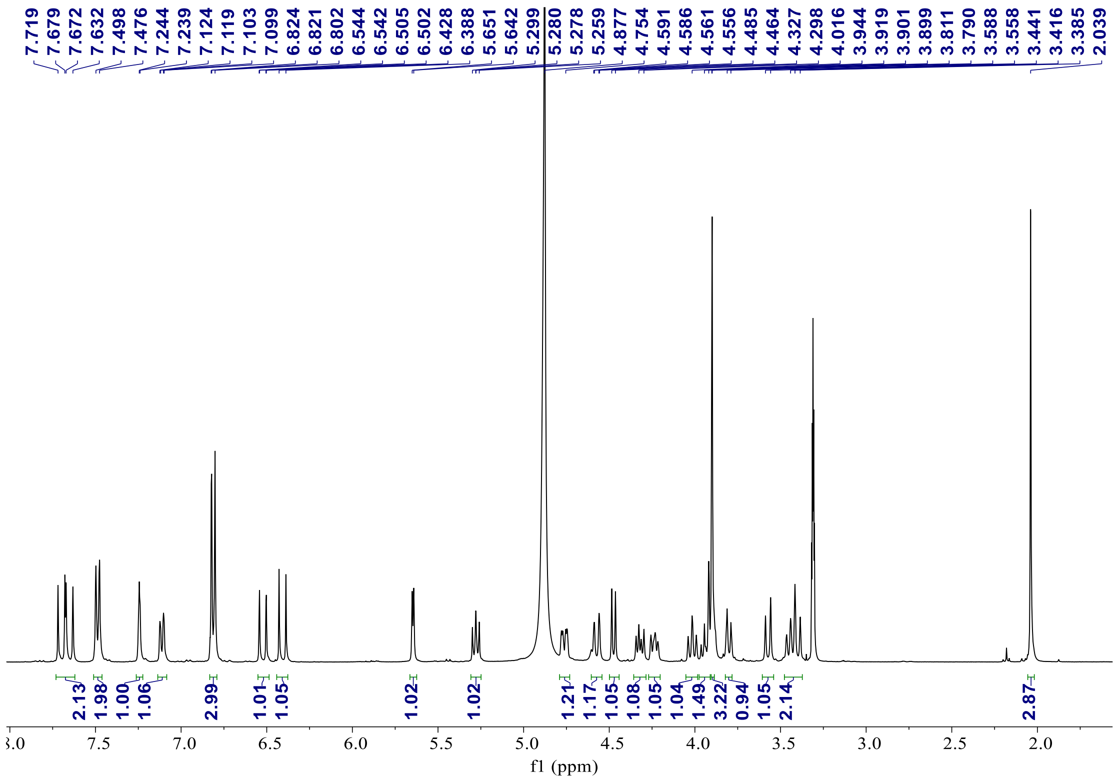

Figure S95. ^{1}H NMR Spectrum of Compound **11** in MeOH-d_4

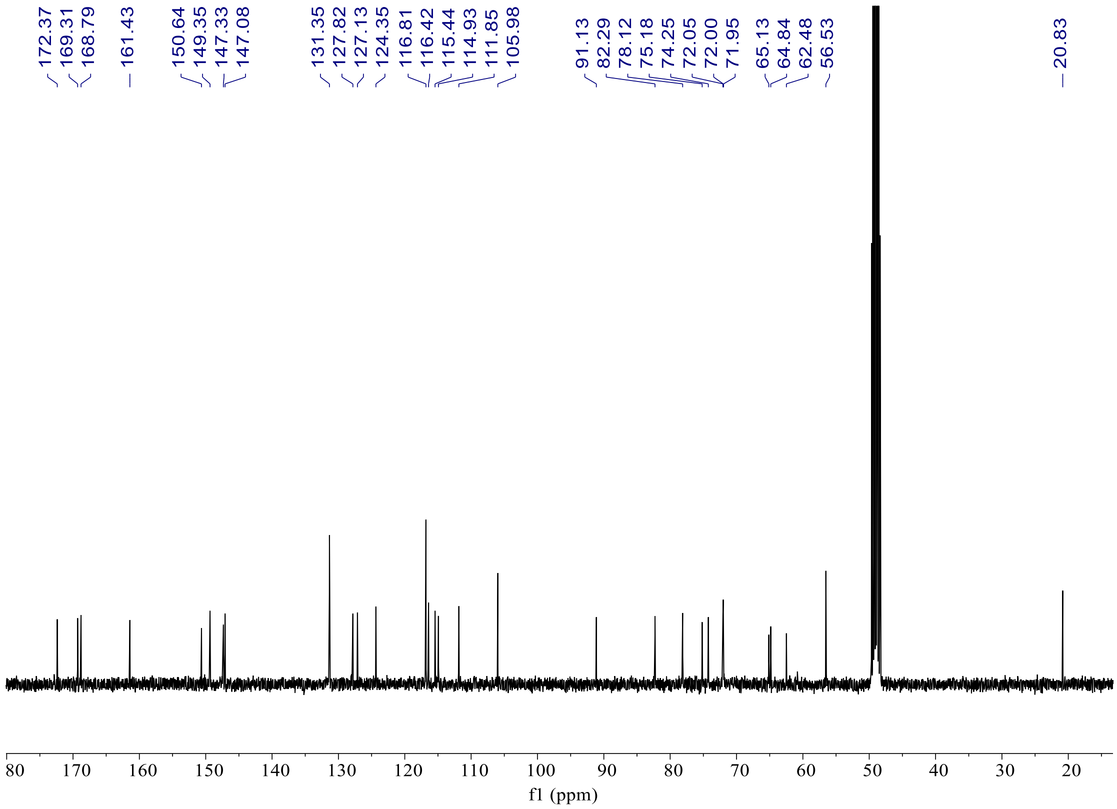

Figure S96. ^{13}C NMR Spectrum of Compound **11** in MeOH-d_4

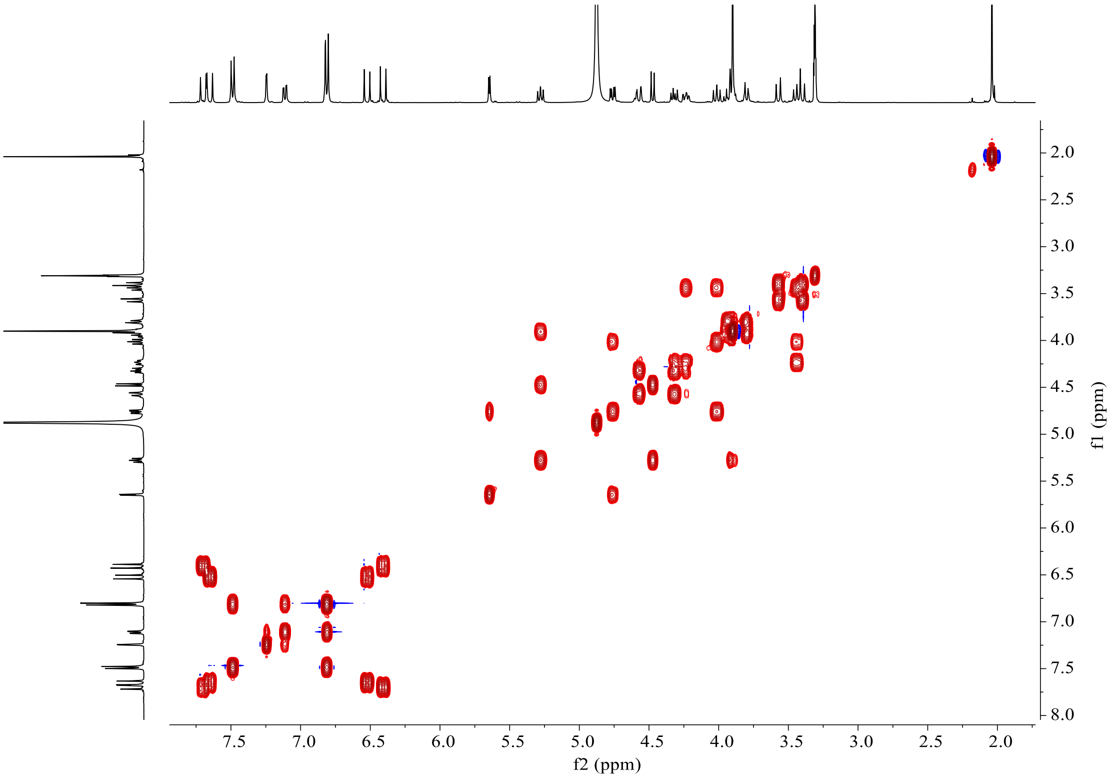

Figure S97. ^{1}H–^{1}H COSY Spectrum of Compound **11** in MeOH-d_4

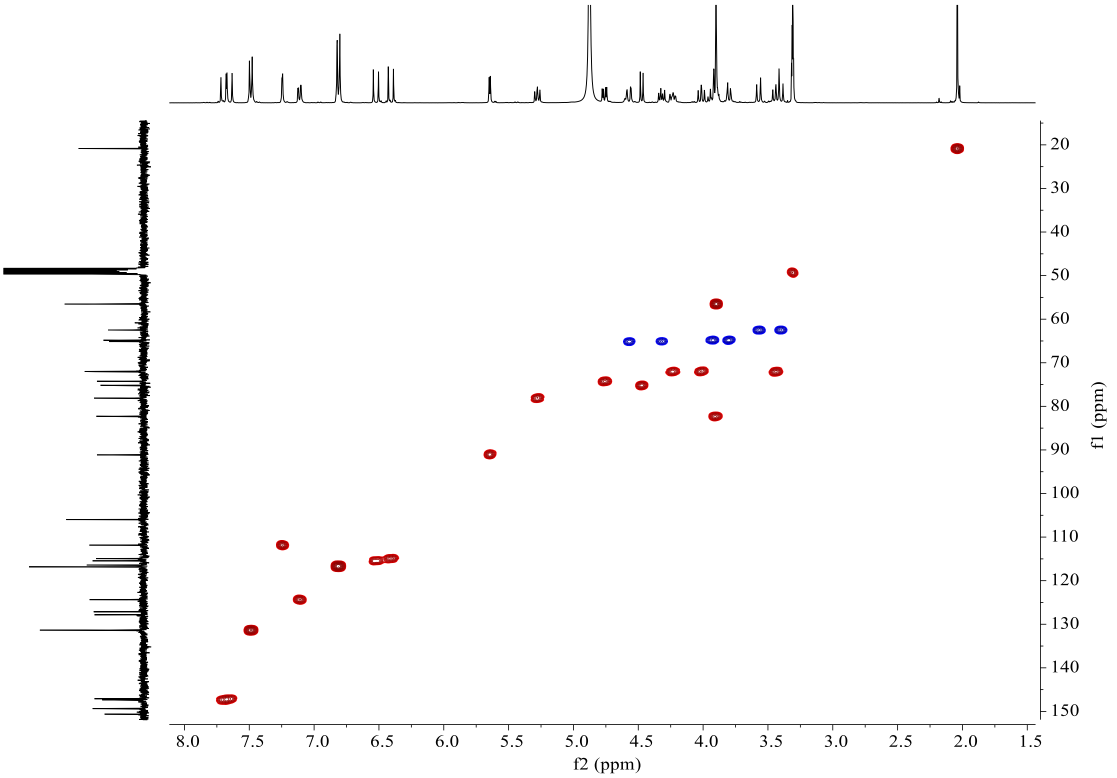

Figure S98. HSQC Spectrum of Compound **11** in MeOH-d_4

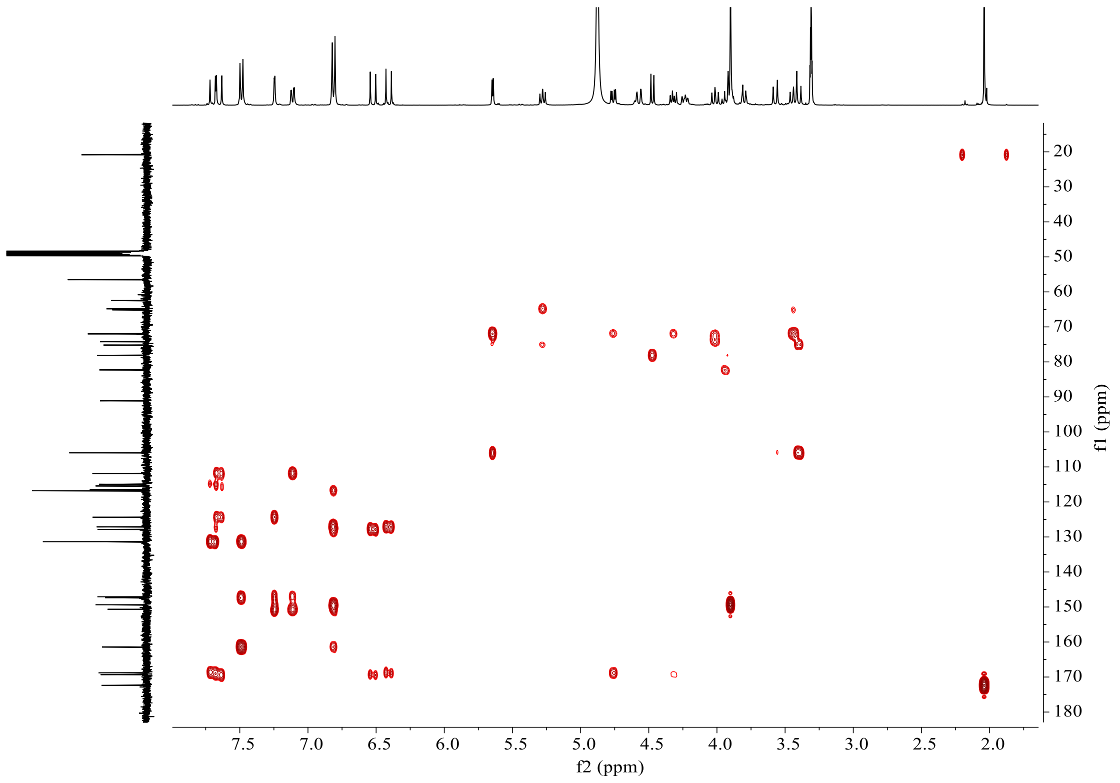

Figure S99. HMBC Spectrum of Compound **11** in MeOH-d_4

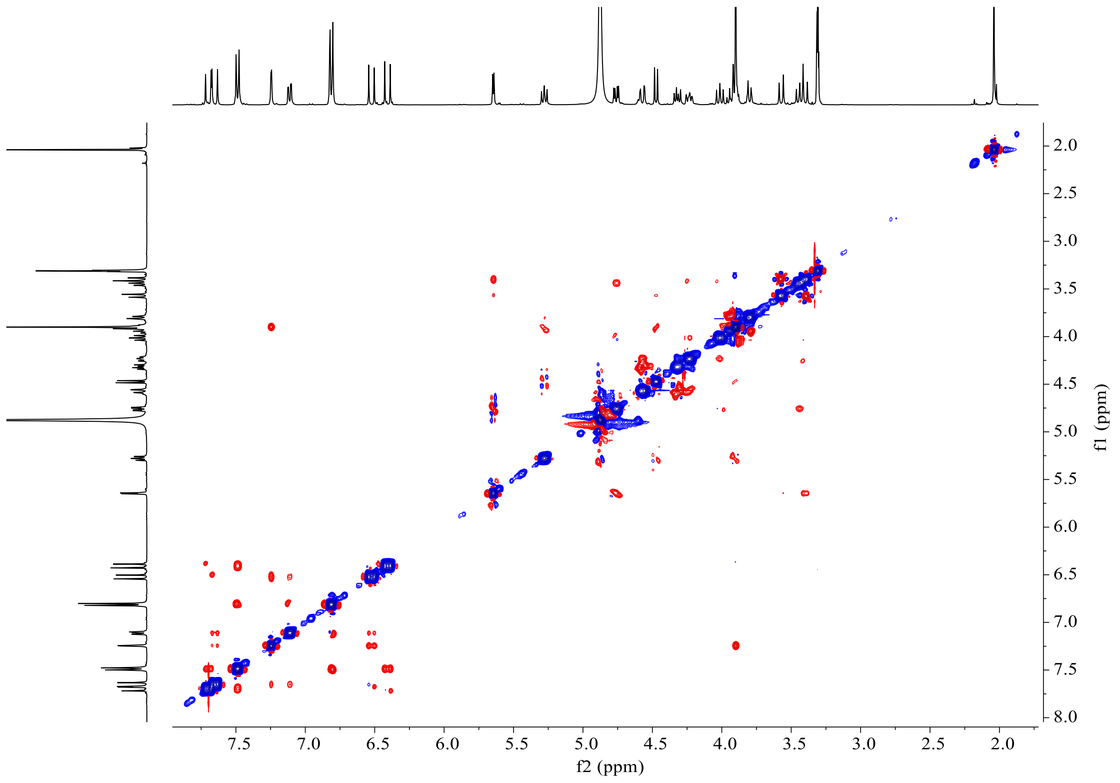

Figure S100. NOESY Spectrum of Compound **11** in MeOH-d_4

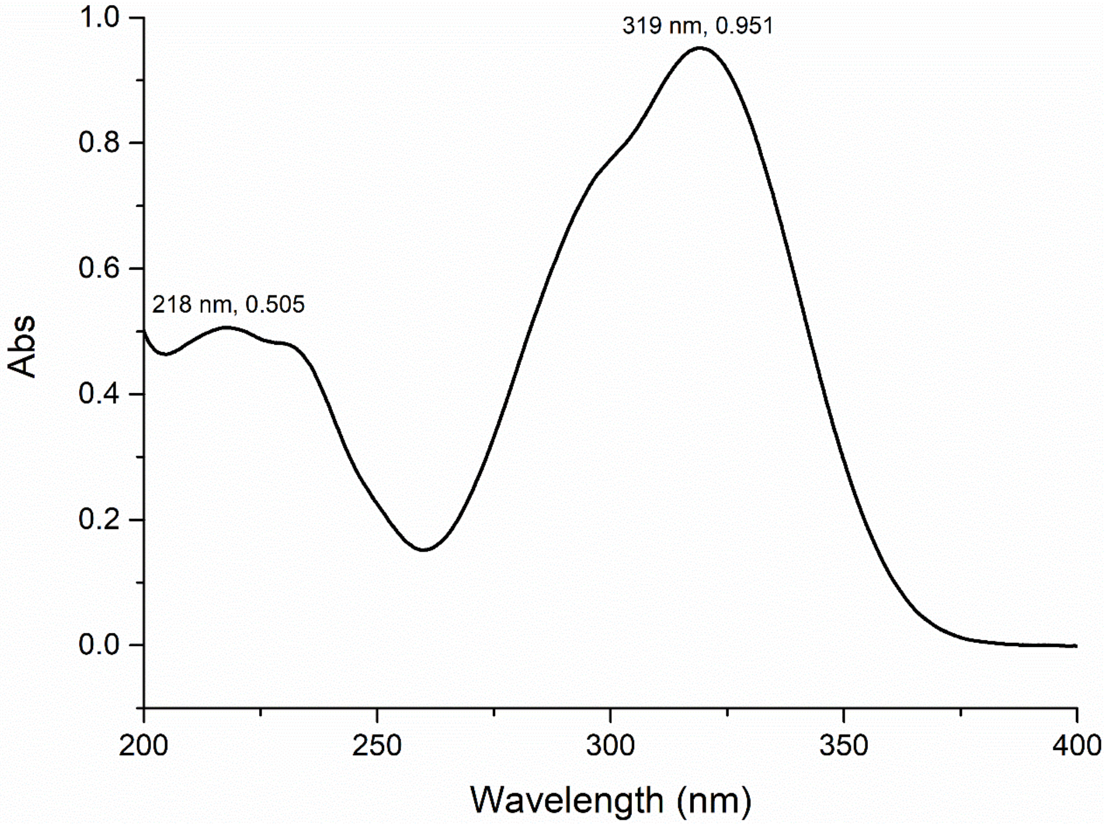

Figure S101. UV Spectrum of Compound **12**

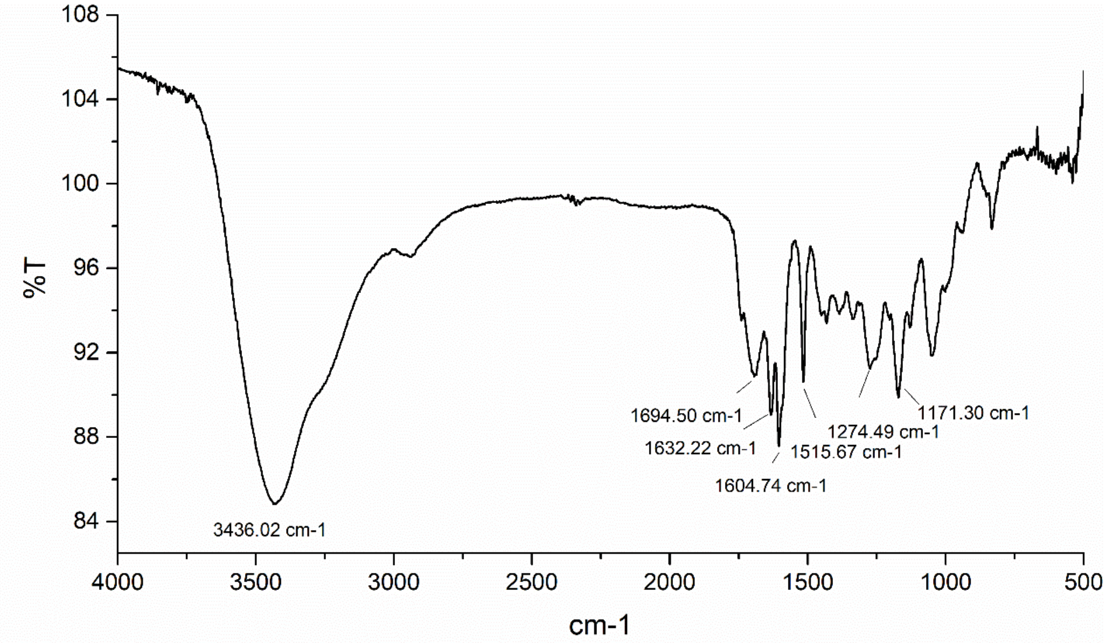

Figure S102. IR (KBr disc) Spectrum of Compound **12**

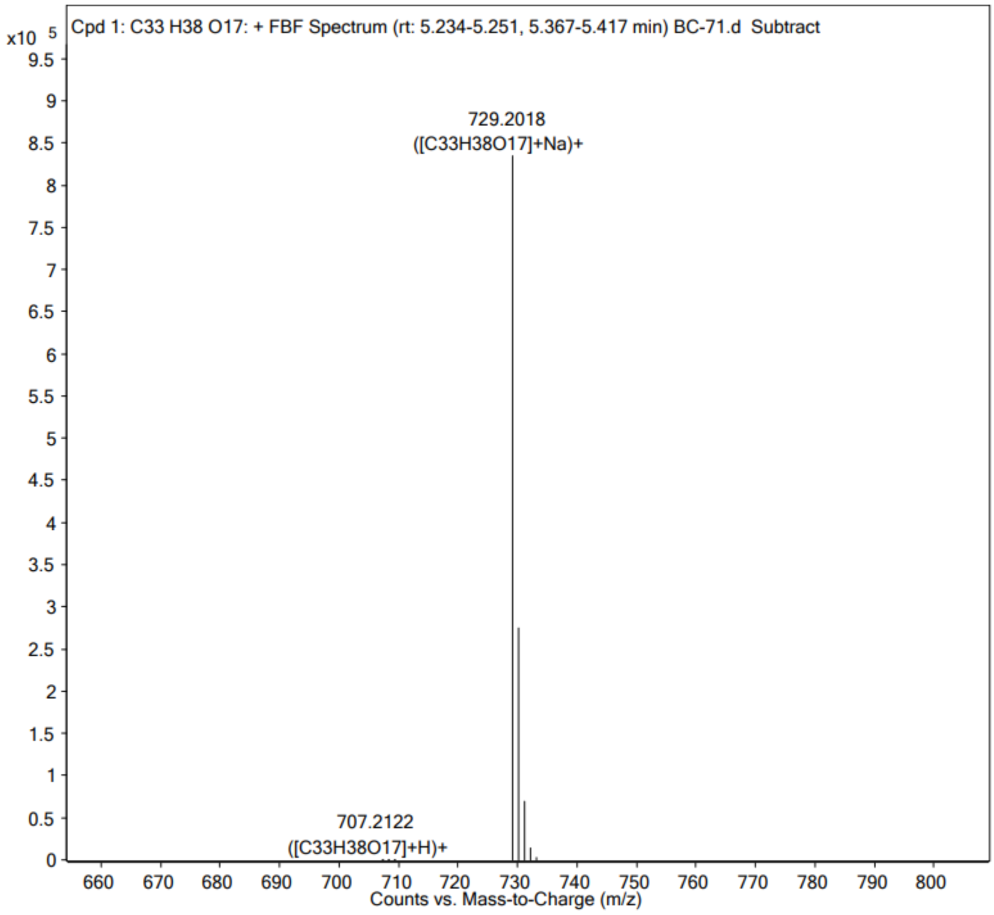

Figure S103. (+) HRESIMS Spectrum of Compound **12**

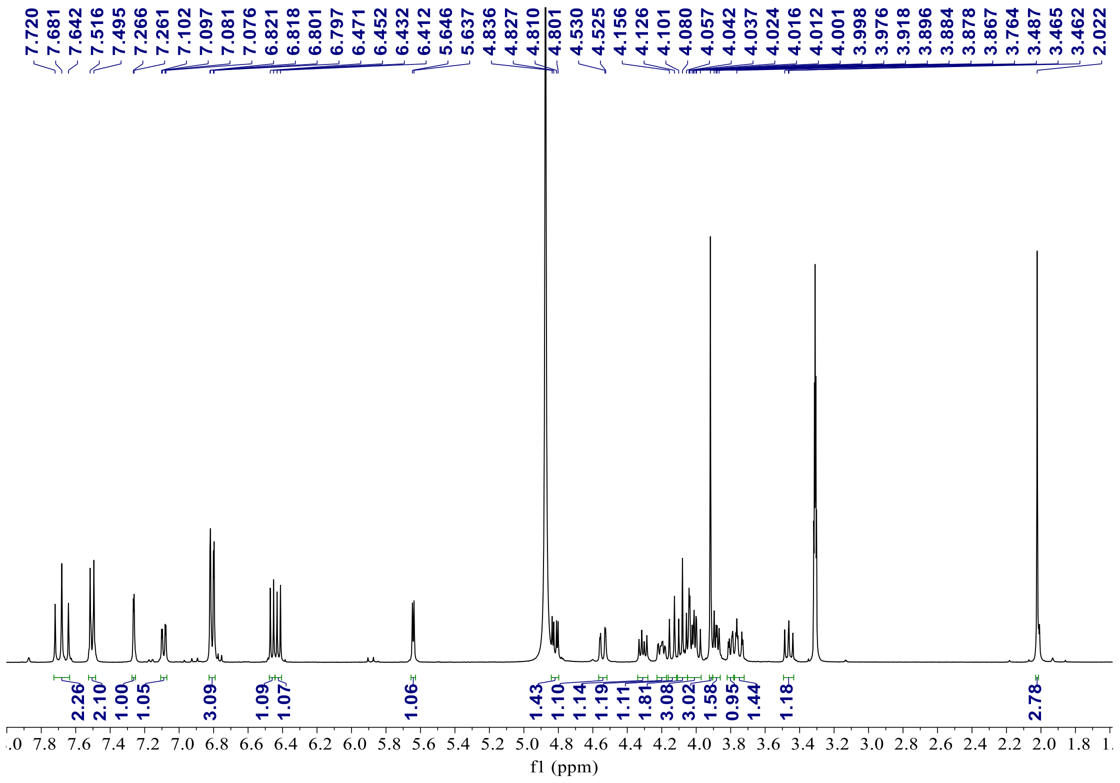

Figure S104. ^{1}H NMR Spectrum of Compound **12** in MeOH-d_4

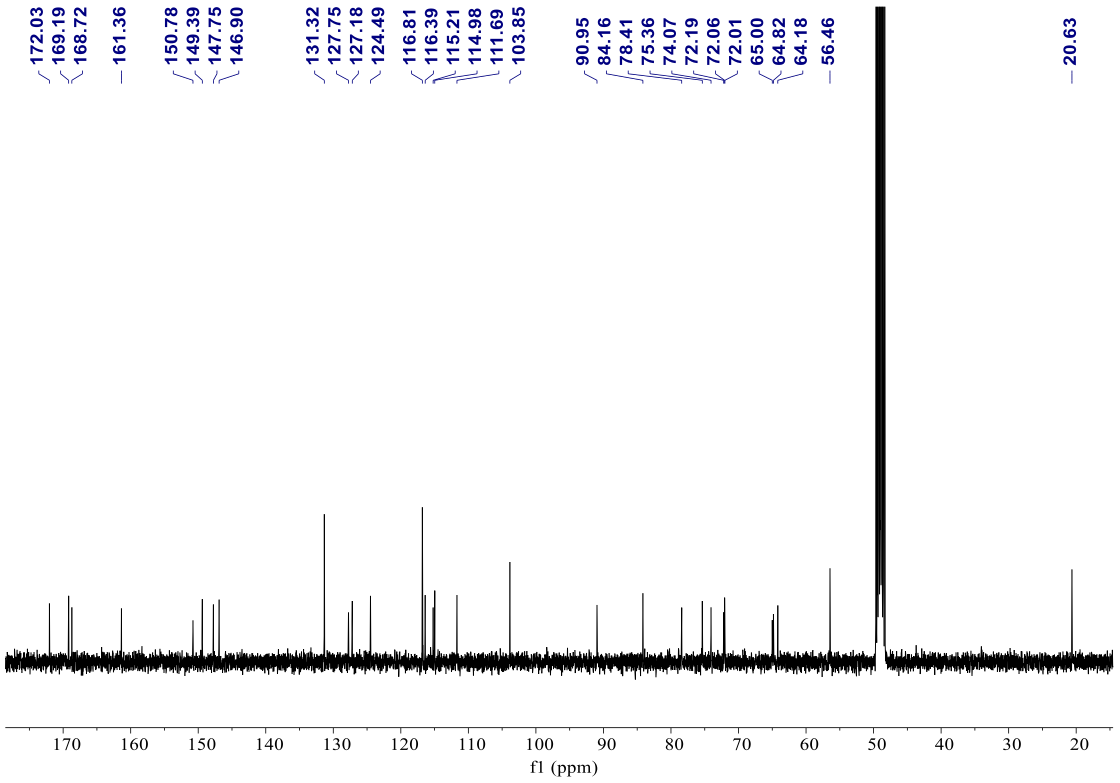

Figure S105. ^{13}C NMR Spectrum of Compound **12** in MeOH-d_4

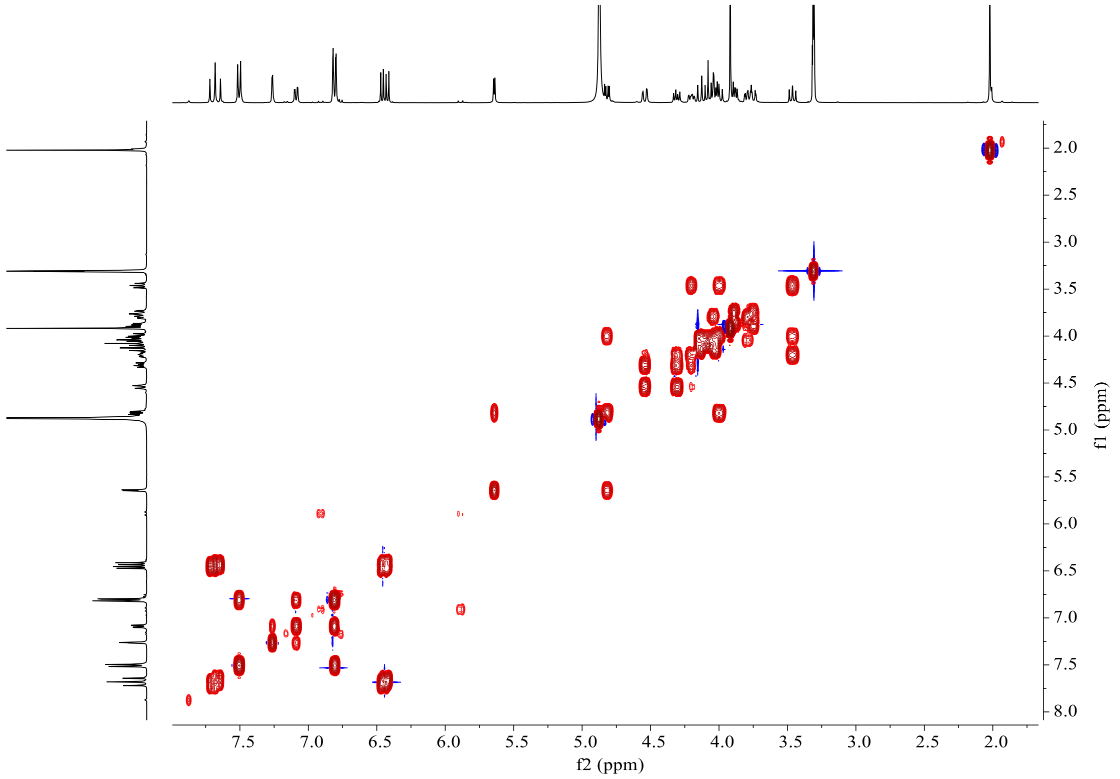

Figure S106. ^{1}H–^{1}H COSY Spectrum of Compound **12** in MeOH-d_4

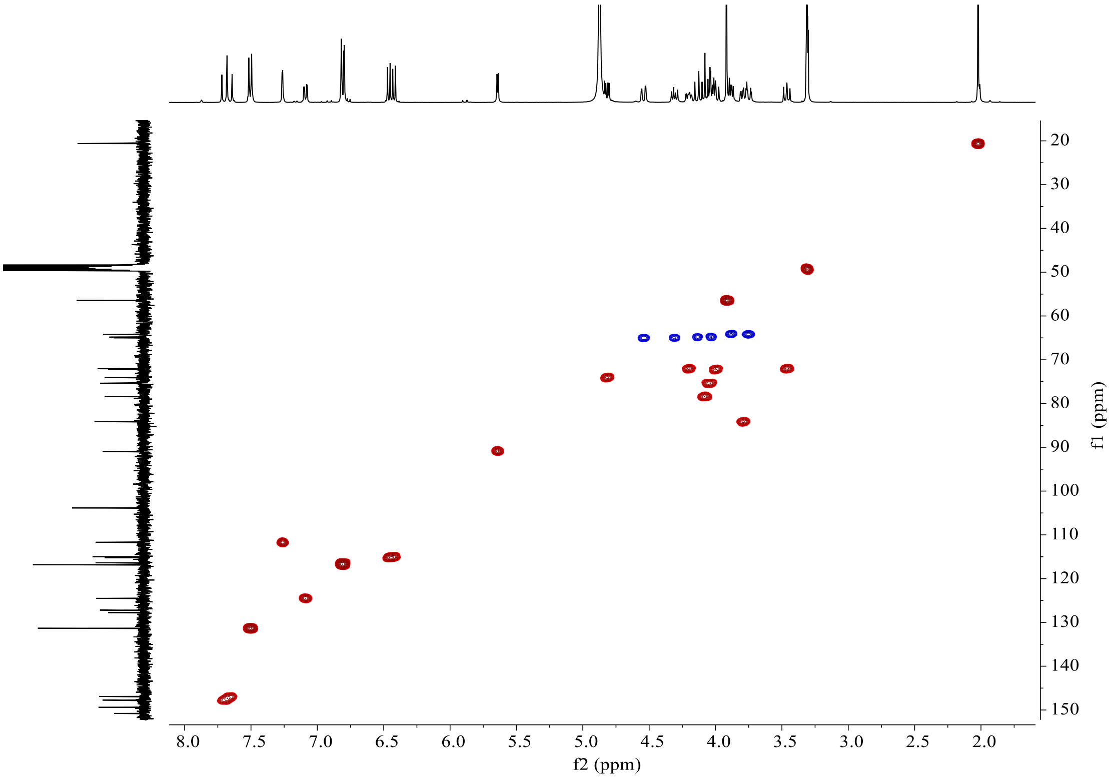

Figure S107. HSQC Spectrum of Compound **12** in MeOH-d_4

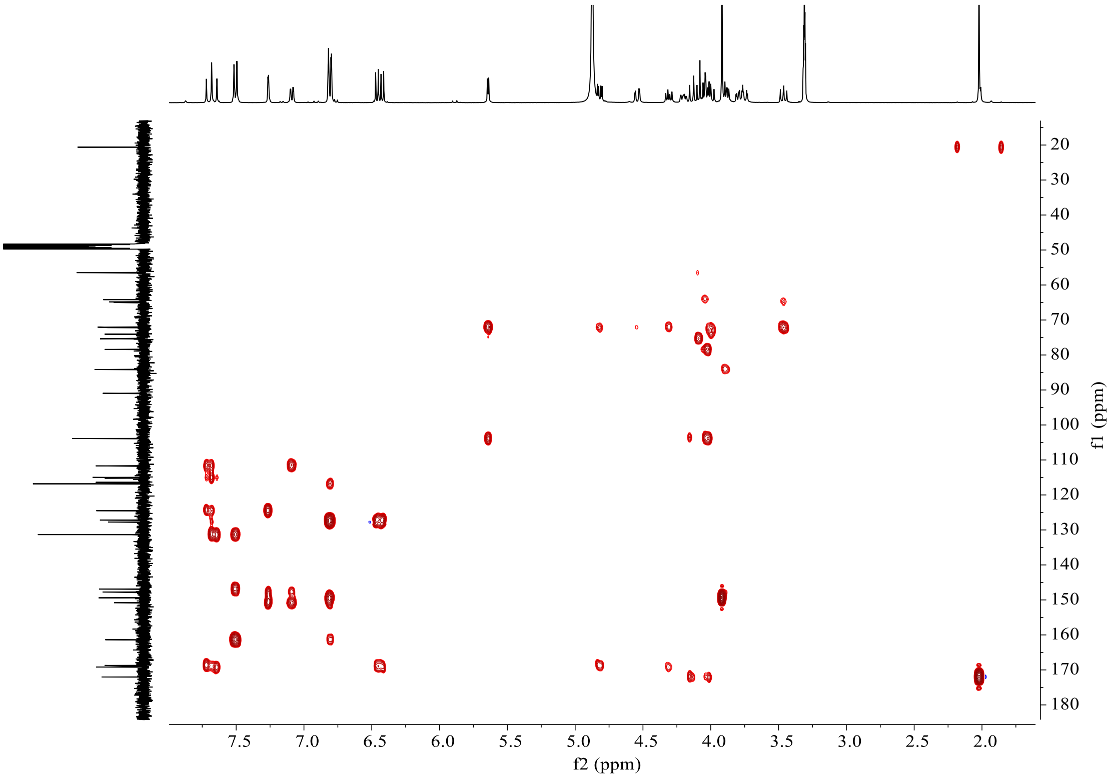

Figure S108. HMBC Spectrum of Compound **12** in MeOH-d_4

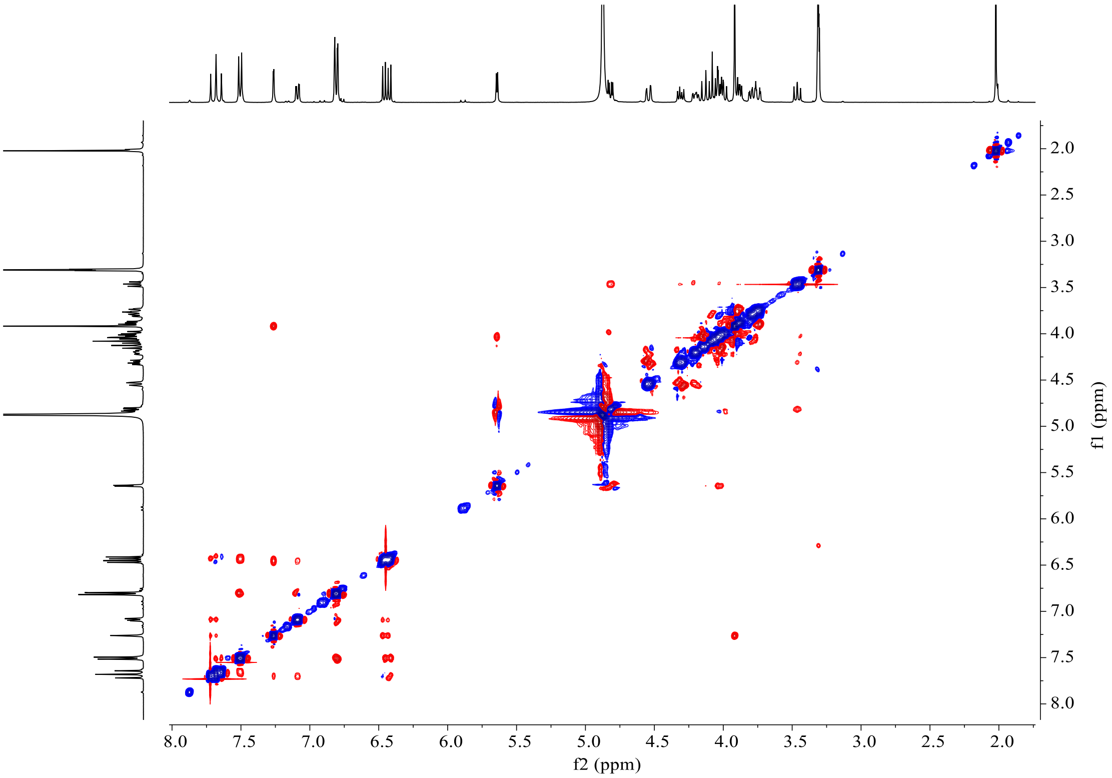

Figure S109. NOESY Spectrum of Compound **12** in MeOH-d_4

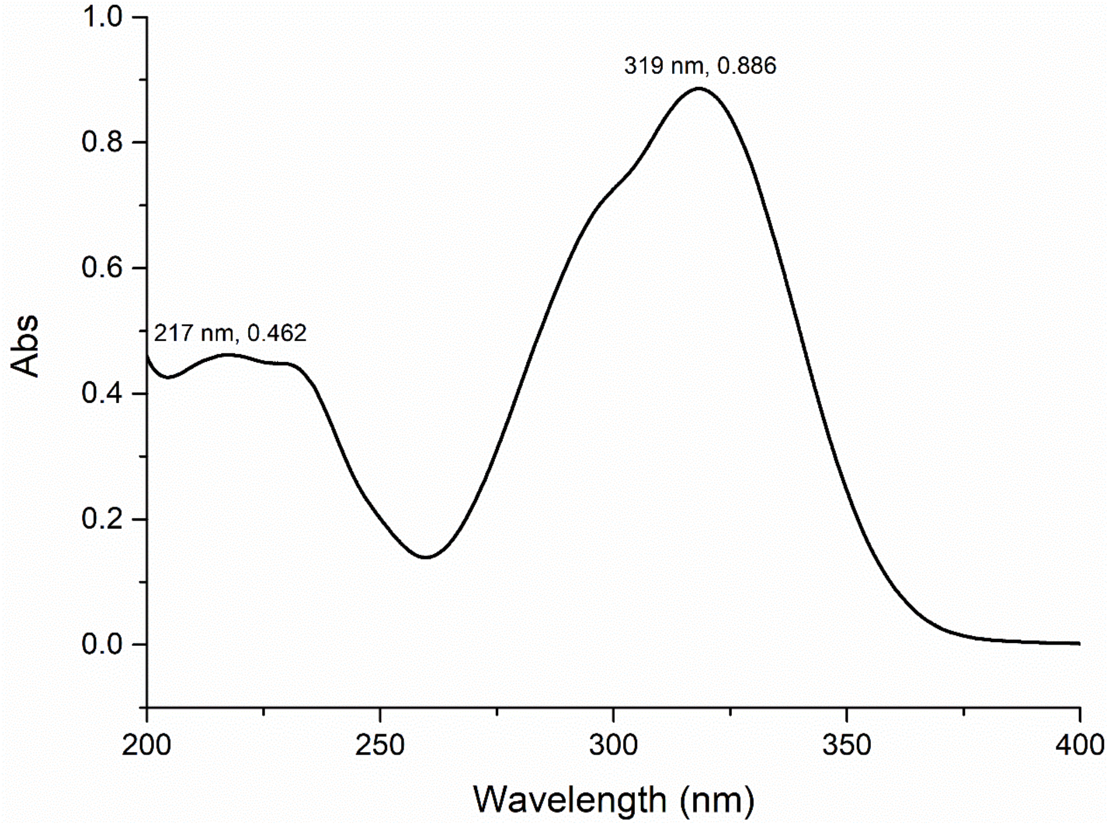

Figure S110. UV Spectrum of Compound **13**

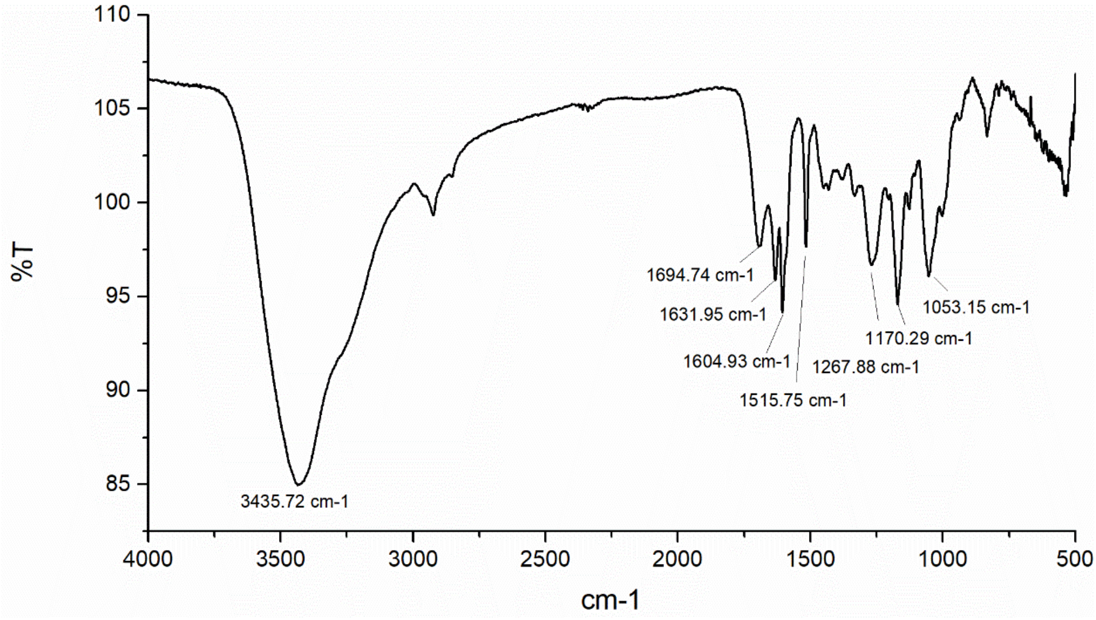

Figure S111. IR (KBr disc) Spectrum of Compound **13**

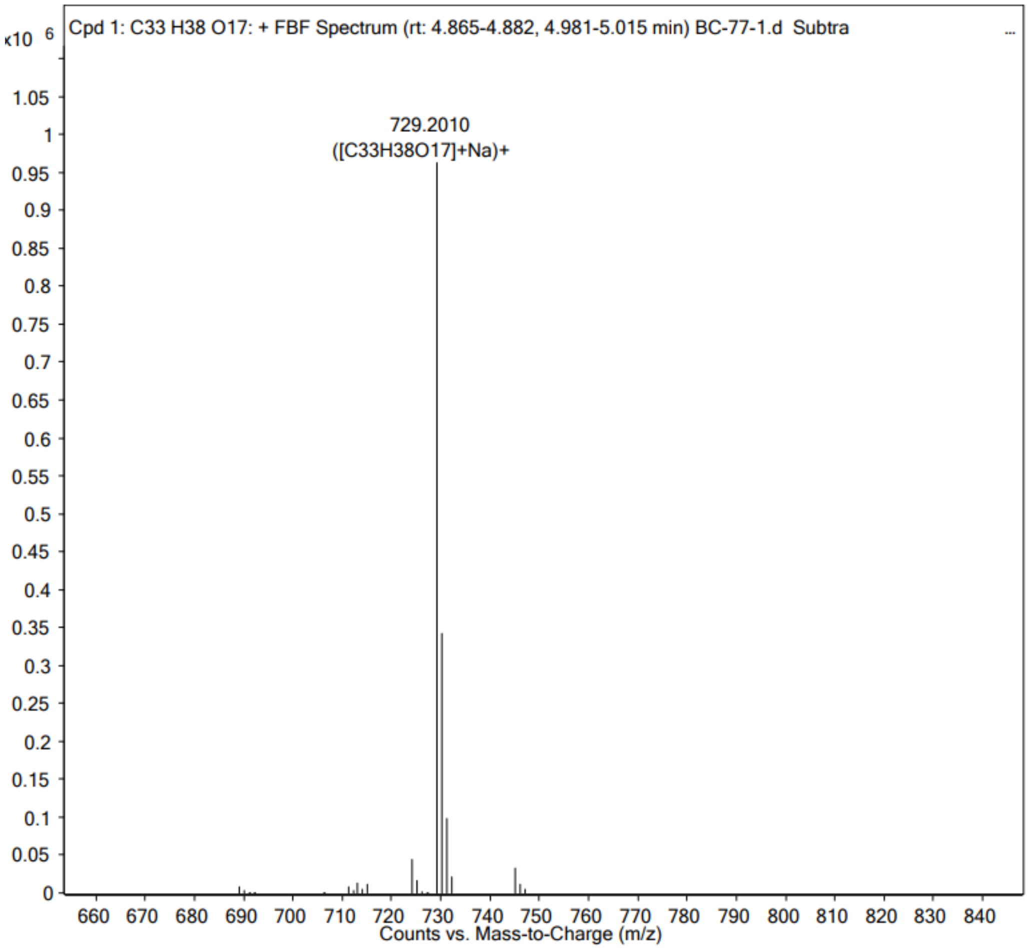

Figure S112. (+) HRESIMS Spectrum of Compound **13**

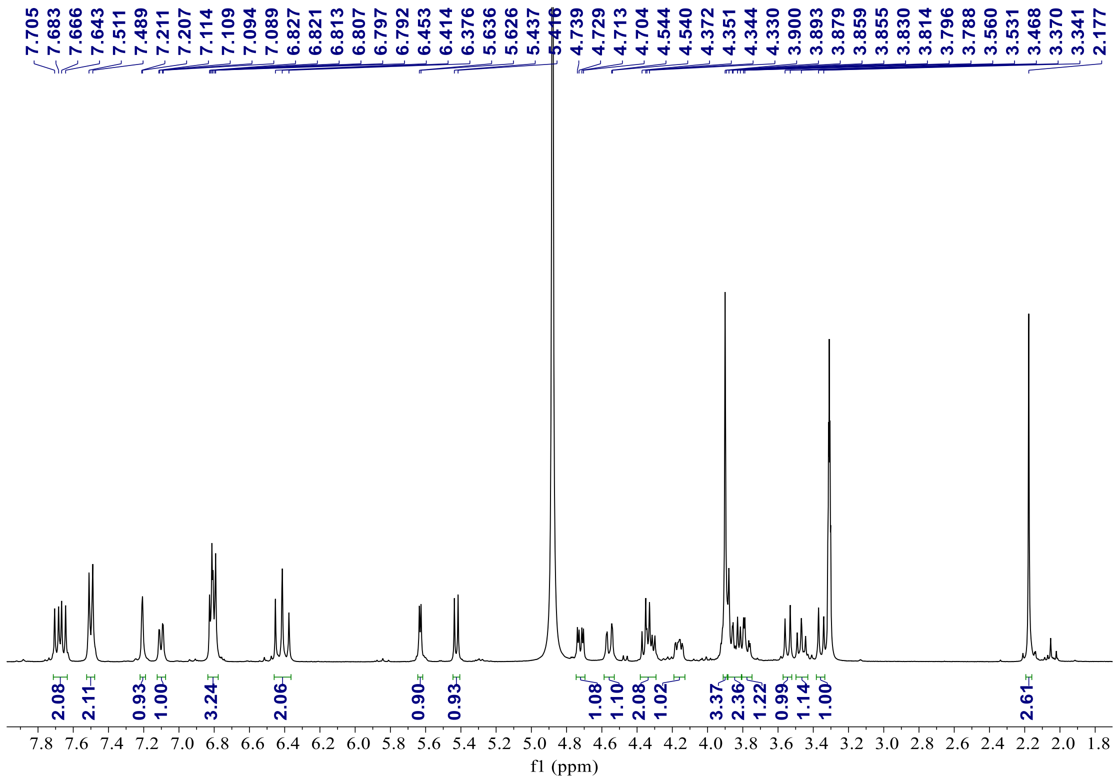

Figure S113. ^{1}H NMR Spectrum of Compound **13** in MeOH-d_4

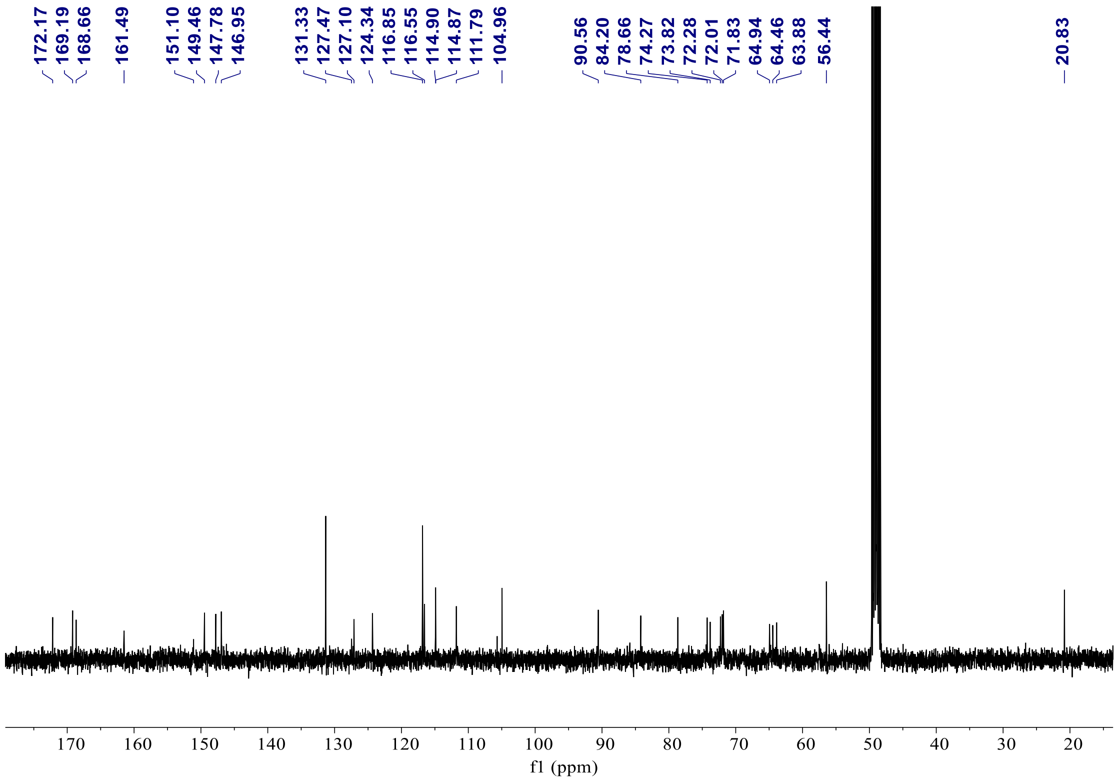

Figure S114. ^{13}C NMR Spectrum of Compound **13** in MeOH-d_4

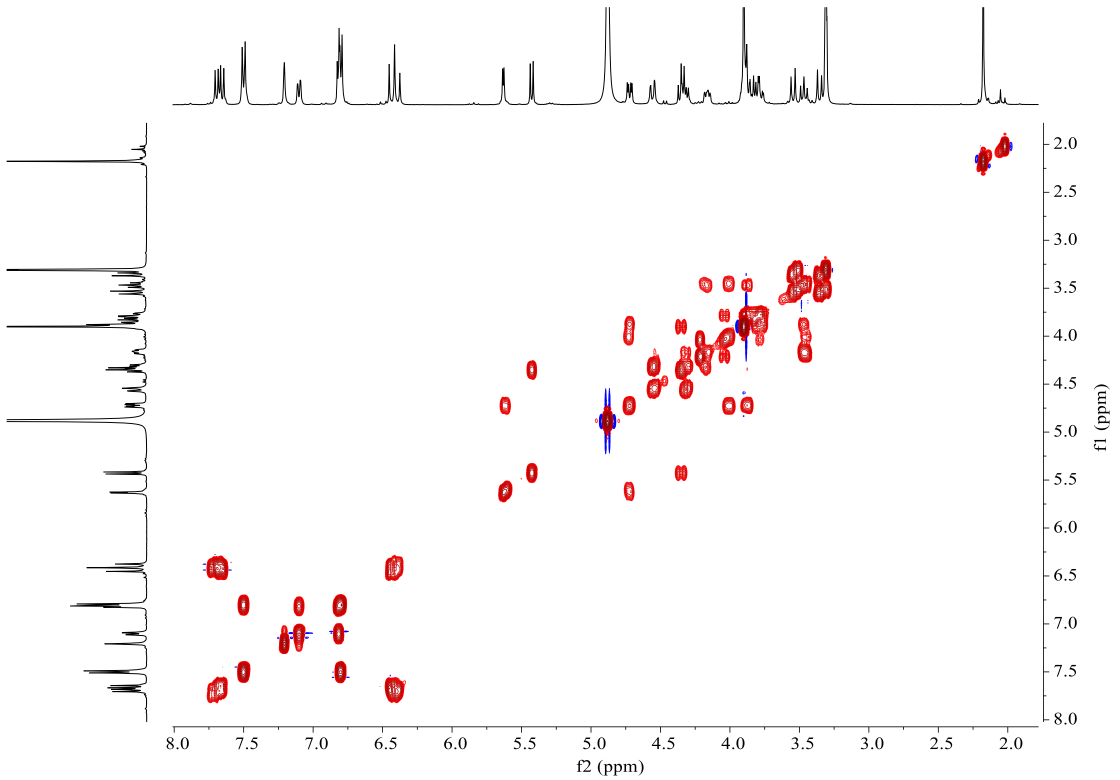

Figure S115. ^{1}H–^{1}H COSY Spectrum of Compound **13** in MeOH-d_4

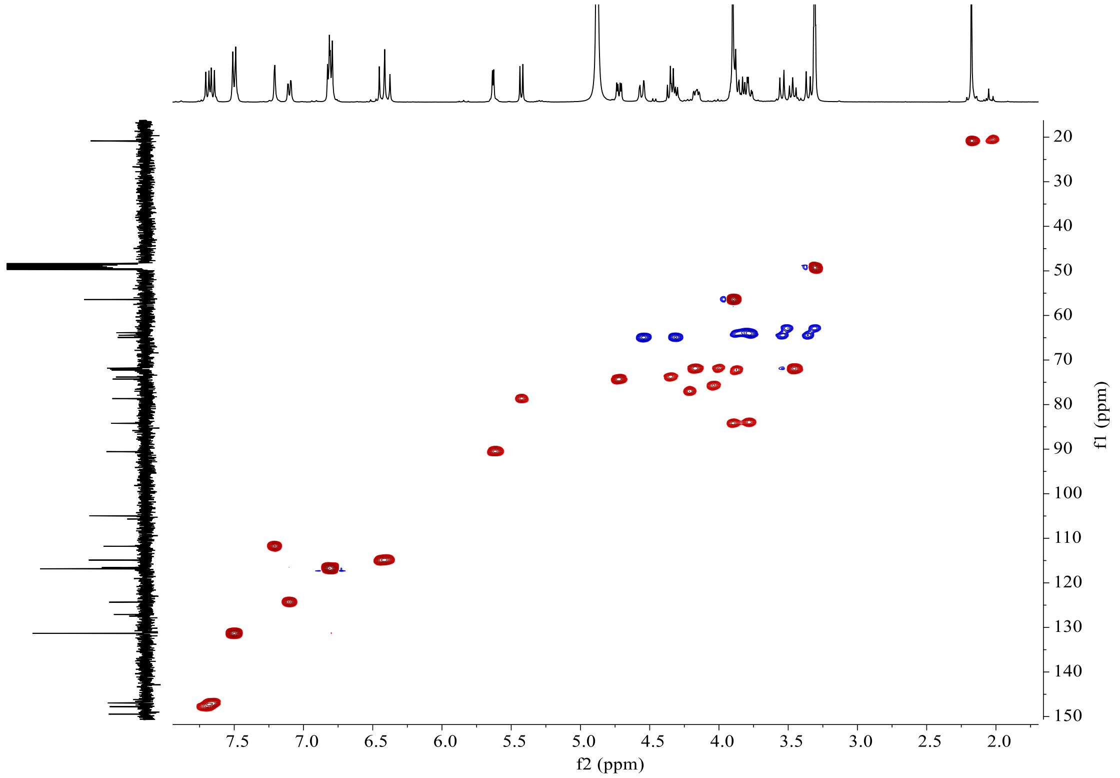

Figure S116. HSQC Spectrum of Compound **13** in MeOH-d_4

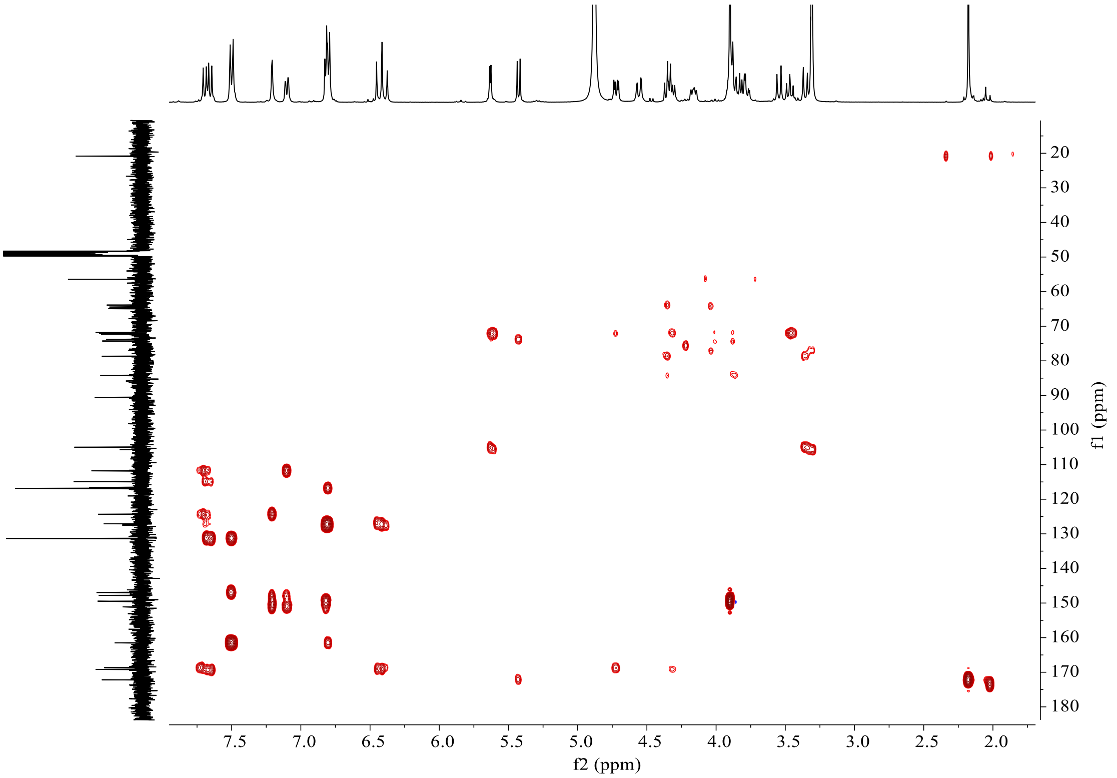

Figure S117. HMBC Spectrum of Compound **13** in MeOH-d_4

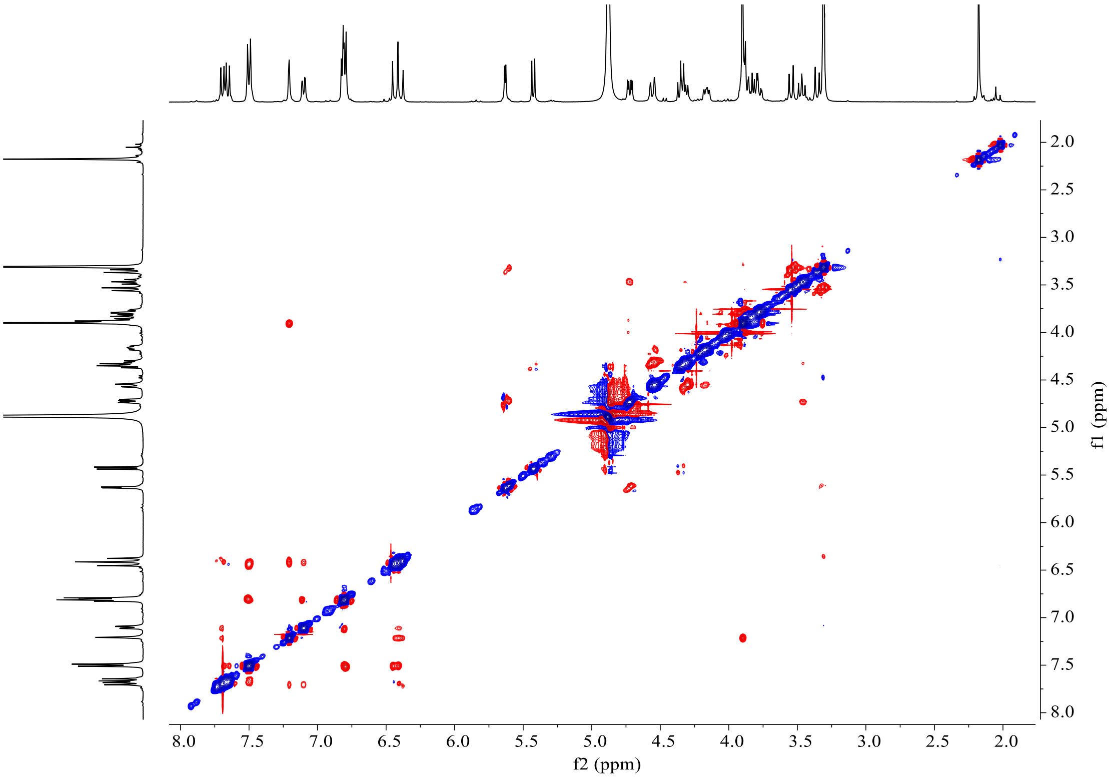

Figure S118. NOESY Spectrum of Compound **13** in MeOH-d_4

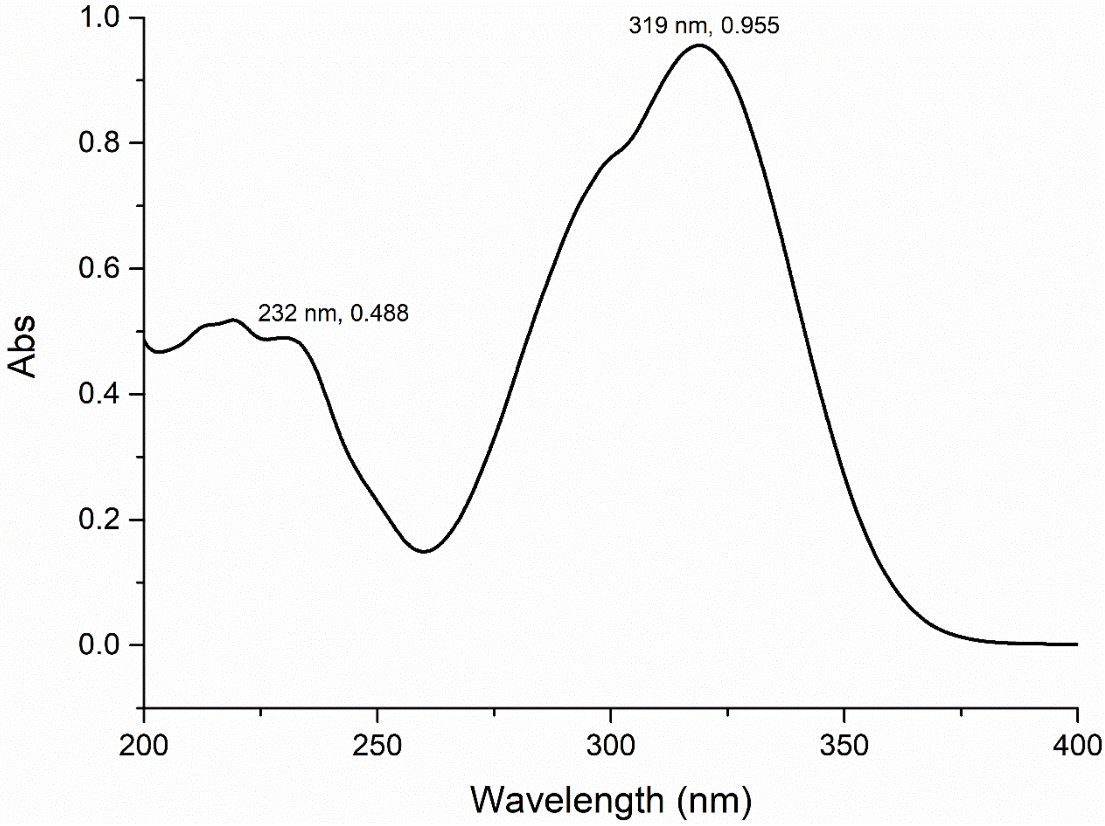

Figure S119. UV Spectrum of Compound **14**

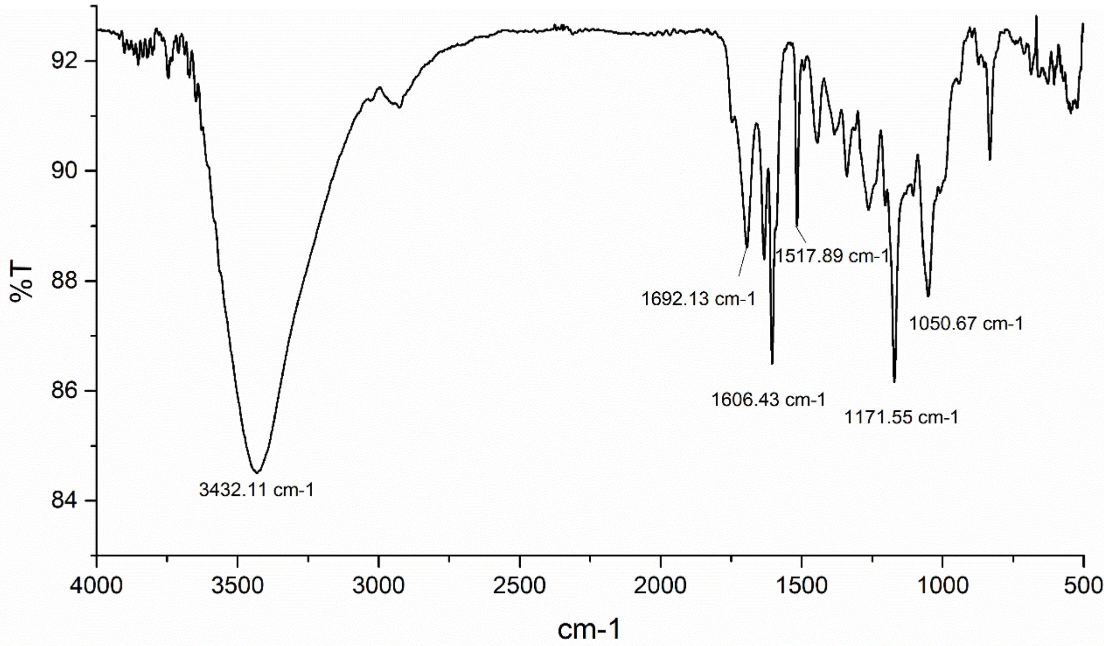

Figure S120. IR (KBr disc) Spectrum of Compound **14**

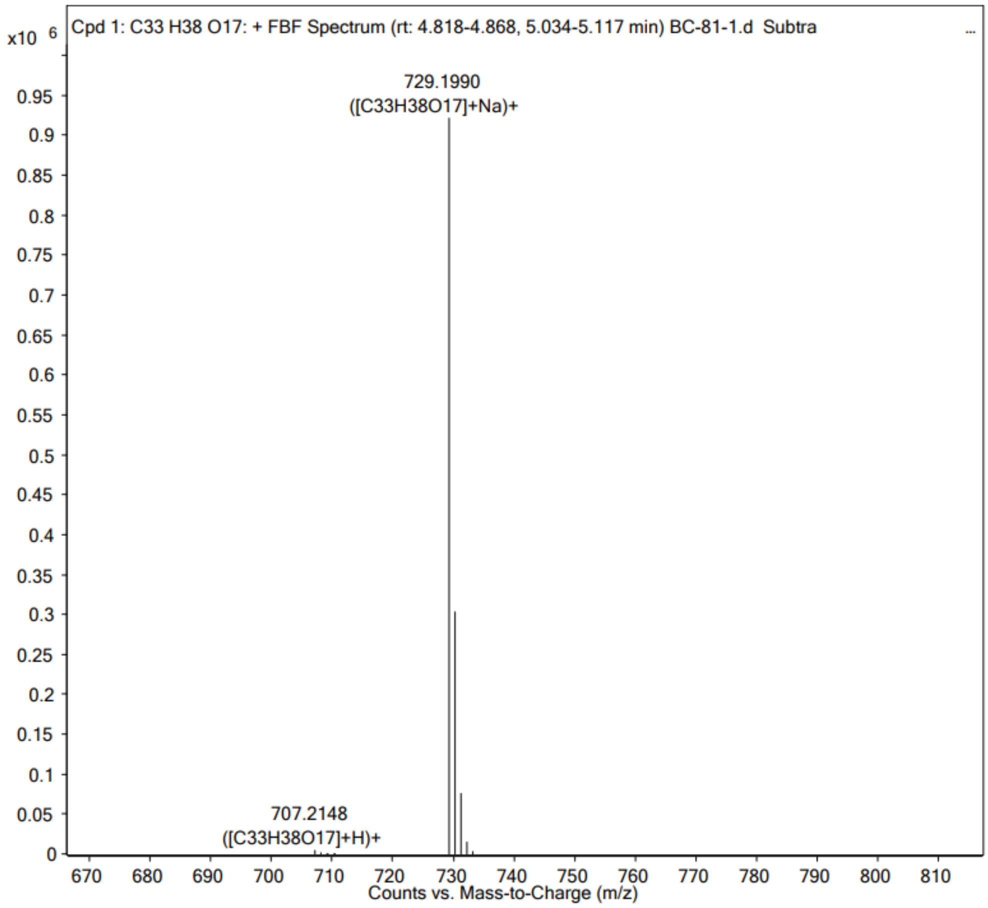

Figure S121. (+) HRESIMS Spectrum of Compound **14**

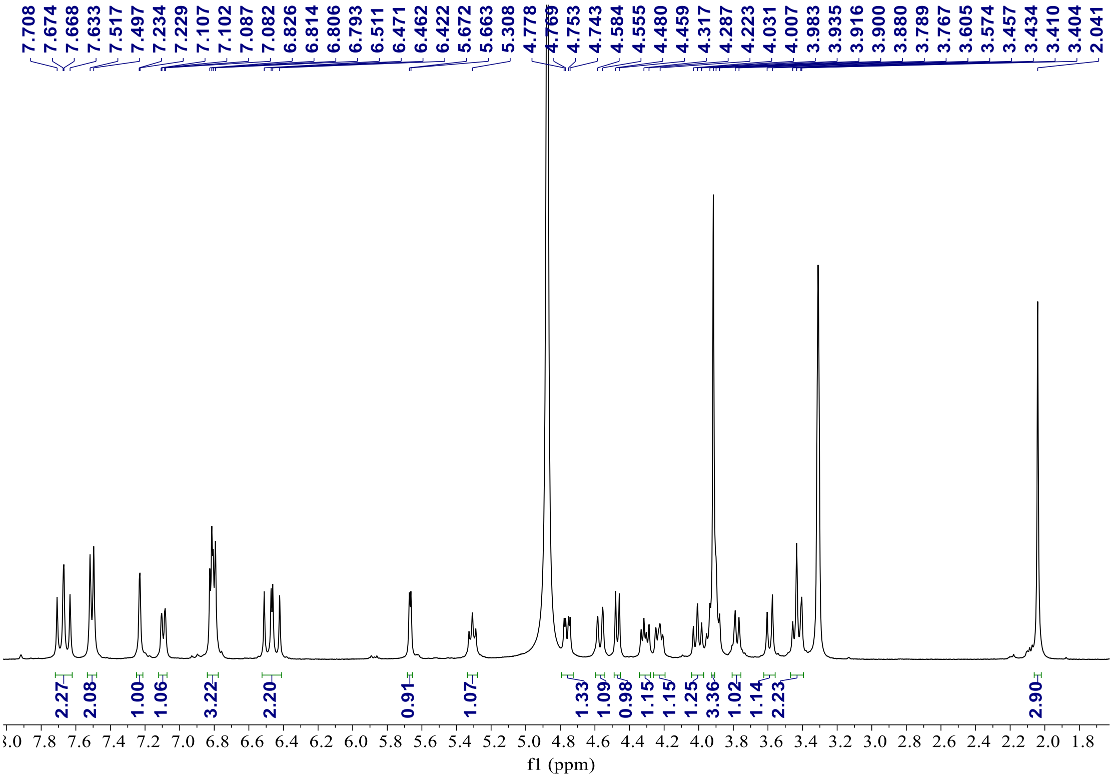

Figure S122. ¹H NMR Spectrum of Compound **14** in MeOH-d_4

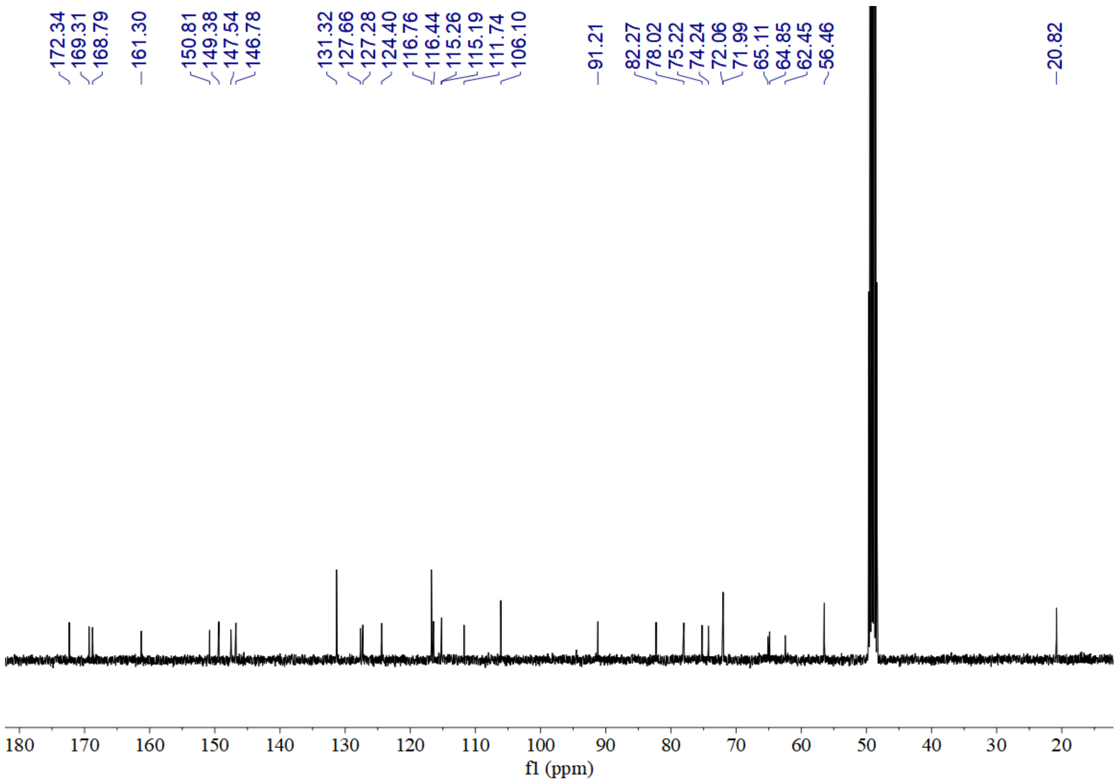

Figure S123. ^{13}C NMR Spectrum of Compound **14** in MeOH-d_4

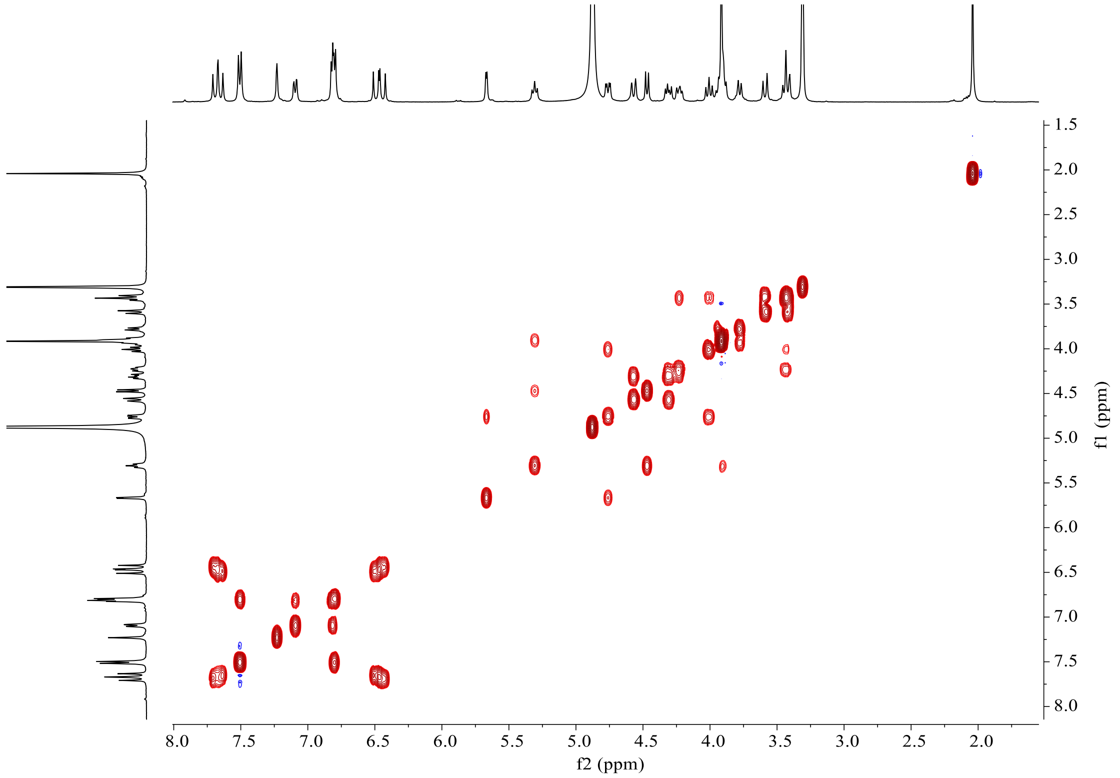

Figure S124. ^{1}H–^{1}H COSY Spectrum of Compound **14** in MeOH-d_4

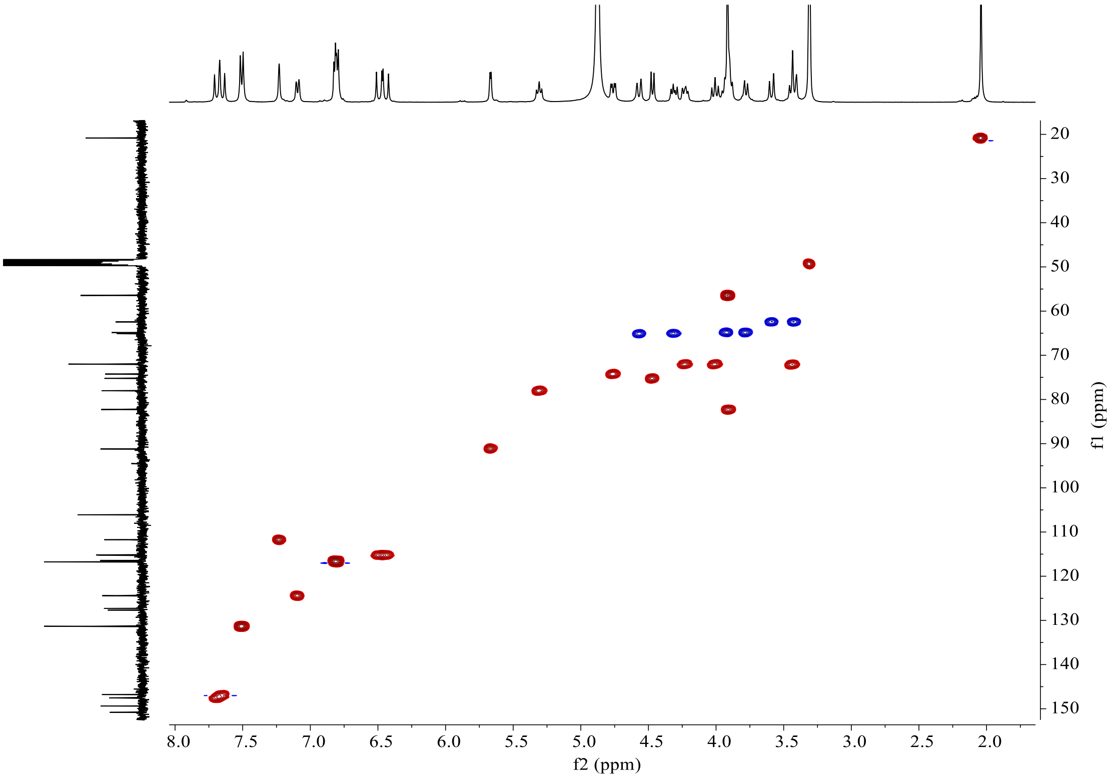

Figure S125. HSQC Spectrum of Compound **14** in MeOH-d_4

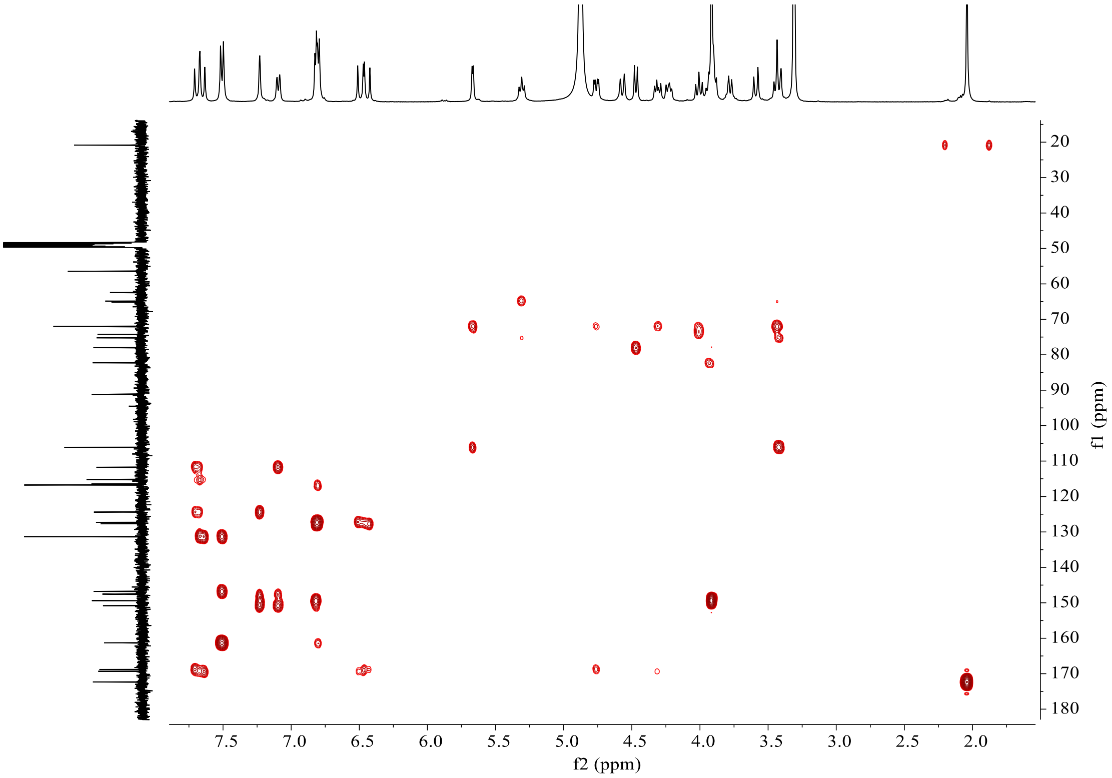

Figure S126. HMBC Spectrum of Compound **14** in MeOH-d_4

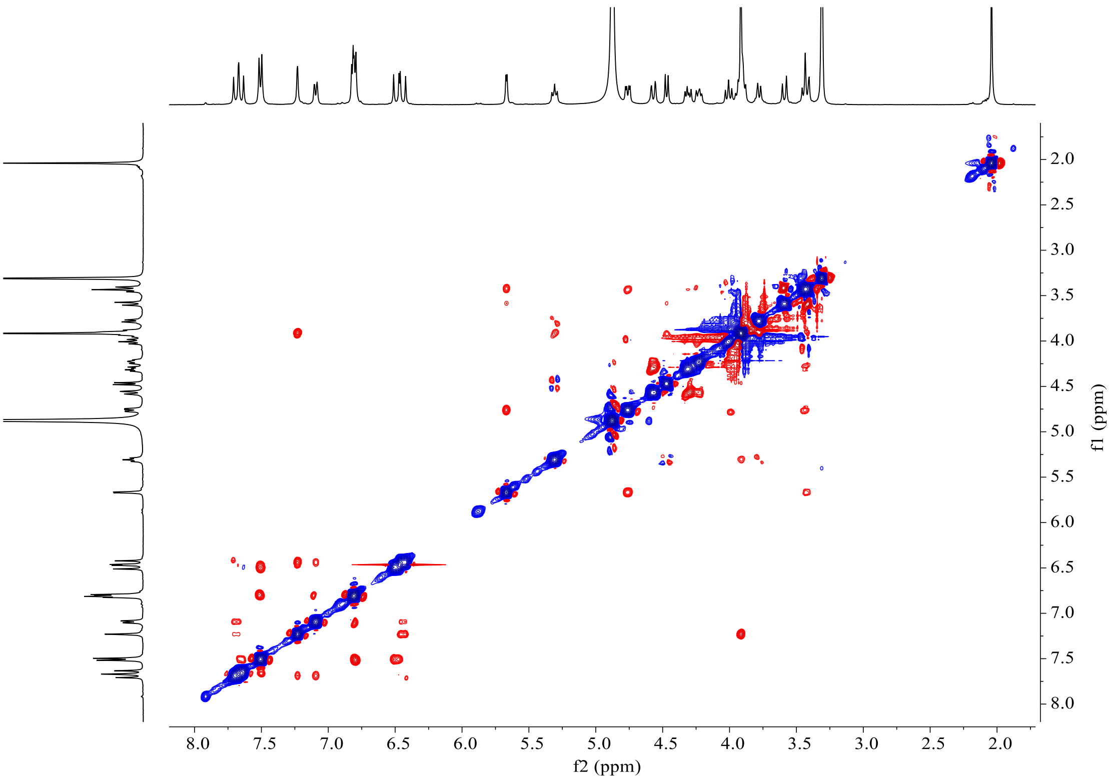

Figure S127. NOESY Spectrum of Compound **14** in MeOH-d_4

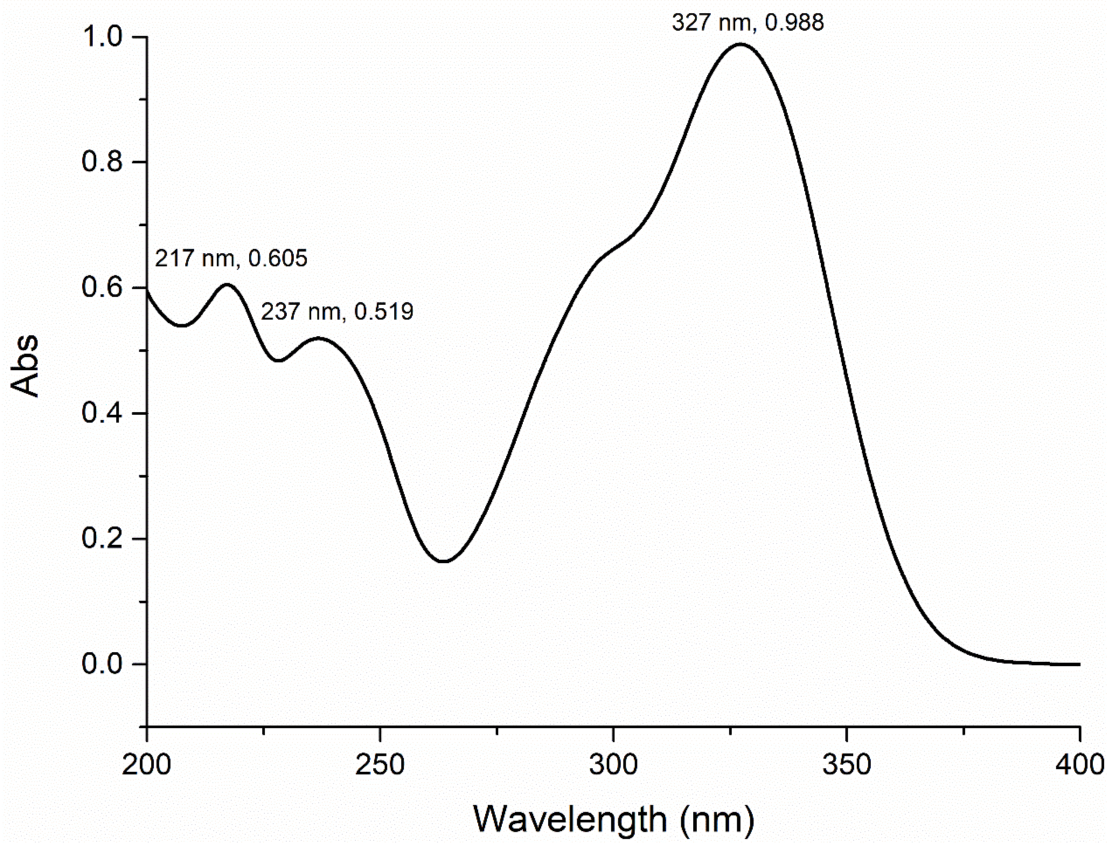

Figure S128. UV Spectrum of Compound **15**

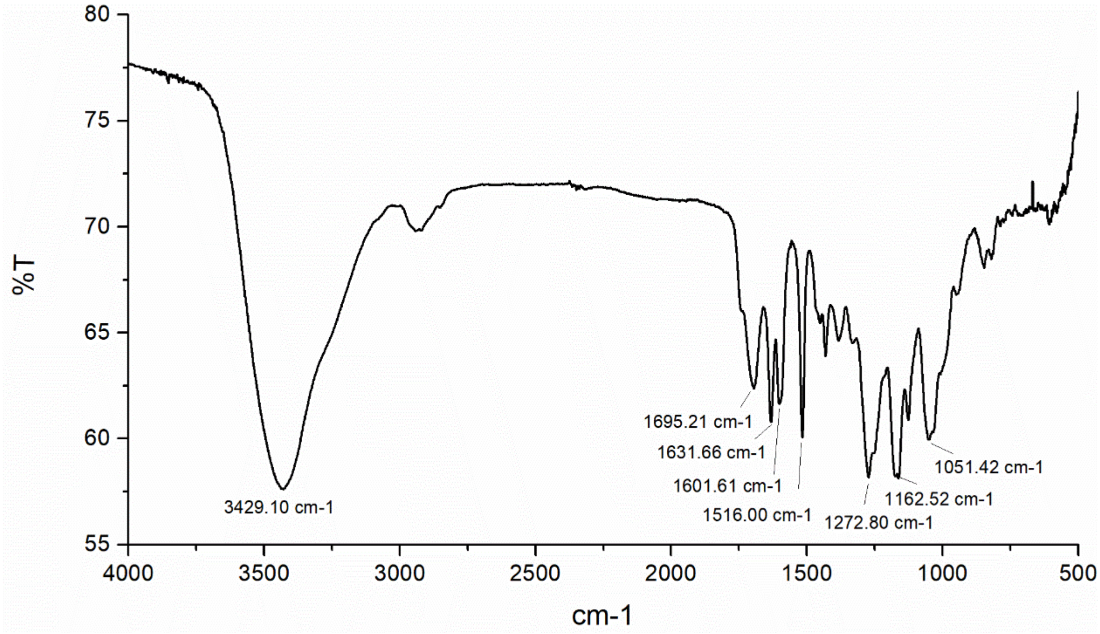

Figure S129. IR (KBr disc) Spectrum of Compound **15**

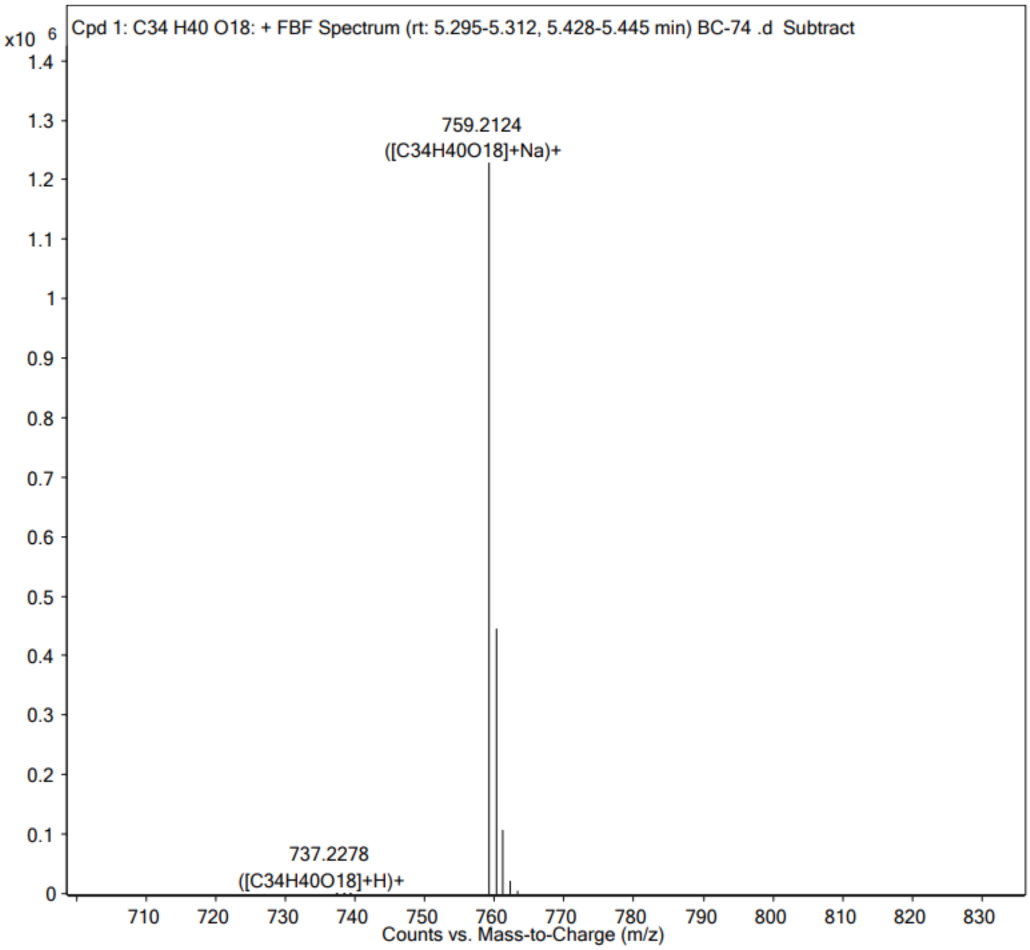

Figure S130. (+) HRESIMS Spectrum of Compound **15**

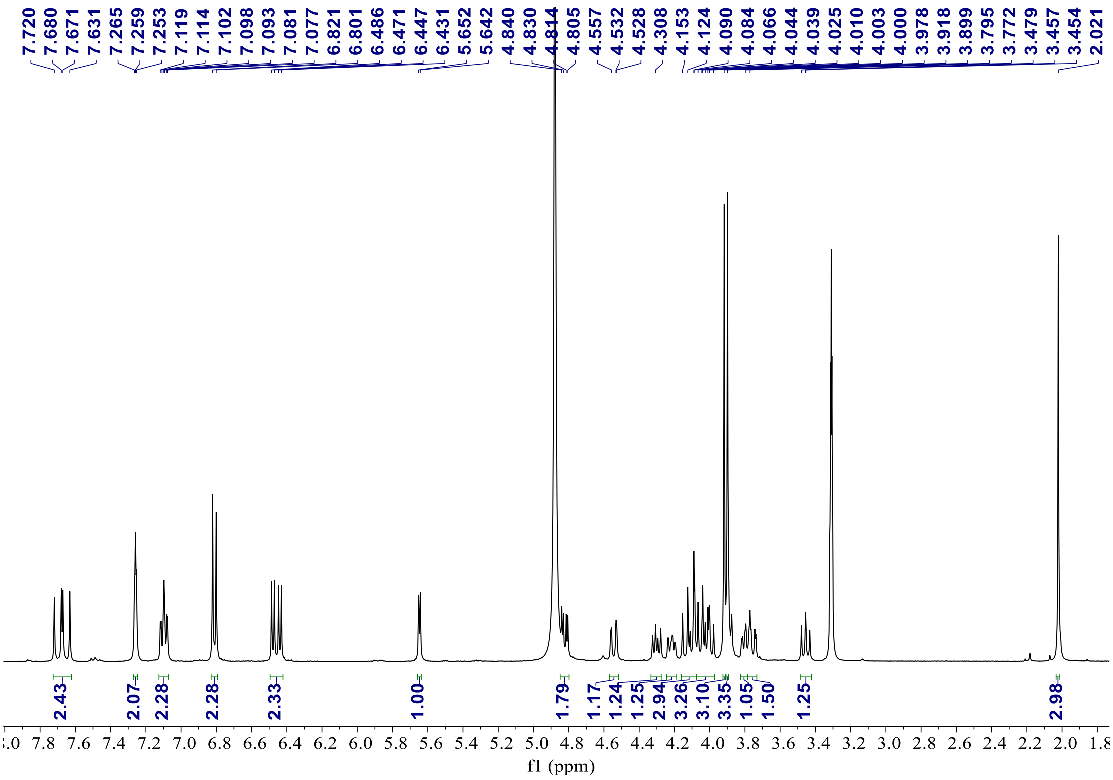

Figure S131. ^{1}H NMR Spectrum of Compound **15** in MeOH-d_4

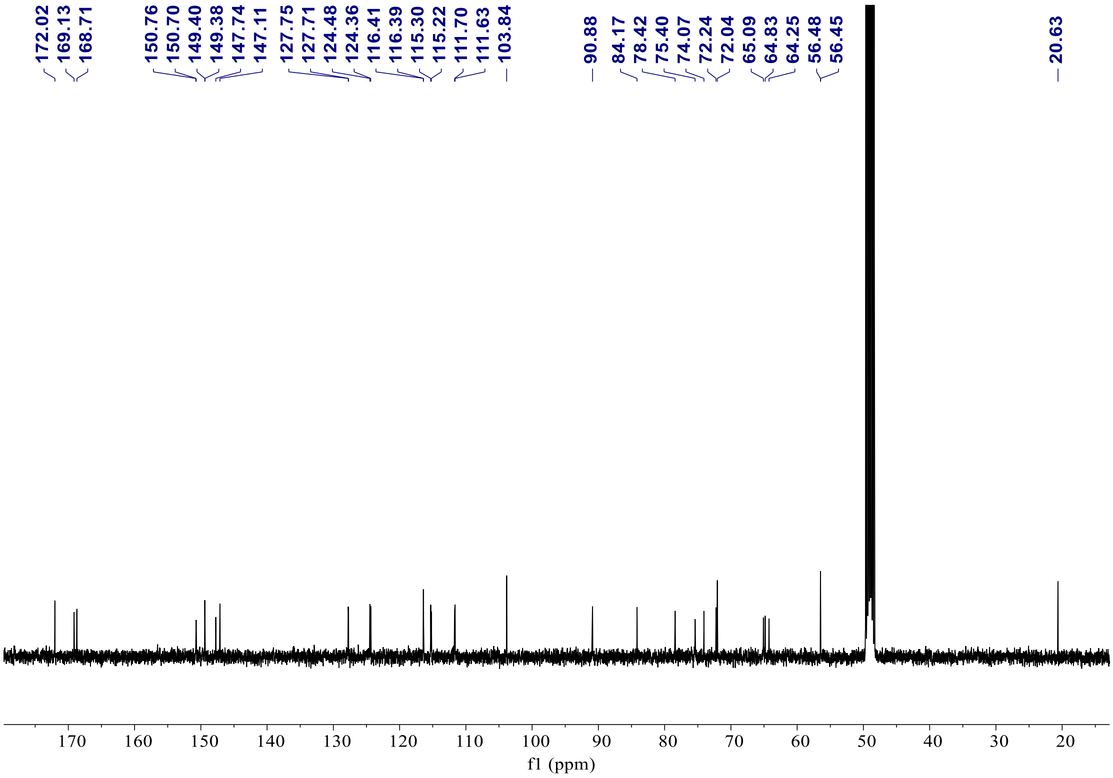

Figure S132. ^{13}C NMR Spectrum of Compound **15** in MeOH-d_4

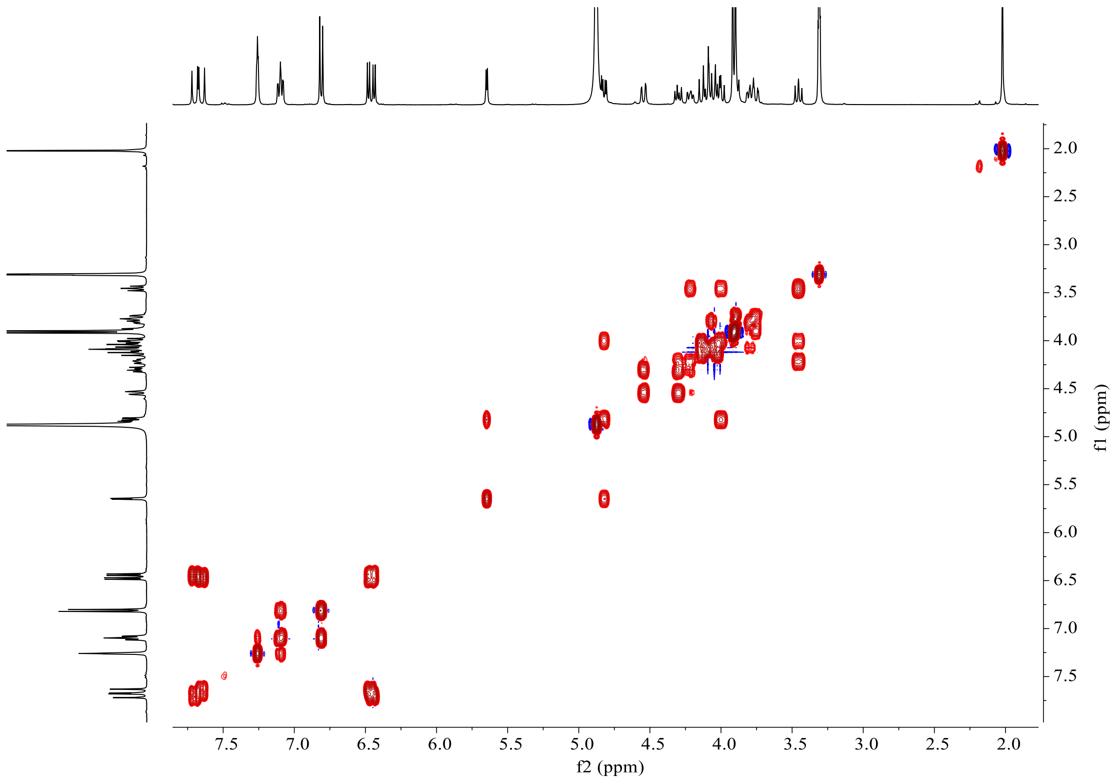

Figure S133. ^{1}H–^{1}H COSY Spectrum of Compound **15** in MeOH-d_4

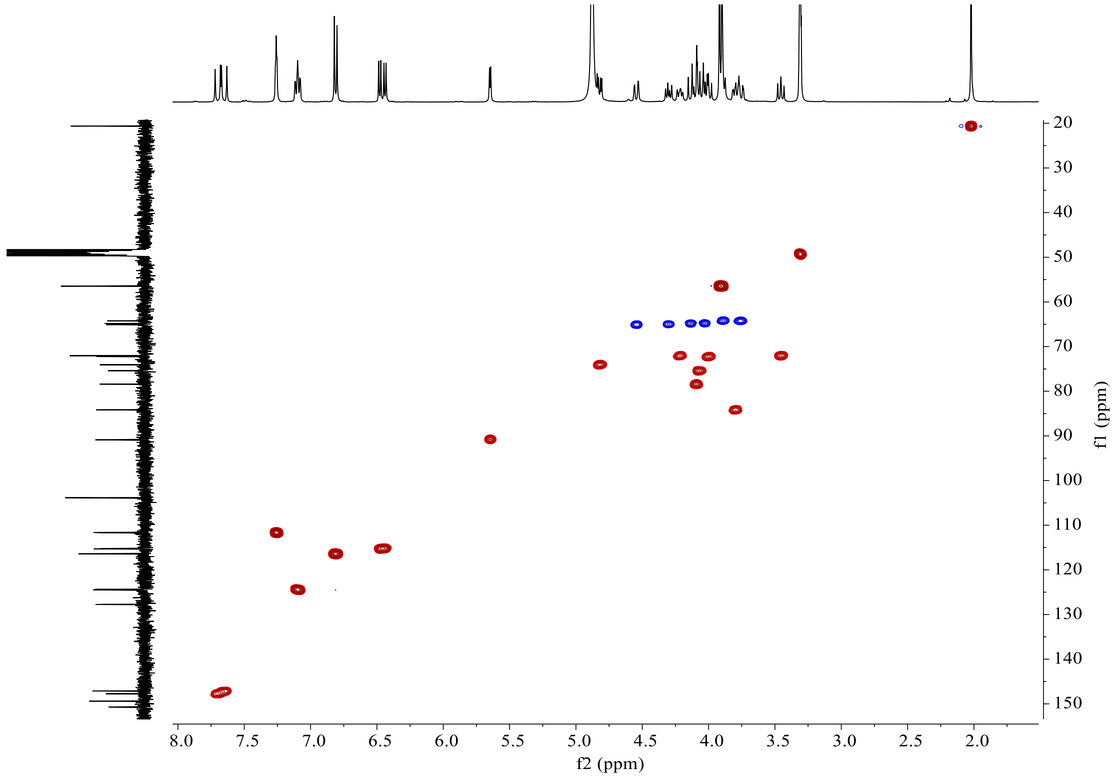

Figure S134. HSQC Spectrum of Compound **15** in MeOH-d_4

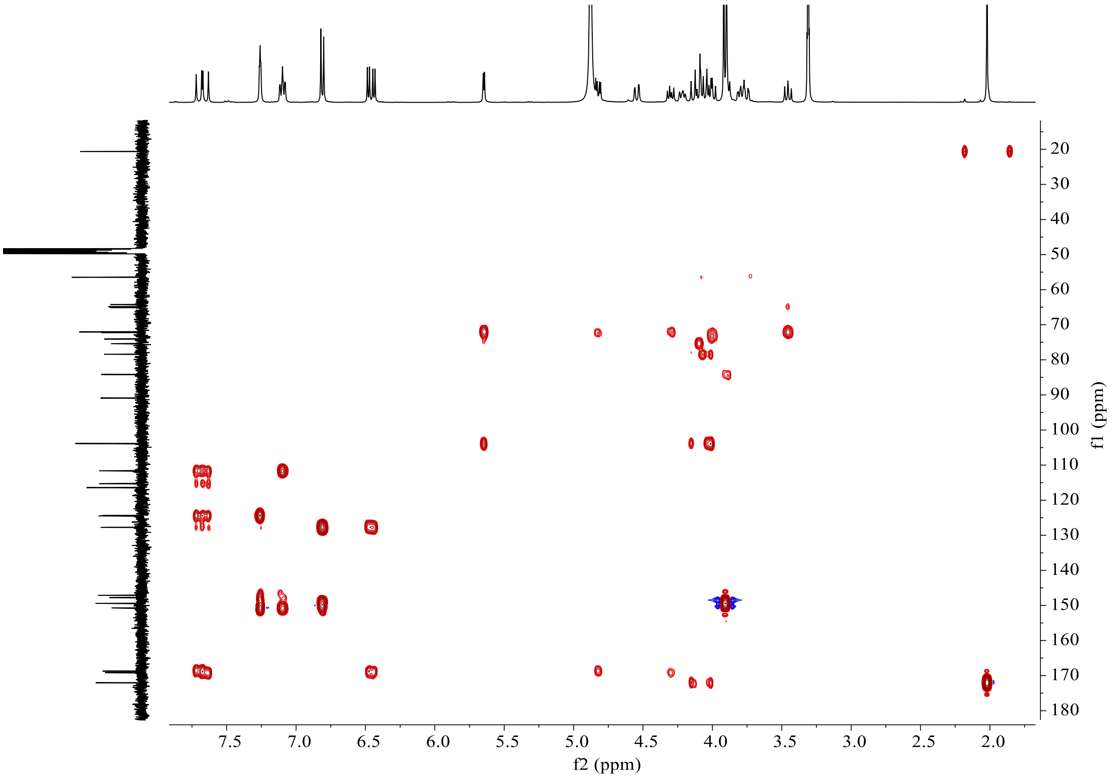

Figure S135. HMBC Spectrum of Compound **15** in MeOH-d_4

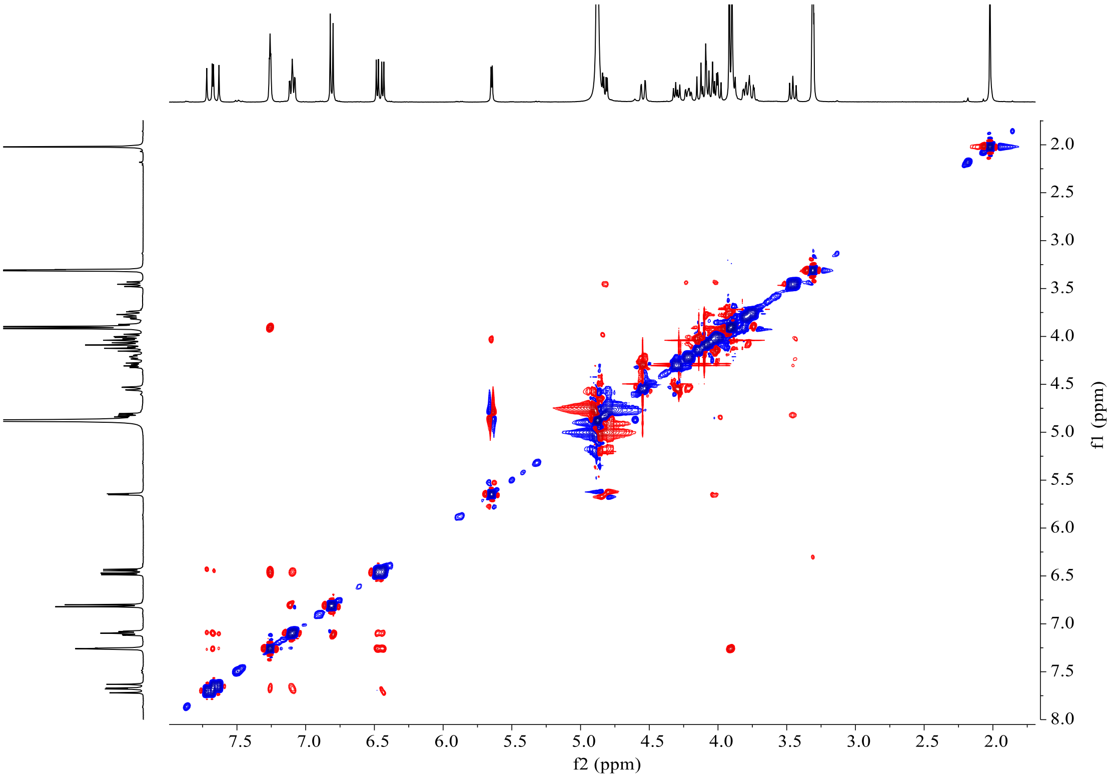

Figure S136. NOESY Spectrum of Compound **15** in MeOH-d_4

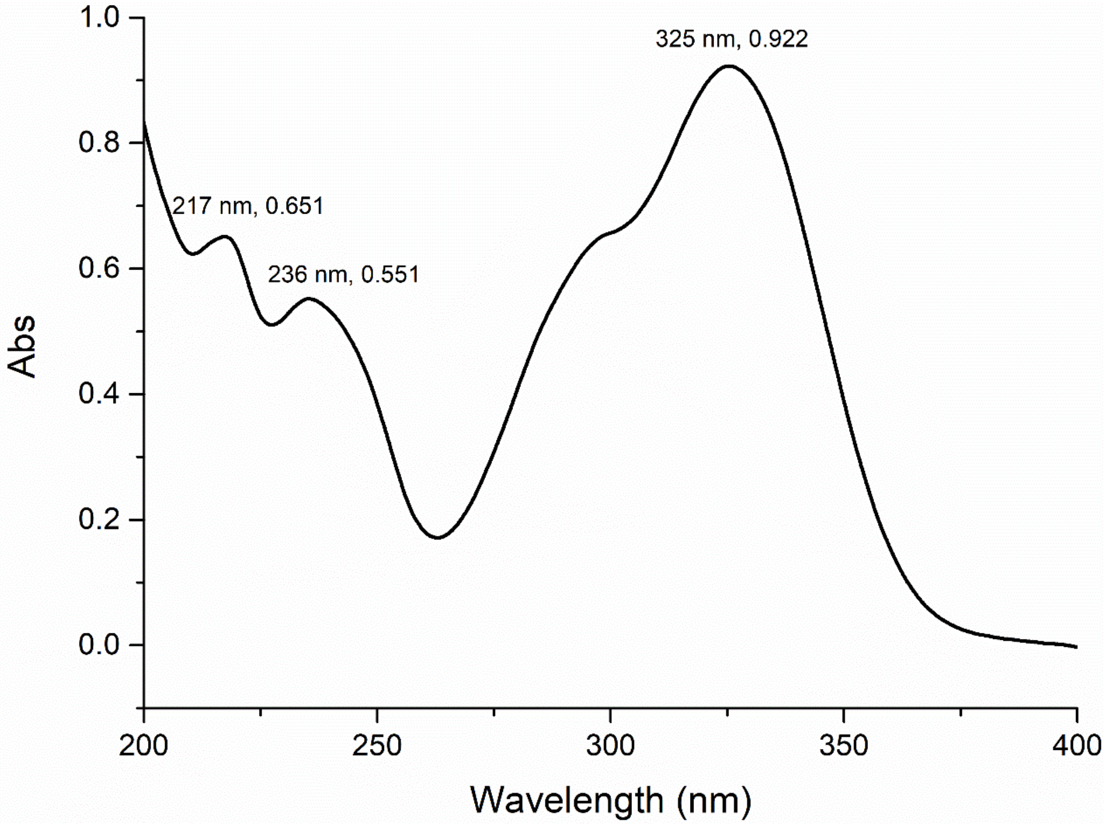

Figure S137. UV Spectrum of Compound **16**

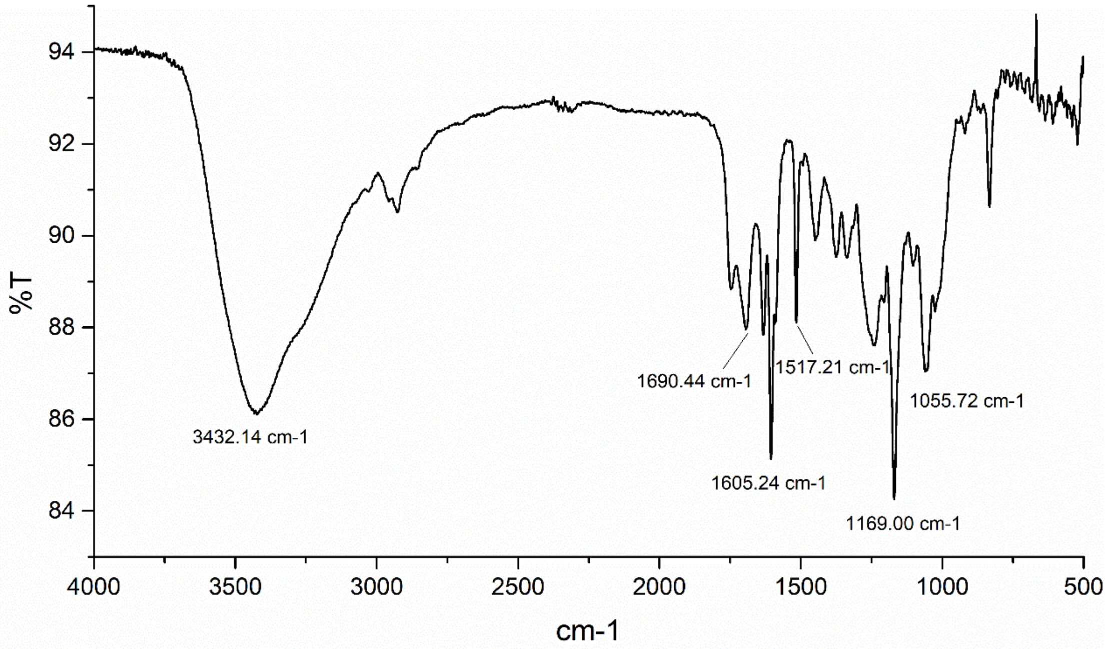

Figure S138. IR (KBr disc) Spectrum of Compound **16**

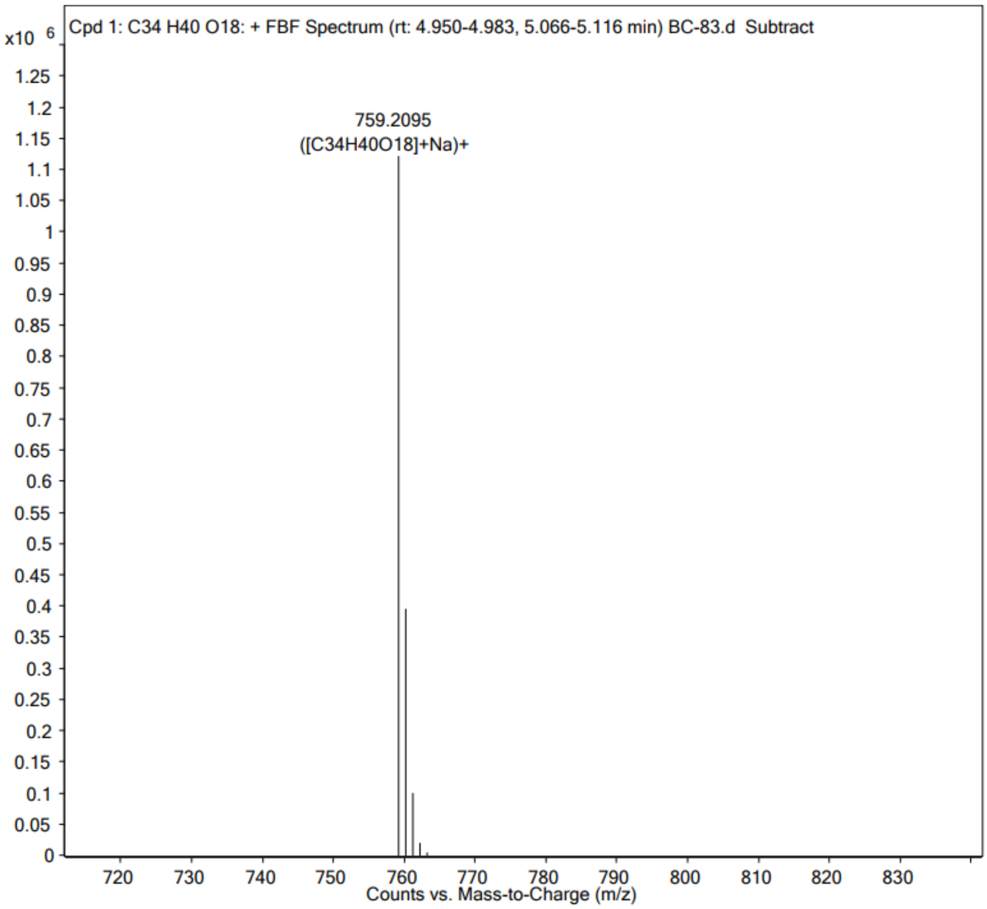

Figure S139. (+) HRESIMS Spectrum of Compound **16**

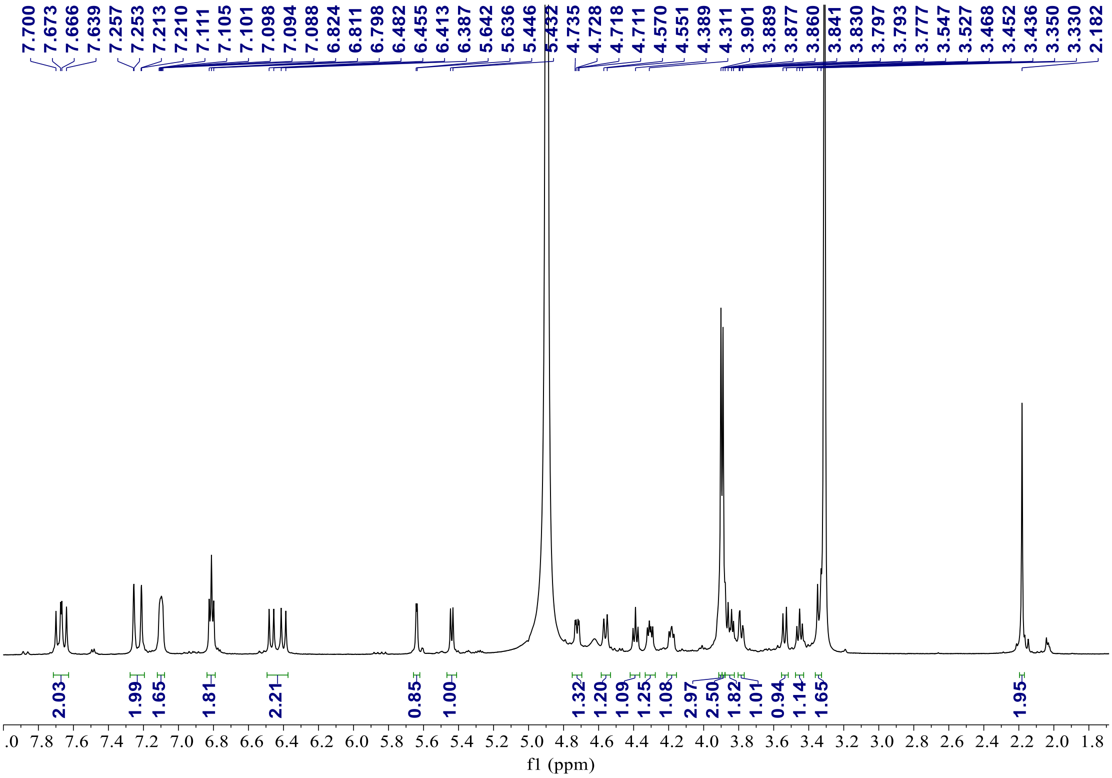

Figure S140. ^{1}H NMR Spectrum of Compound **16** in MeOH-d_4

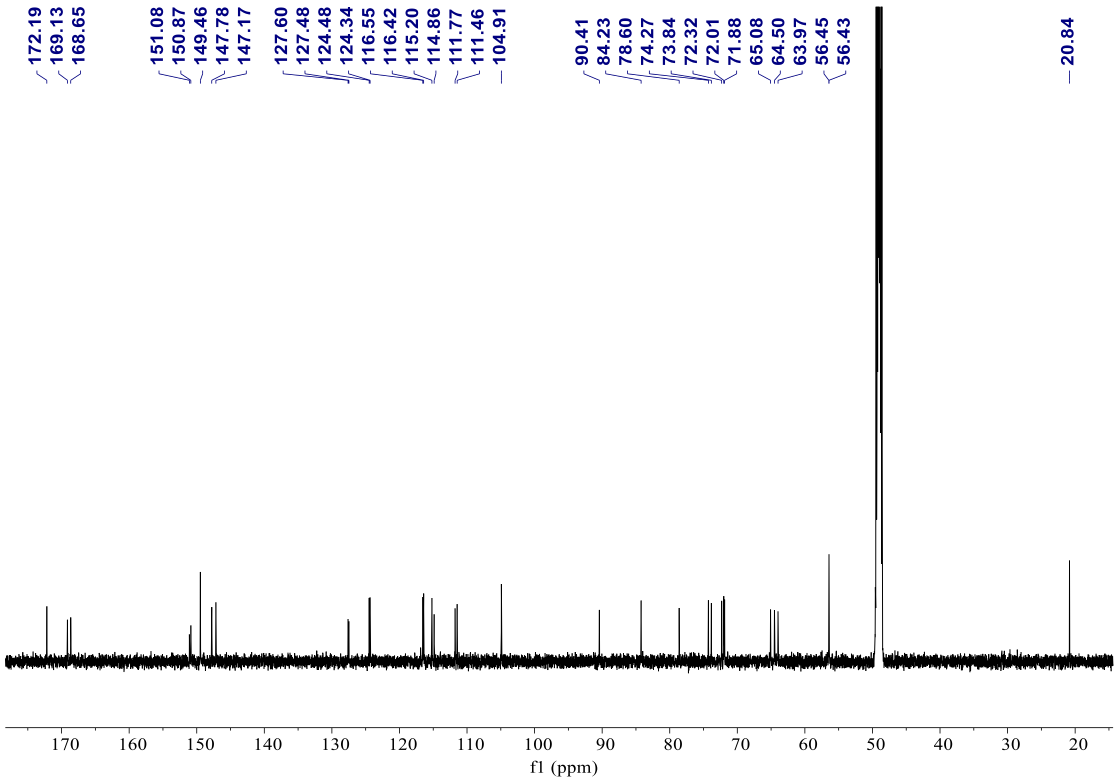

Figure S141. ^{13}C NMR Spectrum of Compound **16** in MeOH-d_4

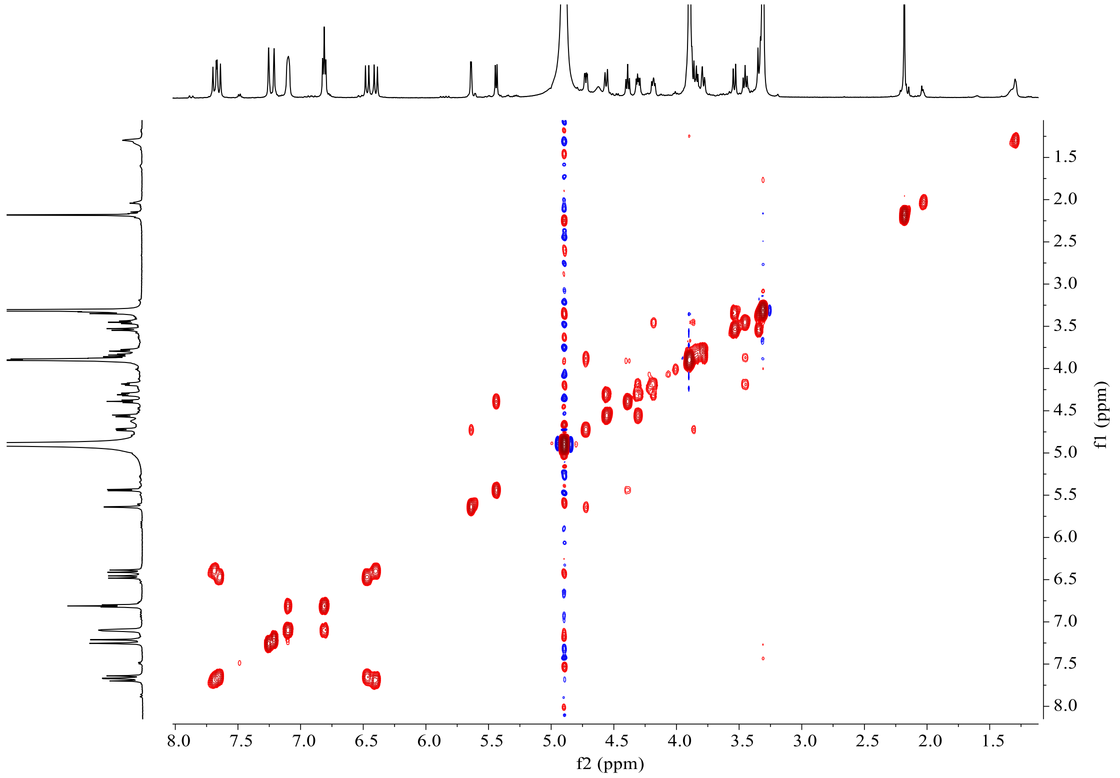

Figure S142. ^{1}H–^{1}H COSY Spectrum of Compound **16** in MeOH-d_4

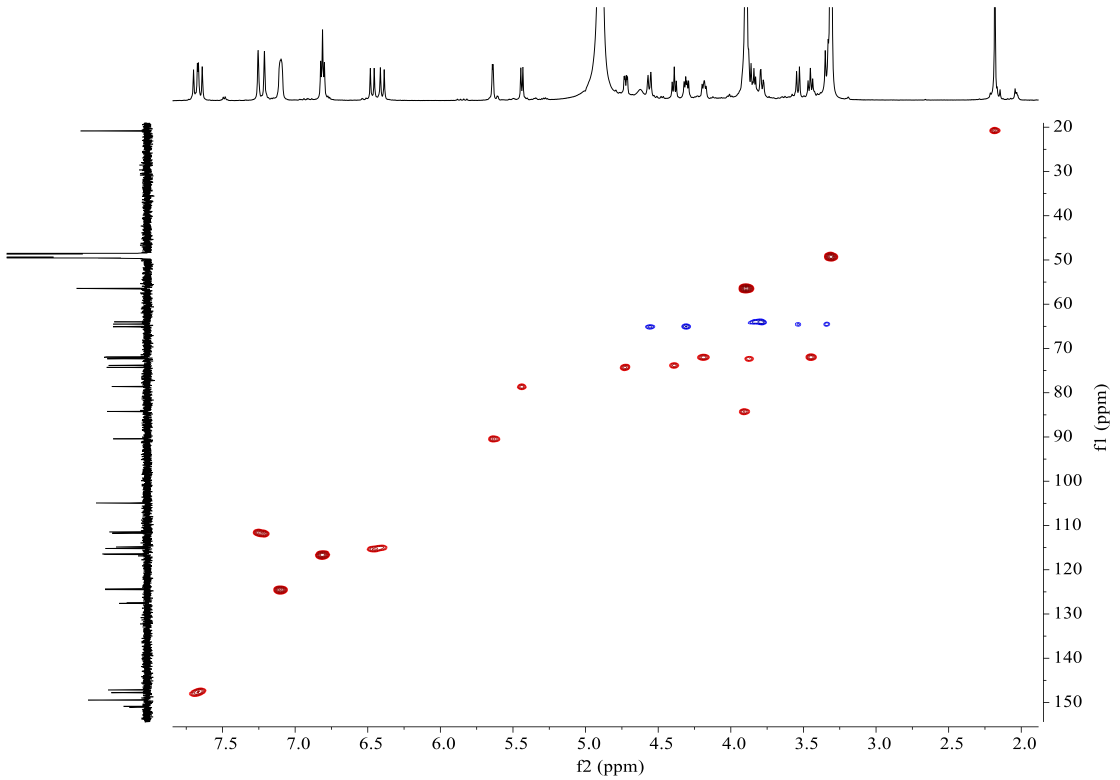

Figure S143. HSQC Spectrum of Compound **16** in MeOH-d_4

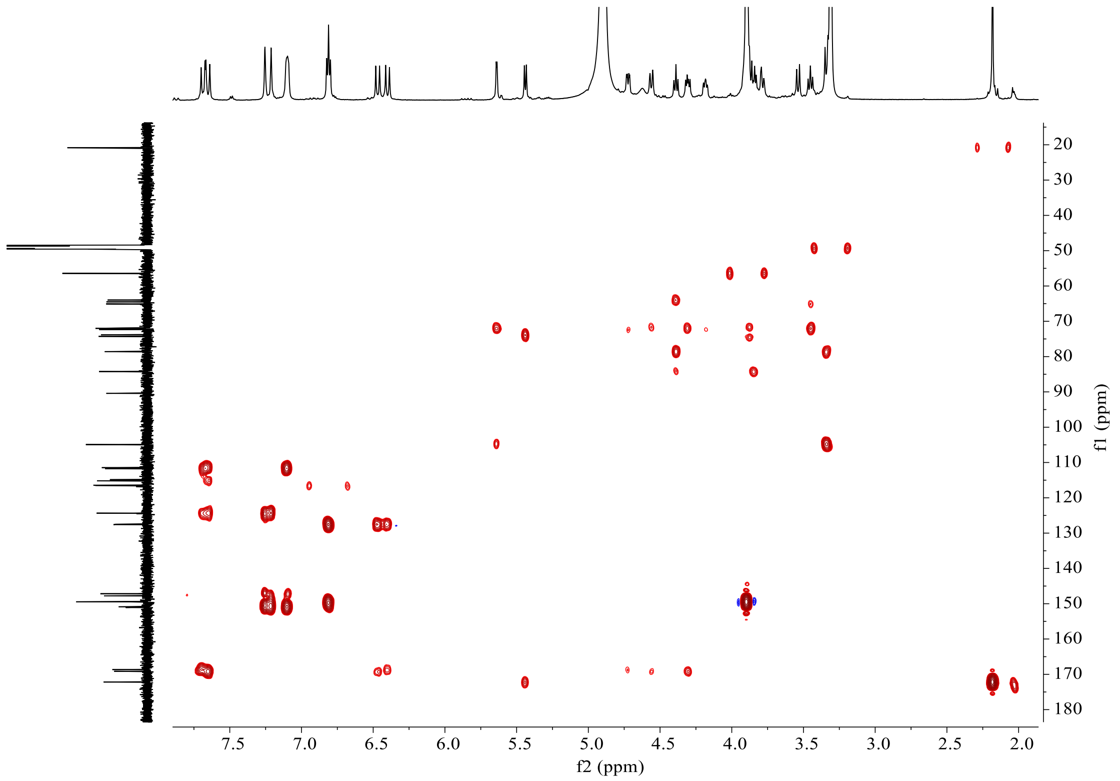

Figure S144. HMBC Spectrum of Compound **16** in MeOH-d_4

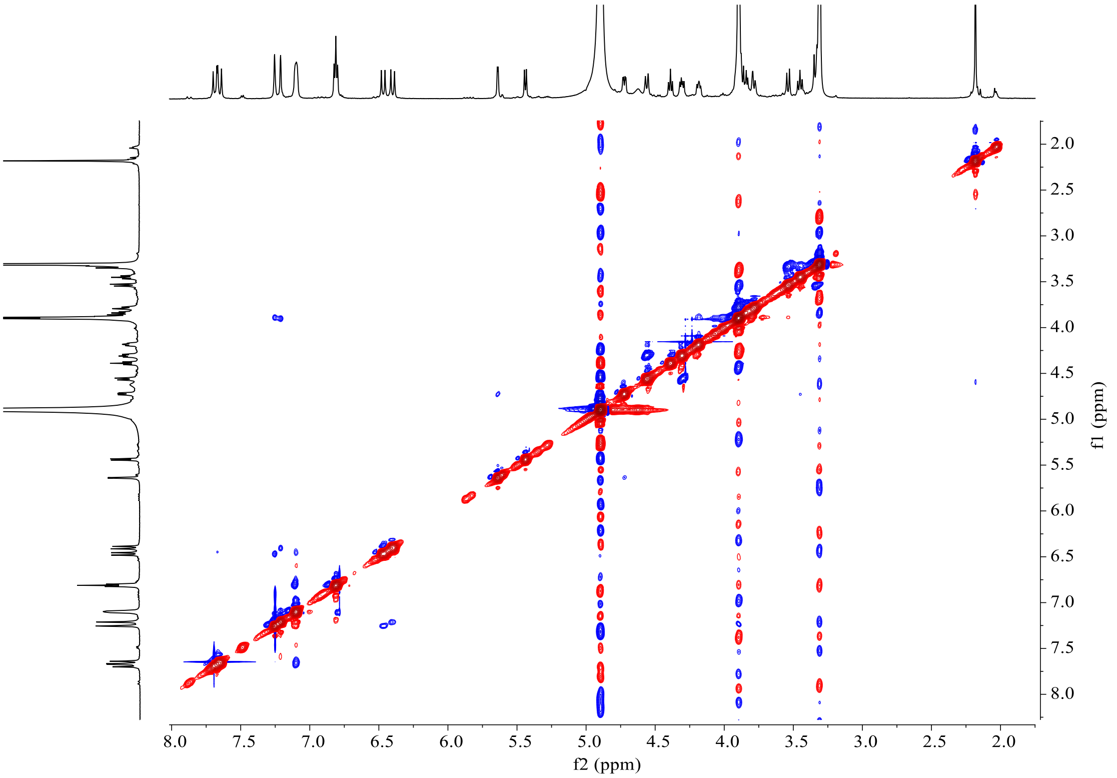

Figure S145. NOESY Spectrum of Compound **16** in MeOH-d_4

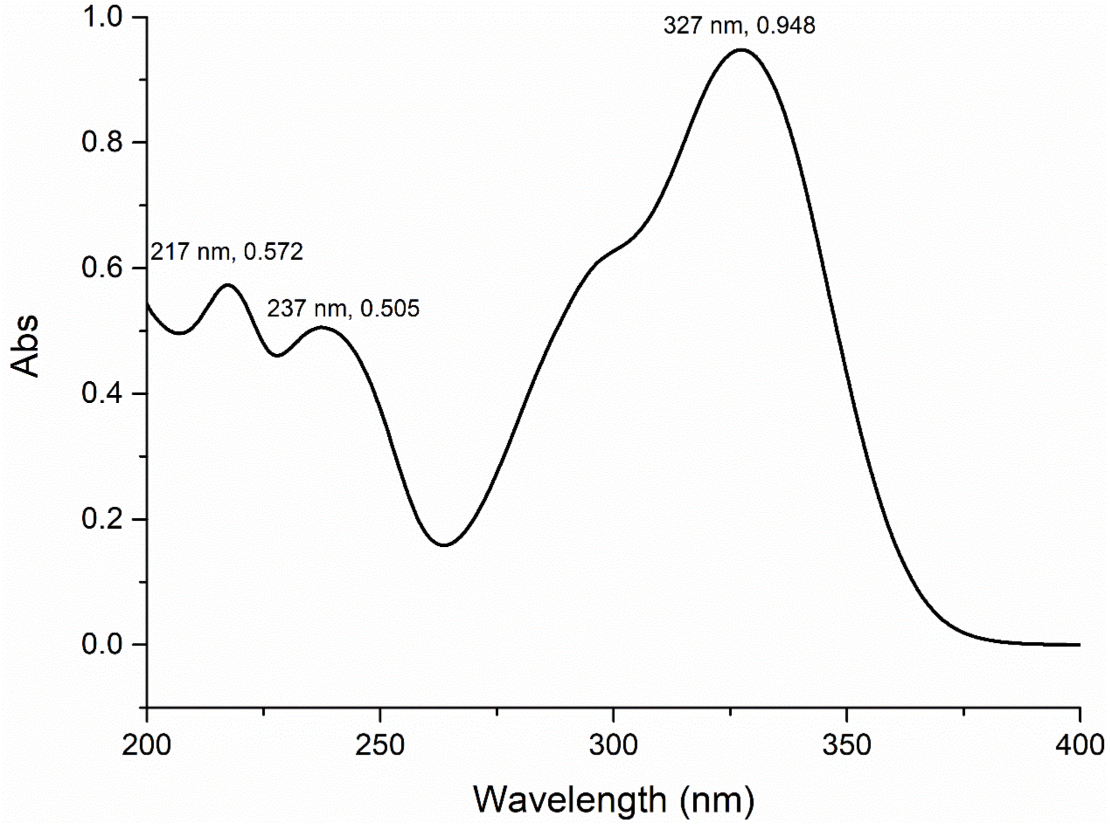

Figure S146. UV Spectrum of Compound **17**

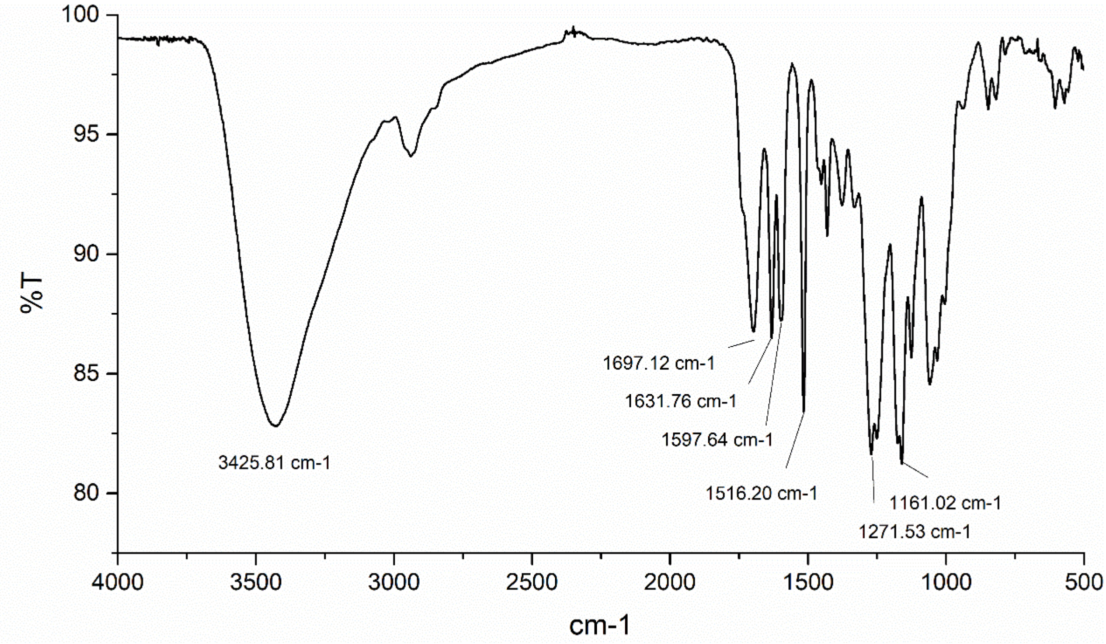

Figure S147. IR (KBr disc) Spectrum of Compound **17**

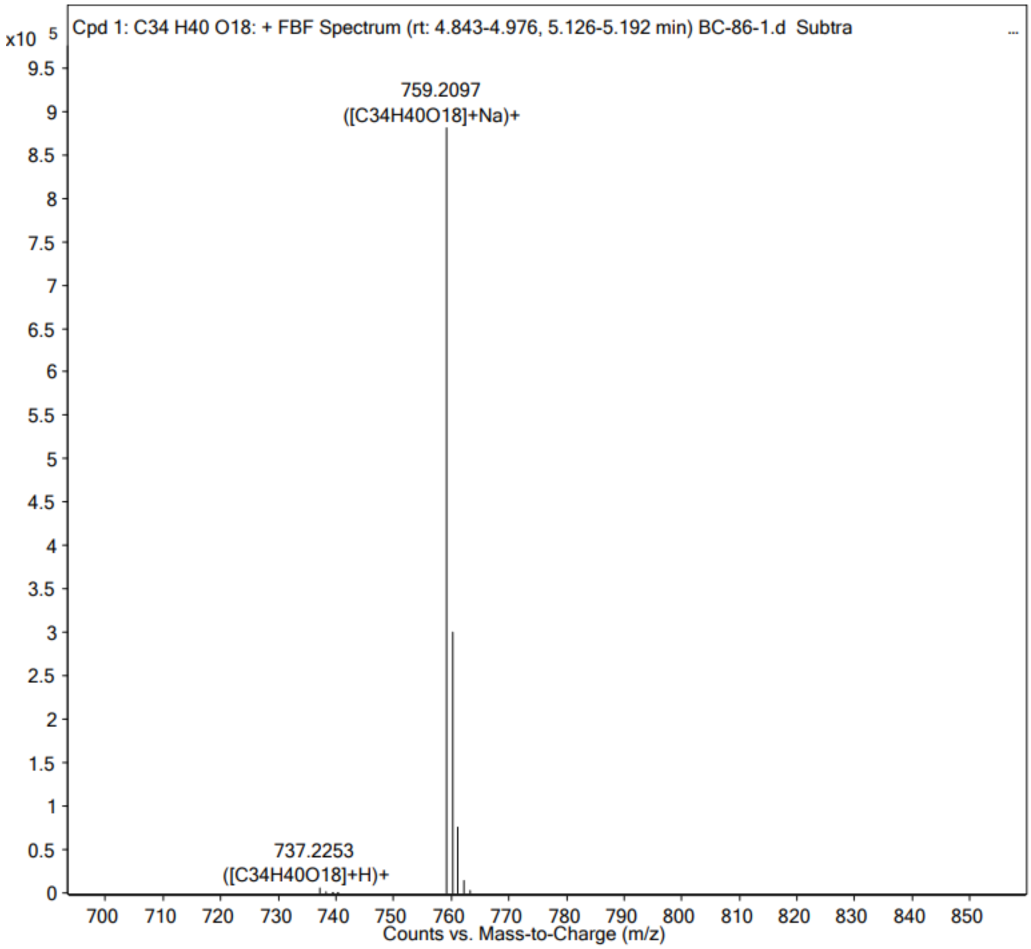

Figure S148. (+) HRESIMS Spectrum of Compound **17**

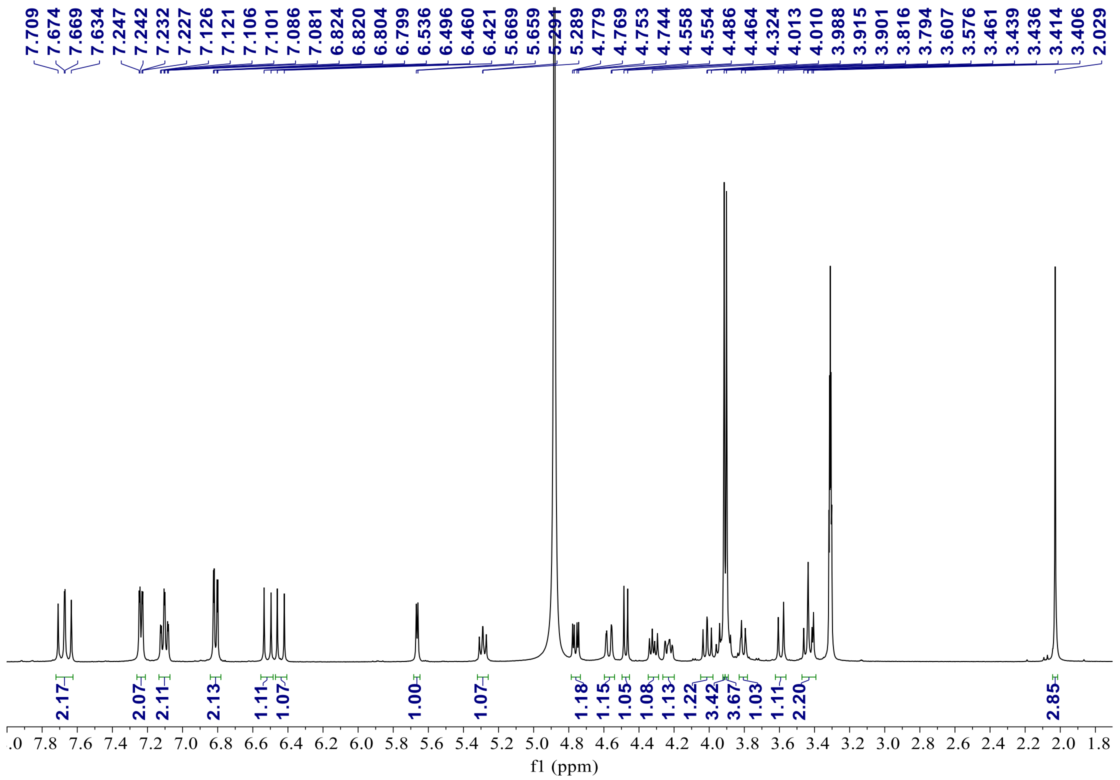

Figure S149. ^{1}H NMR Spectrum of Compound **17** in MeOH-d_4

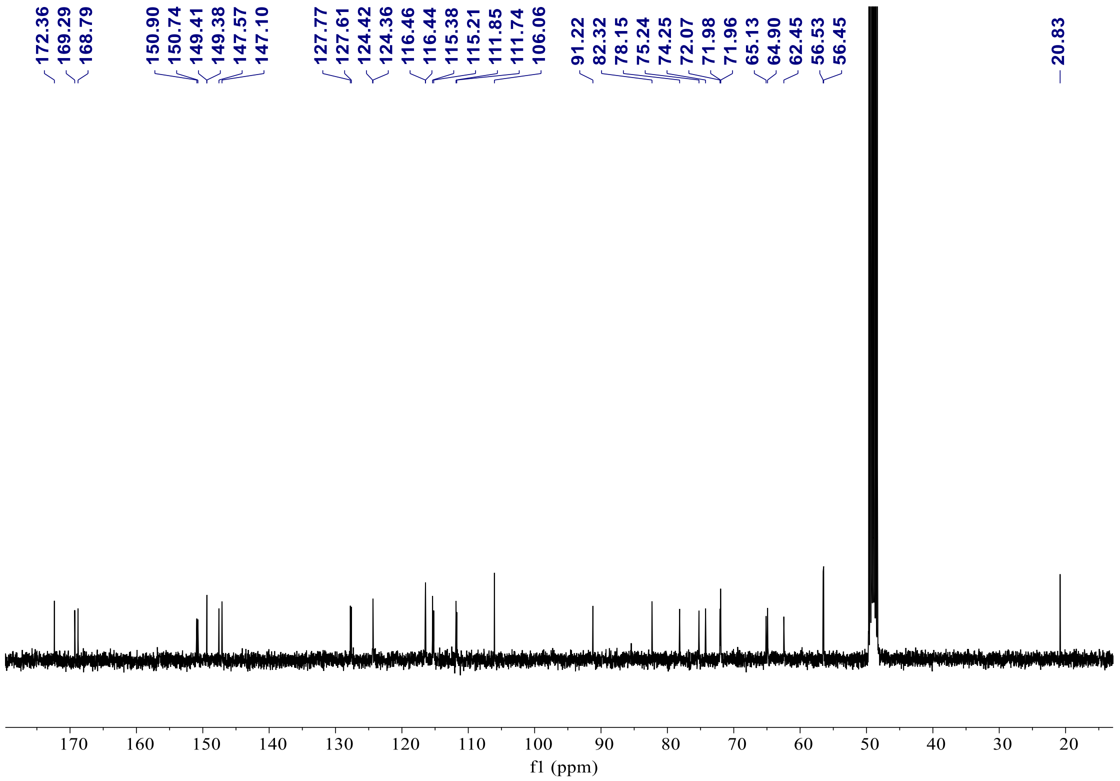

Figure S150. ^{13}C NMR Spectrum of Compound **17** in MeOH-d_4

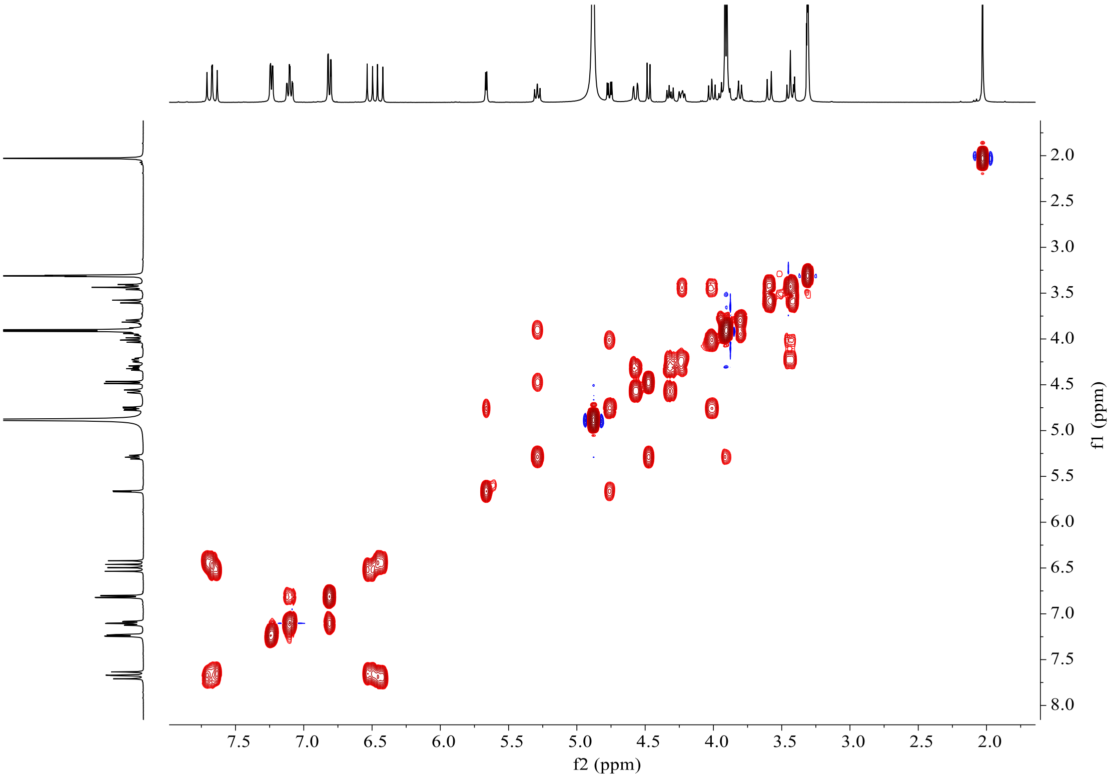

Figure S151. ^{1}H–^{1}H COSY Spectrum of Compound **17** in MeOH-d_4

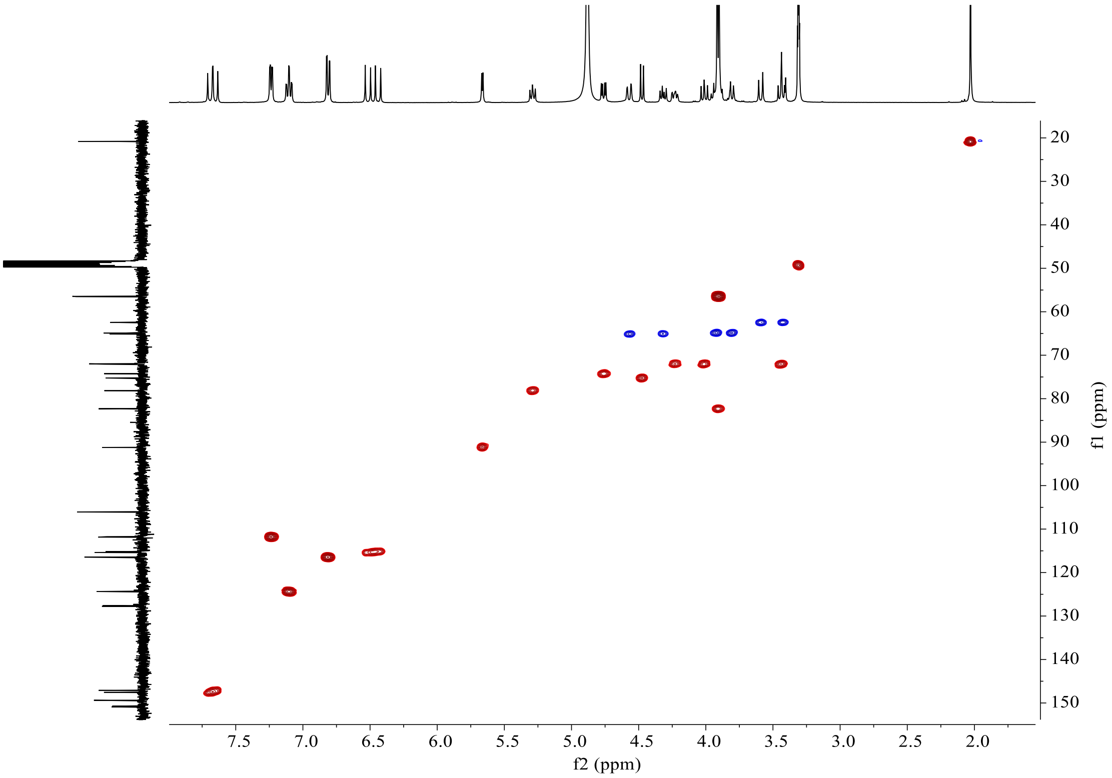

Figure S152. HSQC Spectrum of Compound **17** in MeOH-d_4

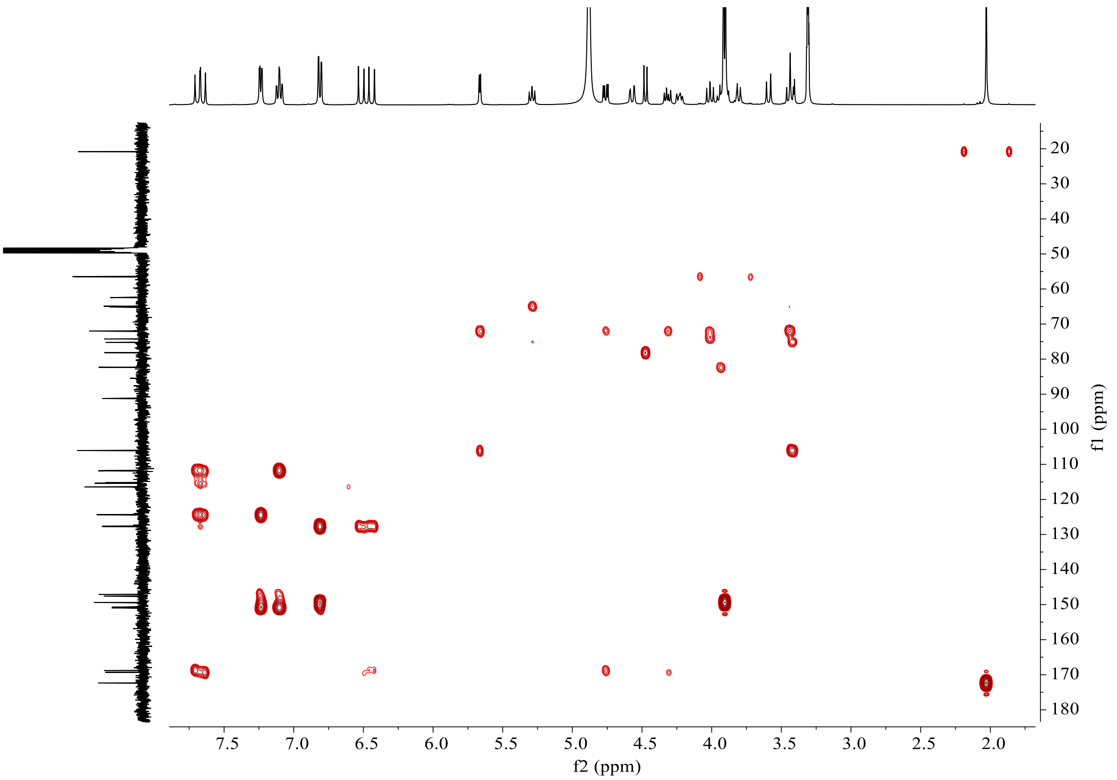

Figure S153. HMBC Spectrum of Compound **17** in MeOH-d_4

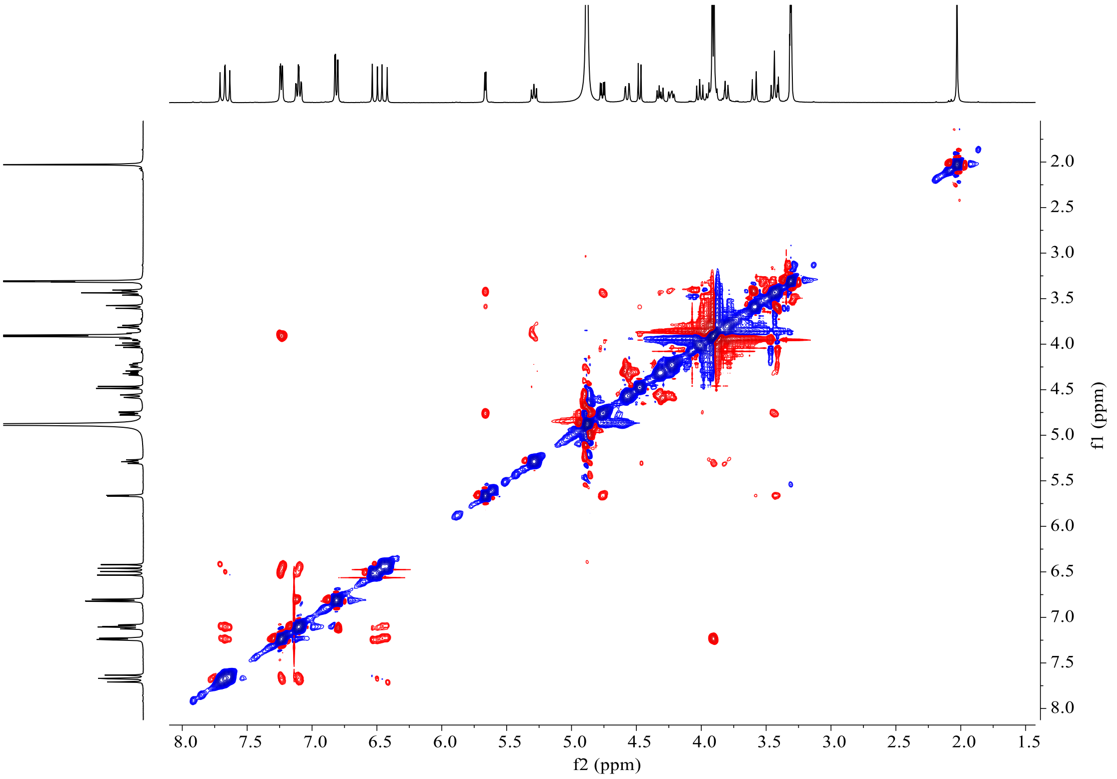

Figure S154. NOESY Spectrum of Compound **17** in MeOH-d_4

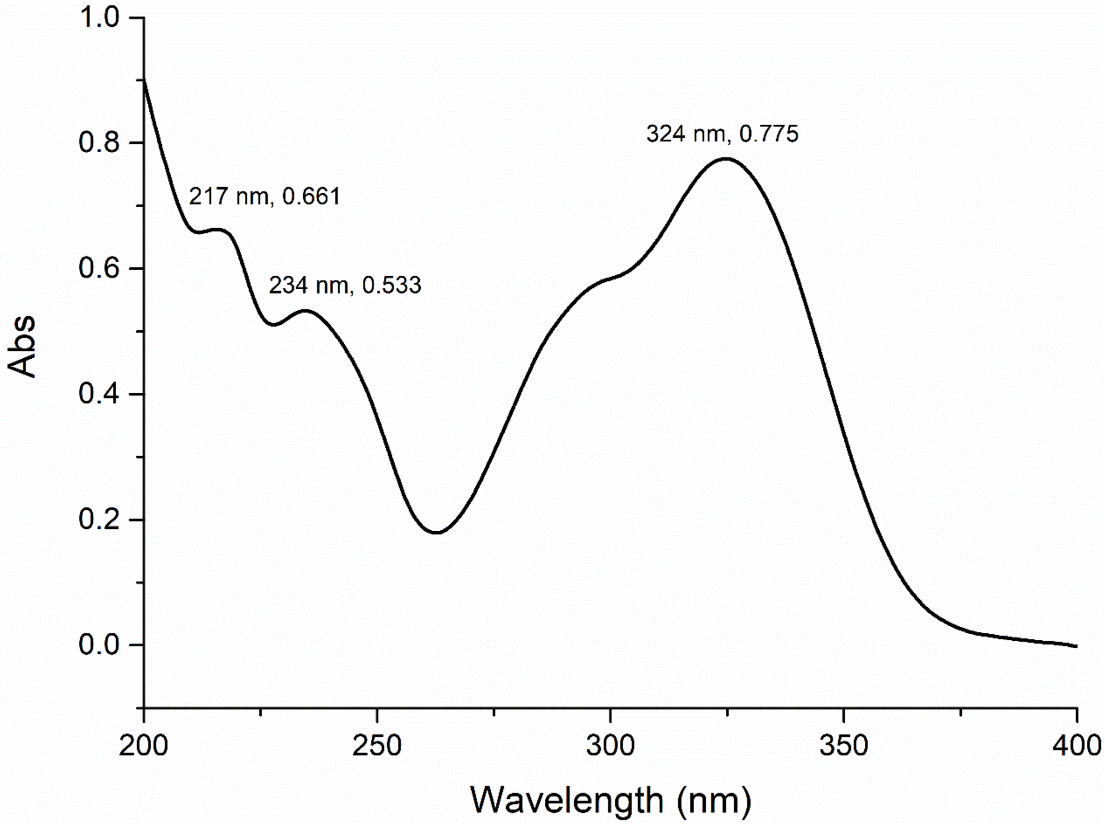

Figure S155. UV Spectrum of Compound **18**

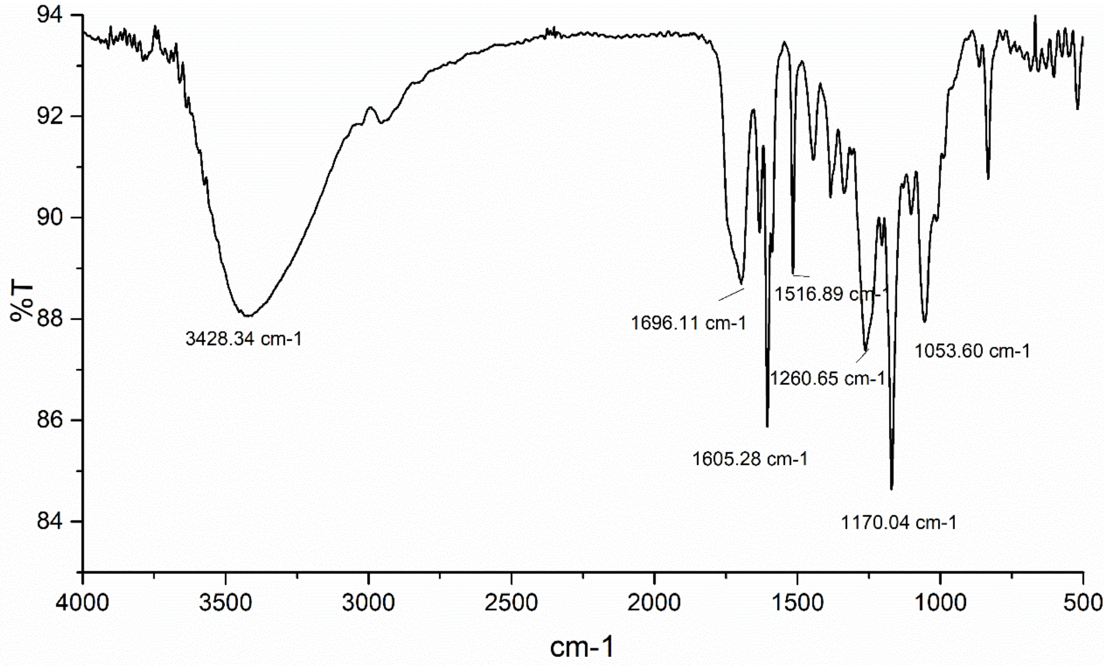

Figure S156. IR (KBr disc) Spectrum of Compound **18**

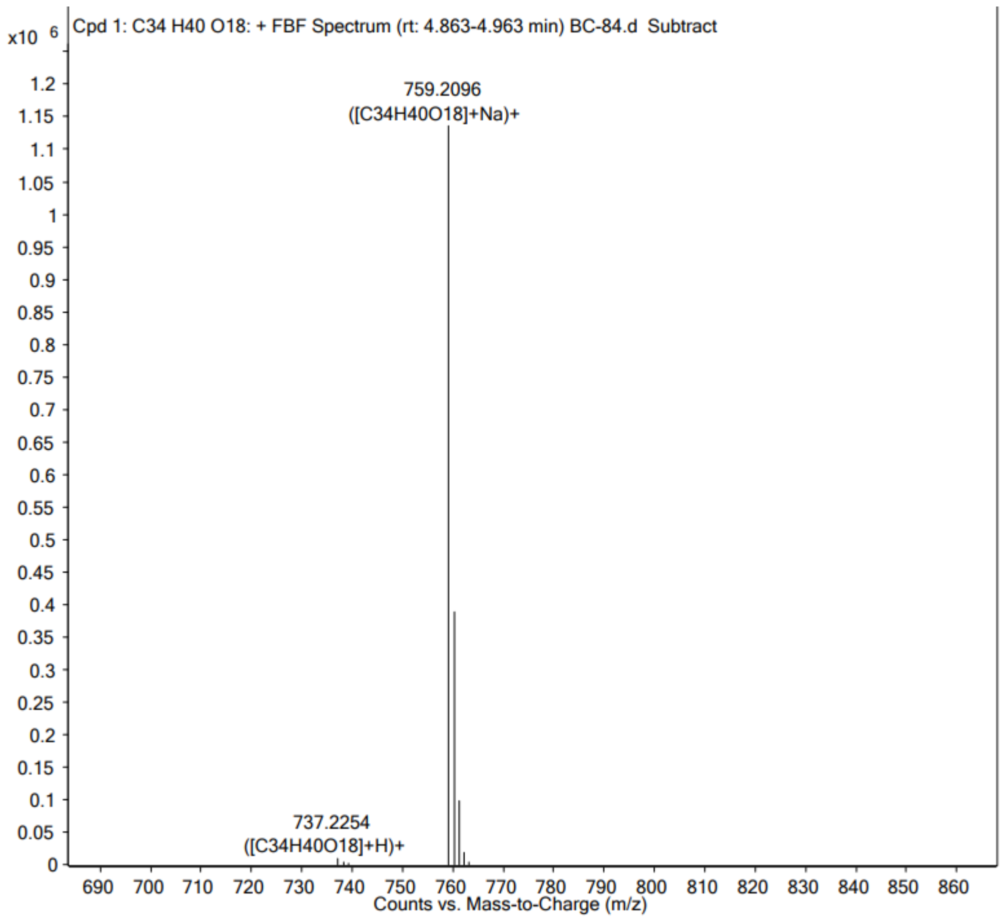

Figure S157. (+) HRESIMS Spectrum of Compound **18**

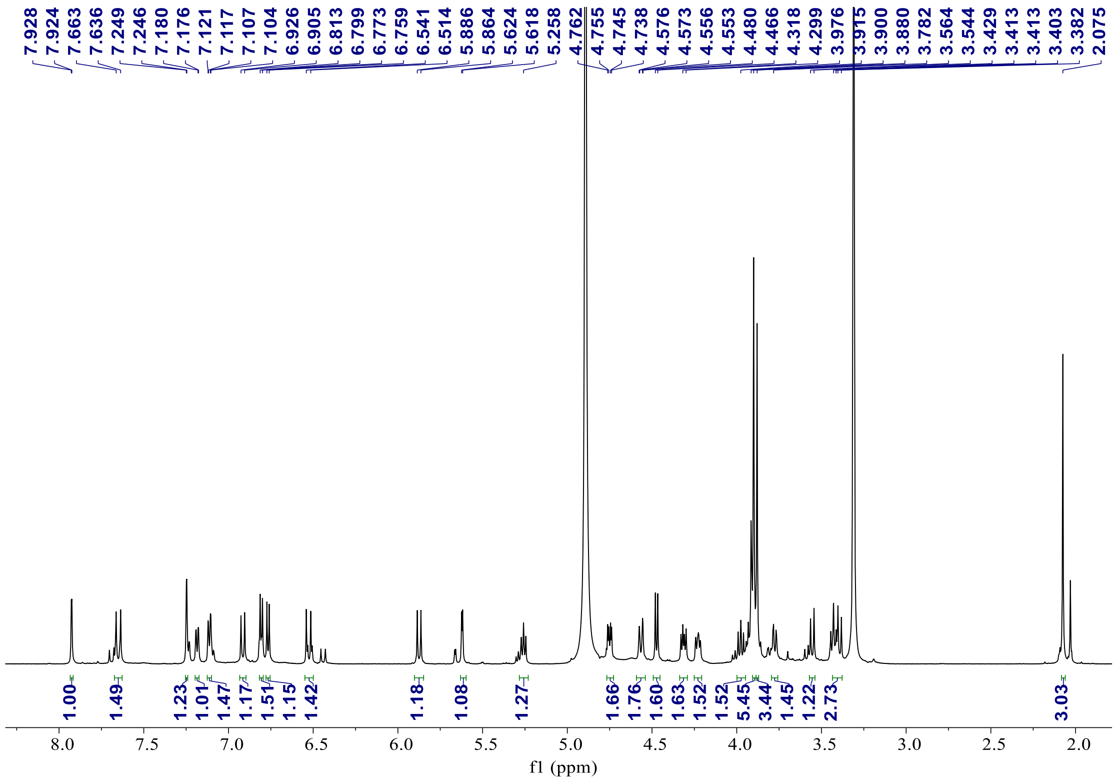

Figure S158. ^{1}H NMR Spectrum of Compound **18** in MeOH-d_4

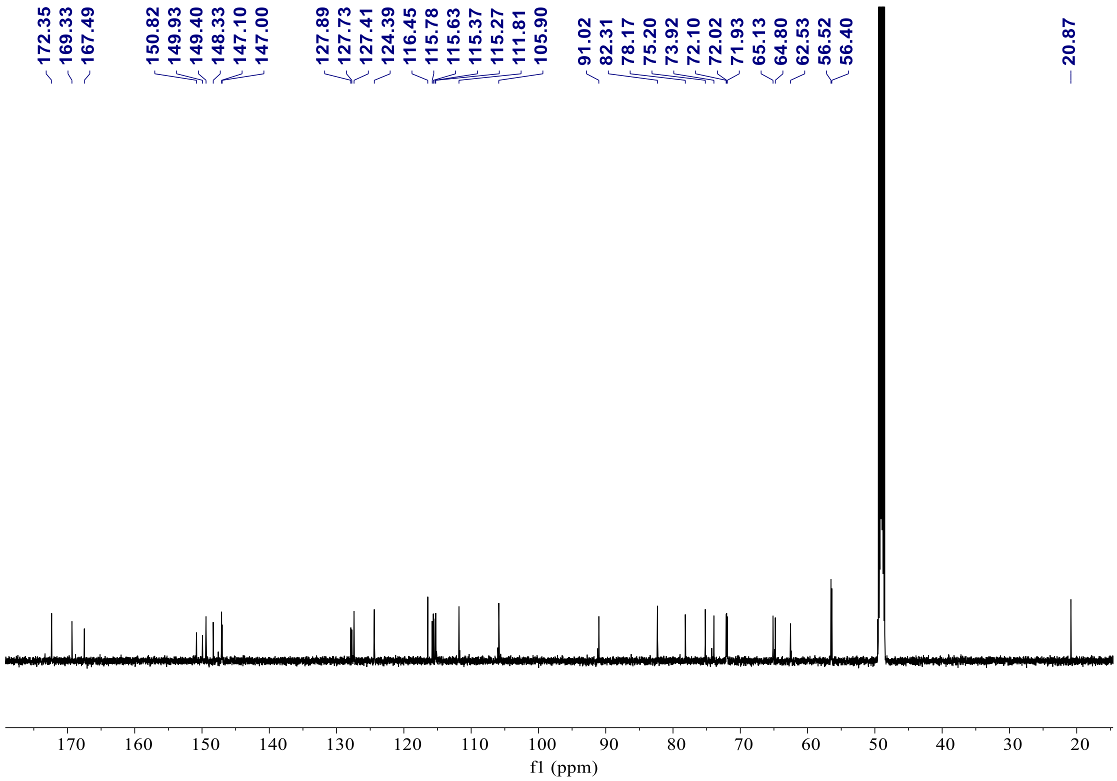

Figure S159. ^{13}C NMR Spectrum of Compound **18** in MeOH-d_4

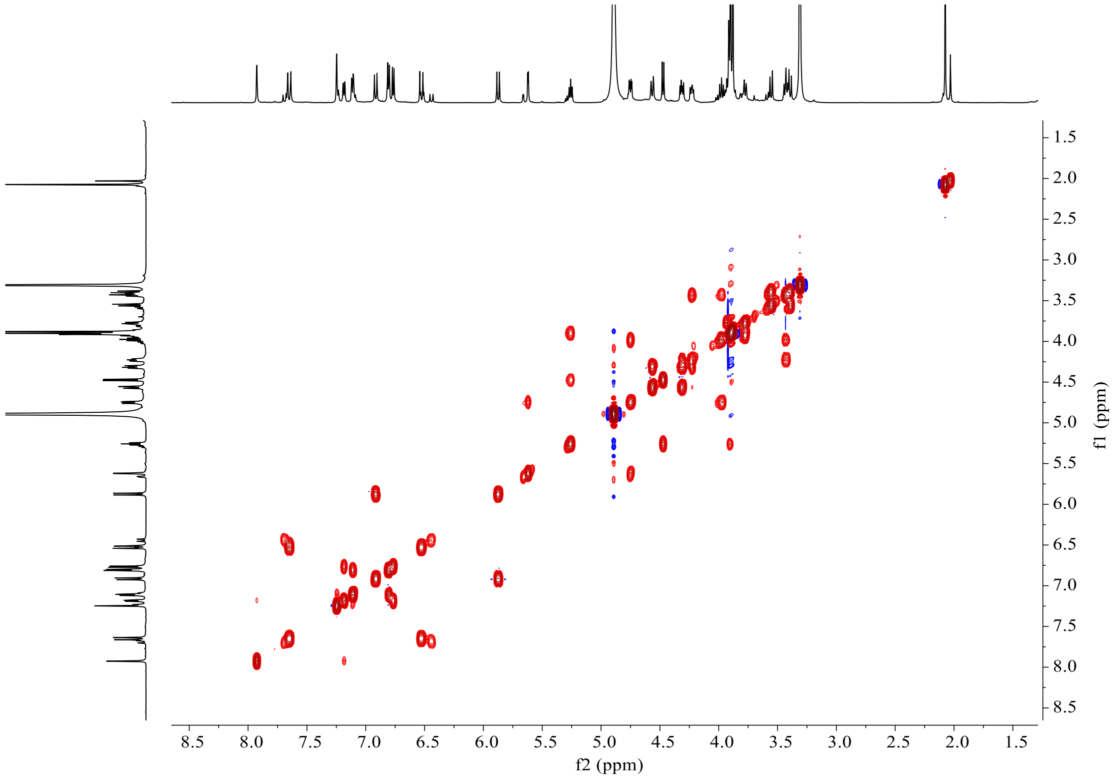

Figure S160. ^{1}H–^{1}H COSY Spectrum of Compound **18** in MeOH-d_4

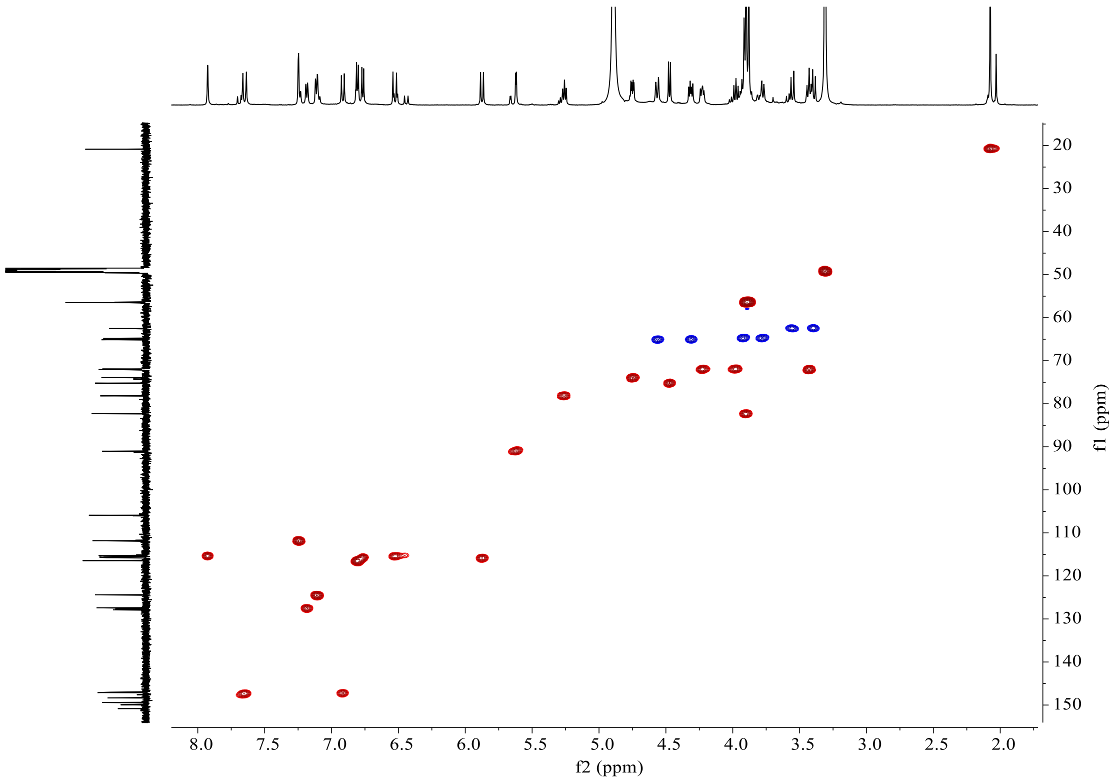

Figure S161. HSQC Spectrum of Compound **18** in MeOH-d_4

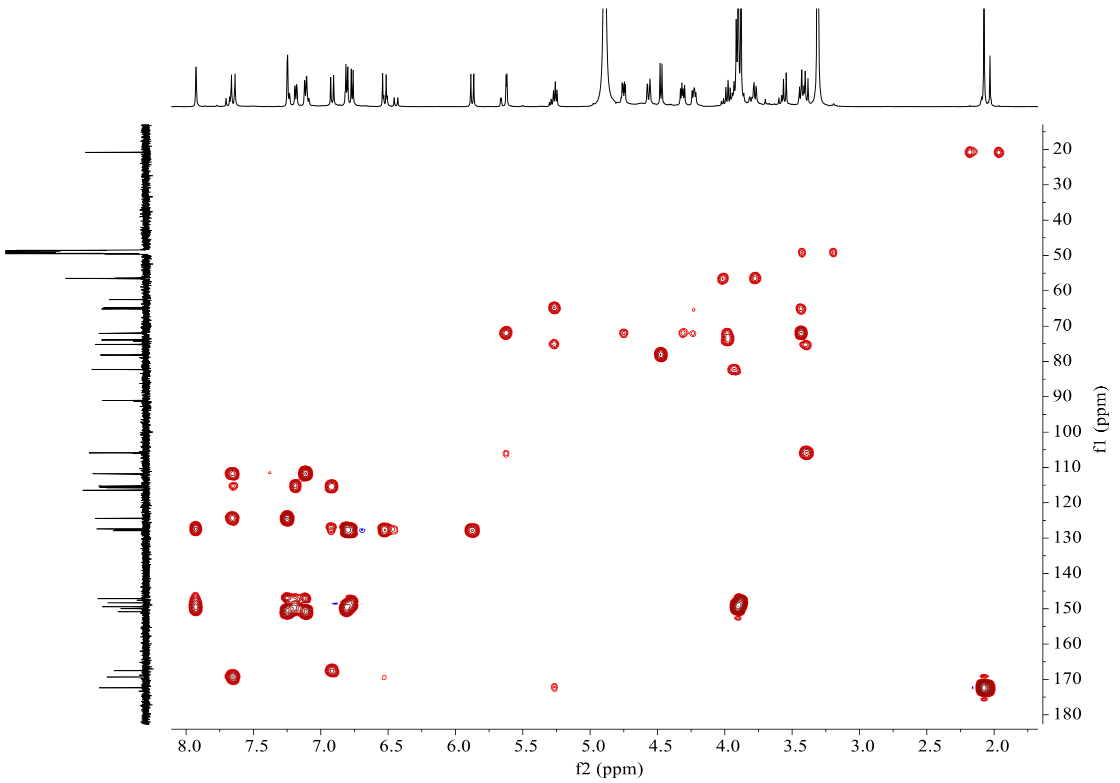

Figure S162. HMBC Spectrum of Compound **18** in MeOH-d_4

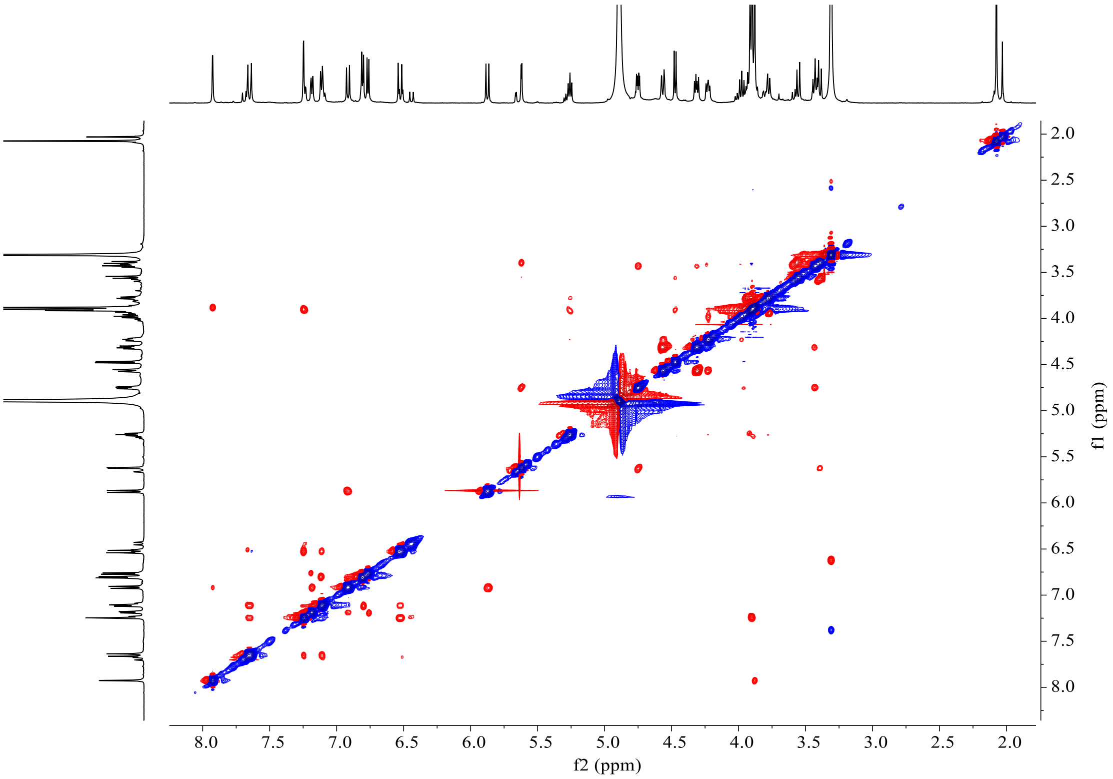

Figure S163. NOESY Spectrum of Compound **18** in MeOH-d_4

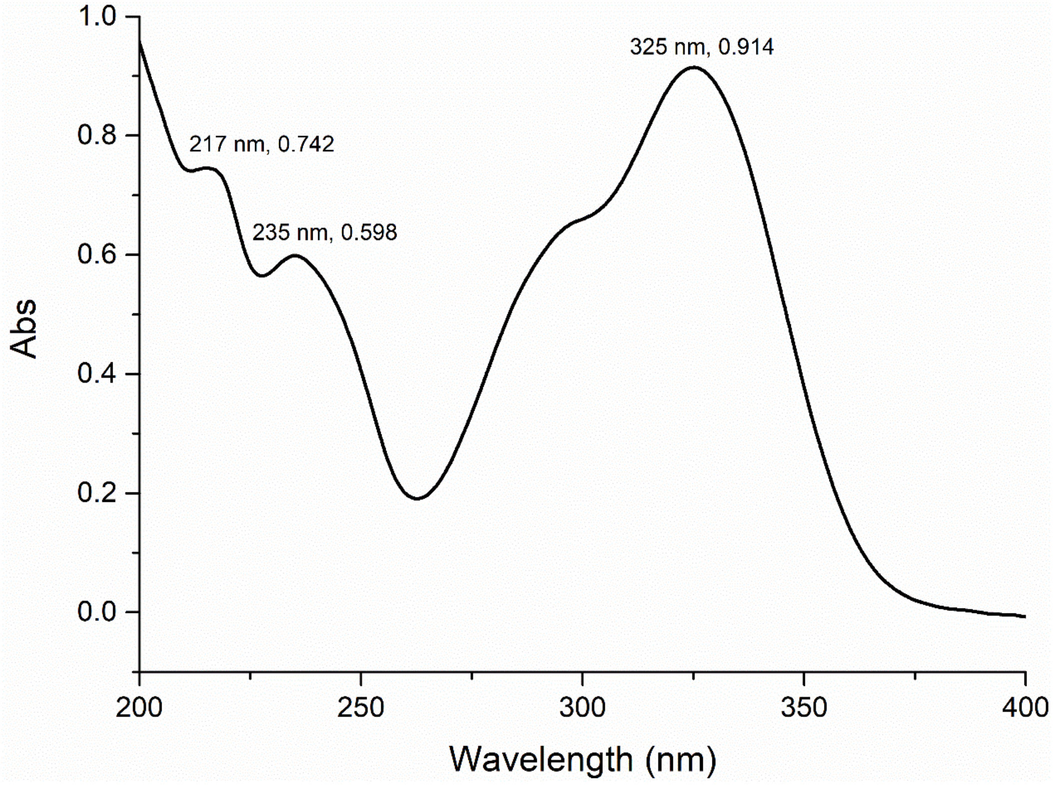

Figure S164. UV Spectrum of Compound **19**

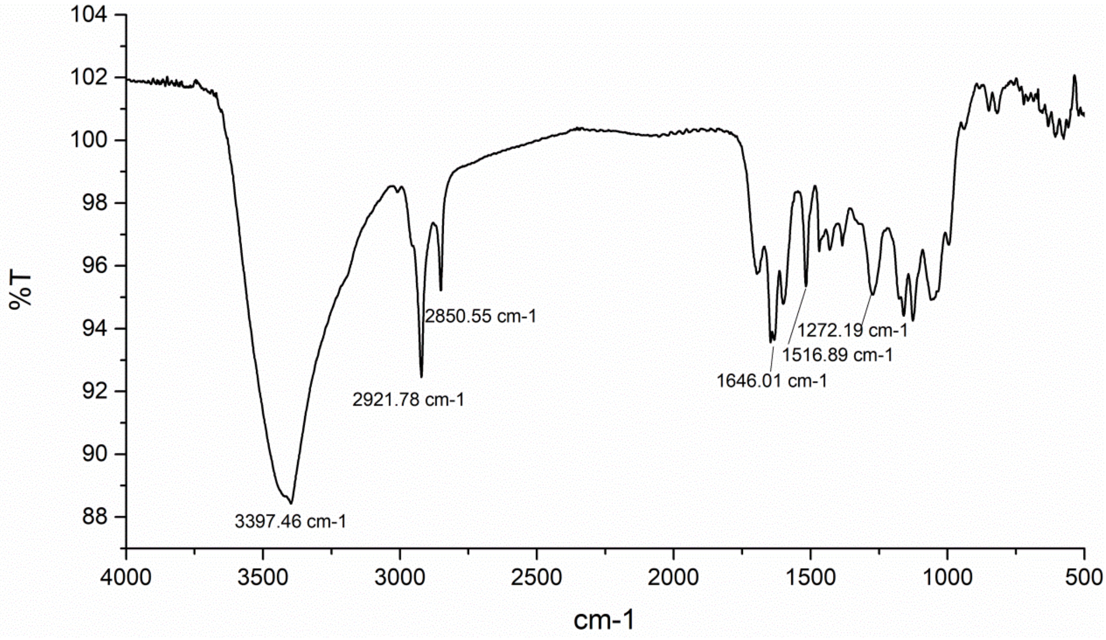

Figure S165. IR (KBr disc) Spectrum of Compound **19**

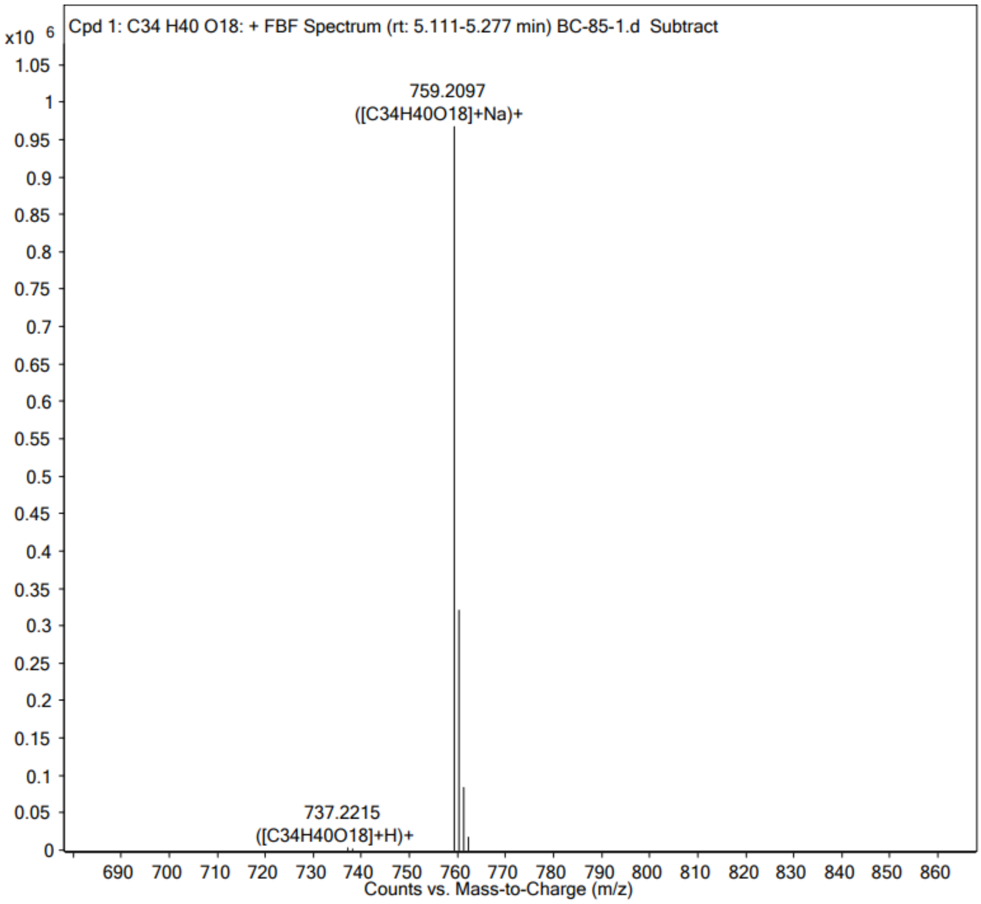

Figure S166. (+) HRESIMS Spectrum of Compound **19**

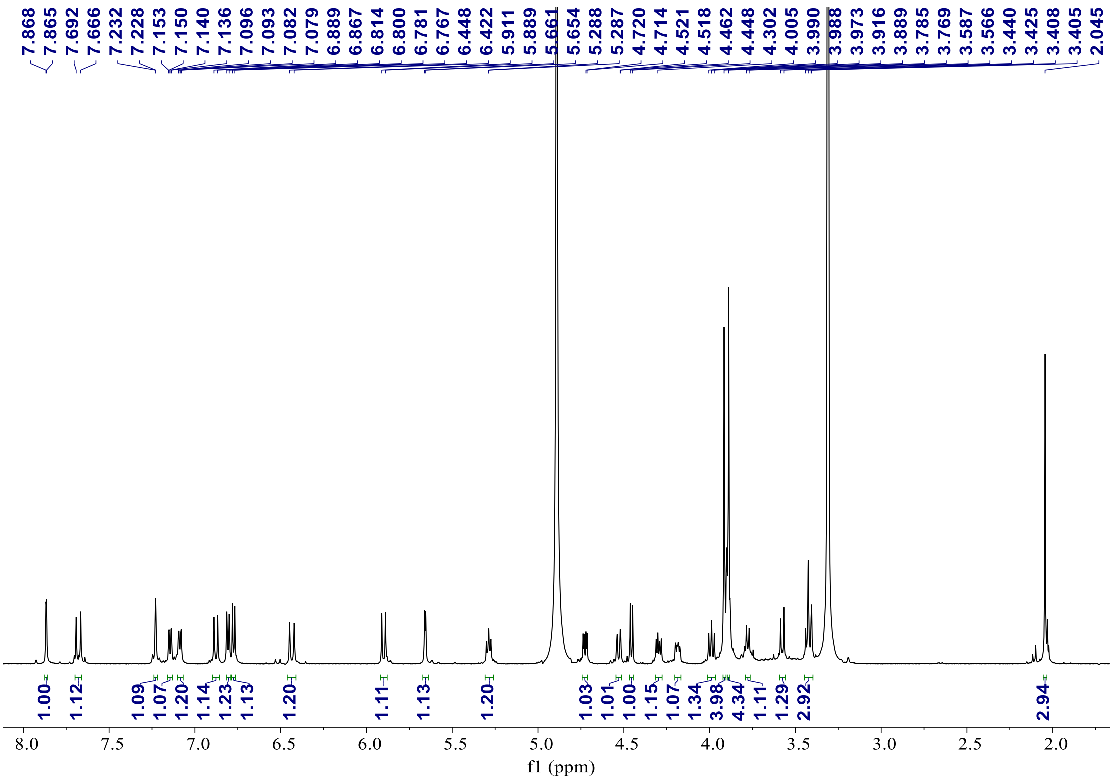

Figure S167. ^{1}H NMR Spectrum of Compound **19** in MeOH-d_4

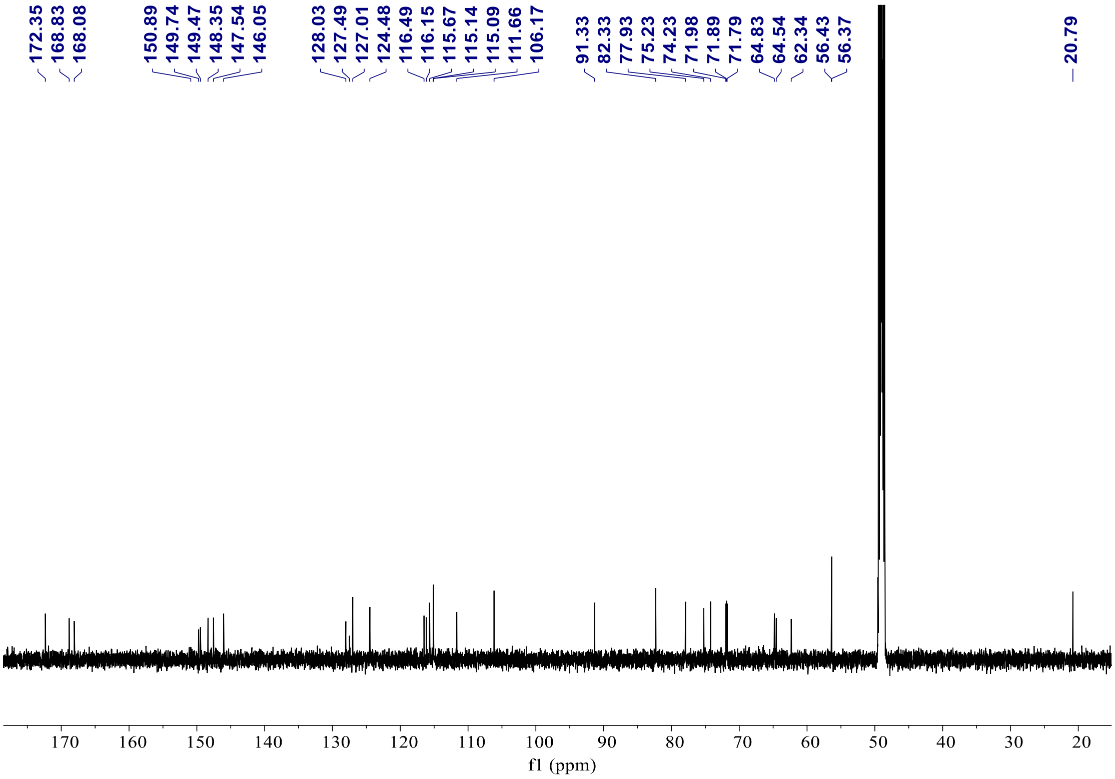

Figure S168. ^{13}C NMR Spectrum of Compound **19** in MeOH-d_4

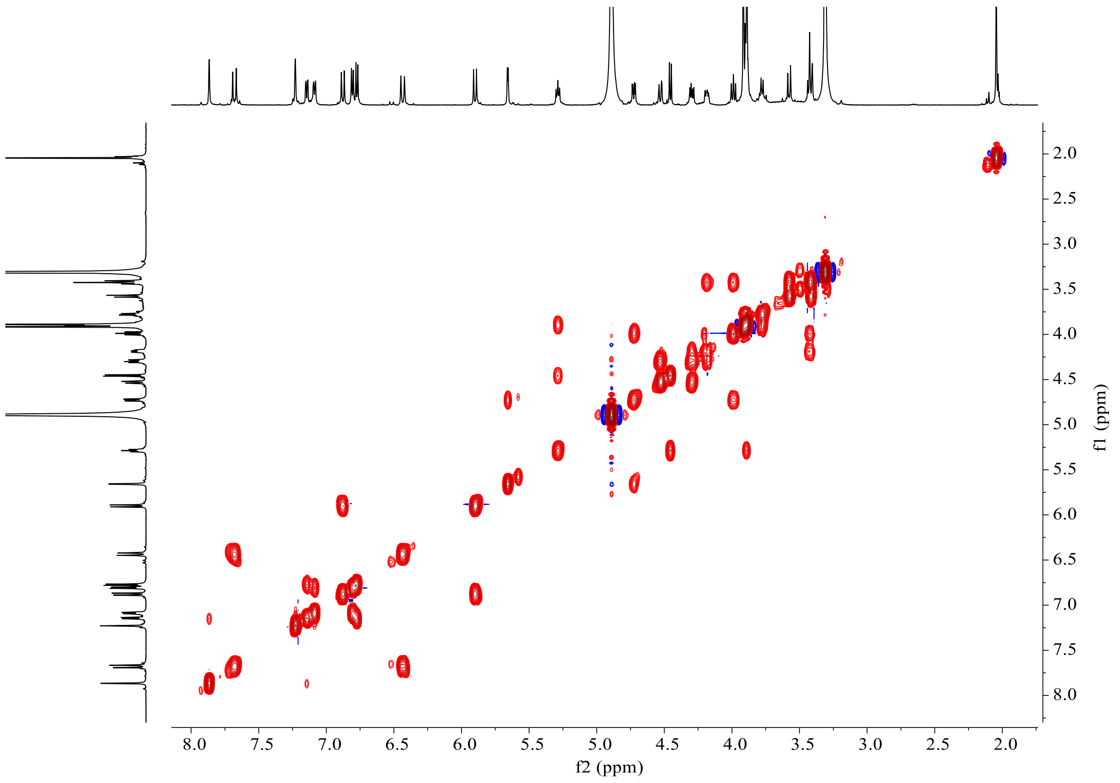

Figure S169. $^{1}H–^{1}H$ COSY Spectrum of Compound **19** in MeOH-d_4

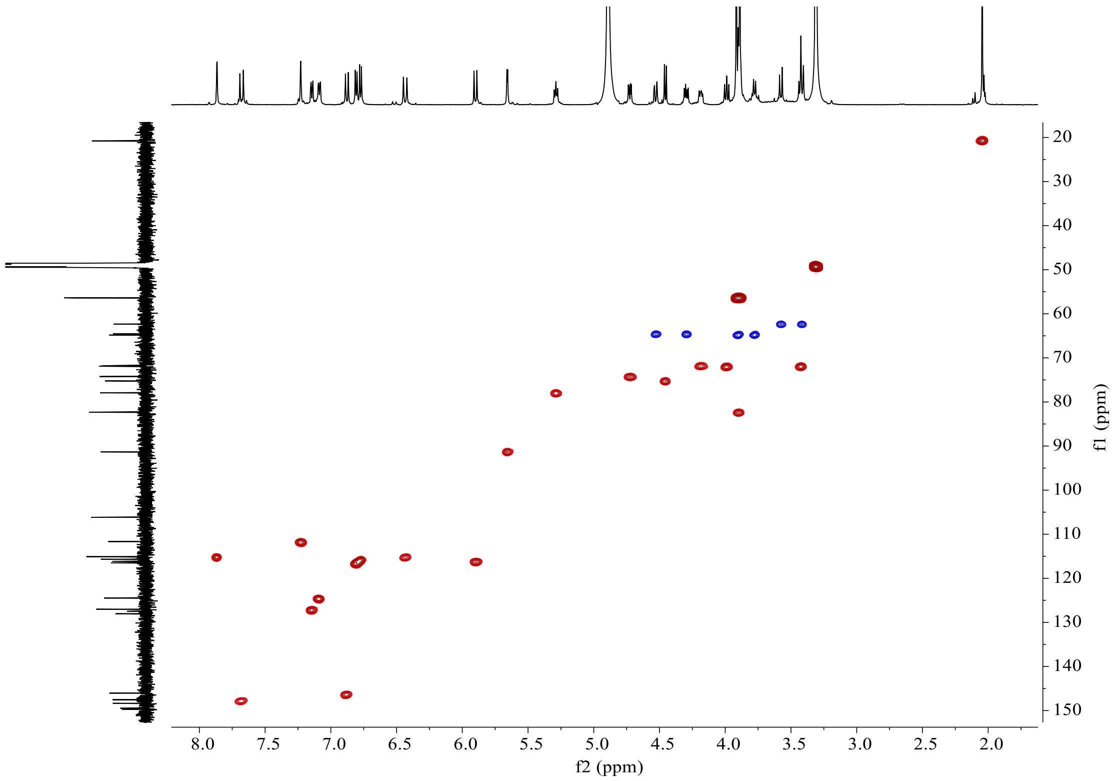

Figure S170. HSQC Spectrum of Compound **19** in MeOH-d_4

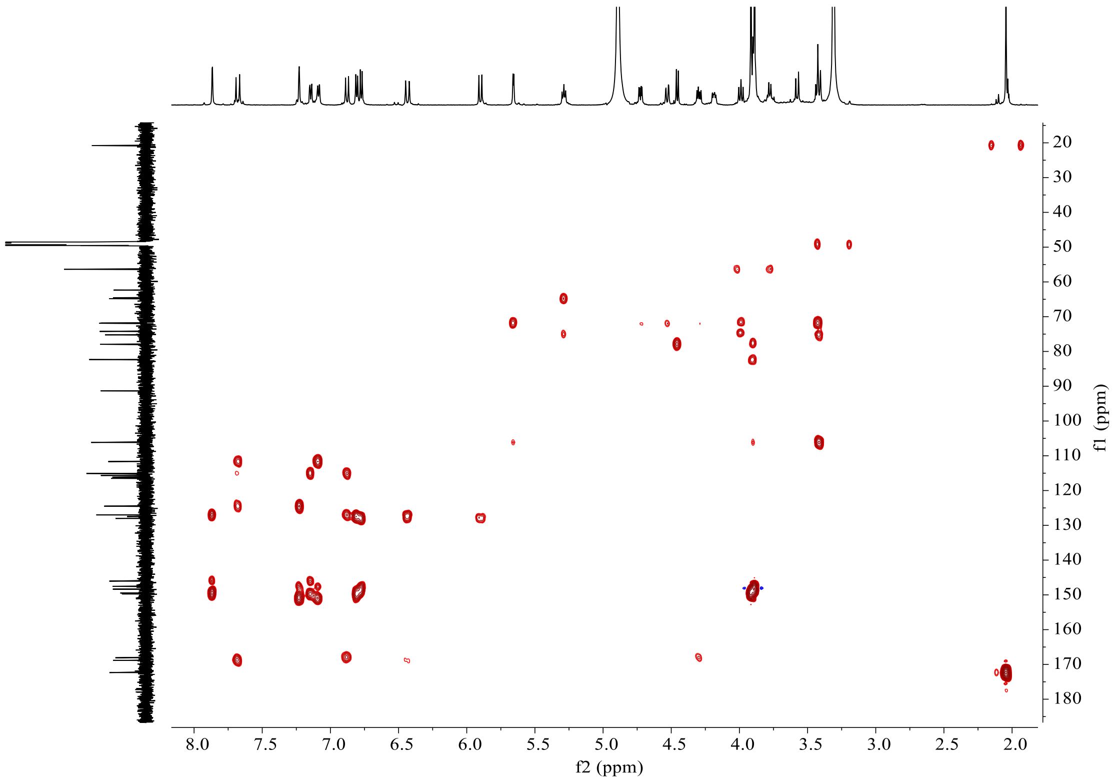

Figure S171. HMBC Spectrum of Compound **19** in MeOH-d_4

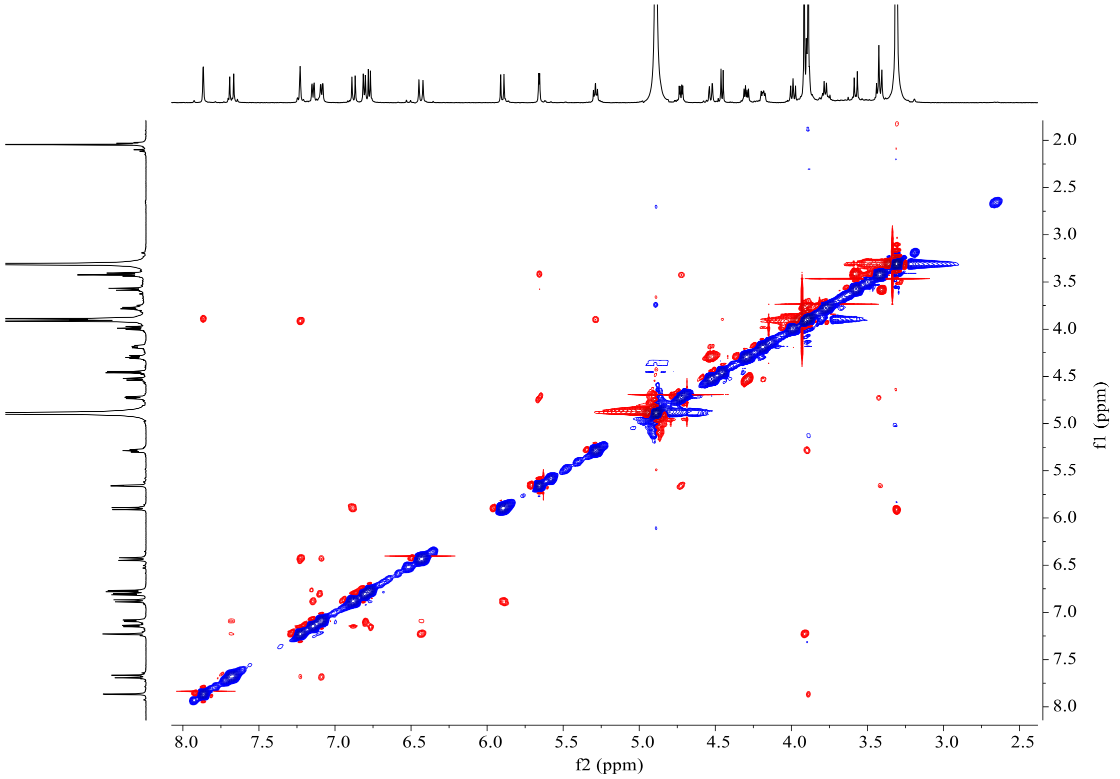

Figure S172. NOESY Spectrum of Compound **19** in MeOH-d_4

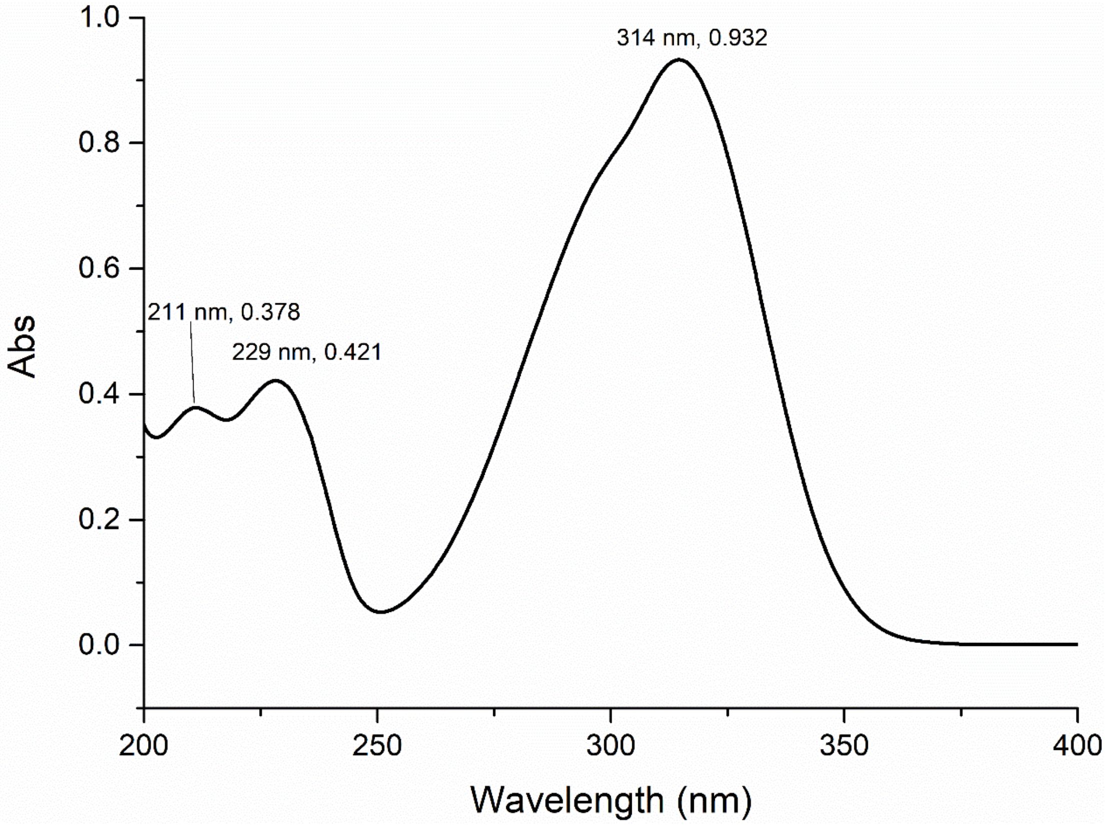

Figure S173. UV Spectrum of Compound **20**

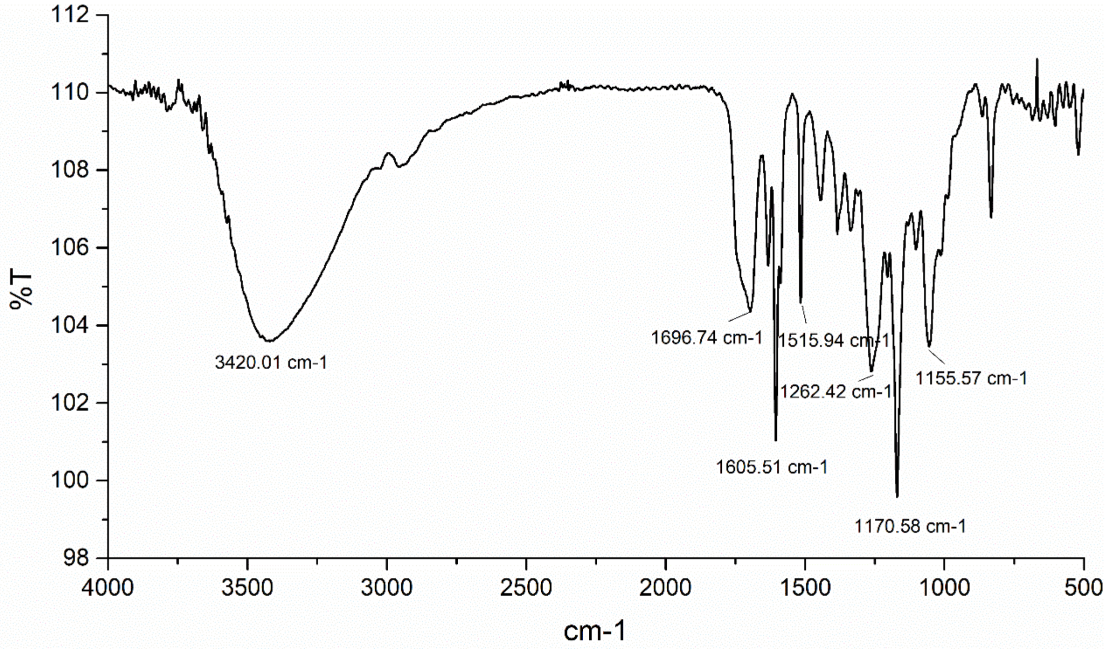

Figure S174. IR (KBr disc) Spectrum of Compound **20**

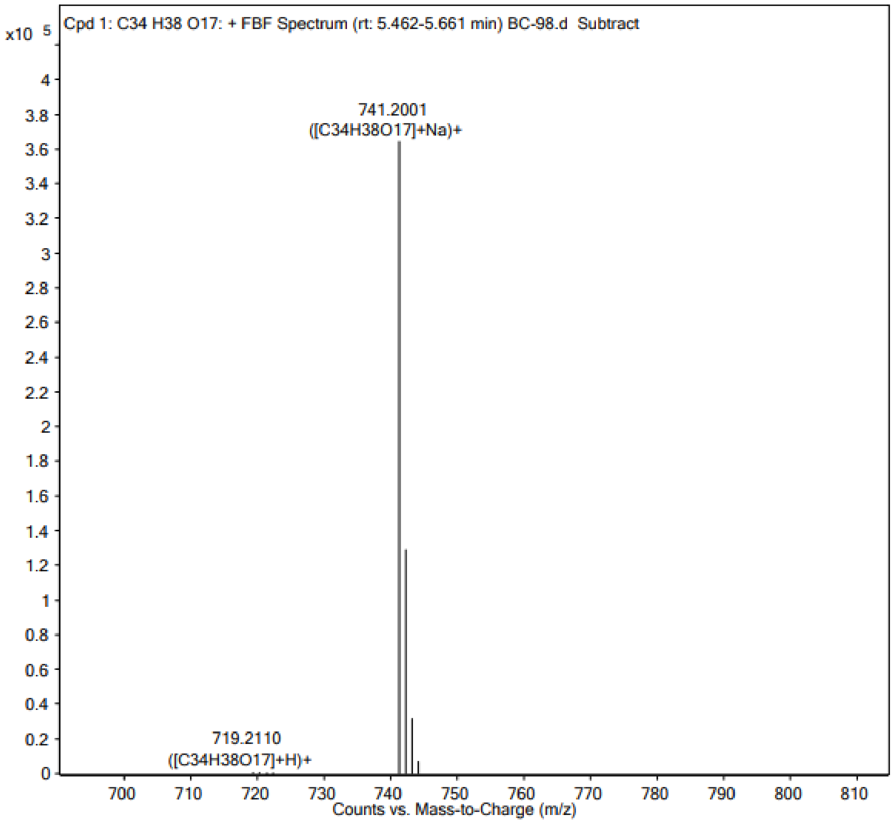

Figure S175. (+) HRESIMS Spectrum of Compound **20**

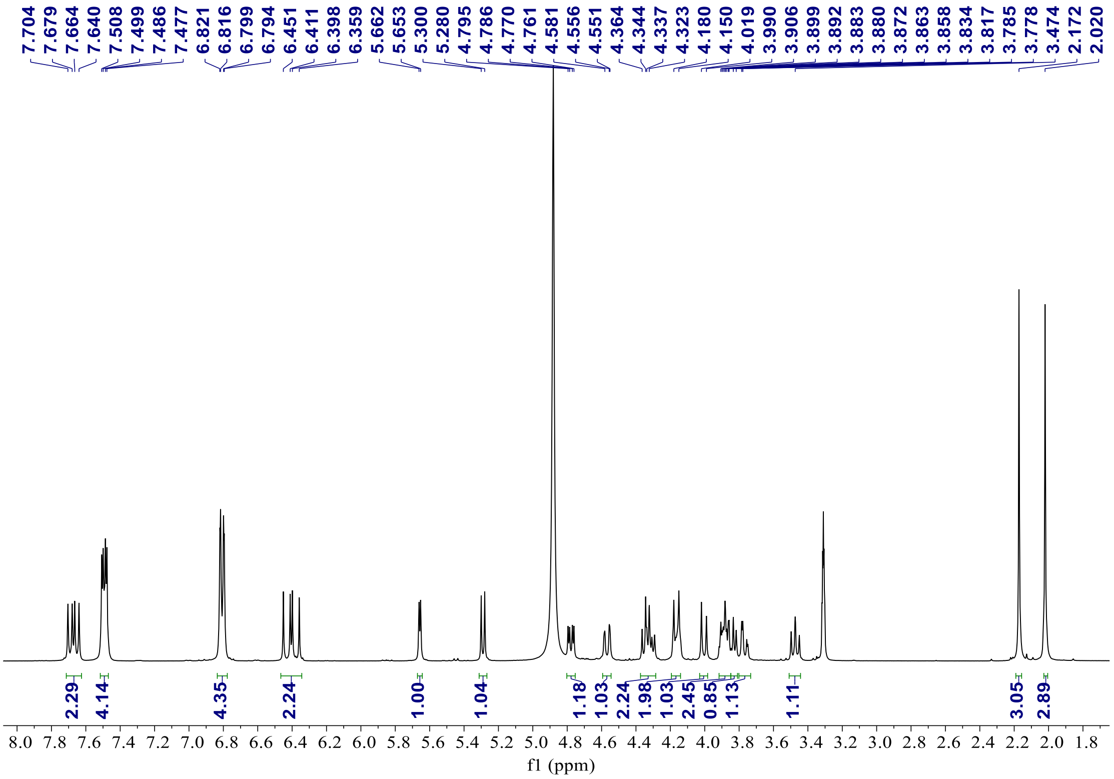

Figure S176. ^{1}H NMR Spectrum of Compound **20** in MeOH-d_4

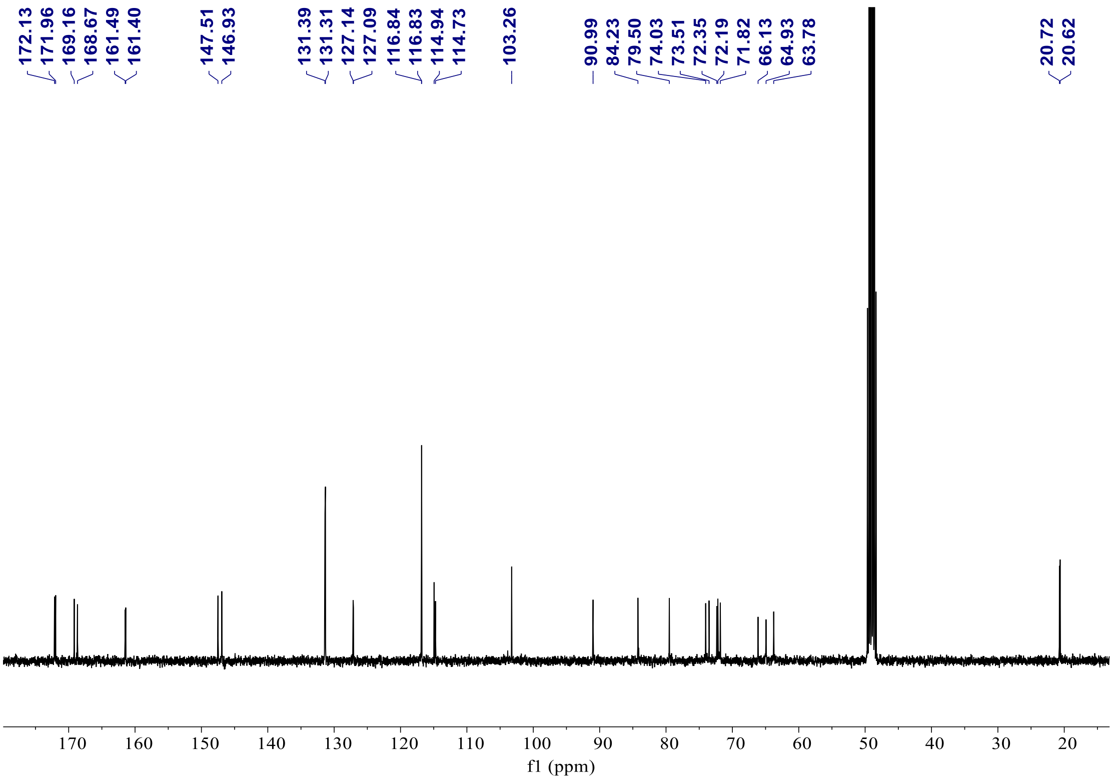

Figure S177. ^{13}C NMR Spectrum of Compound **20** in MeOH-d_4

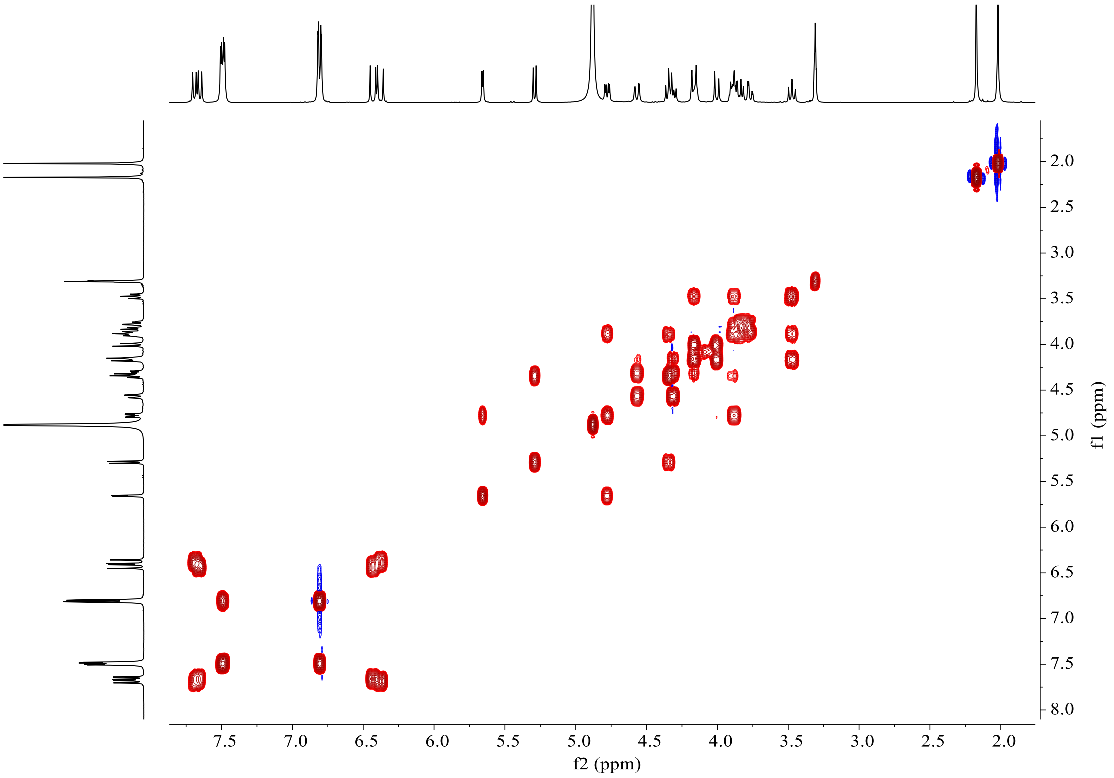

Figure S178. ^{1}H–^{1}H COSY Spectrum of Compound **20** in MeOH-d_4

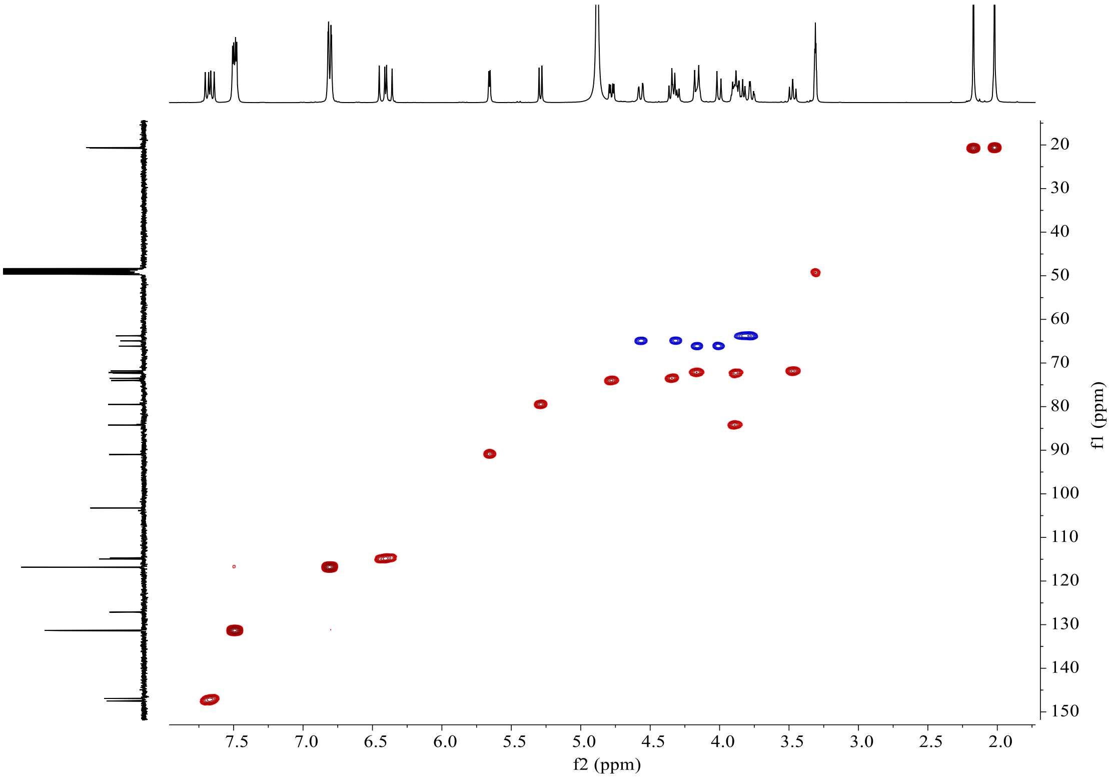

Figure S179. HSQC Spectrum of Compound **20** in MeOH-d_4

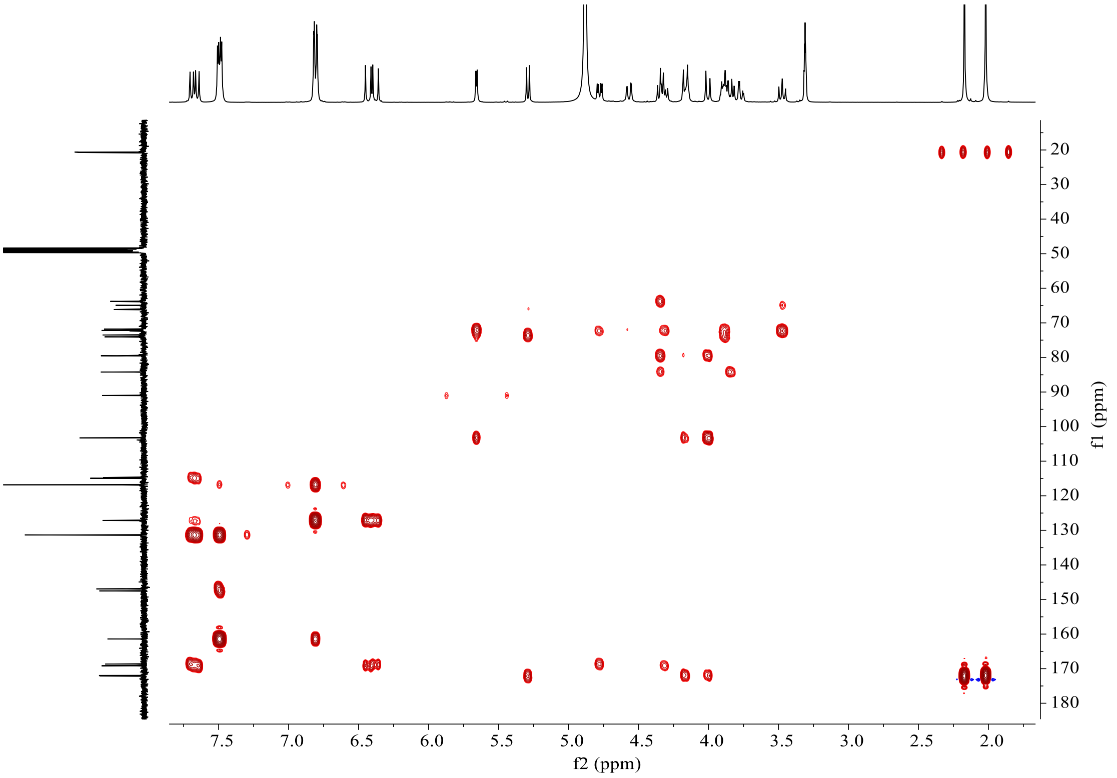

Figure S180. HMBC Spectrum of Compound **20** in MeOH-d_4

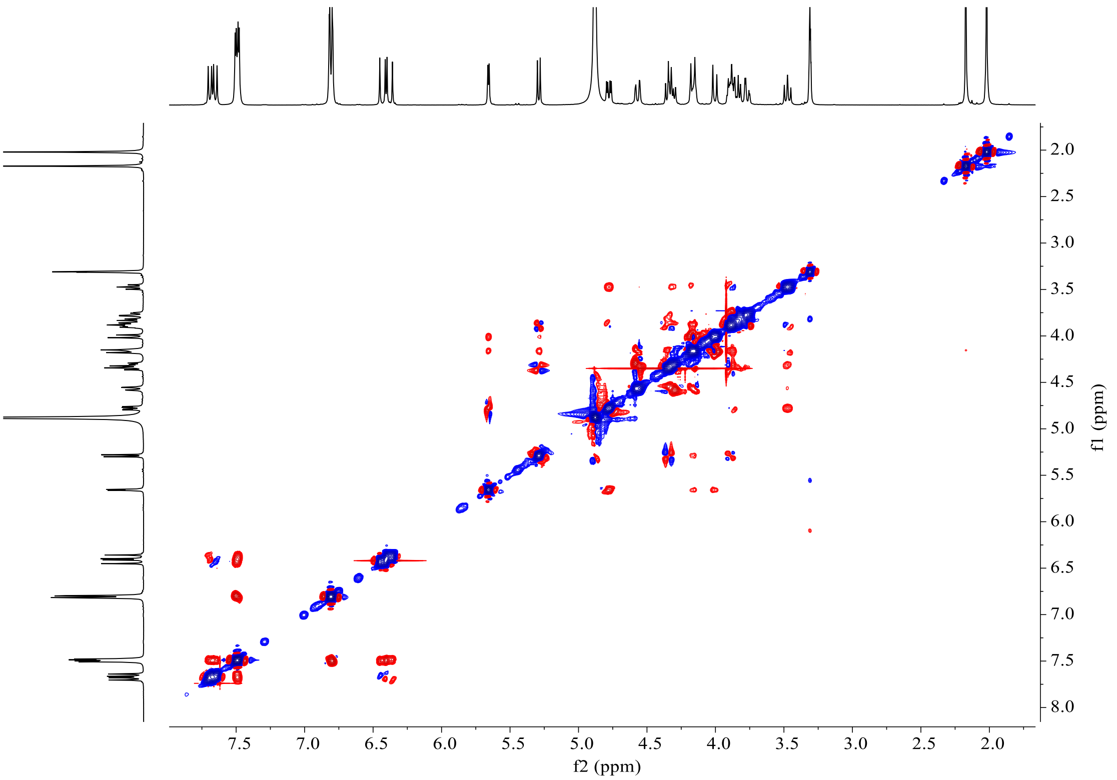

Figure S181. NOESY Spectrum of Compound **20** in MeOH-d_4

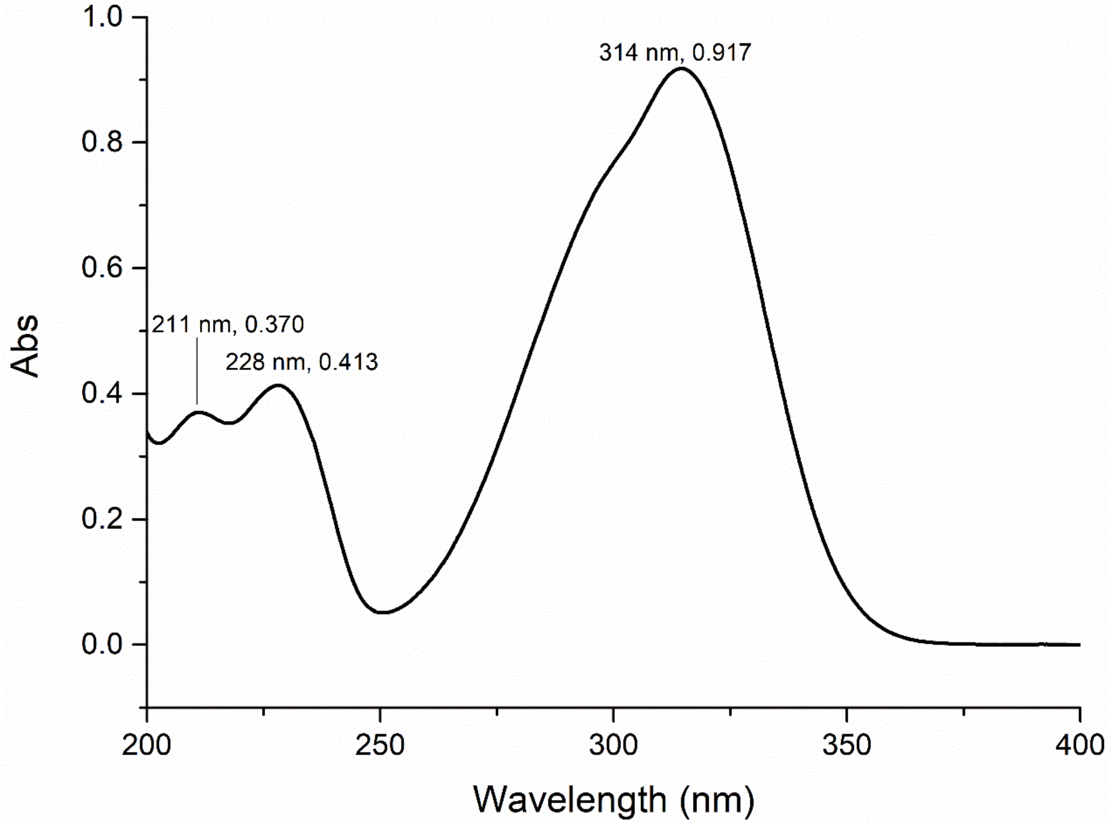

Figure S182. UV Spectrum of Compound **21**

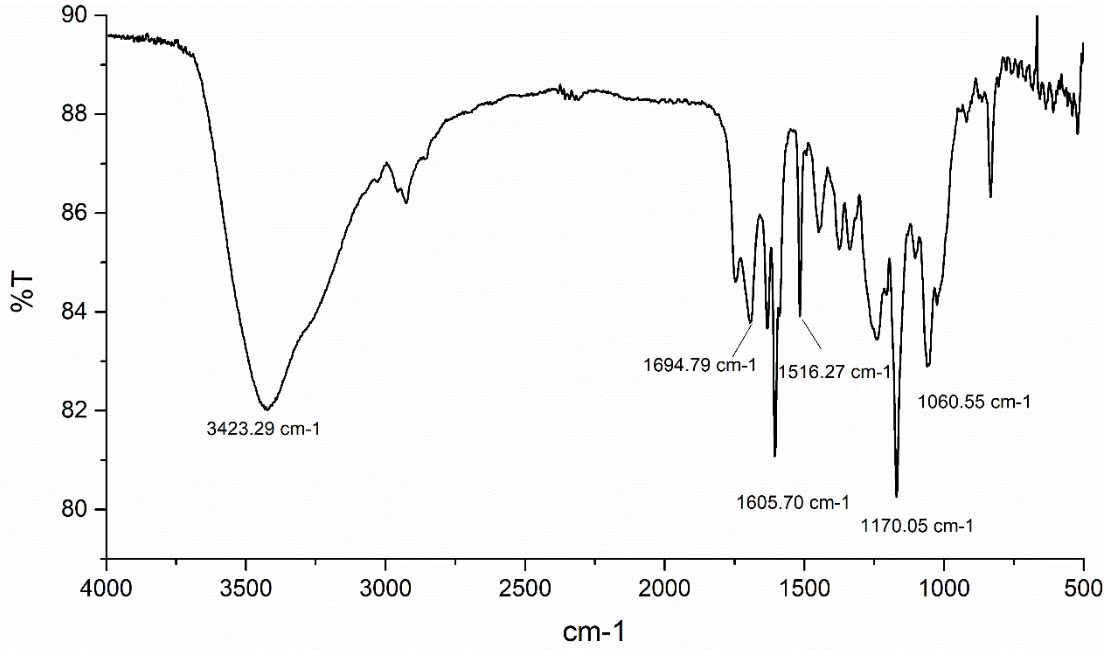

Figure S183. IR (KBr disc) Spectrum of Compound **21**

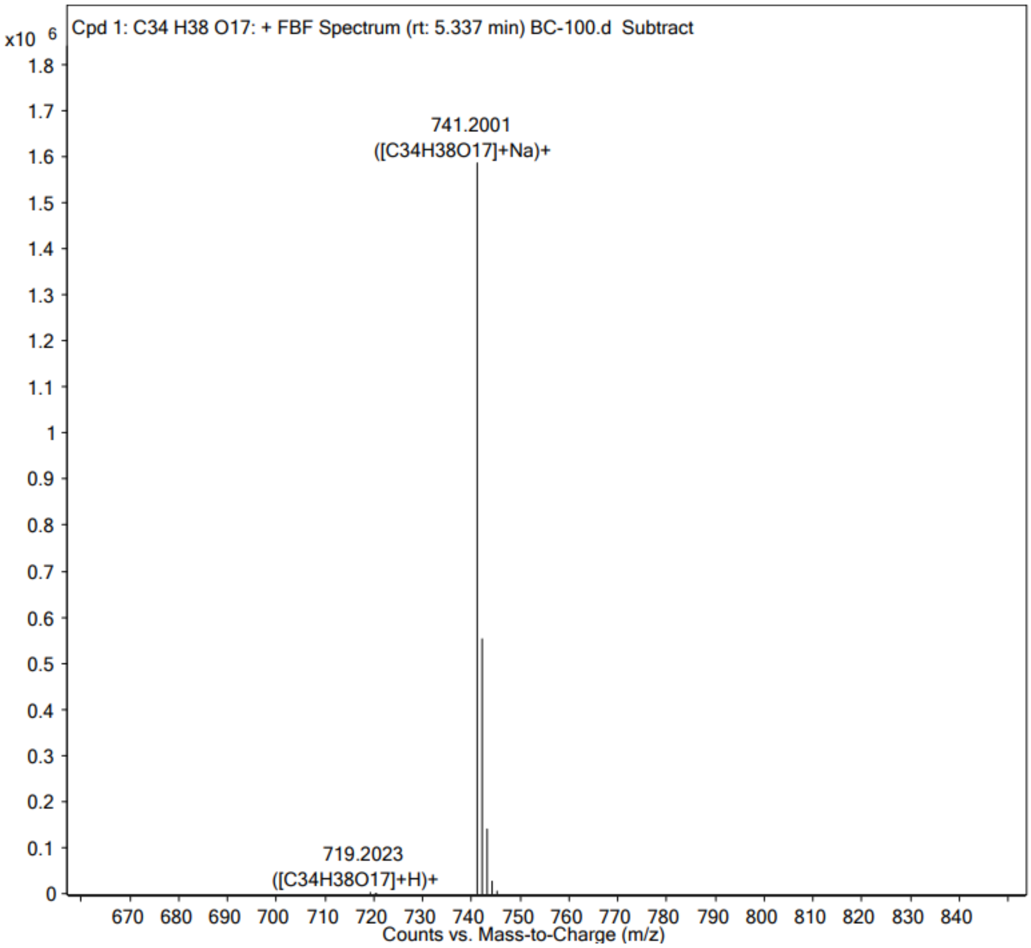

Figure S184. (+) HRESIMS Spectrum of Compound **21**

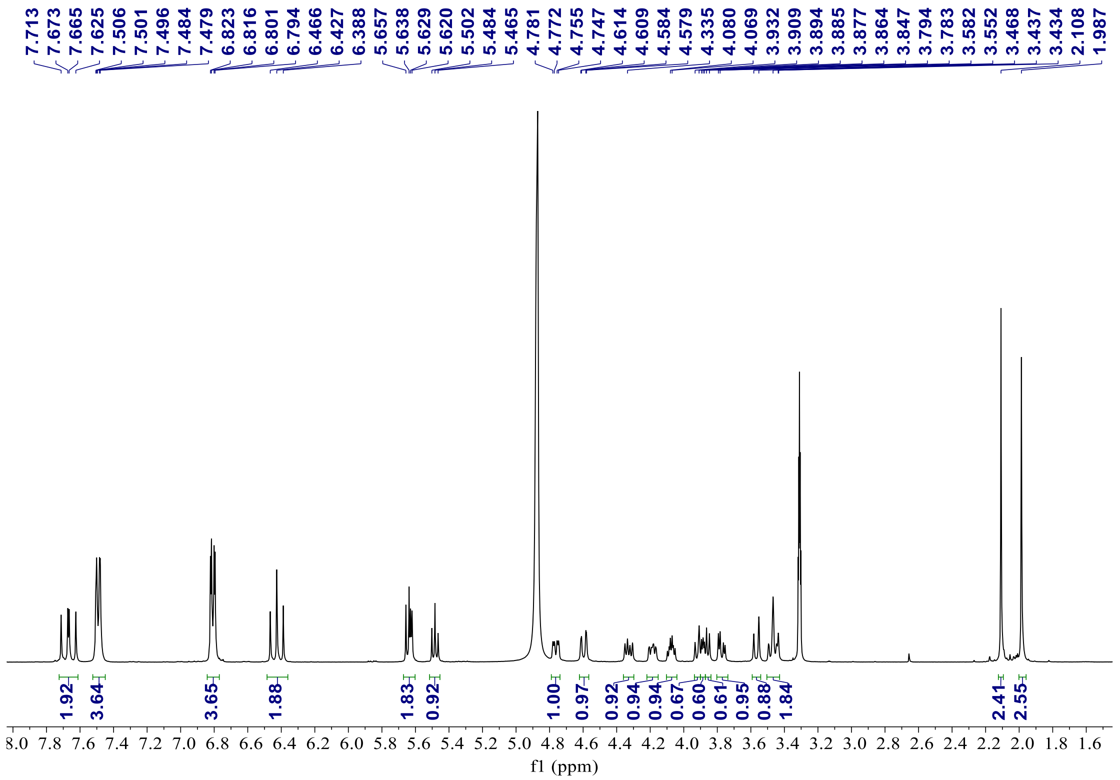

Figure S185. ^{1}H NMR Spectrum of Compound **21** in MeOH-d_4

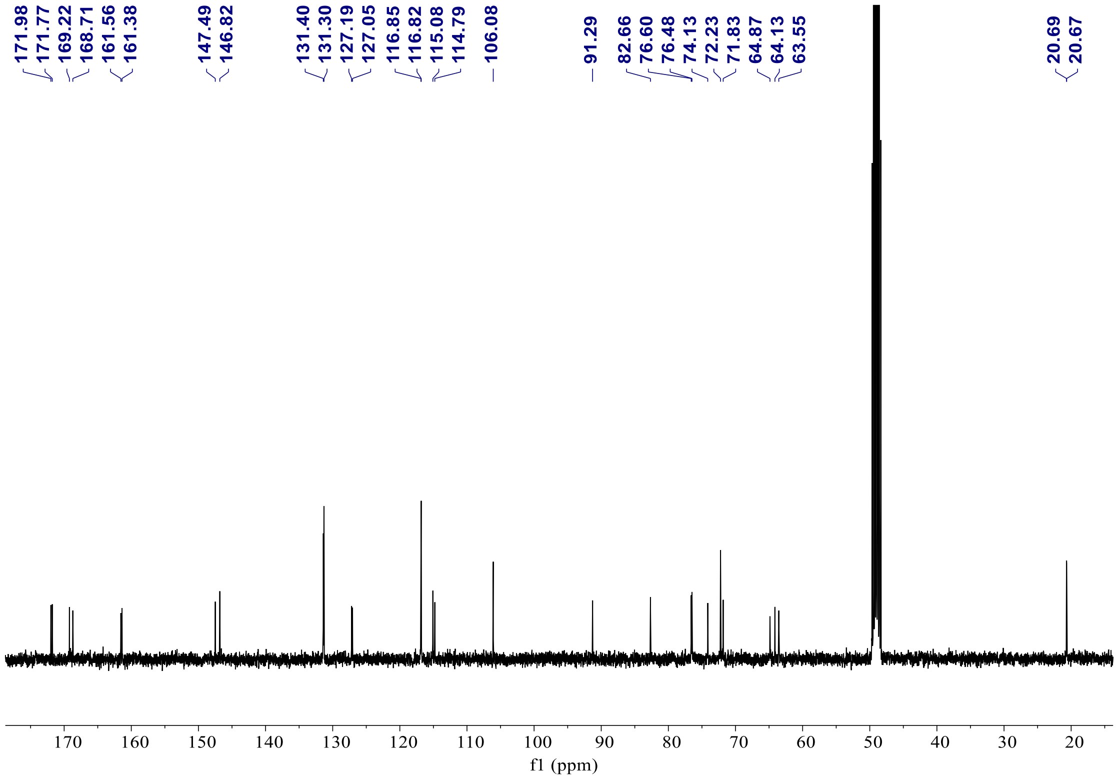

Figure S186. ^{13}C NMR Spectrum of Compound **21** in MeOH-d_4

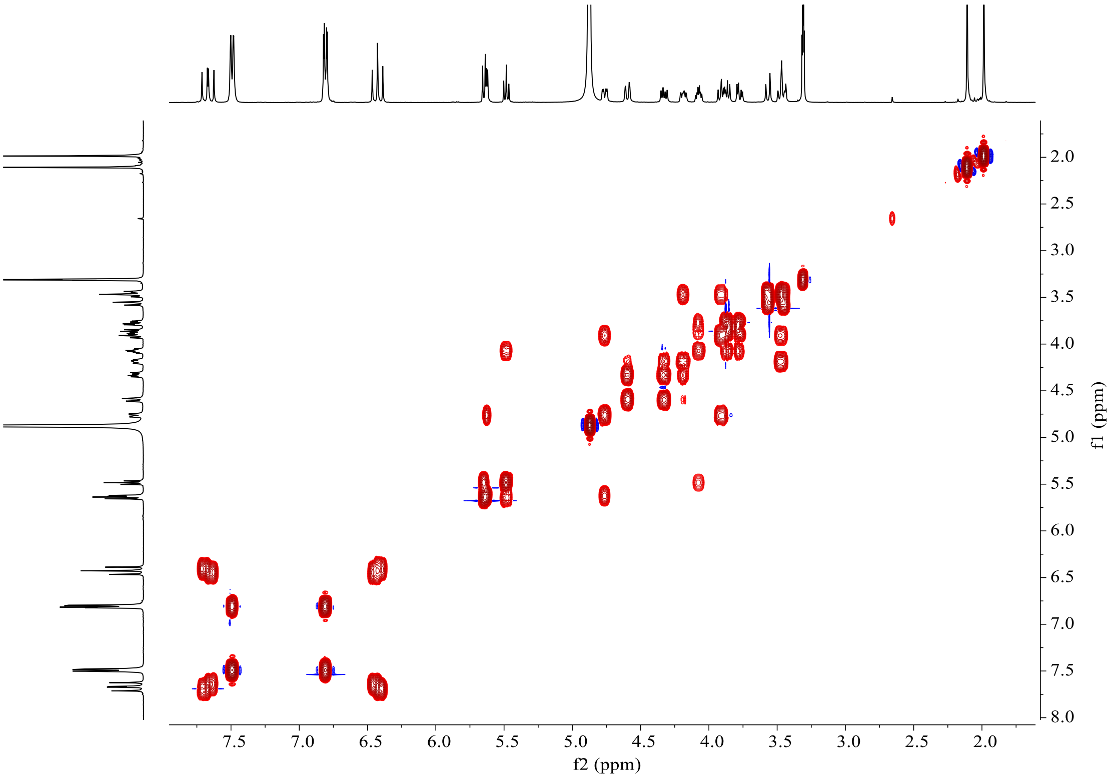

Figure S187. ^{1}H–^{1}H COSY Spectrum of Compound **21** in MeOH-d_4

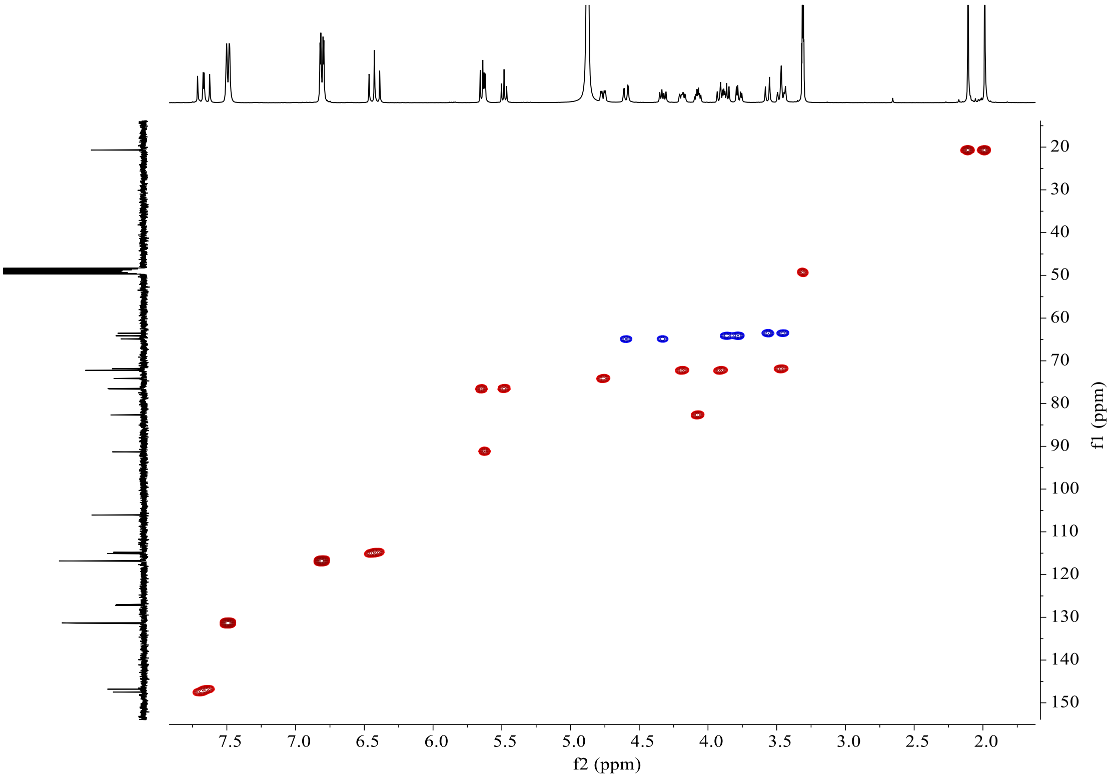

Figure S188. HSQC Spectrum of Compound **21** in MeOH-d_4

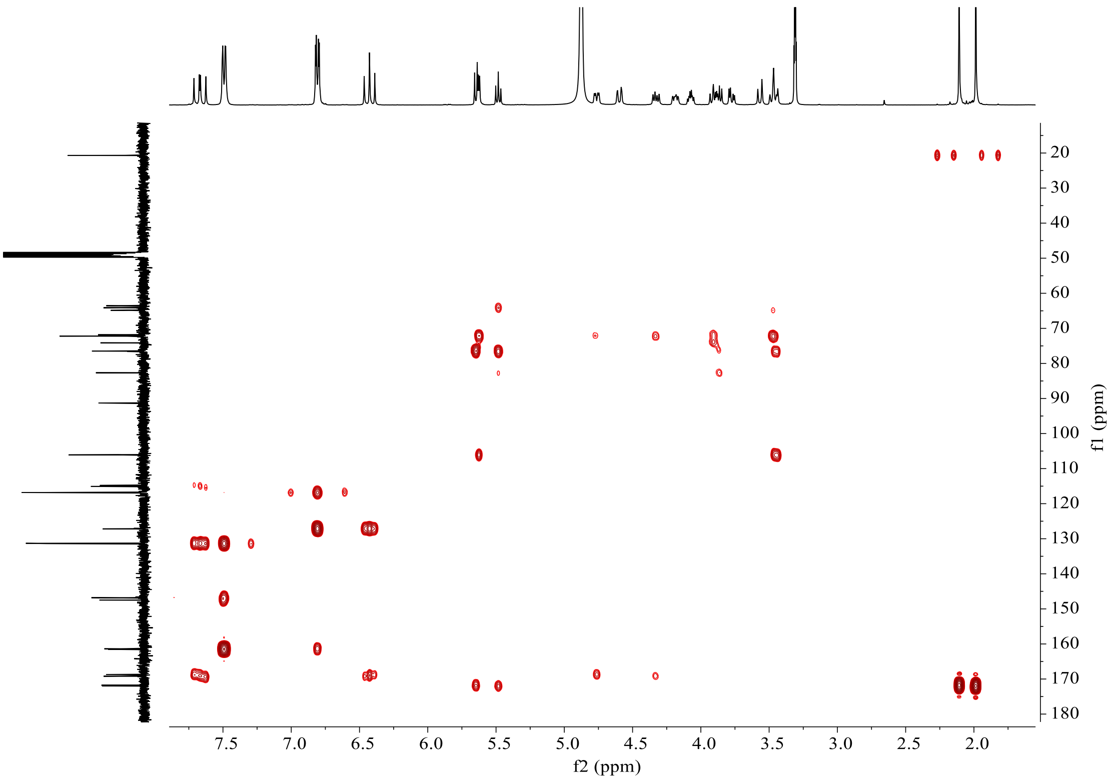

Figure S189. HMBC Spectrum of Compound **21** in MeOH-d_4

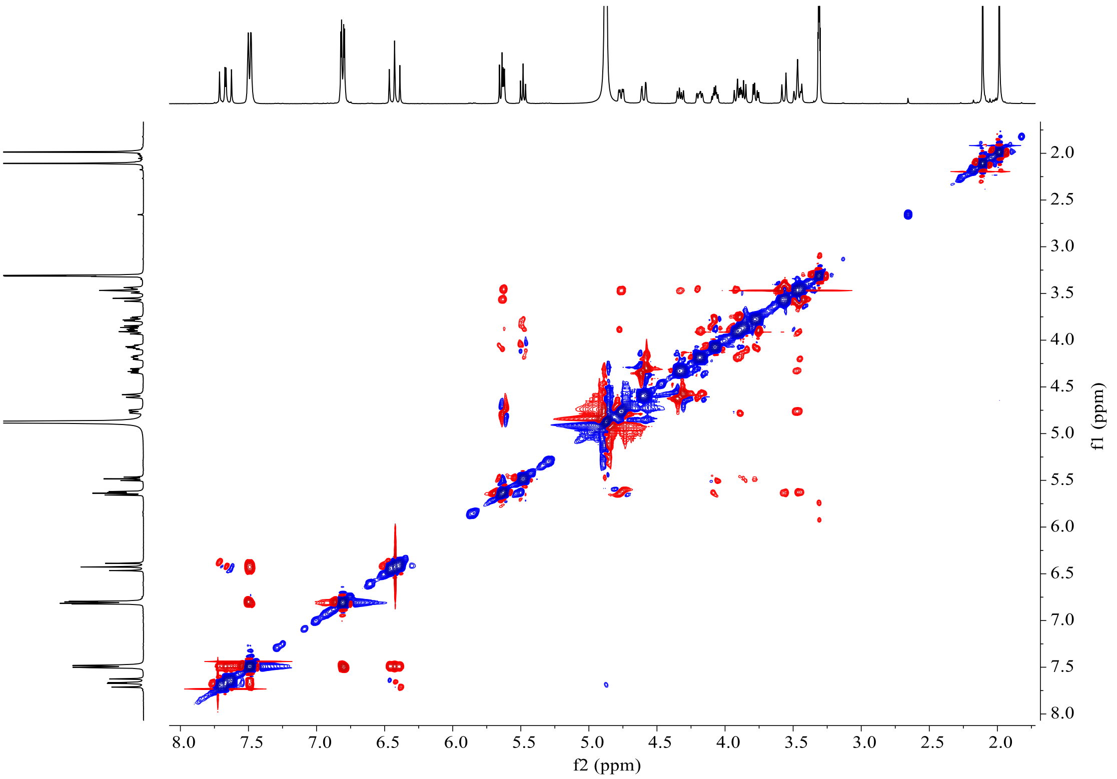

Figure S190. NOESY Spectrum of Compound **21** in MeOH-d_4

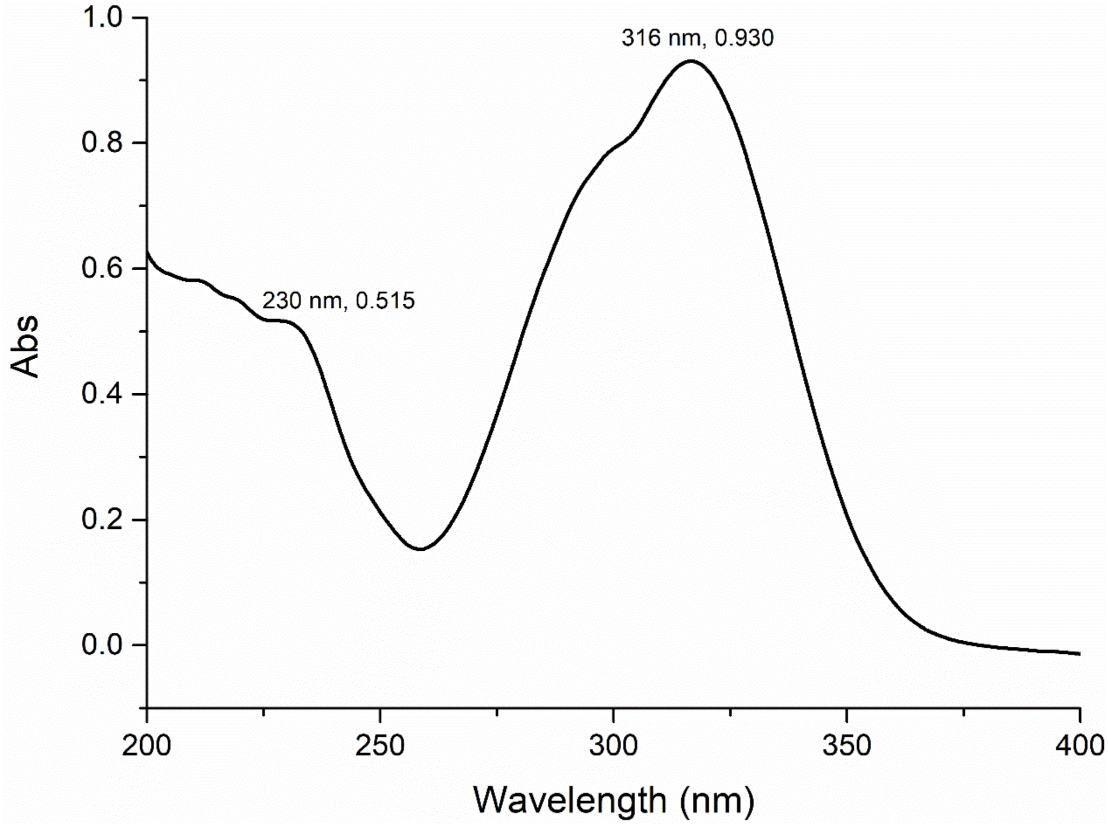

Figure S191. UV Spectrum of Compound **22**

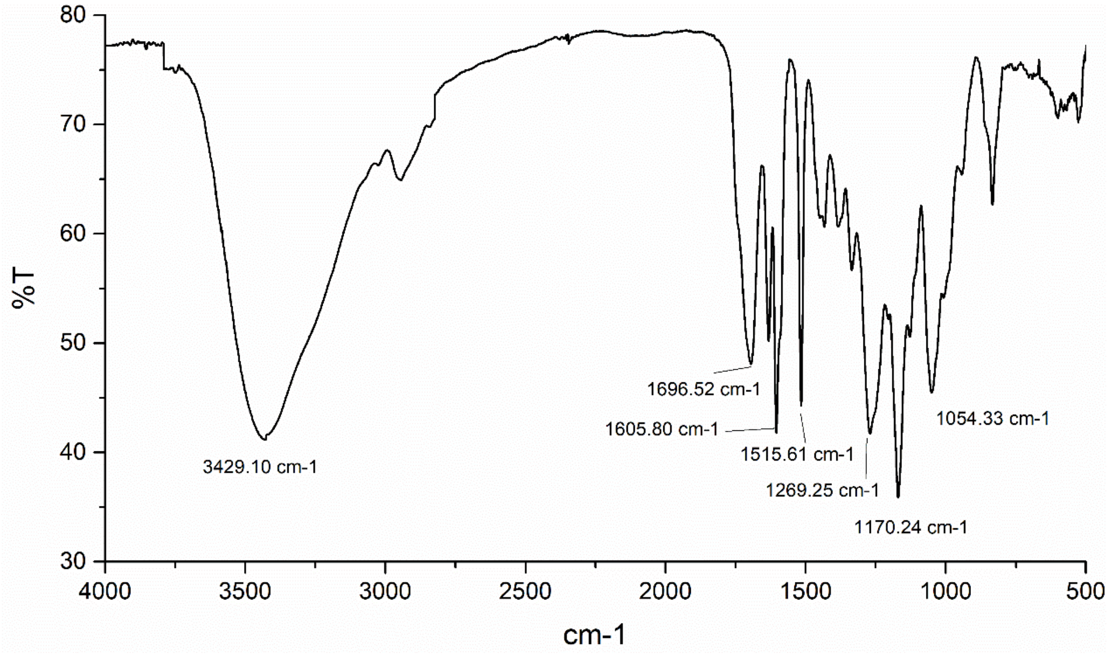

Figure S192. IR (KBr disc) Spectrum of Compound **22**

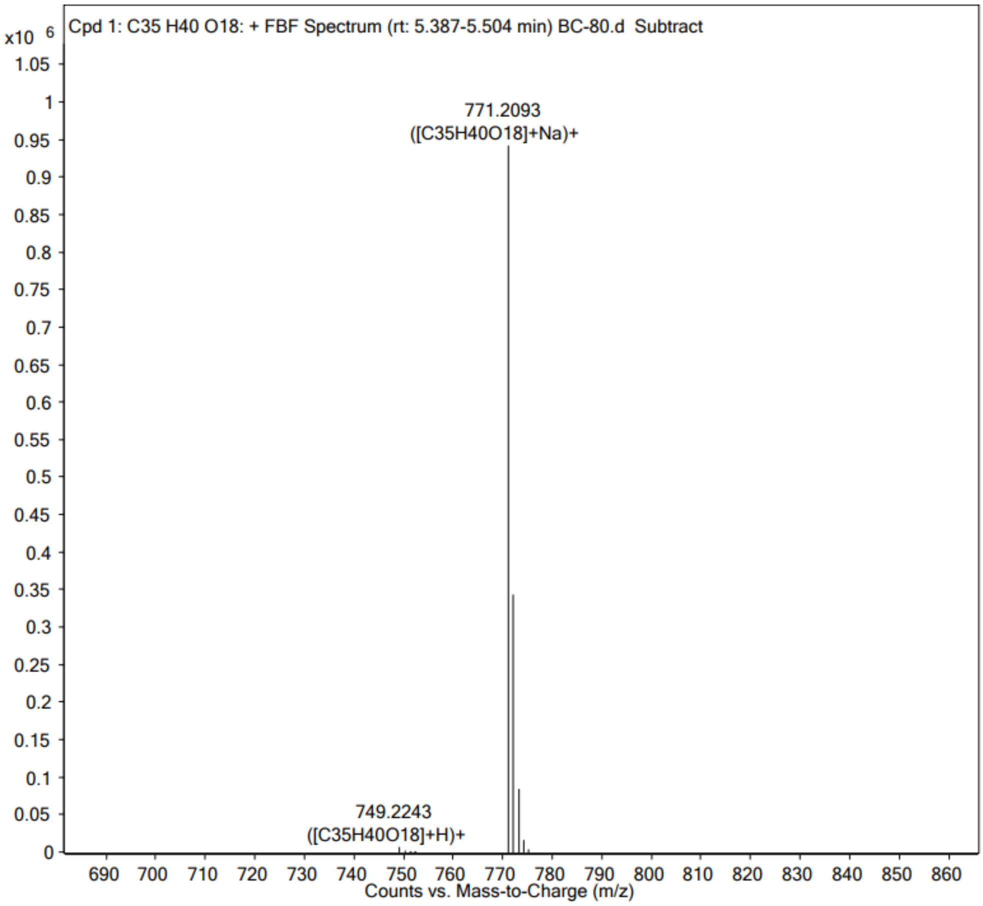

Figure S193. (+) HRESIMS Spectrum of Compound **22**

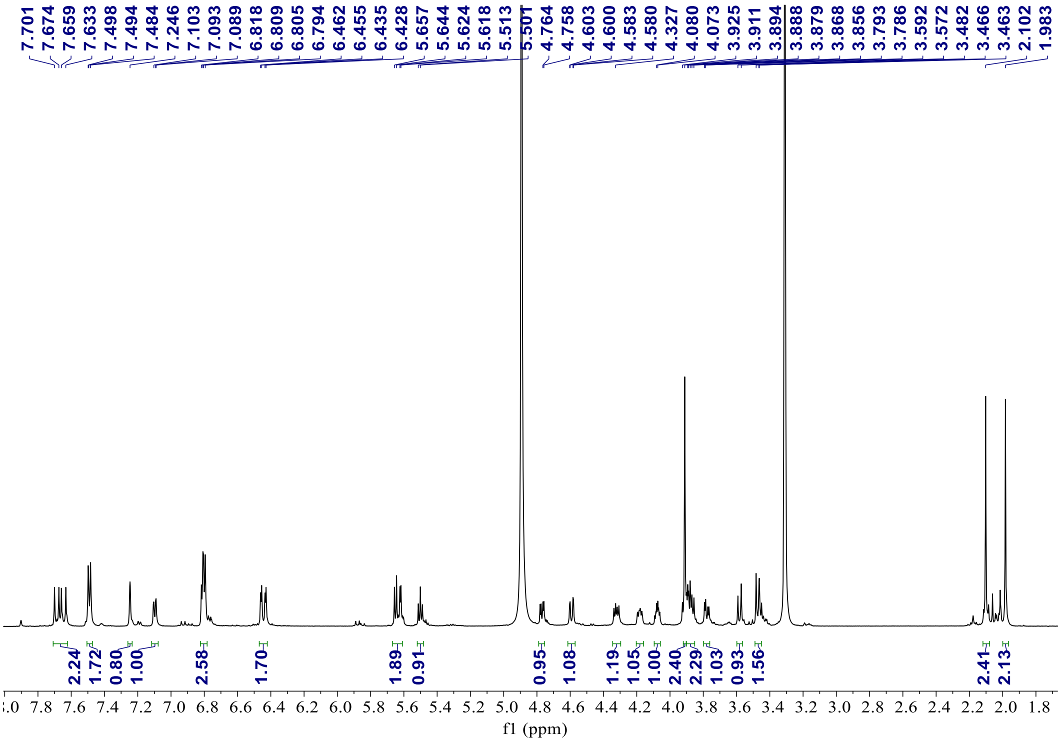

Figure S194. ^{1}H NMR Spectrum of Compound **22** in MeOH-d_4

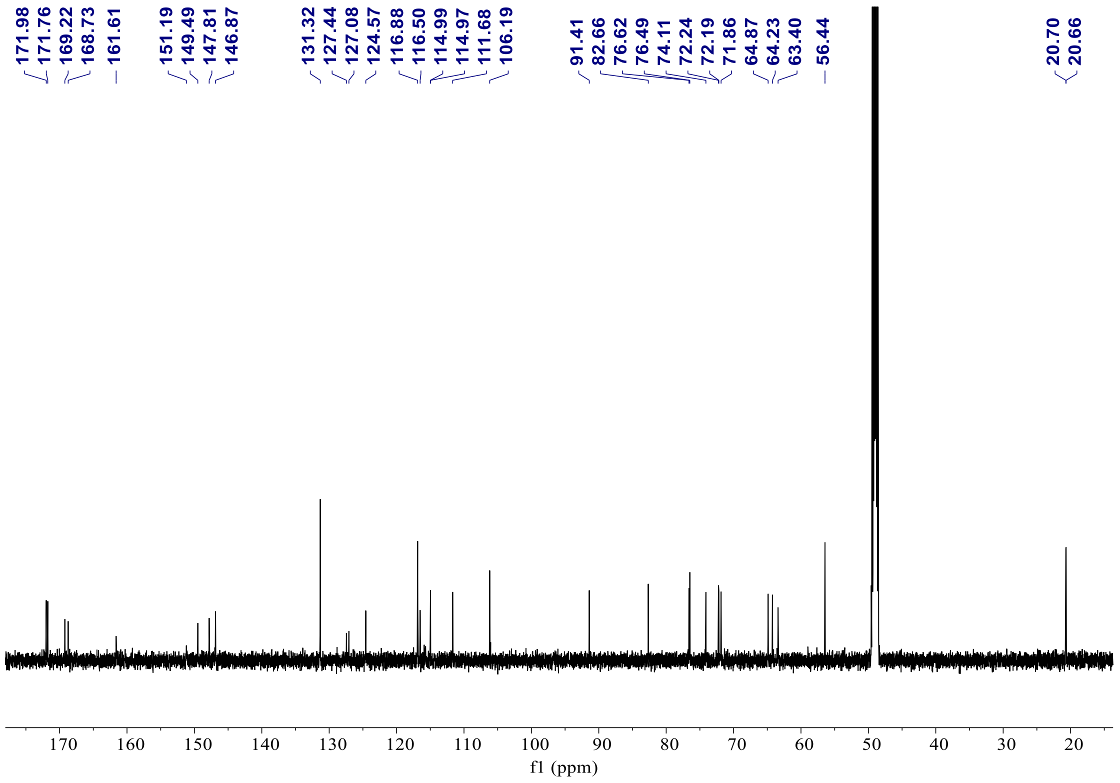

Figure S195. ^{13}C NMR Spectrum of Compound **22** in MeOH-d_4

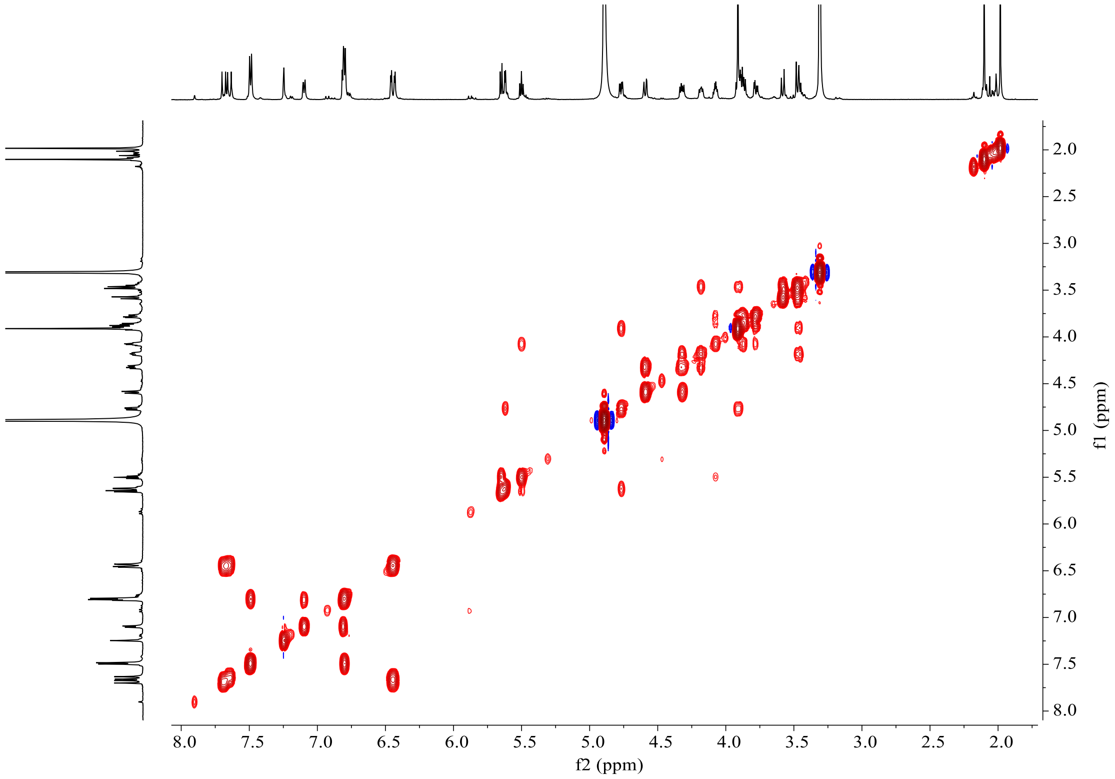

Figure S196. ^{1}H–^{1}H COSY Spectrum of Compound **22** in MeOH-d_4

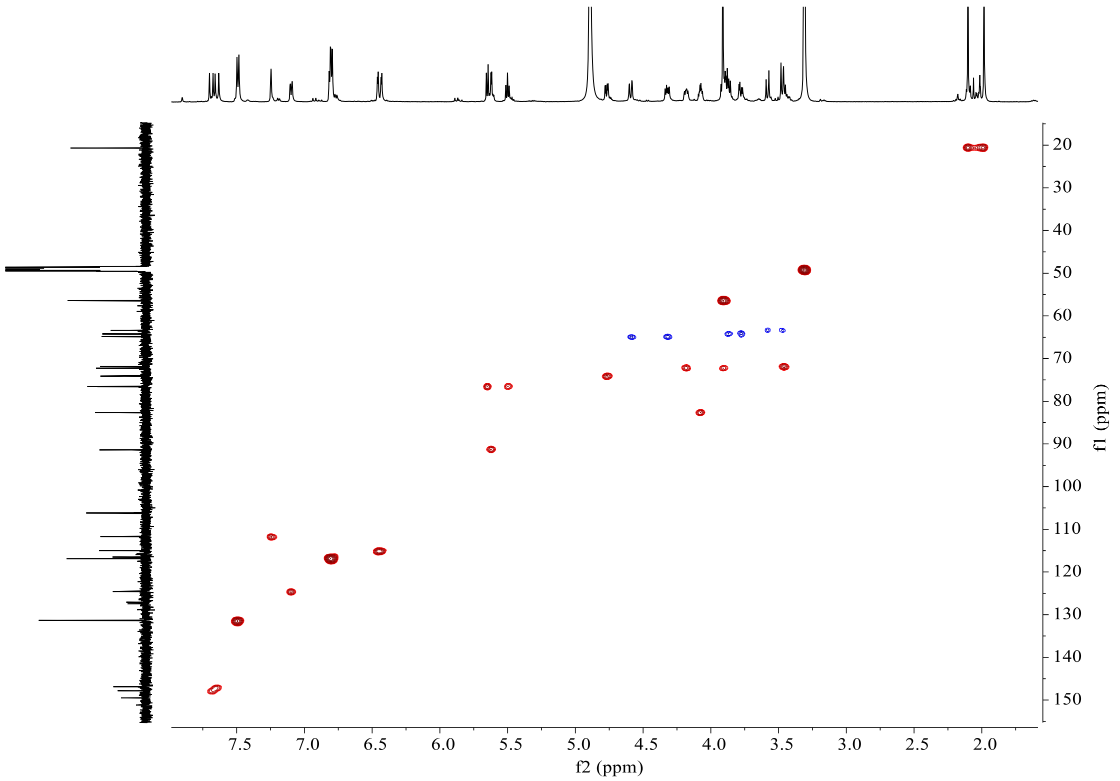

Figure S197. HSQC Spectrum of Compound **22** in MeOH-d_4

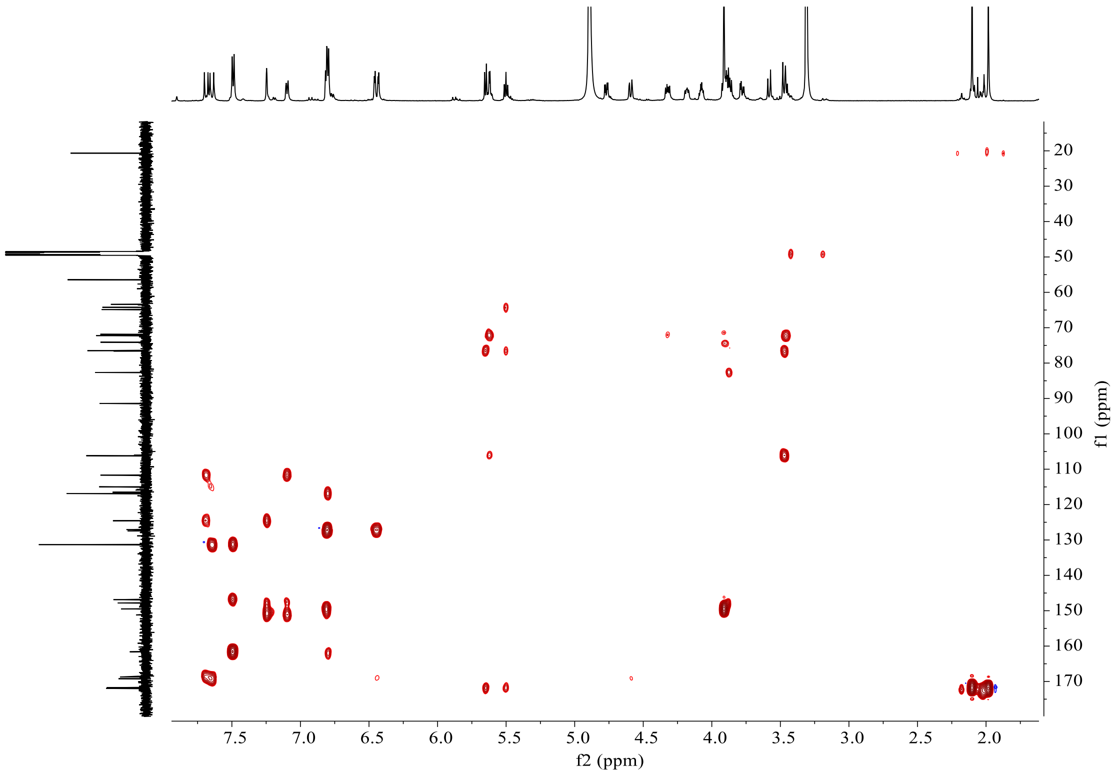

Figure S198. HMBC Spectrum of Compound **22** in MeOH-d_4

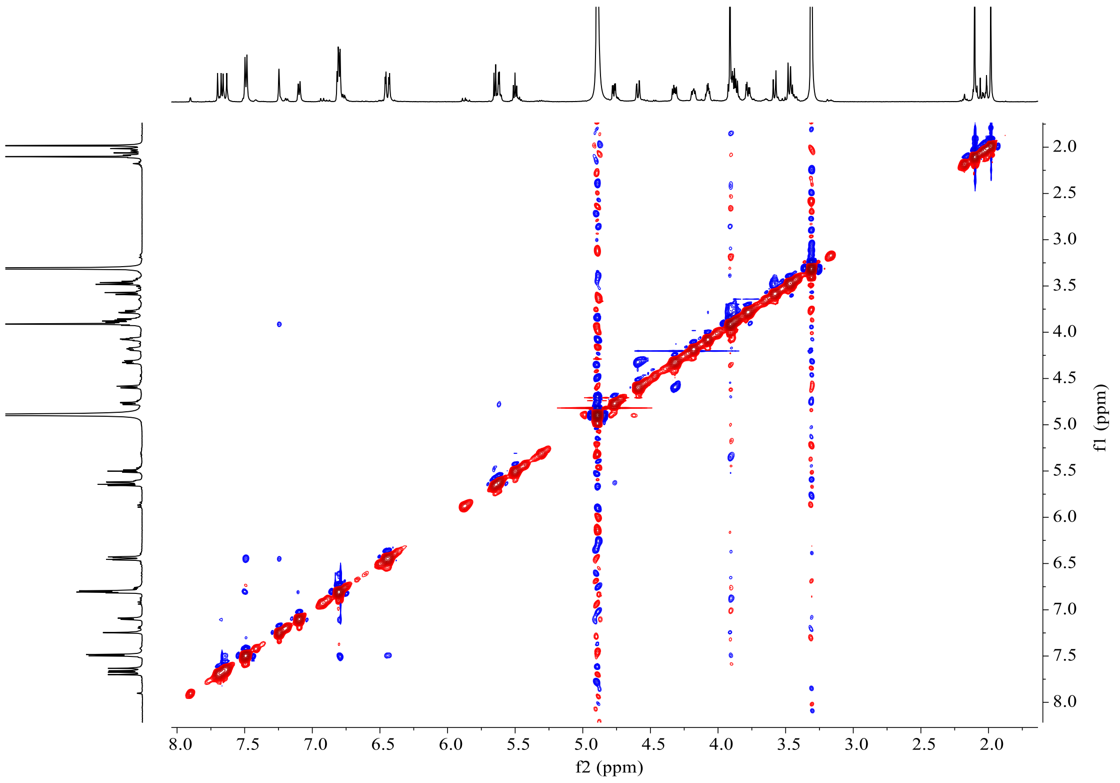

Figure S199. NOESY Spectrum of Compound **22** in MeOH-d_4

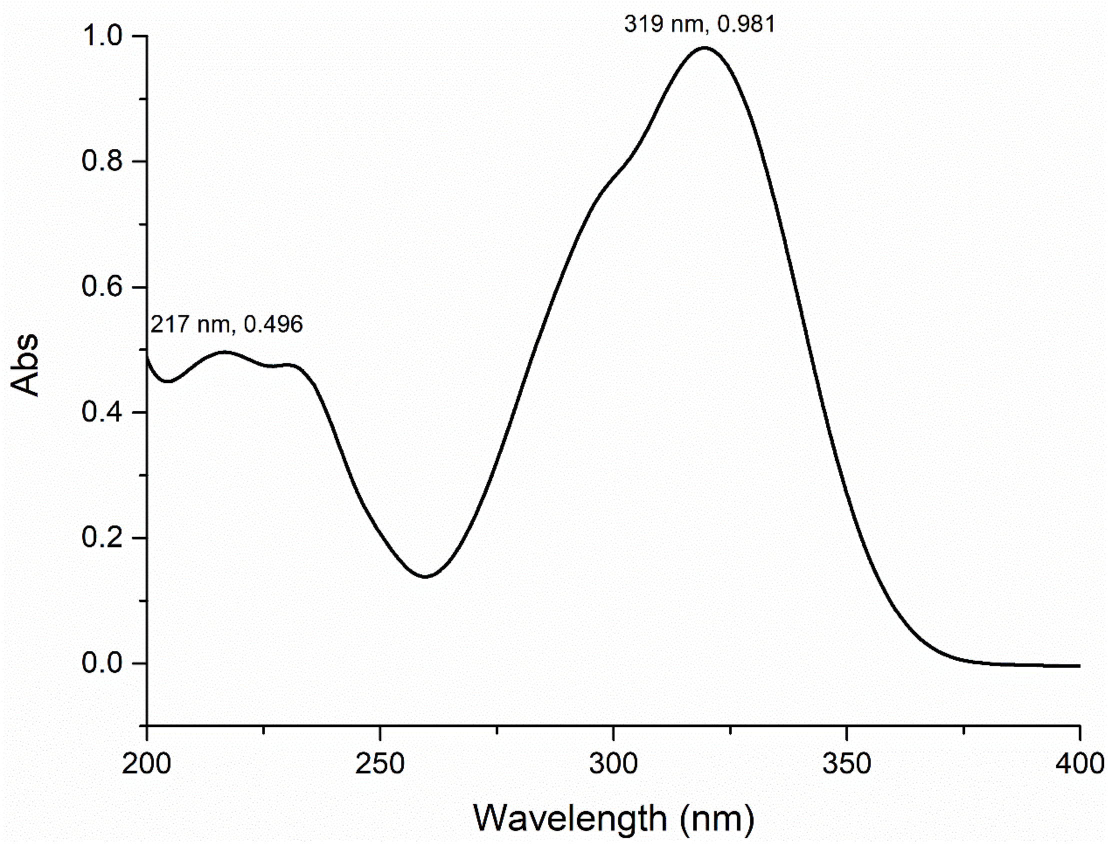

Figure S200. UV Spectrum of Compound **23**

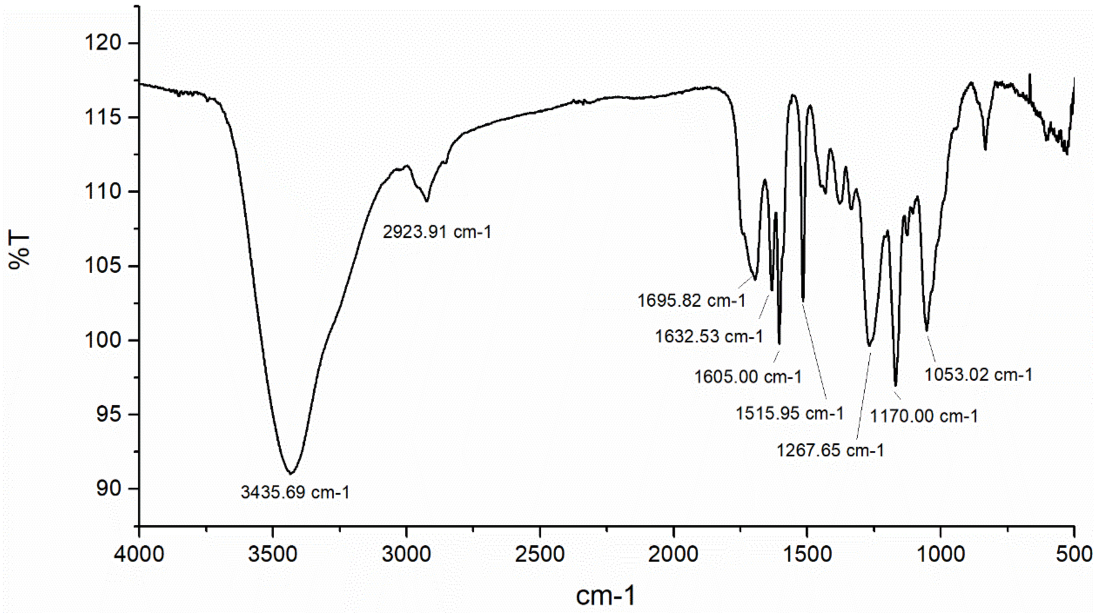

Figure S201. IR (KBr disc) Spectrum of Compound **23**

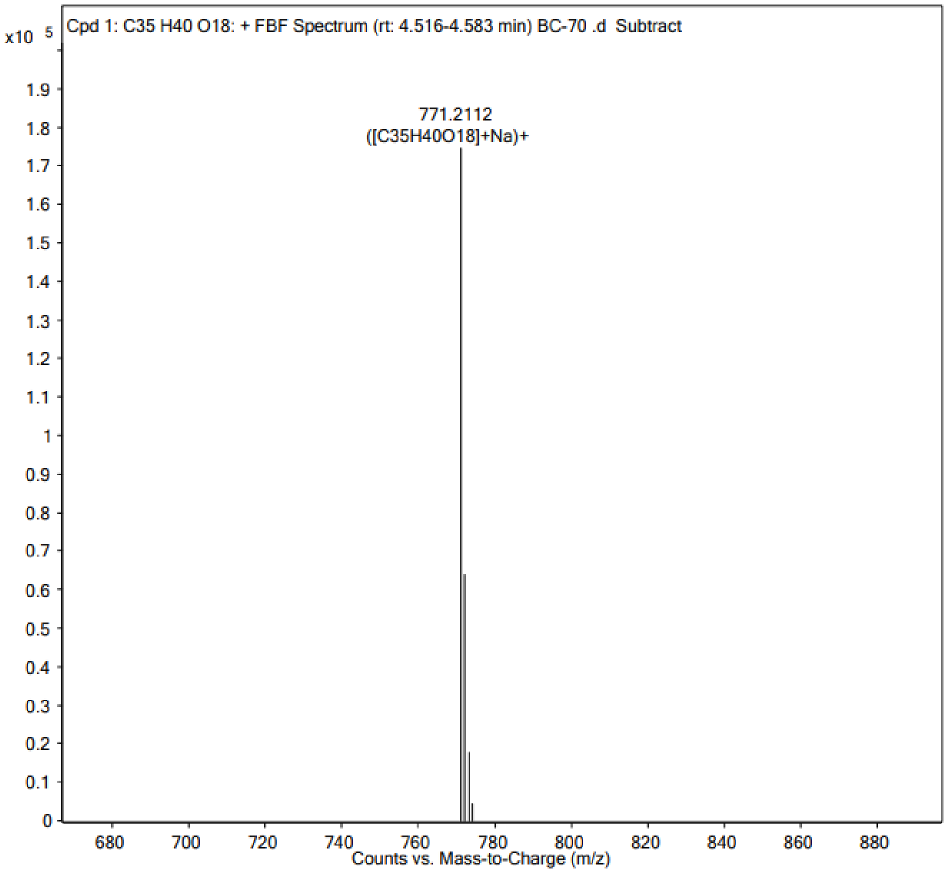

Figure S202. (+) HRESIMS Spectrum of Compound **23**

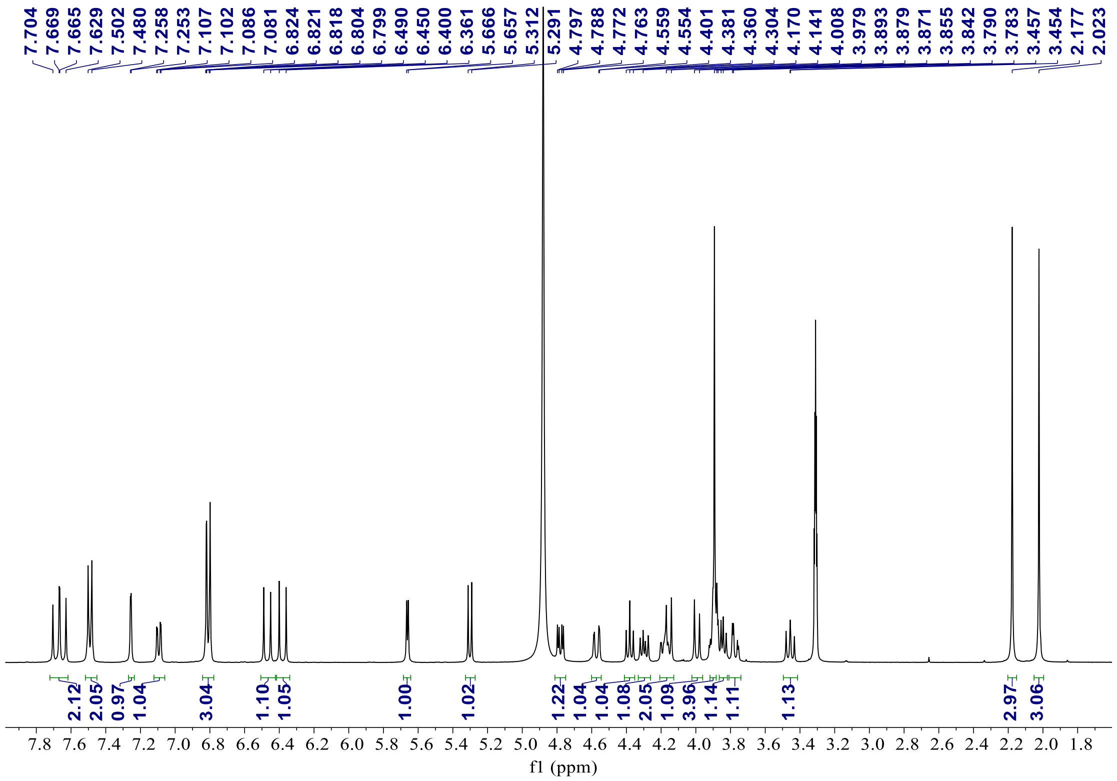

Figure S203. ^{1}H NMR Spectrum of Compound **23** in MeOH-d_4

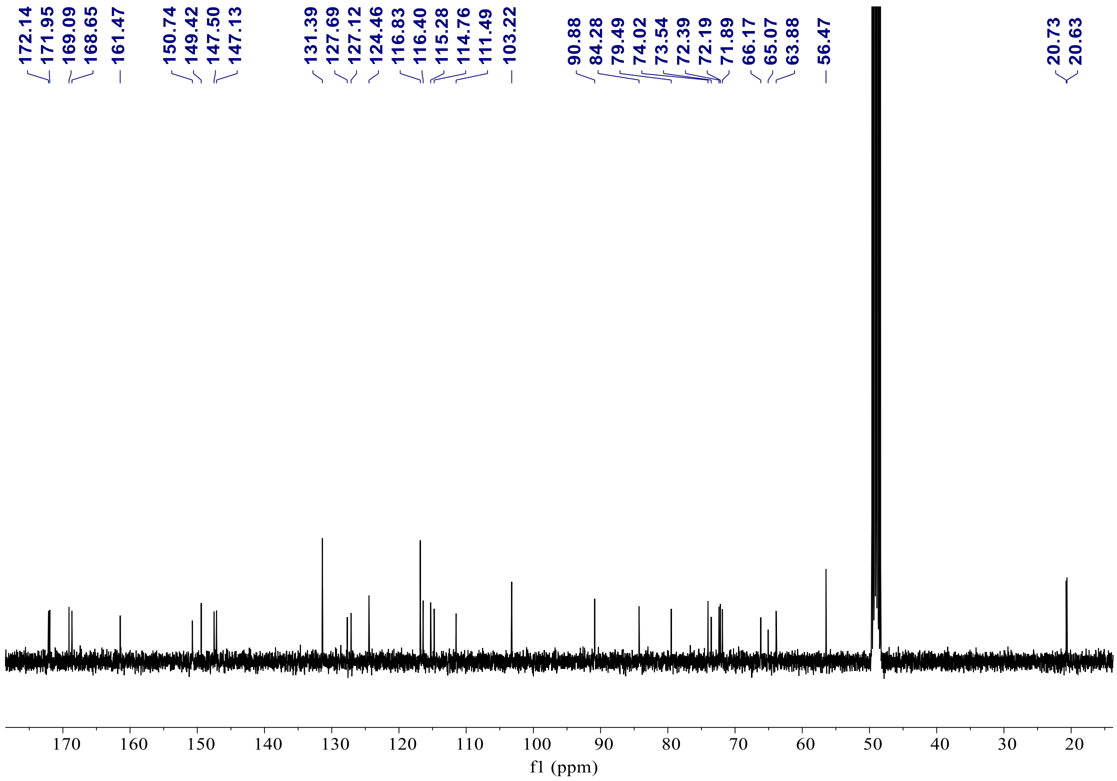

Figure S204. ^{13}C NMR Spectrum of Compound **23** in MeOH-d_4

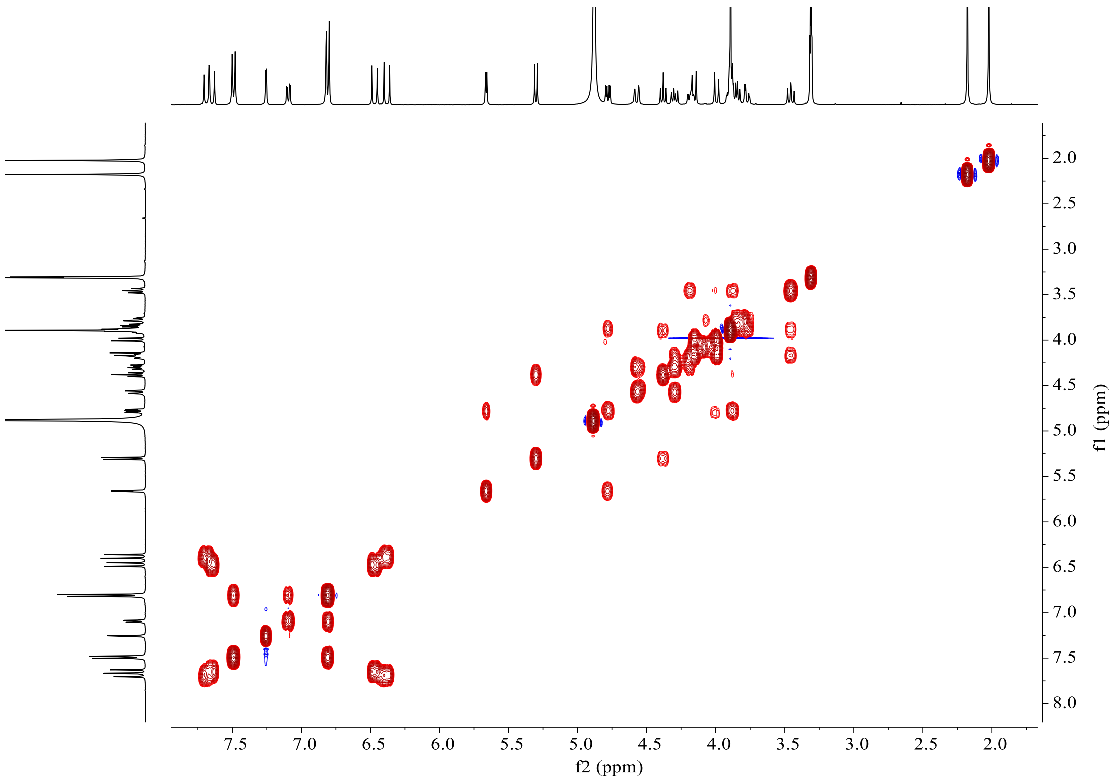

Figure S205. ^{1}H–^{1}H COSY Spectrum of Compound **23** in MeOH-d_4

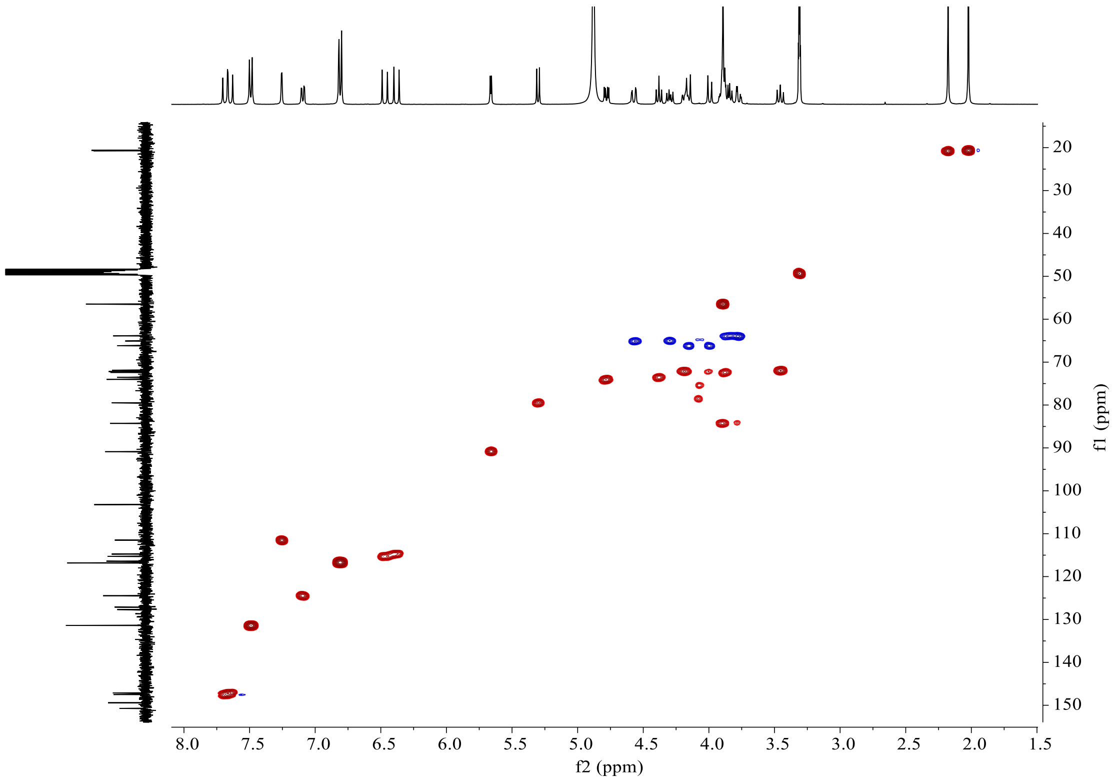

Figure S206. HSQC Spectrum of Compound **23** in MeOH-d_4

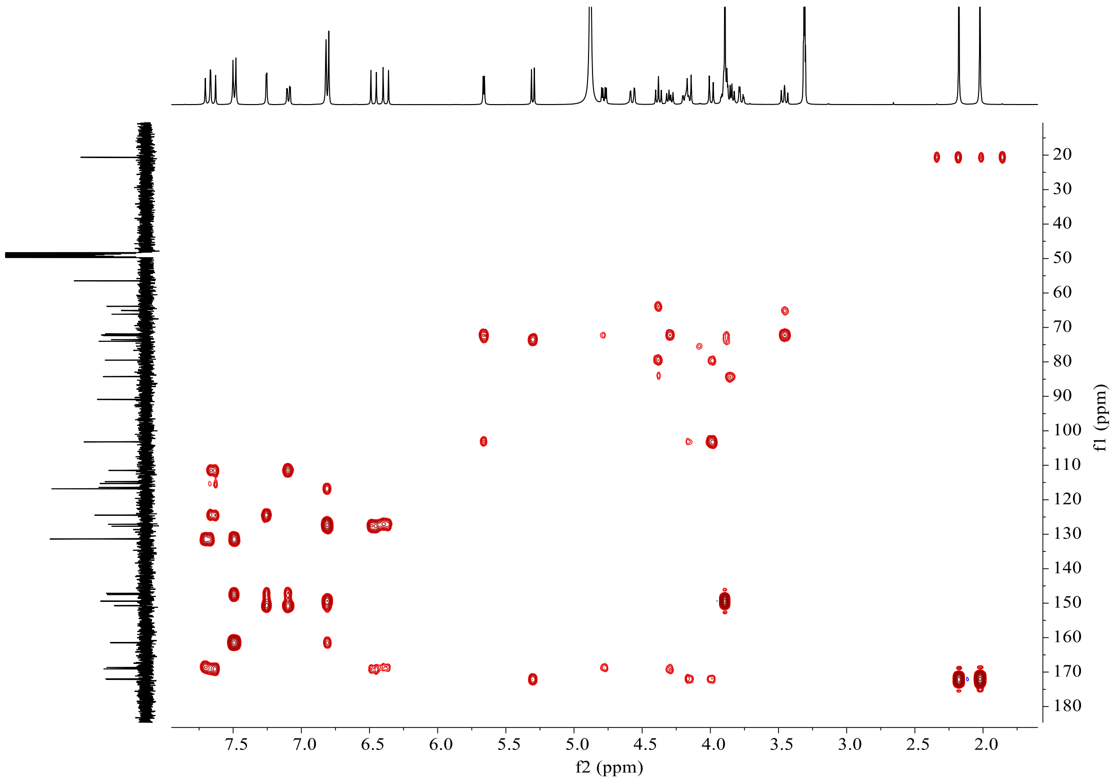

Figure S207. HMBC Spectrum of Compound **23** in MeOH-d_4

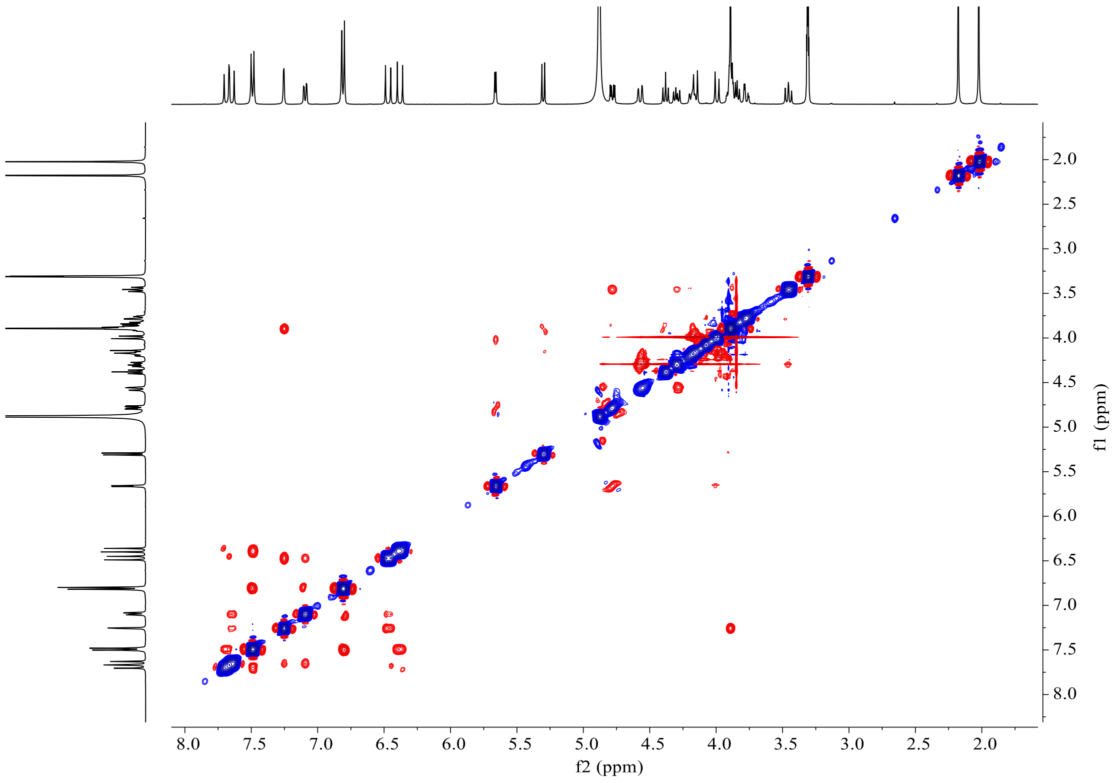

Figure S208. NOESY Spectrum of Compound **23** in MeOH-d_4

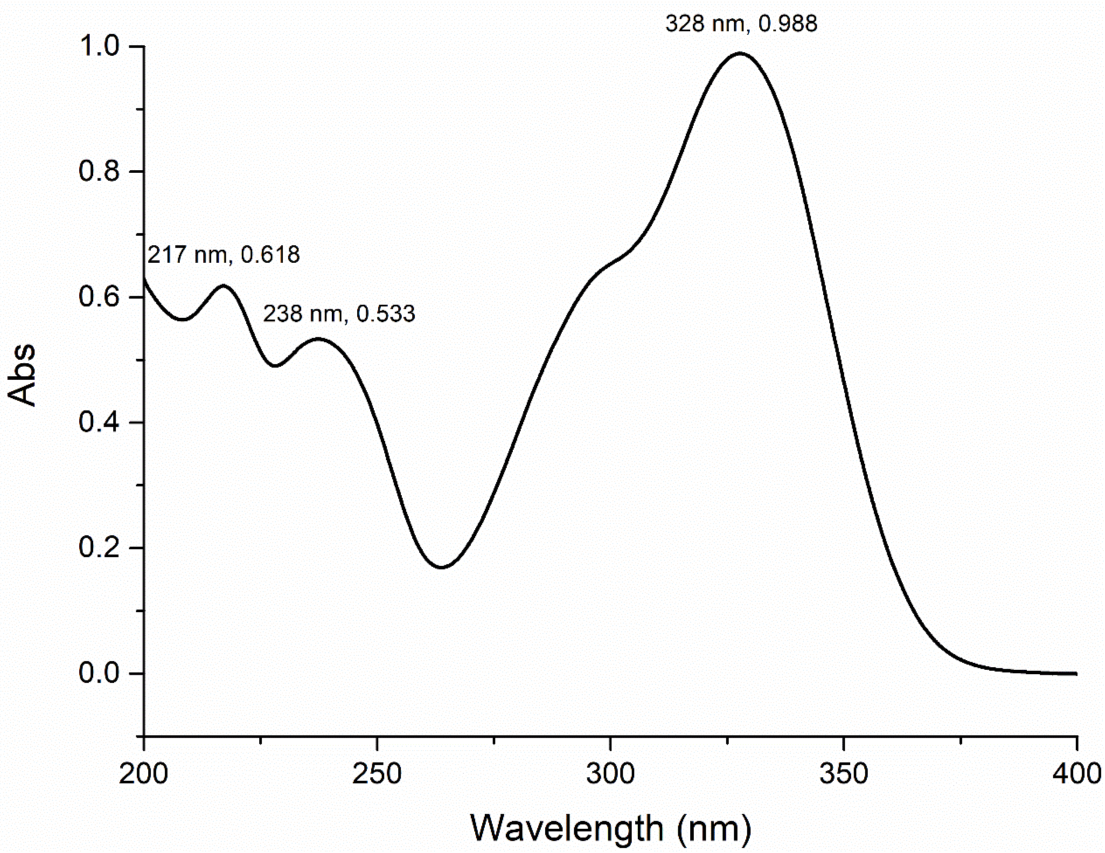

Figure S209. UV Spectrum of Compound **24**

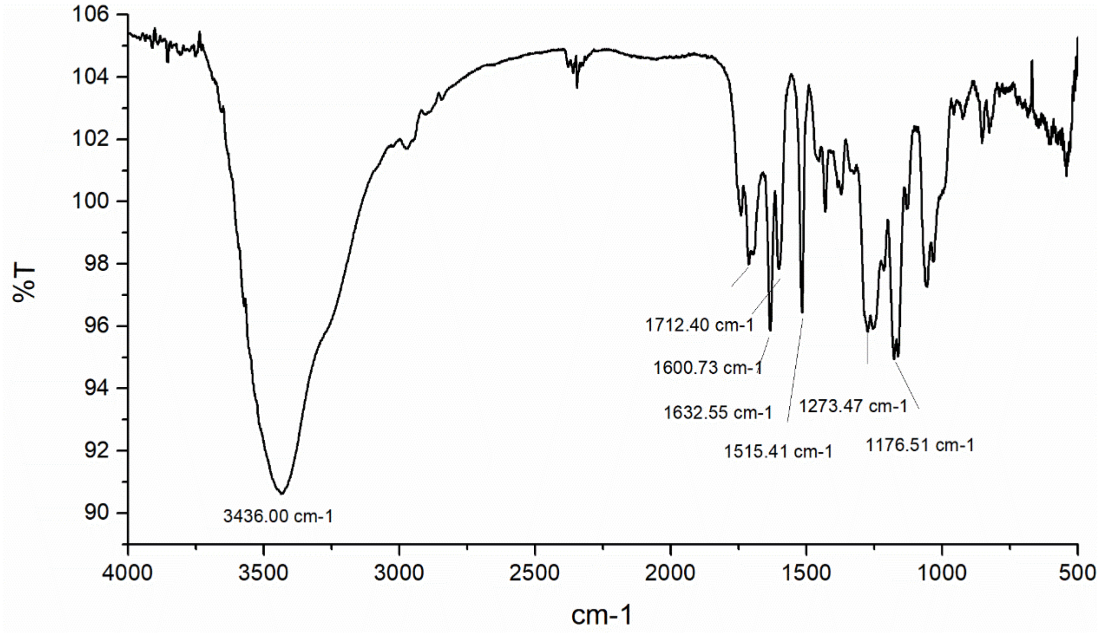

Figure S210. IR (KBr disc) Spectrum of Compound **24**

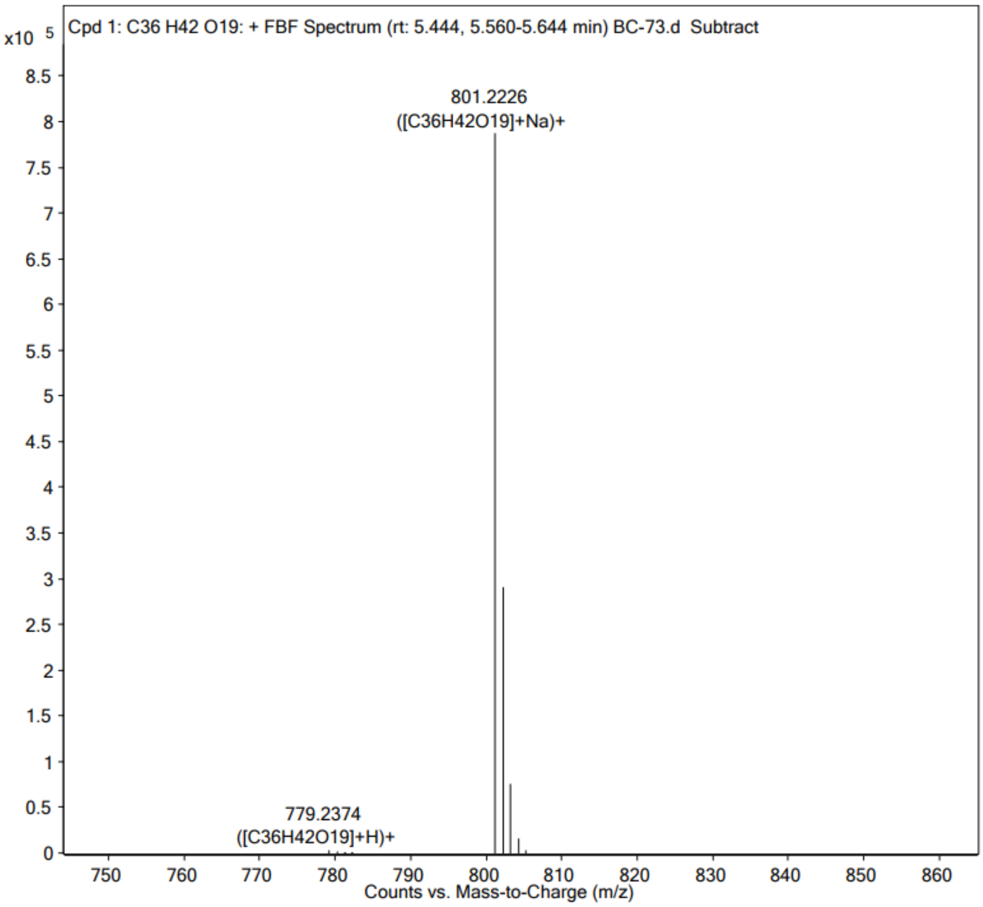

Figure S211. (+) HRESIMS Spectrum of Compound **24**

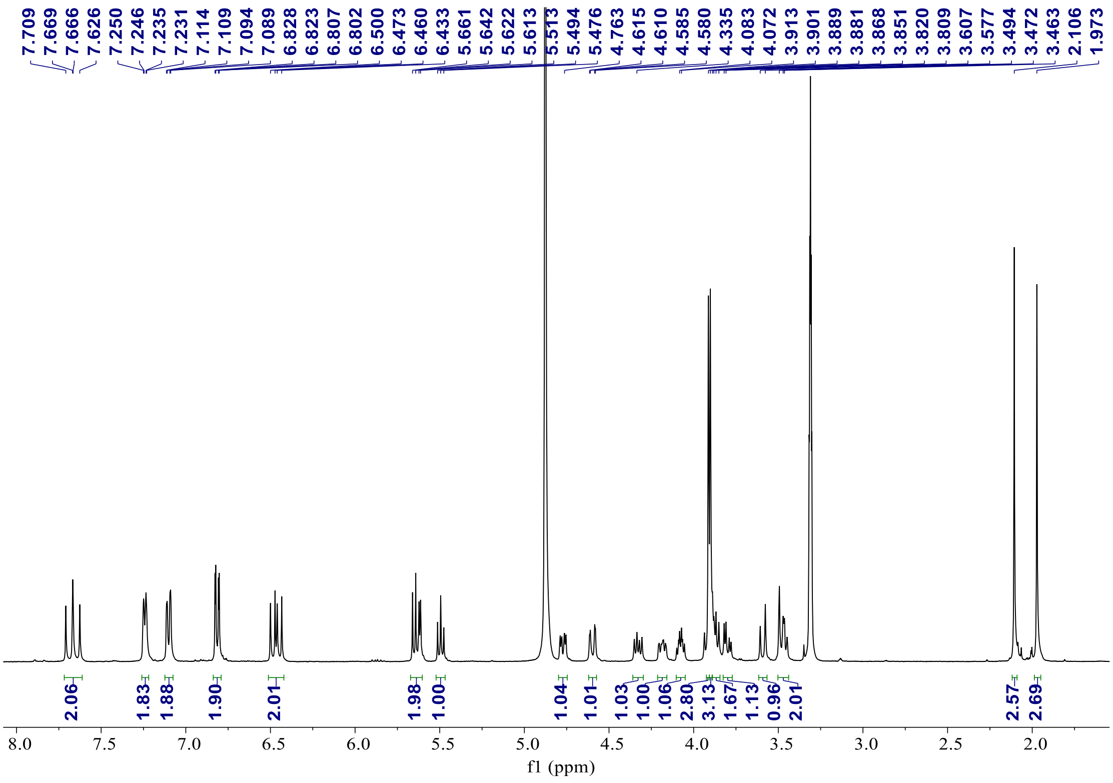

Figure S212. ^{1}H NMR Spectrum of Compound **24** in MeOH-d_4

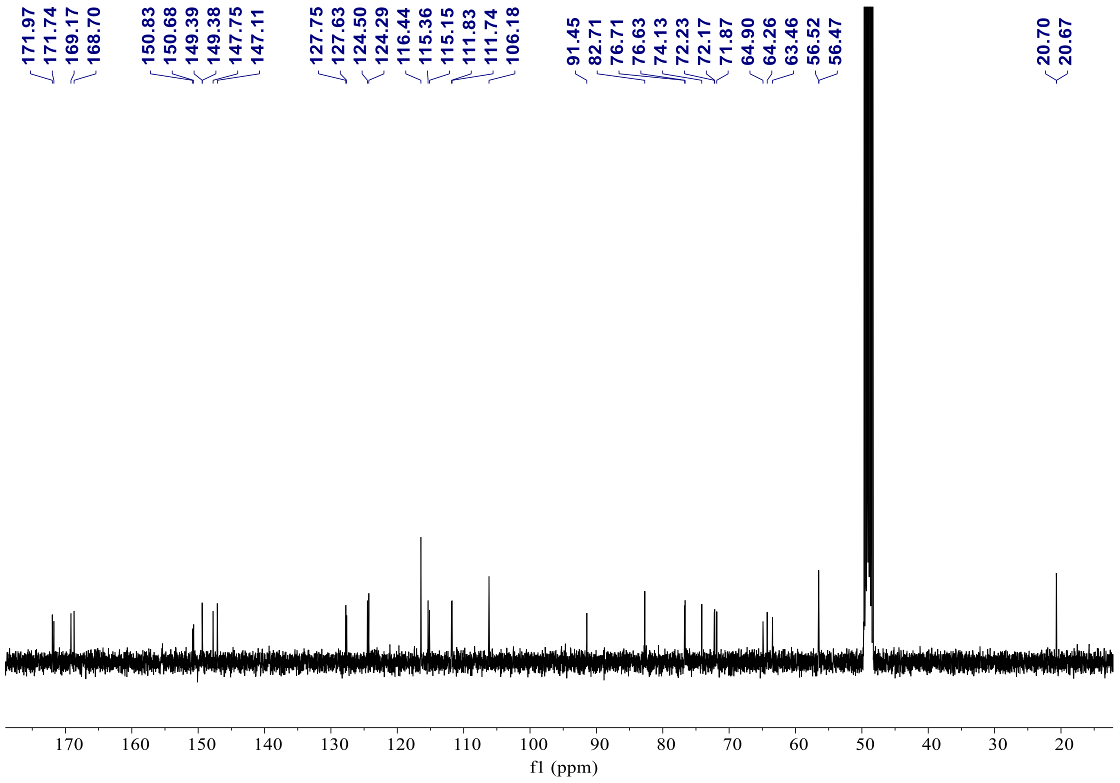

Figure S213. ^{13}C NMR Spectrum of Compound **24** in MeOH-d_4

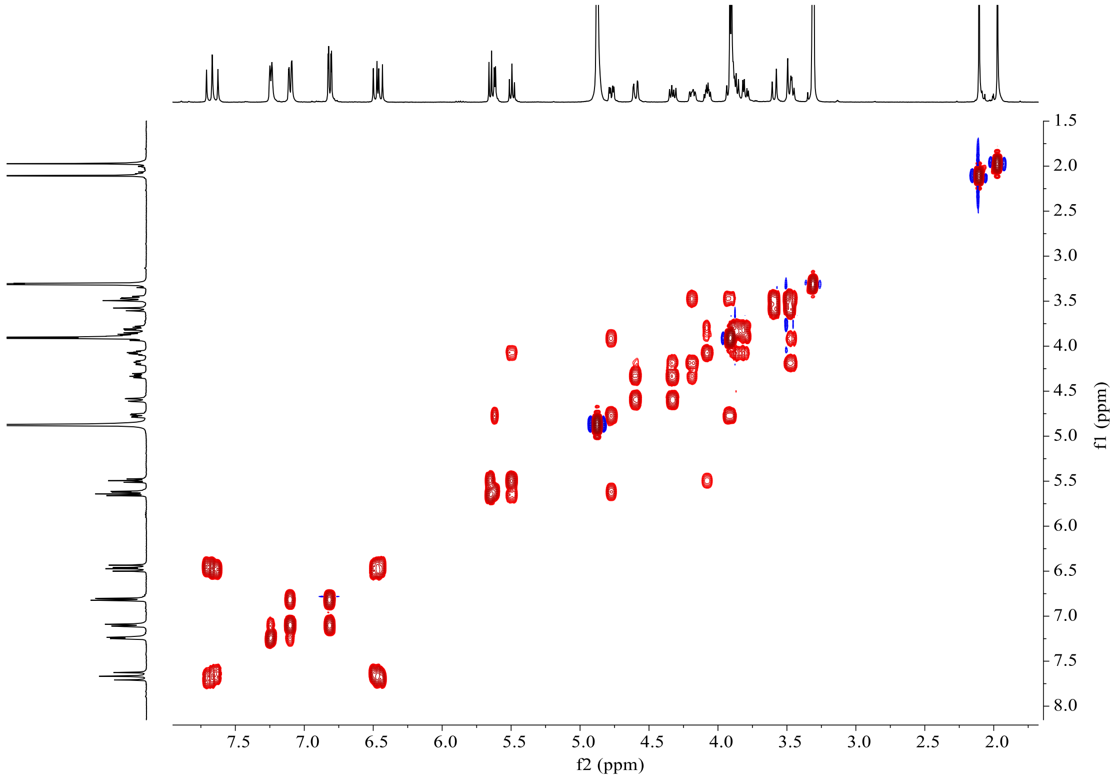

Figure S214. ^{1}H–^{1}H COSY Spectrum of Compound **24** in MeOH-d_4

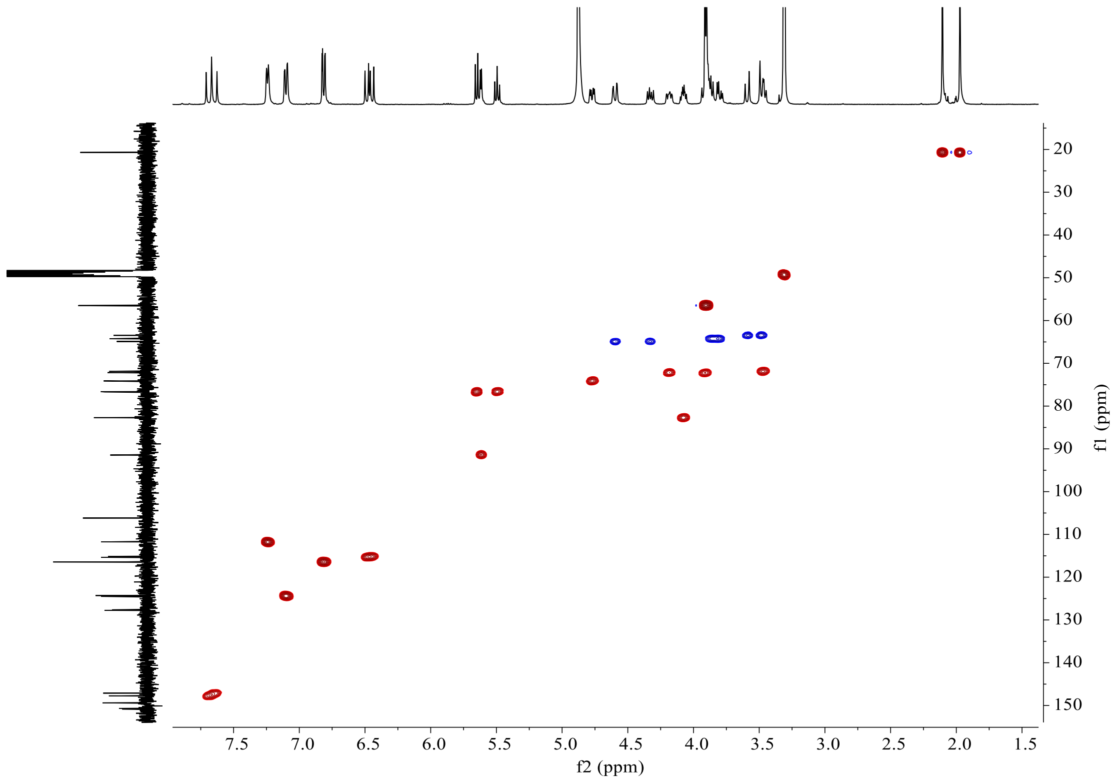

Figure S215. HSQC Spectrum of Compound **24** in MeOH-d_4

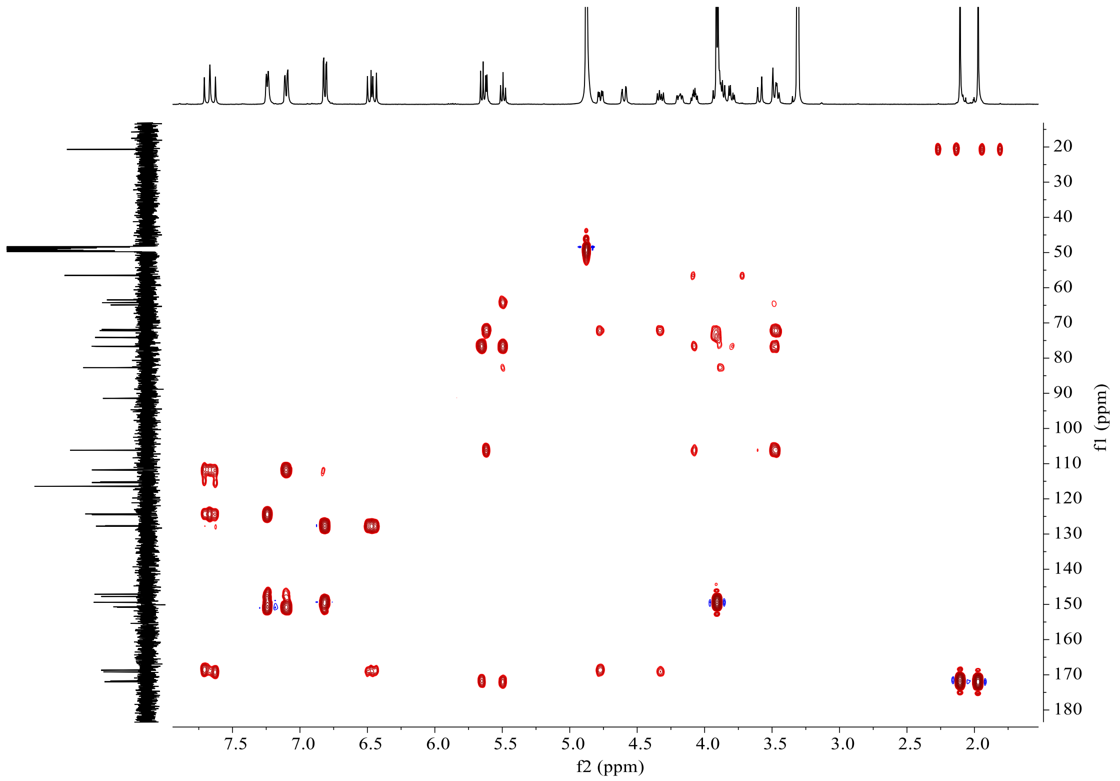

Figure S216. HMBC Spectrum of Compound **24** in MeOH-d_4

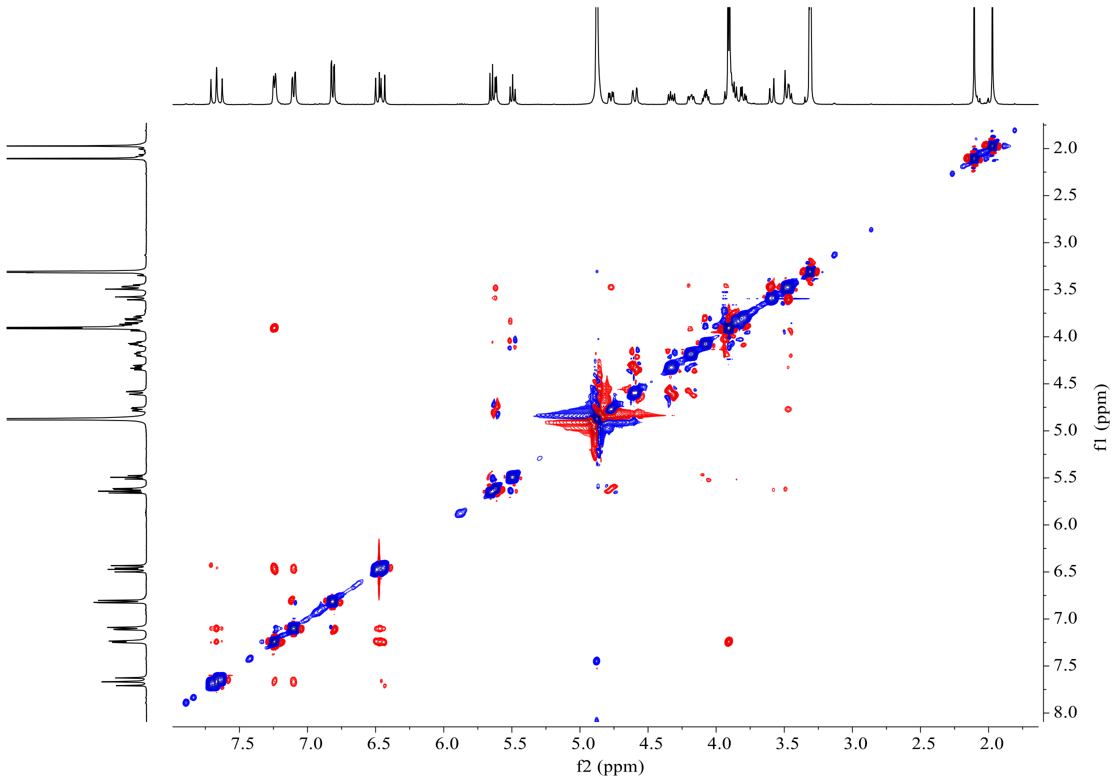

Figure S217. NOESY Spectrum of Compound **24** in MeOH-d_4
